# Plus Sutures for preventing surgical site infection: a systematic review of clinical outcomes with economic and environmental models

**DOI:** 10.1186/s12893-023-02187-0

**Published:** 2023-10-03

**Authors:** M. Edwards, S. Graziadio, J. Shore, N. D. Schmitz, T. Galvain, W. A. Danker, M. Kocaman, D. J. Pournaras, D. M. Bowley, K. J. Hardy

**Affiliations:** 1 York Health Economics Consortium, University of York, Enterprise House, Innovation Way, York, YO10 5NQ UK; 2 Johnson & Johnson MEDICAL GmbH, Robert-Koch-Strasse 1, 22851 Norderstedt, Germany; 3 Global Health Economics, Johnson & Johnson Medical Devices, New Brunswick, NJ USA; 4 Ethicon Inc., 1000 US-202, Raritan, NJ 08869 USA; 5 Johnson & Johnson Medical Limited, Berkshire, UK; 6 Department of Bariatric and Metabolic Surgery, Southmead Hospital, North Bristol NHS Trust, Bristol, UK; 7 University Hospitals Birmingham NHS Foundation Trust, Mindelsohn Way, Edgbaston, Birmingham, B15 2WB UK; 8 Derbyshire Pathology, University Hospitals Derby and Burton NHS Trust, Royal Derby Hospital, Derby, UK

**Keywords:** Triclosan, Antibiotic coating, Sutures, SSI, Surgery, Infection, Systematic review, Meta-analysis

## Abstract

**Background:**

Surgical site infections (SSIs) represent ~ 20% of all hospital-acquired infections in surgical patients and are associated with prolonged hospital stay, admission to intensive care, and mortality. We conducted a systematic review with economic and environmental models to assess whether triclosan-coated sutures (Plus Sutures) provide benefits over non-coated sutures in the reduction of SSI risk.

**Methods:**

Searches were conducted in fifteen databases. A total of 1,991 records were retrieved. Following deduplication and screening by two independent reviewers, 31 randomized controlled trials in adults and children were included in the review.

Similarity of the studies was assessed by narrative review and confirmed by quantitative assessment. A fixed effects meta-analysis of SSI incidence model including all groups of patients estimated a risk ratio of 0.71 (95% confidence interval: 0.64 to 0.79) indicating those in the Plus Sutures group had a 29% reduction in the risk of developing an SSI compared with those in the control group (*p* < 0.001). Safety outcomes were analysed qualitatively.

**Results:**

The economic model estimated the use of Plus Sutures to result in average cost savings of £13.63 per patient. Plus Sutures remained cost-saving in all subgroup analyses with cost-savings ranging between £11 (clean wounds) and £140 (non-clean wounds).

The environmental impact of SSI is substantial, and the model suggests that the introduction of Plus Sutures could result in potential environmental benefits.

**Conclusions:**

The evidence suggests that Plus Sutures are associated with a reduced incidence of SSI across all surgery types alongside cost savings when compared with standard sutures.

**Supplementary Information:**

The online version contains supplementary material available at 10.1186/s12893-023-02187-0.

## Background

Surgical site infections (SSIs) represent around 20% of all hospital-acquired infections in patients undergoing surgery and are associated with prolonged hospital stay and increased risk of admission to intensive care, morbidity and mortality [1, 2]. Alongside the negative impact on patients, SSI poses a significant financial and resource burden for hospitals. In the United States, the annual cost to the health care system of treating SSI has been estimated to be $3.3 billion [3], and a recent study estimated the additional hospital inpatient cost associated with SSI at between $3.7 and $5.5 billion [4]. In the UK, the average cost of managing an SSI is reported to be over £6,000 (accounting for inflation) [5] for National Health Service (NHS) hospitals. These costs do not include any potential litigation costs arising from acquisition of SSI during a hospital stay [6].

SSI is multifactorial and it has been estimated that 40% to 60% of cases may be preventable [7, 8]. Reducing the risk of SSI requires an evidence-based surgical care bundle approach, including management of patient risk factors for infection, appropriate skin antisepsis, instrument sterilisation, environmental control within the operating theatre, infection prevention and control measures, and antibacterial devices [9, 10].

Suture material is a risk factor for SSI. Bacteria can colonize on the suture developing a polymicrobial biofilm which increases the likelihood of an SSI [11–17]. Plus Antibacterial Sutures (Ethicon, Johnson & Johnson Medical Ltd), a range of synthetic, absorbable sutures, were developed to address this risk factor. Plus Sutures are coated with medical-grade triclosan, IRGACARE® MP, a broad-spectrum antibacterial agent that actively inhibits the colonization of bacteria on the suture and is effective against the most common organisms associated with SSI [18–20]. The use of triclosan-coated sutures to prevent SSIs has been recommended internationally by the World Health Organization (WHO), the National Institute for Health and Care Excellence (NICE) in the UK, the European Network for Health Technology Assessment (EUnetHTA), the Centers for Disease Control (CDC), the American College of Surgeons and the Surgical Infection Society, the Canadian Agency for Drugs and Technologies in Health (CADTH), and the Australian Guidelines for the Prevention and Control of Infection in Healthcare, among others [9, 10, 21–23].

The aim of this work was to synthesise the available published evidence around Plus Suture to prevent SSI in terms of clinical outcomes and estimate the effectiveness in terms of cost and environmental impact. This review and analyses also formed part of a submission for Plus Sutures to the NICE Medical Technologies Evaluation Programme (MTEP) [24]. NICE is an executive non-departmental public body that produces guidelines and advice for commissioners, practitioners and managers throughout the NHS and other public health and social care services in the UK [25]. As part of the MTEP process, the findings presented here were reviewed and corroborated by independent reviewers based at an External Assessment Centre [24].

## Methods

The population was adults and children needing wound closure after a surgical procedure in any country, in whom absorbable sutures are an appropriate option.

The intervention was triclosan-coated sutures, with a focus on the Plus Antibacterial Sutures range that included:

- ▪ Coated VICRYL™ Plus Antibacterial (polyglactin 910) Suture, a synthetic absorbable multifilament suture (multiple braided threads).
- ▪ MONOCRYL™ Plus Antibacterial (poliglecaprone 25) Suture, a synthetic absorbable monofilament suture (solid and smooth thread).
- ▪ PDS™ Plus Antibacterial (polydioxanone) Suture, a synthetic absorbable monofilament suture (solid and smooth thread).
- ▪ STRATAFIX™ Knotless Tissue Control Devices, a barbed suture material to allow tissue approximation without the need to tie surgical knots. The STRATAFIX range includes the:
  - STRATAFIX™ Symmetric PDS Plus Knotless Tissue Control Device.
  - STRATAFIX™ Spiral PDS Plus Knotless Tissue Control Device.
  - STRATAFIX™ Spiral MONOCRYL Plus Knotless Tissue Control Device.

Studies that did not disclose the specific triclosan-coated suture product assessed or used “triclosan-coated sutures” as a generic term, were also eligible for inclusion. 

The comparator was sutures that do not contain an antibacterial agent. This was intended to capture all studies comparing against sutures used in clinical practice across the wide range of geographical settings eligible for inclusion in the review.

The outcomes of interest as defined in the review protocol were:

- ▪ Incidence of SSI, as defined by authors of the included studies (primary outcome).
- ▪ Length of post-operative stay in hospital relating to SSI.
- ▪ Readmission related to SSI, as reported in the included studies.
- ▪ Antibiotics use for SSI (including prescription, duration and dose).
- ▪ Severity of SSI using any validated scoring systems such as ASEPSIS (additional treatment, serous discharge, erythema, purulent exudate, separation of tissues, isolation of bacteria, stay duration as an inpatient) wound score.
- ▪ Device-related adverse events.

### Systematic review methods

This systematic review was undertaken following the principles of systematic reviewing embodied in the Cochrane handbook [26] and is reported following the Preferred Reporting Items for Systematic Reviews and Meta-Analyses (PRISMA) guidelines [27]. The scope of the review was guided by the decision problem published by NICE for the health technology assessment (HTA) of Plus Sutures in the UK [24].

The PRISMA checklist is presented in Supplementary Sect. 8. The review protocol was registered on the Open Science Foundation database to ensure transparency [28]. Full details of the eligibility criteria are presented in Table 1.

**Table 1:** Eligibility criteria for the systematic review

	**Inclusion Criteria**	**Exclusion Criteria**
Population	• Studies in adults and children in whom Plus Sutures (including Stratafix Plus) are an appropriate option • Studies assessing sutures for wound closure following an invasive surgical procedure Population subgroups of interest are as follows: • Adults • Children • Clean wound procedures • Non-clean wound procedures	• Participants with a known allergy to triclosan or contraindicated for the use of Plus Sutures • Studies assessing sutures for wound closure in settings other than invasive surgery
Intervention	Plus Sutures (Ethicon, Johnson & Johnson Medical Ltd): • PDS Plus Antibacterial (polydioxanone) Suture • MONOCRYL Plus Antibacterial (poliglecaprone 25) Suture • Coated VICRYL Plus Antibacterial (polyglactin 910) Suture • STRATAFIX Symmetric PDS Plus Knotless Tissue Control Device • STRATAFIX Spiral PDS Plus Knotless Tissue Control Device • STRATAFIX Spiral MONOCRYL Plus Knotless Tissue Control Device Studies assessing “triclosan-coated sutures” that do not refer to a brand name, will also be eligible	• Studies of any sutures other than the named eligible technologies • Studies of mixed eligible and ineligible interventions where results are not disaggregated according to suture variety or variant, i.e. studies where some patients in the intervention group receive one or more of the named Plus Sutures, and the remaining patients in the intervention group receive an ineligible intervention
Comparators	Standard of care, i.e.: • Sutures without any antibacterial coating	• Other sutures with an antibacterial coating, including other types of Plus Suture
Outcomes	• Incidence of SSI • Antibiotic use for SSI • Hospital stay related to SSI ◦ Length of post-operative stay in hospital relating to SSI ◦ Rate of readmission related to SSI • Severity of SSI, as reported by study authors, including ASEPSIS (additional treatment, serous discharge, erythema, purulent exudate, separation of tissues, isolation of bacteria, duration of stay as an inpatient) wound score • Device-related adverse events *Outcomes added to the scope at a later date were not specified in the protocol but were summarised with a narrative synthesis from the studies included based on the criteria detailed in this table*	Any other outcomes
Study design	• RCTs of any design	Any studies other than RCTs, including intraindividual trials
Limits	• Full text documents or clinical trial records containing results for at least one outcome of interest to this review • Records of ongoing trials (to be listed for information rather than data extracted) • Otherwise relevant clinical trial records, detailing completed trials for which no results are available (to be listed in the section for relevant unpublished data rather than data extracted) • Only studies with a publication date of 2000 and onwards • English language publications	• Full text publications of studies with a publication date of 1999 or earlier • Clinical trials with a completion date of 1999 or earlier • Studies published in languages other than English

*RCT* randomised controlled trial, *SSI* surgical site infection

#### Searches for the systematic review

The searches for the NICE submission were conducted between 01 February 2021 and 08 February 2021 in multiple databases and information resources (see Table 2). These included: databases covering biomedical healthcare and nursing journal literature; databases of controlled trials, systematic reviews and health technology assessments; databases containing conference abstracts; and databases containing information on ongoing trials. The full search strategy for MEDLINE (Supplementary Fig. 1) was translated appropriately for the other databases (Supplementary Appendix A).

**Table 2:** Databases and information sources searched

Database / Information Source	Interface / URL
MEDLINE ALL	OvidSP
Embase	OvidSP
CINAHL Complete	EBSCOhost
Cochrane Central Register of Controlled Trials	Cochrane Library / Wiley
Cochrane Database of Systematic Reviews	Cochrane Library / Wiley
Database of Abstracts of Reviews of Effects (DARE)	https://www.crd.york.ac.uk/CRDWeb
NHS Economic Evaluation Database (NHS EED)	https://www.crd.york.ac.uk/CRDWeb
HTA Database	https://www.inahta.org/hta-database/
Econlit	OvidSP
Conference Proceedings Citation Index – Science (CPCI-S)	Web of Science
Epistemonikos	https://www.epistemonikos.org/en/
ClinicalTrials.gov	https://clinicaltrials.gov/ct2/home
WHO International Clinical Trials Registry Portal (ICTRP)	http://apps.who.int/trialsearch/Default.aspx
National Institute for Health Research (NIHR) Be Part of Research	https://bepartofresearch.nihr.ac.uk/
IDEAS	https://ideas.repec.org/

Searches of economic databases were included because search results were also considered for use in the cost-effectiveness model of Plus Sutures.

Reference lists of any relevant systematic reviews published in the last five years were checked for any eligible studies that may have been missed by the database searches, and the manufacturer provided details of any ongoing or unpublished trials with which they were associated.

#### Screening, selection and data extraction

Results were downloaded into Endnote bibliographic software [29], deduplicated using several algorithms, and the duplicate references held in a separate EndNote database. A single researcher assessed the search results according to their relevance in providing information on the clinical efficacy and safety of the intervention and comparator and removed the obviously irrelevant records.

Two reviewers independently assessed the titles and abstracts of remaining records for relevance against the eligibility criteria, with disagreements adjudicated by a third reviewer. Assessment of full texts was then conducted by the same three reviewers; two reviewers independently assessed the records, and the third reviewer adjudicated any disagreements.

The first reviewer extracted data from the eligible studies and the second reviewer quality checked all the data points against the papers to ensure accuracy. Data were extracted as reported by study authors, with calculations performed only where the required data were not presented in the format required for the meta-analyses. Calculations were minimal and were based only on reported data.

As recommended by Cochrane guidance [30], timepoints at which data were to be extracted were specified prior to starting the review. One timepoint per study was extracted (the optimum timepoint for each surgery type was either 30 or 90 days post-surgery); if a paper reported data at more than one timepoint, CDC guidance [3] was used to select the most appropriate timepoint.

The NICE Risk of Bias tool [31] was used to quality assess each of the included studies, with one researcher completing the assessment and a second verifying this. This tool considers seven different criteria.

#### Synthesis

A high-level assessment of the similarity of studies and availability of data was performed, guided by the Australian Pharmaceutical Benefits Advisory Committee criteria [32]. Where meta-analysis was possible, i.e. sufficient homogenous studies reported suitable data, statistical methods were used to analyse and summarise the results of the included studies.

For outcomes for which meta-analysis was not possible, a narrative summary exploring the quality of the studies, the relationship between studies and any patterns discerned in the data were presented.

### Meta-analysis methods

Where meta-analysis was deemed appropriate, results were statistically pooled for the outcomes of interest using both fixed- and random-effects models, both of which produced estimated risk ratios. Further details are presented in Supplementary Sect. 2. Robustness of the model chosen, model assumptions and susceptibility to outliers were assessed. Potential sources of heterogeneity were defined a priori and were evaluated through sensitivity and subgroup analyses. All statistical analyses were conducted using R version 4.0.2 [33], with additional R packages meta (v.4.16–2 [34]) and dmetar (v.0.0.9000 [35]). Results were presented as forest plots, and the significance level was set at p ≤ 0.05.

The primary outcome for the meta-analysis was the relative risk (RR) of developing an SSI between the intervention (Plus Sutures) and control group.

#### Subgroup and sensitivity analyses

The base case analysis included all studies that provided sufficient data. Subgroup analysis considered four subgroups as defined in the NICE scope (adults only, children only, patients classified as having clean wounds, and patients classified as having non-clean wounds). Studies in which the authors did not explicitly state wound type were mapped to the subgroups by surgery type. Finally, a sensitivity analysis was carried out removing Stratafix Plus as an intervention to explore its potential effect on heterogeneity.

For the subgroup analyses by wound type, we recorded authors’ descriptions of the status of the wounds assessed in each study. Where the authors did not explicitly report this information, the independent opinion of three clinicians was sought as to the likely wound status following the surgery detailed in each of the studies. The categorisation of the wound status was then compared across the clinicians and any divergence of opinion discussed before a consensus was reached. The decisions reached determined which subgroup analysis each study would contribute to.

### Modelling methods

#### Model structure

**Fig. 1:**
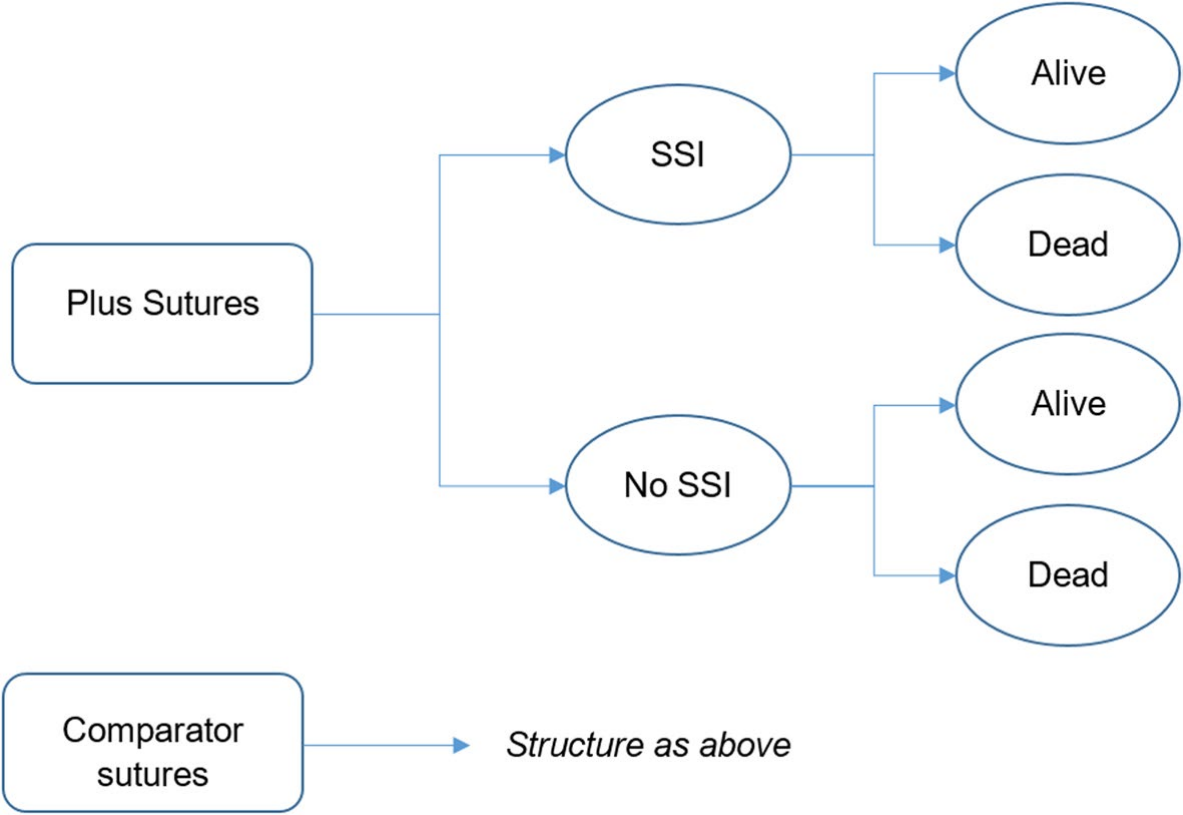
Structure of the model

A decision-tree model was developed to estimate changes in healthcare costs (from the perspective of the NHS) associated with the use of Plus Sutures compared with conventional sutures in people undergoing a surgical procedure. The population considered in the model was all people undergoing a surgical procedure with subgroup analysis conducted for adults (≥ 18 years of age), children, clean and non-clean wounds. Following their surgical procedure people enter the model and receive either Plus Sutures or conventional sutures and then follow the pathway of SSI or no SSI, followed by survival or death. The model structure is presented in Fig. 1.

Patients who developed an incisional SSI accrued additional mortality risk and costs which incorporated hospital readmission, increased length of stay plus other resource use required during SSI treatment. Given that no system changes were anticipated to implement the technology and no additional training was required for health care professionals to use Plus Sutures, no additional resource costs were included in the model.

Adverse events related to sutures were not included in the model because no events were identified from the clinical review that were judged to have a substantial impact on health-related quality of life (HRQoL) or health care resource use. Those reported were considered unlikely to be related to the Plus technology.

The model structure was aligned with the clinical pathway and other SSI models in the published literature as well as those developed or assessed by NICE, where the risk of SSI in both arms was captured and the cost of this applied [36–45]. The input parameters were not aligned with these studies; rather the best available evidence relevant to the NHS at the time of constructing the model (March 2021) was used instead, to ensure the model was current and relevant to the UK.

#### Model inputs

The model inputs are presented in Supplementary Table 1. In addition to the searches run to inform the systematic review, reports of adverse events associated with the technology were sought via searches of the Manufacturer and User Facility Device Experience (MAUDE) database and Medicines and Healthcare products Regulatory Agency (MHRA) resources. Results of these searches did not inform the systematic review but were used to check for any adverse events not reported in the published literature that might need to be considered in the model.

##### Baseline infection risk

Baseline infection risk of 1.04% with comparator sutures was derived from UK Health Security Agency (previously Public Health England; PHE) [46] surveillance data on SSI. The baseline infection risk of 1.04% was also used for the adult and children subgroups. This was judged to be reasonable based on data from the literature [47–50]. No alternative data were identified that were judged to be more representative of these subgroups.

For the baseline risk of infection in clean (0.8%) and non-clean (6.8%) subgroups the PHE data was also used. Surgical categories were split into those most likely to result in a clean and unclean wound in line with Troughton 2018 et al. [51].

##### Surgical Site Infection risk with Plus Sutures

The post-surgery SSI risk with Plus Sutures was calculated by applying the RR of infection derived from the meta-analysis to the base case SSI risk from PHE. The RR derived from the fixed effects model was used. RRs for each of the subgroups (children, adults, clean wounds and non-clean wounds) were used for each of the subgroup analyses.

##### Cost of SSI

The cost for SSI used in the base case was £6,016 based on Jenks et al. 2014 [5] (inflated from 2011/12 to 2019/20 [52]). This was used in the base case and for the adult and children subgroups. Jenks et al. 2014 reports a different median cost attributable to SSI for each category of surgery. These were used to calculate a cost of SSI for the clean and non-clean subgroups in line with Troughton 2018 [51].

#### Model outputs

The model generated the total per patient costs in each arm over a one-year time horizon. The incremental cost per patient is presented as well as a cost per SSI averted and the cost per death averted.

#### Subgroup and sensitivity analysis

One-way deterministic sensitivity analysis was conducted to explore the impact of varying individual parameters and identify key drivers of the analysis. Threshold/breakeven analysis was conducted around the baseline SSI incidence with comparator sutures, the cost of SSI, the RR reduction of SSI incidence with Plus Sutures, and the number of sutures. A tornado diagram (see Fig. 4) was used to present one-way analysis for all model inputs. Ranges reported were taken from the literature, and where unavailable, clinical opinion or assumptions were used.

Probabilistic sensitivity analysis (PSA) was conducted to explore second order uncertainty in the results using 1,000 iterations to ensure model stability.

The ranges considered in the sensitivity analyses are presented in Supplementary Table 1.

Subgroup analysis was conducted as described throughout for adults, children, clean and non-clean wounds.

### Environmental sustainability model

An environmental sustainability model was developed to evaluate the environmental impact of SSI and the environmental benefits of SSI reductions. The model structure is shown in Supplementary Fig. 2. Patients who developed an SSI were associated with a 10 day longer length of hospital stay, 4.1 additional outpatient appointments, and 22% more A&E visits compared with patients without an SSI [5, 53]. Environmental impact per SSI for the additional length of stay in general and intensive care wards, additional outpatient and A&E visits and associated travel was calculated through the application of the environmental sustainability reference information provided in the Sustainable Care Pathways Guidance [54]. The model inputs are presented in Supplementary Table 2. The environmental impact of antibiotic prescriptions, GP/community care appointments, and community care home visits were not included as these data were not available [5, 46, 51, 53–56]. The model calculated the environmental impact of SSI using three environmental metrics in accordance with the Sustainable Care Pathway Guidance: greenhouse gas (GHG) emissions, freshwater use, and waste generation. Following this, the potential environmental benefit of reduction in SSI with the use of Plus Sutures was computed by applying the RR of infection derived from the meta-analysis to the base case SSI risk from PHE. The sustainability analysis underwent independent validation.

## Results

### Results of the systematic review

**Fig. 2:**
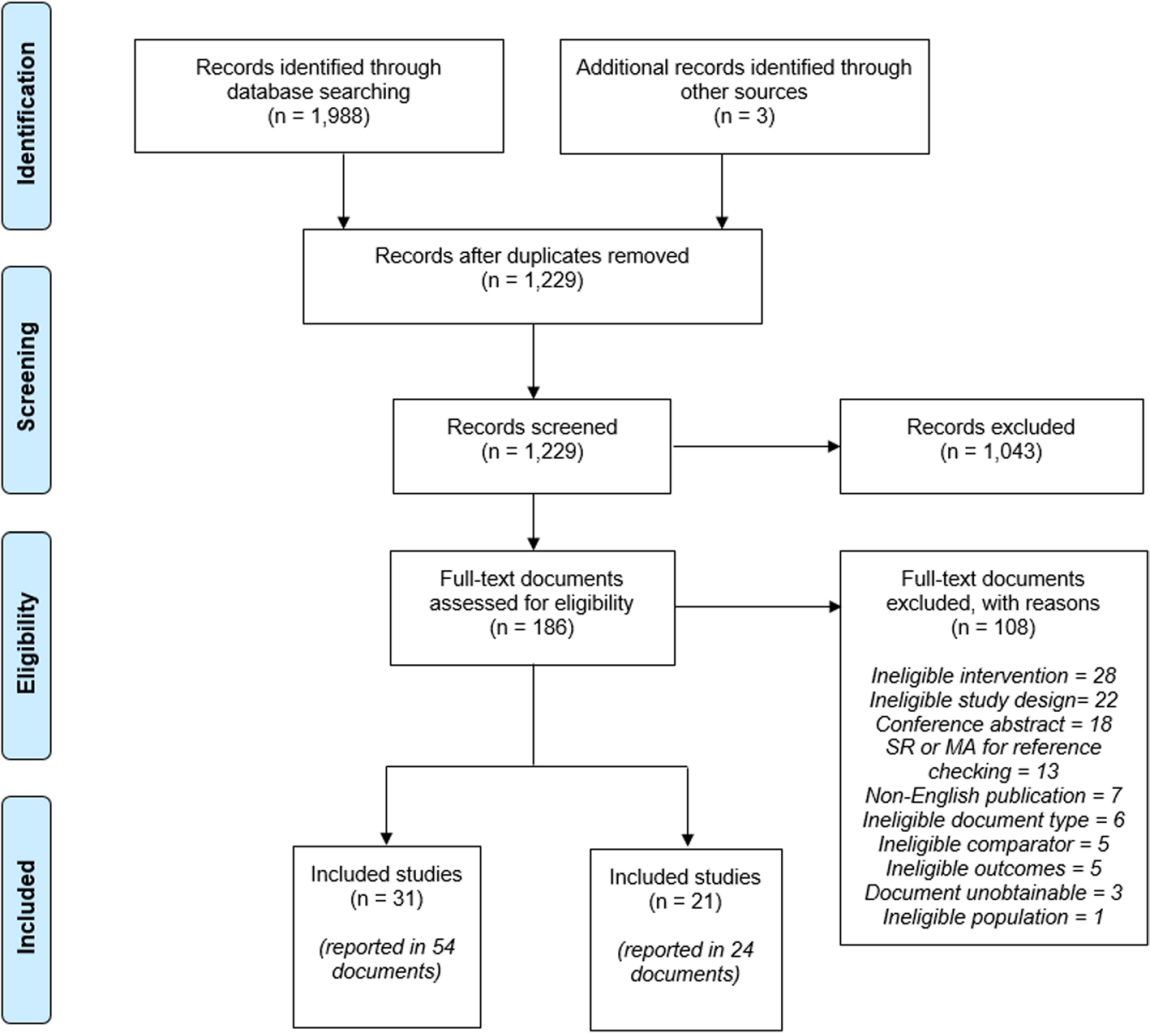
PRISMA flow diagram for the review

The searches retrieved a total of 1,991 records. Following deduplication, 1,229 unique records remained, and 186 publications and trial records proceeded to full text review, which excluded 108 documents (see Supplementary Table 3). Thirty-one studies (reported in 54 documents) were eligible for this review, and a further 21 ongoing or unpublished studies (reported in 24 documents) were listed for information. Figure 2 shows the PRISMA flow diagram, Table 3 presents a summary of the risk of bias assessment, and Supplementary Tables 4 to 7 present full details of the included studies. An overview of the study characteristics of the included studies is presented in Table 4. All but two studies [57, 58] explicitly stated that the sutures assessed were part of the Plus suture range (see Table 4), which uses medical-grade triclosan, IRGACARE® MP.

**Table 3:** Risk of bias assessment of studies included in the review

Study	Was randomisation carried out appropriately?	Was the concealment of treatment allocation adequate?	Were the groups similar at the outset of the study in terms of prognostic factors?	Were the care providers, participants and outcome assessors blind to treatment allocation?		Were there any unexpected imbalances in dropouts between groups? If so, were they explained or adjusted for?	Is there any evidence to suggest that the authors measured more outcomes than they reported?	Did the analysis include an appropriate intention to-treat analysis with appropriate methods used to account for missing data?	Were any other issues observed that might have caused the study to be at risk of bias?	Overall summary assessment of quality
				**Care providers and participants?**	**Outcome assessors?**					
Arslan 2018 [59], Turkey	Unclear	Unclear	Yes	No	Unclear	No unexpected imbalances	No	No	None observed	Methodological concerns
Baracs 2011 [60], Hungary	Yes	No	Yes	Unclear	Unclear	Unclear	Yes	No	None observed	Methodological concerns
Diener 2014 [61], Germany	Yes	Yes	Yes	Yes	Yes	No unexpected imbalances	No	Yes	None observed	High
Ford 2005 [62], USA	Unclear	Unclear	Unclear	No	Unclear	No unexpected imbalances	No	No	Yes	Methodological concerns
Galal 2011 [63], Egypt	Yes	Unclear	Yes	Yes	Yes	No unexpected imbalances	No	Unclear	Yes	Methodological concerns
Ichida 2018 [64], Japan	Unclear	Yes	Yes	Yes	Yes	No unexpected imbalances	No	No	Yes	Methodological concerns
Isik 2012 [65], Turkey	No	Unclear	Yes	Unclear	Unclear	No unexpected imbalances	No	No	None observed	Methodological concerns
Justinger 2013 [66], Germany	Unclear	Unclear	Yes	Yes	Yes	Unclear	Unclear	No	Yes	Methodological concerns
Karip 2016 [67], Turkey	Yes	Unclear	Unclear	Unclear	Yes	No unexpected imbalances	No	Unclear	Yes	Methodological concerns
Lin 2018 [68], Taiwan	Unclear	Yes	Unclear	Yes	Yes	No unexpected imbalances	Yes	Yes	Yes	Methodological concerns
Mattavelli 2015 [69], Italy	Yes	Yes	Yes	No	Yes	No unexpected imbalances	No	No	Yes	Methodological concerns
Mingmalairak 2009 [70], Thailand	Yes	Yes	Yes	Yes	Unclear	No unexpected imbalances	No	Yes	None observed	Unclear
Nakamura 2013 [43], Japan	Unclear	Unclear	Yes	No	Yes	No unexpected imbalances	No	No	Yes	Methodological concerns
Olmez 2019 [71], Turkey	Yes	Unclear	No	Unclear	Yes	No unexpected imbalances	No	No	None observed	Methodological concerns
Rasic 2011 [72], Croatia	Yes	Yes	Yes	Unclear	Unclear	Unclear	Yes	Unclear	Yes	Methodological concerns
Renko 2017 [73], Finland	Yes	Yes	Yes	Yes	Yes	No unexpected imbalances	No	Yes	Yes	High
Rozzelle 2008 [74], USA	Yes	Yes	Yes	Yes	Unclear	Unclear	No	Yes	None observed	Methodological concerns
Ruiz-Tovar 2020 [75], Spain	Yes	Unclear	Yes	No	Yes	No unexpected imbalances	Yes	No	Yes	Methodological concerns
Ruiz-Tovar 2015 [57], Spain	Yes	Yes	Yes	No	Yes	No unexpected imbalances	No	No	None observed	Methodological concerns
Santos 2019 [76], Brazil	Yes	Yes	Yes	Yes	Yes	No unexpected imbalances	No	No	None observed	Methodological concerns
Seim 2012 [77], Norway	Unclear	Unclear	Yes	No	Unclear	No unexpected imbalances	No	No	Yes	Methodological concerns
Soomro 2017 [58], Pakistan	No	No	Unclear	No	Yes	No unexpected imbalances	No	Yes	Yes	Methodological concerns
Sprowson 2018 [78], UK	Unclear	Yes	Yes	No	Yes	No unexpected imbalances	No	No	Yes	Methodological concerns
Sukeik 2019 [79], UK	Yes	Yes	Yes	Yes	Yes	No unexpected imbalances	No	Unclear	Yes	Unclear
Sundaram 2020a [80], USA	Unclear	Unclear	Yes	No	Yes	No unexpected imbalances	No	Yes	Yes	Methodological concerns
Sundaram 2020b [81], USA	Unclear	Unclear	No	No	Yes	No unexpected imbalances	Yes	Yes	Yes	Methodological concerns
Tabrizi, 2019 [82], Iran	Yes	Unclear	No	No	No	No unexpected imbalances	No	Yes	Yes	Methodological concerns
Thimour-Bergström 2013 [83], Sweden	Unclear	Yes	Yes	Yes	Yes	No unexpected imbalances	No	No	Yes	Methodological concerns
Turtiainen 2012 [84], Finland	Yes	Yes	Yes	Yes	Yes	No unexpected imbalances	No	Yes	None observed	High
Williams 2011 [85], UK	Yes	Yes	Yes	Yes	Yes	No unexpected imbalances	No	No	None observed	Methodological concerns
Zhang 2011 [86], China	Yes	Yes	Yes	No	No	No unexpected imbalances	Yes	Yes	Yes	Methodological concerns

**Table 4:** Overview of study characteristics of included studies

**Type of surgery**	Including (but not limited to): • Multiple types of abdominal surgery • Knee and hip arthroplasty • Surgery for pilonidal disease • Coronary artery bypass graft surgery with saphenous vein harvesting • Breast surgery • Dental surgery • Sinus excision • Implantation of a cerebrospinal fluid shunting device
**Population studied**	• Paediatric population (2 studies) • Adult only population (23 studies) • Mixed population (including both adults and children; 4 studies) Two studies [57, 65] did not provide sufficient information to determine the population studied and were excluded from the child or adult subgroup analyses
**Suture type**	All studies compared a triclosan-coated suture against a non-coated suture material: • 26 studies assessed either Vicryl Plus, Monocryl Plus, or PDS Plus against an uncoated suture material • 2 studies [57, 58] assessed an unnamed triclosan-coated suture against an uncoated suture • 1 study [75] assessed three arms: Stratafix Symmetric Plus, PDS Plus, and uncoated PDS • 2 studies [80, 81] assessed Stratafix Symmetric Plus against an uncoated suture
**Study design**	All studies were randomized controlled trials: • 28 studies randomized individual patients to the intervention or control arm • 1 study [66] randomized groups of patients rather than individuals • 1 study [78] quasi randomised based on the monthly assignment of the participating hospitals to one of the two interventions • 1 study [74] randomised procedures rather than patients: 84 shunt procedures were performed in 61 patients. Patients receiving new shunts following infection of the original and patients undergoing revision were rerandomized and included again in the assessment. However, as these patients were successfully and fully treated for their shunt infections prior to re-implantation, Rozelle 2008 was retained for inclusion in the meta-analyses
**Publication date**	Studies were conducted across a span of 15 years between 2005 [62] and 2020 [80, 81] Clinical pathways and practices are likely to have changed somewhat across this timespan. However, as the meta-analysis utilised within-study comparisons, this was not considered to be a significant problem

Of the 31 studies included in the review, three studies [61, 73, 84] were judged to be at a ‘low’ risk of bias, and two further studies [70, 79] were considered to have an overall ‘unclear’ risk of bias (Table 3). The remaining 26 studies were judged to have possible ‘methodological concerns’, most of which related to the adequacy of blinding of individuals involved in the trial and whether an appropriate intention-to-treat (ITT) analysis was conducted.

#### Results of outcomes not suitable for meta-analysis

All outcomes, except incidence of SSI, were unsuitable for assessment using meta-analysis. The main reason was a lack of reporting of the outcomes; when reported, the information was often incomplete. A summary of the reported outcomes, including safety outcomes, is presented in Supplementary Sect. 5.1.

The included studies reported minimal adverse events related to triclosan-coated sutures. Since adverse events are likely to emerge within the follow-up time of the RCTs, the evidence seems robust enough to exclude the possibility of significant adverse events related to the triclosan-coated sutures.

### Results of the meta-analysis

Incidence of SSI was reported in all but one [81] of the 31 included studies. The similarity assessment to determine suitability of the trials for inclusion in the meta-analysis indicated that overall there was homogeneity between the studies. All but two studies [67, 72] reported outcomes at a timepoint of one month or longer, meaning that all SSI as described by the CDC definition [3] should have been captured by the studies. These two studies were excluded from the meta-analysis due to the potential impact of their short follow up: Karip 2016 [67] assessed infection rates at two weeks after surgery, and Rasic 2011 [72] only monitored outcomes during the hospitalization period (mean of 13.2 and 21.4 days for the intervention and comparator arms, respectively).

Similarity of the studies was confirmed by quantitative assessment. A Baujat diagnostic plot (Supplementary Fig. 3) showed that no study highly influenced the pooled effect size while also highly contributing to the overall heterogeneity of the meta-analysis, and a Leave-One-Out analysis (Supplementary Fig. 4) showed that no single study highly influenced heterogeneity or the pooled effect size with I^2^ ranging from 33 to 41% and the pooled effect size ranging from 0.67 to 0.70. Removal of Karip 2016 [67] and Rasic 2011 [72] did not unduly influence the primary outcome.

#### Selection of data for analyses

Thimour-Bergström 2013 [83] contributed two datasets to the meta-analysis; further details of these data can be found in the Supplementary Sect. 2.

#### Meta-analysis results

**Fig. 3:**
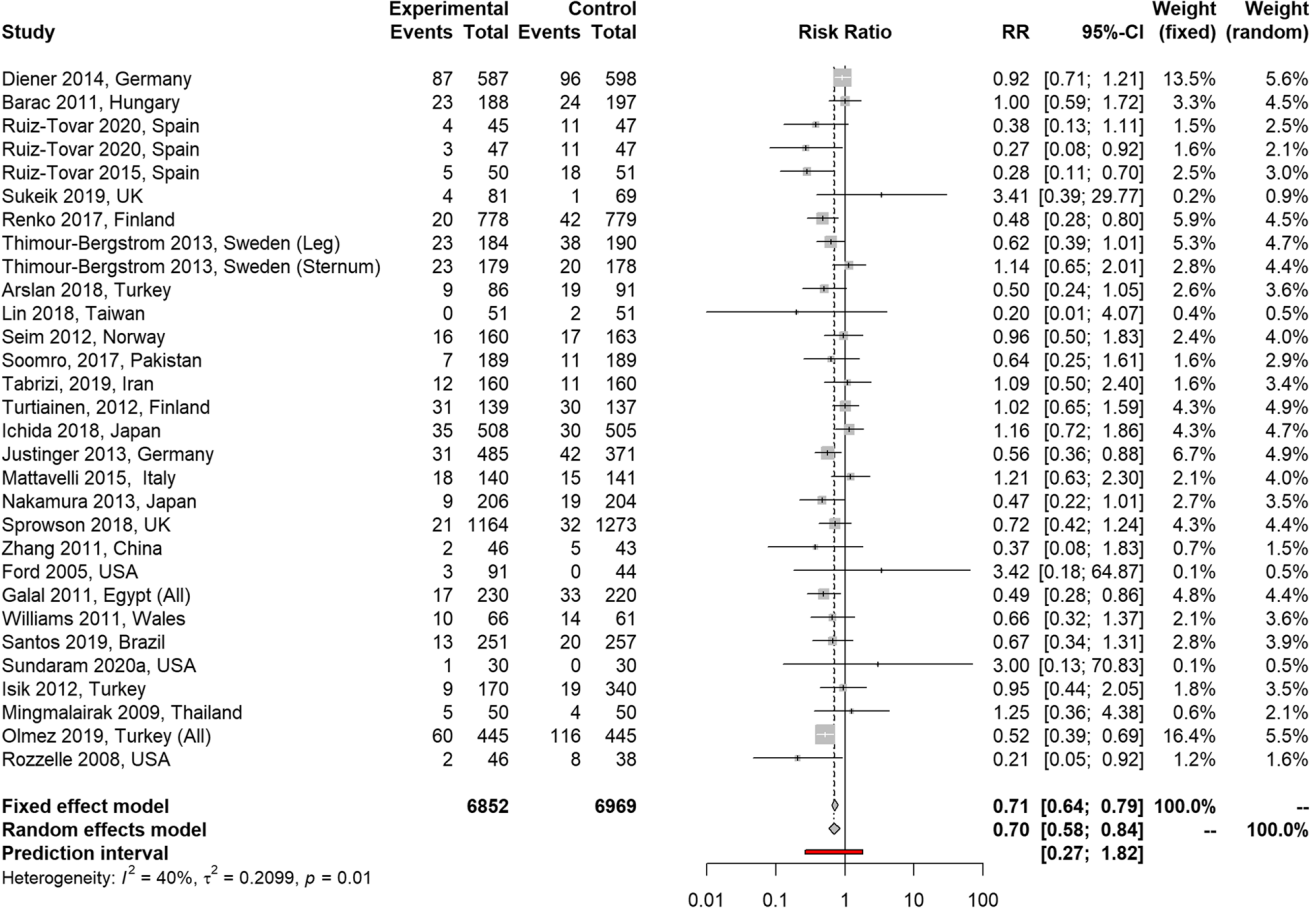
Meta- analysis results—All SSI incidence studies

The fixed effects model for SSI including all groups of patients estimated a RR of 0.71 (95% confidence interval (CI): 0.64 to 0.79) indicating those in the triclosan-coated sutures (including Stratafix Plus) group had a 29% reduction in the risk of developing an SSI compared to those in the control group (*p* < 0.001). The random effects model estimated a RR of 0.70 (95% CI: 0.58 to 0.84; *p* < 0.001). These results are presented in Fig. 3 and are based on 6,852 and 6,969 total patients, and 503 and 708 events in the triclosan-coated sutures (including Stratafix Plus) and control arm, respectively. No outliers or substantial publication bias (see Supplementary Fig. 5) were noted during the analysis.

Triclosan-coated sutures were found to be associated with a significant reduction in the risk of developing an SSI compared with those in the control group in all analyses conducted (25% to 48% reduction using fixed effects models across main analysis and subgroups). Full details of subgroups are presented in Supplementary Fig. 6a to d. Consistent findings were reported when Stratafix Plus was removed from the analyses (28% reduction; Supplementary Fig. 7).

### Results of the economic model

The base case model results are presented in Table 5. The use of triclosan-coated sutures was estimated to result in average cost savings of £13.63 per patient. Triclosan-coated sutures remained cost saving in all subgroup analyses with cost savings estimated ranging between £11 (clean) and £140 (non-clean). Further details are presented in Supplementary Sect. 7.

**Table 5:** Results of the economic model: base case results – differences in costs

	**Triclosan-coated sutures**	**Comparator sutures**^**a**^	**Difference (Triclosan-coated sutures minus Comparator)**^**b**^
***Key model outcomes***			
Device cost (Mean cost per patient—£)	£21.25	£16.75	£4.50
Cost of SSI treatment (Mean cost per patient—£)	£44.39	£62.53	-£18.13
**Total cost per patient**	**£65.64**	**£79.28**	**-£13.63**
**Total cost (per 1,000 patients)**	**£65,645**	**£79,278**	**-£13,633**
***Other model outcomes***			
Number of SSIs per 1,000 patients	7.4	10.4	-3.0
**Cost per SSI averted**			**Dominant**
Number of deaths per 1,000 patients	13.04	13.06	-0.02
**Cost per death averted**			**Dominant**

Note: Dominant = more effective and less costly than the comparator

^a^Sutures that do not contain an antibacterial agent

^b^Negative values indicate a cost saving

**Fig. 4:**
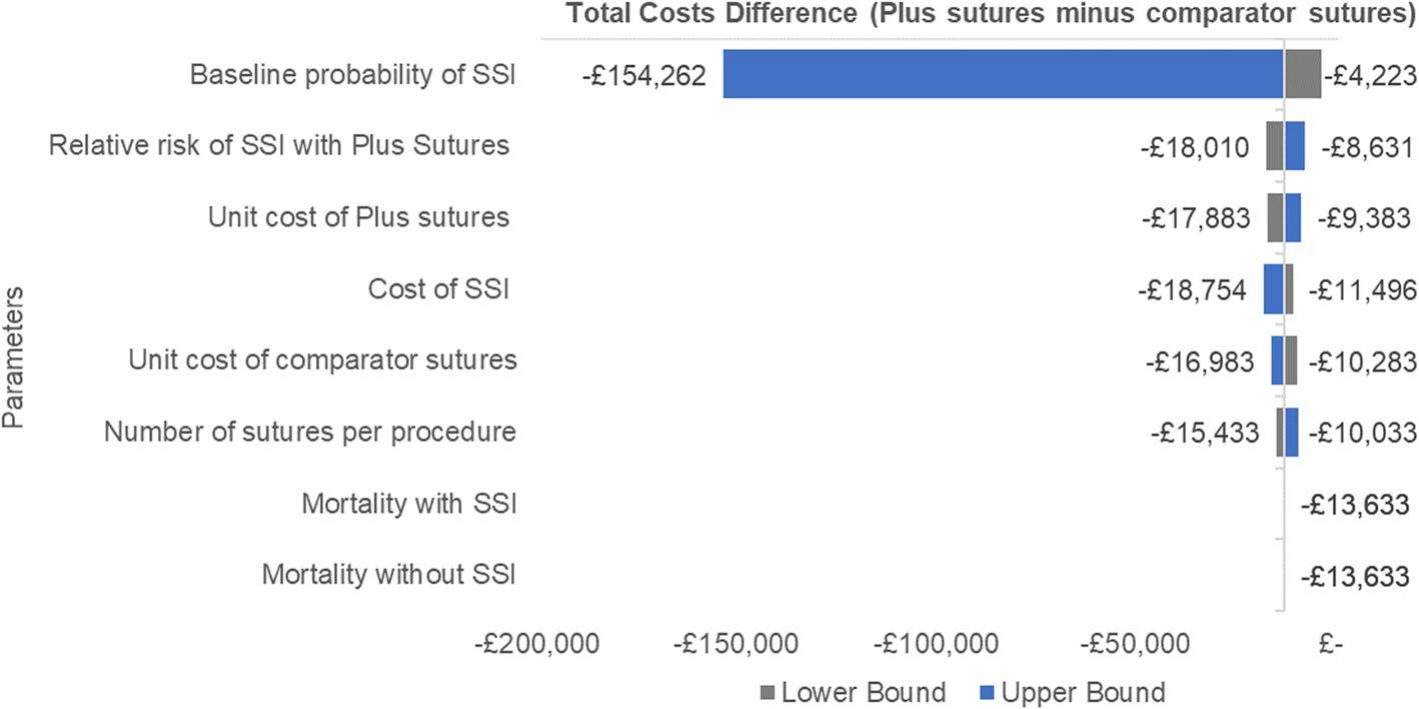
Tornado plot presenting one-way sensitivity analysis

As shown in the tornado plot (Fig. 4), use of triclosan-coated sutures remained the cost saving treatment strategy across all parameters that were changed individually within plausible ranges. The main driver of the analysis is the baseline risk of SSI with comparator sutures, followed by the RR of SSI with triclosan-coated sutures, and the cost of SSI.

The probabilistic sensitivity analysis demonstrated that the results are robust when varying all input parameters simultaneously around pre-specified distributions that aimed to reflect the uncertainty in each parameter. These distributions were either based on uncertainty estimates from the literature or plausible assumptions where data were not available. All parameters were varied in the PSA with the majority of distributions based on confidence intervals reported in the literature or as per the meta-analysis, particularly for those parameters that are key drivers of the results (baseline risk of SSI, RR of SSI with triclosan-coated sutures and cost of SSI). 99.8% of iterations were cost saving when 1,000 iterations of the model were run.

### Results of the sustainability model

The environmental model results are presented in Table 6. The environmental impact of an SSI was estimated to incur 576 kg CO_2_e GHG emissions (equivalent to return flights from London to Rome for two people) [87], 872 m^3^ water use and 65 kg waste generation. The use of triclosan-coated sutures, through reduction of SSI risk, was estimated to result in the environmental savings to NHS England of 1.74 tCO2e (equivalent to return flights from London to Rome for six people) [87], 2,629 m^3^ water use (equivalent to 25 times the annual drinking water use of an average European household) [88] and 0.2 tonnes of waste (equivalent to over four times the monthly waste generation of an average European person) [89], per 1,000 patients.

**Table 6:** Results of the environmental sustainability model

**Environmental impact of SSI**					
**Activity**	**Unit**	**per SSI**	**GHG emissions (kg CO**_**2**_**e)**	**Fresh water use (m**^**3**^**)**	**Waste generation (kg)**
Additional LOS in general ward	Days	6.8	258	413	22
Additional LOS in ICU	Days	3.2	286	440	42
Additional outpatient visits	Number	4.1	5	9	1
Additional outpatient journeys (including return journeys)	Number	8.2	24	4	0
Additional A&E attendance	Number	0.22	3	5	0
Journeys to A&E (return not included)	Number	0.22	1	0	0
**Total environmental impact of an SSI**			**576**	**872**	**65**
**Potential environmental benefits of reductions in SSI with the use of triclosan-coated sutures**					
	**Triclosan-coated sutures**		**Comparator Sutures**^**a**^		**Difference (triclosan-coated sutures minus Comparator)**
Number of SSIs per 1,000 patients	7.4		10.4		-3.0
GHG emissions due to SSI per 1,000 patients (tCO2e)	4.26		6.0		-1.74
Water use due to SSI per 1,000 patients (m^3^)	6,438		9,067		-2,629
Waste generation due to SSI per 1,000 patients (t)	0.48		0.68		-0.2

Note: Negative values indicate an environmental saving

^a^Sutures that do not contain an antibacterial agent

## Discussion

### Clinical outcomes

Thirty-one RCTs, including 13,754 patients and 1,211 episodes of SSI were eligible for this systematic review.

Results of the overall population meta-analysis indicated that patients in the triclosan-coated sutures (including Stratafix Plus) group had a 29% reduction in the risk of developing an SSI compared with those in the control group. All analyses showed a statistically significant reduction (between 25 to 48% depending on subgroup) in incidence of SSI with the use of Plus Sutures. Overall, the meta-analysis incorporated a homogenous group of studies (assessed using both quantitative and qualitative methods) and supports the use of Plus Sutures in reducing the risk of SSI [24]. Furthermore, these findings support the results of previous systematic reviews and meta-analyses which evaluated SSI risk in triclosan-coated sutures against standard sutures [90–93]. While the existing meta-analyses were informed by older searches [90, 91] or differed in focus and scope from this review (Otto-Lambertz 2023 limited to clean and clean-contaminated procedures only), the results consistently show a benefit for triclosan-coated sutures of between 24% [93] and 28% [91].

However, we note that a recently published systematic review [94] (searches in September 2021) concluded that there were no differences between triclosan-coated and uncoated sutures. The 2021 review had a restricted PICO (clean wounds and children excluded) and the meta-analysis only included studies deemed to have a low risk of bias as judged by an unvalidated tool (adapted specifically for SSI from the Cochrane risk of bias-2 tool, with input from the review authors and clinicians).

Efficacy outcomes other than SSI were not reported consistently in the published primary studies, and where they were reported, the strength of the evidence varied. More consistent reporting of these important outcomes is recommended in line with NICE recommendations [95].

The included studies reported minimal device-related adverse events. Since adverse events were likely to emerge within the follow-up time of the RCTs, the evidence seems robust enough to exclude the possibility of significant adverse events related to the triclosan-coated sutures [24].

#### Strengths and limitations of the clinical evidence

The clinical evidence is drawn from a good number of RCTs (31) including a high number of SSIs (1,211) across a wide range of surgery types. In 27 of the included studies, incidence of SSI was the primary outcome, with most studies using a commonly accepted definition of SSI and a clearly defined patient population.

As Plus Sutures were the only triclosan-coated sutures on the market at the time of conducting the review [J&J to provide citation as detailed above], these analyses reflect the available evidence base and may not be applicable to any future antibiotic suture products that may come to market.

The 31 studies represented eight middle income and eleven high income countries. This range of countries provides good generalisability to the middle / high income global context, although low income countries are not represented in this review. The FALCON trial [96] (published after searches for this review were conducted) has recently suggested that in 54 hospitals located in low and middle income countries (Benin, Ghana, India, Mexico, Nigeria, Rwanda and South Africa) the use of triclosan-coated sutures did not reduce the risk of SSI. Some of the pragmatic choices made in the conduct of the trial limit the generalizability of the results. Among them, (i) allowing for different techniques of skin closure, (ii) use of triclosan-coated sutures only for fascia closure and not for skin closure, (iii) compliance with the individual component of the WHO checklist [10] was not mandated and (iv) no standard postoperative management of the wounds was prescribed. SSI are multifactorial and the absence of clear standardisation in pre-, intra-, and post-operative management of the wounds in the trial raises the risk that the effect of single components (like the use of Plus Sutures) is obscured by other non-controlled factors. This risk may be heightened in emergency procedures which comprised over 60% of the cases in FALCON. The results of FALCON may therefore not be applicable outside the geographical context, the surgical technique used, and the population included in the trial.

A recent systematic review and analysis of 29 trials in clean and clean-contaminated procedures, including FALCON (Otto-Lambertz 2023 [93]), found a significant 24% reduction in postoperative wound infection rate with the use of triclosan-coated sutures.

An exploratory analysis (reported in Supplementary Fig. 8) was conducted to assess the impact of the FALCON trial on the meta-analysis described in this paper, and found similar results to those of Otto-Lambertz. No publication bias was identified, and the fixed effects model indicated that patients in the triclosan-coated sutures (including Stratafix Plus) group had a 18% reduction in the risk of developing an SSI compared with those in the control group. This reduces the effect of the main analysis, which showed a 29% reduction of risk, but still suggests benefits associated with the triclosan-coated sutures compared to standard sutures.

Studies included in this review covered a wide range of surgical interventions, both emergency and planned, as laid out in Table 4. Most of the studies included patients with comorbidities including diabetes, chronic obstructive pulmonary disease (COPD), malignant diseases, chronic renal insufficiency, anaemia, and people living with obesity or malnourishment. This large heterogeneity in patient population in conjunction with the positive result and low heterogeneity of the meta-analysis suggests that the intervention can be recommended to a wide population of patients.

Not all studies were blinded in the same way; 15 of the studies were double-blind and the remaining 16 were either single-blind, open label, or not clearly reported. In addition, the studies were conducted in a wide range of countries over a fifteen-year date span (from 2005 to 2020). During this time, the clinical pathways are likely to have changed. Despite these factors, heterogeneity across studies was not substantial, and the use of random effects models accounted for any variability.

As recommended by Cochrane to increase precision of the results [97], the current review incorporated evidence from all available trials without removing the high-risk of bias studies. We acknowledge that only limited number of RCTs (three of the total 31 included in the review) were deemed to have a low risk of bias.

### Economic evidence

The economic model indicates that the use of triclosan-coated sutures results in estimated cost savings of almost £14 per patient if introduced in the NHS. This equates to an estimated cost saving for an average UK NHS hospital of around £13,000 based on 954 surgical procedures per year [98]. Cost savings result from a reduction in SSI (as demonstrated in the clinical evidence) and, therefore, a reduction in the healthcare related costs and resources associated with treating SSI. The increase (~ £4.50 per patient) in costs of using triclosan-coated sutures compared with alternative sutures is outweighed by savings from a reduction in SSI incidence. Results were robust to changes in individual input parameters as demonstrated in the sensitivity analyses. Plus Sutures remained cost saving in all subgroup analyses with cost savings estimated between £11 (clean wound procedures) and £140 (non-clean wound procedures) per patient.

The results of the economic model are consistent with other literature which also demonstrates cost savings with the introduction of Plus Sutures compared with sutures that do not contain an antibacterial agent [38–45]. Only one study [40] was identified which adopted the same UK perspective and this estimated greater cost savings with the introduction of Plus Sutures compared than those reported in this work (£91 vs £14). The assumptions used in the current economic model presented a conservative case for the introduction of Plus Sutures. Therefore, the model outputs and cost savings are likely to be lower than those reported in other literature. Leaper and colleagues [40] also reported cost savings of £57 per clean wound procedures and £248 per non-clean wound procedures. These savings are greater than those estimated in this model. Leaper and colleagues [40] did not report the model inputs used, thus making direct comparisons challenging. However, it appears that the cost of SSI and the baseline risk of SSI with comparator sutures used in the Leaper model may have been higher than in the current study.

The baseline risk used for incidence of SSI with comparator sutures in this model is likely to underestimate the true incidence in the NHS due to lack of robust SSI surveillance and the fact that Plus Sutures are currently used in the UK. The PHE registry is likely to be subject to important selection biases that may produce lower estimates of SSI incidence than are observed in practice [36]. Data submission to the registry is mostly voluntary and is unlikely to identify SSIs arising in the community after discharge. These issues have also been acknowledged in published literature [99, 100]. Using a higher value for the incidence of SSI with comparator sutures increased the estimated cost savings with Plus Sutures. For example, where the higher baseline risk of SSI (1.97%) reported by Jenks et al. is used [5], the cost savings with Plus Sutures are estimated to be £30 per patient. Strengths of the economic analysis include that:

- ▪ The structure, inputs and results are aligned with previously published models and with models used in the UK’s health technology assessment agency guidance (NICE) [36, 37].
- ▪ The RR of SSI with triclosan-coated sutures was identified through a systematic review and meta-analysis and is based on a sizable body of RCTs with statistically significant confidence intervals estimated, and was judged to accurately reflect the range of patients and procedures within the NHS.
- ▪ Extensive sensitivity analyses were conducted, and the model results were robust to plausible changes in input parameters.
- ▪ Conservative parameter estimates/assumptions were used. Therefore, the uncertainty in the model is minimised and robust estimates of the cost savings associated with the use of Plus Sutures within the NHS are presented.

Limitations of the cost analysis include that:

- ▪ The source used for the baseline risk of SSI is widely accepted to underreport the incidence of SSI in the NHS. Therefore, the cost savings in the model may be underestimated.
- ▪ The source used for the cost of SSI is potentially outdated; however, a more suitable source could not be identified. If the average cost of SSI is higher than that reported by Jenks et al. [5], then the cost savings in the model may be underestimated. It was noted that several changes in clinical practice which have occurred since publication of the Jenks study. These changes include the number of infections caused by multi-drug resistant bacteria, which could result in longer duration of IV antibiotics and longer admissions in hospital, and the increase in complexity of care due to multi-morbidity of the population, which suggest the costs of SSI may have increased.
- ▪ QoL was not considered in the model (in line with the NICE MTEP methods guide [101]. However, it is very likely that a reduction in the incidence of SSI will impact on patient’s QoL [36, 102, 103].

Overall, the results of the cost analysis are likely to provide a good reflection of the impact of introducing Plus Sutures into routine care in the NHS. However, it is expected that these results underestimate the true savings that could be released within clinical practice from adoption of Plus Sutures across the NHS.

Although this analysis was conducted from a UK NHS perspective it is expected that the findings would generalise and Plus Sutures would lead to cost savings in other settings and countries. Indeed these results are in line with existing published cost analyses [38, 39, 41–45] of Plus Sutures conducted in other countries, which used varying methods to estimate costs across a range of patient populations and settings but all found the technology to be cost saving. The studies were conducted in Italy [38], Austria [39], the US [41, 44, 45], India [42] and Japan [43].

### Environmental impact

The sustainability analysis demonstrated that the environmental impact of SSI is substantial. Furthermore, this analysis highlighted that the use of Plus Sutures, through a lowered risk of SSI, could result in potential environmental benefits to the healthcare systems. Although this analysis was consistent with the methods described in the SHC guidance and provided a reasonable estimate of the environmental impact, the identification of more specific data relating to some of the care pathway activities could enhance the overall accuracy of the environmental impact assessment.

Impacts associated with energy consumption for care pathway activities were sourced from the Sustainable Care Pathways Guidance [54], which is based on the UK government emission factor data. Therefore, climate impacts from energy consumption for other countries could vary, due to differences in national electricity grid mixes. However, it is likely that the general findings of this study regarding the contribution that specific activities of the pathway make to the overall environmental impact are likely applicable to wider settings.

Through reducing risk of SSI and subsequent antibiotic prescribing, Plus Sutures has potential to deliver a direct positive contribution to environmental sustainability across healthcare systems. A recent European Public Health Alliance report [104] states that antimicrobial resistance (AMR) jeopardises the achievement of sustainable development goals and includes a focus on infection prevention and control to reduce the need for antibiotics and consequently decreasing risk of antimicrobial resistance.

Triclosan has been used since the 1970s in consumer and professional products [105]. Its safety and effectiveness in consumer antiseptic washes and healthcare handwashes, scrubs, and skin preps was questioned because of insufficient data [106, 107]. However, Plus Sutures have been shown in vivo and in vitro to be non-toxic, non-irritating, non-carcinogenic, and non-teratogenic [105]. The small amount of triclosan used in Plus Sutures does not accumulate in the body and it is metabolized and excreted in a neutralised form [105]. Despite the theoretical possibility of resistance to Triclosan, there is no known clinical connection to antibiotic cross-resistance [108, 109]. In addition the Scientific Committee on Consumer Safety (SCCS) clearly states that “there is no evidence that using triclosan leads to an increase in antibiotic resistance…to preserve the role of triclosan in infection control and hygiene, SCCS can only recommend its prudent use, for instance limited to applications where a health benefit can be demonstrated…” [110]. There is a likely reduction of antibiotic prescribing as a consequence of the prevention of SSI with the routine use of Plus Suture [111]. In the opinion of the authors, this could have a beneficial impact and reduce the development of antimicrobial resistance.

This was the first time that an environmental sustainability analysis was included in a NICE MTEP submission and formed a key part of the final report. Given the increased interest and importance of assessing the impact on the environment, the methodology illustrated in this work can serve as an example to guide future evaluation of interventions.

## Conclusions

Overall, the available evidence demonstrates that triclosan-coated sutures are associated with a reduced incidence of SSI across all surgery types. A qualitative analysis of safety outcomes found that no significant harms were reported, while the economic model indicated cost savings for Plus Sutures when compared with non-coated sutures that do not contain an antimicrobial agent. Furthermore, the environmental impact of SSI is substantial, and the introduction of Plus Sutures could result in potential environmental benefits.

More robust trials with standardized surgical protocols (in particular we would advocate the use of triclosan sutures for both fascia and skin closure), especially in low-income countries, are still needed to further validate the SSI incidence in these challenging environments. Safety outcomes should be more consistently reported across trials, as existing evidence around safety is weak.

Considering the available evidence, Plus Sutures appear to be a useful device to minimise the risk of SSI. This is aligned with recommendations included in global guidelines, including NICE SSI Guidelines [112], WHO Global guidelines [10] and the 2017 CDC guidelines [9] all of which recommend the use of triclosan-coated sutures for the purpose of reducing the risk of SSI, independent of the type of surgery.

### Supplementary Information

**Additional file 1: Supplementary Figure 1.** Search strategies. **Supplementary Table 1.** Model inputs. **Supplementary Figure 2.** Structure of the environmental model. **Supplementary Table 2.** Environmental sustainability model inputs. **Supplementary Table 3.** Documents excluded at full text review (*n* = 108). **Supplementary Table 4.** Studies included in the review. **Supplementary Table 5.** Summary of review included study characteristics. **Supplementary Table 6.** Summary of review included study population details. **Supplementary Table 7.** Risk of bias assessment of studies included in the review. **Supplementary Table 8.** Antibiotic use for SSI (presenting only those studies reporting eligible data by arm). **Supplementary Table 9.** Hospital stay (presenting only those studies reporting eligible data by arm). **Supplementary Table 10.** Severity of SSIs (presenting only those studies reporting eligible data by arm). **Supplementary Figure 3.** Baujat diagnostic plot. **Supplementary Figure 4.** Leave-One-Out analysis. **Supplementary Figure 5.** Publication bias: funnel plot. **Supplementary Figure 6a.** Meta- analysis results – Adult only SSI incidence studies (with Stratafix). **Supplementary Figure 6b.** Meta- analysis results – Children only SSI incidence studies (with Stratafix). **Supplementary Figure 6c.** Meta- analysis results – Clean wound only SSI incidence studies (with Stratafix). **Supplementary Figure 6d.** Meta- analysis results – Non-clean wound only SSI incidence studies (with Stratafix). **Supplementary Figure 7.** Meta- analysis results – Without Stratafix sensitivity analysis. **Supplementary Figure 8.** Meta- analysis results – including Falcon trial. **Supplementary Figure 9.** Labbe plot – including Falcon trial. **Supplementary Figure 10.** Baujat plot – including Falcon trial. **Supplementary Figure 11.** Left-one-out plot – including Falcon trial. **Supplementary Figure 12.** Funnel plot – including Falcon trial. **Supplementary Table 11.** Threshold/breakeven analyses results. **Supplementary Table 12.** Subgroup analyses for adults only. **Supplementary Table 13.** Subgroup analyses for children only. **Supplementary Table 14.** Subgroup analyses for clean wounds only. **Supplementary Table 15.** Subgroup analyses for non-clean wounds only. **PRISMA 2020 Checklist.** **Supplementary Appendix A.** Full Search Strategies for the Systematic Review.

## Data Availability

The protocol for this review was registered with the Open Science Foundation and can be accessed at https://osf.io/yvjna. The datasets used and/or analysed during the current study may be available from the corresponding author on reasonable request.

## Abbreviations

AMR: Antimicrobial resistance
ASEPSIS: Additional treatment, serous discharge, erythema, purulent exudate, separation of tissues, isolation of bacteria, stay duration as an inpatient
CADTH: Canadian Agency for Drugs and Technologies in Health
CDC: Centers for Disease Control
CI: Confidence interval
COPD: Chronic obstructive pulmonary disease
EUnetHTA: European Network for Health Technology Assessment
GHG: Greenhouse gas
HRQoL: Health-related quality of life
HTA: Health technology assessment
ITT: Intention-to-treat
MAUDE: Manufacturer and User Facility Device Experience
MHRA: Medicines and Healthcare products Regulatory Agency
MTEP: Medical Technologies Evaluation Programme
NHS: National Health Service
NICE: National Institute for Health and Care Excellence
PHE: Public Health England
PRISMA: Preferred Reporting Items for Systematic Reviews and Meta-Analyses
PSA: Probabilistic sensitivity analysis
RCT: Randomized controlled trial
RR: Risk ratio
SCCS: Scientific Committee on Consumer Safety
SSI: Surgical site infection
WHO: World Health Organization
