# Supplementary material for: Plus Sutures for preventing surgical site infection: a systematic review of clinical outcomes with economic and environmental models
Source: BMC Surg. 2023 Oct 3;23:300. doi: 10.1186/s12893-023-02187-0 (PMC10548560; doi:10.1186/s12893-023-02187-0)
Supplement: Supplementary file 1 — Additional file 1: Supplementary Figure 1. Search strategies. Supplementary Table 1. Model inputs. Supplementary Figure 2. Structure of the environmental model. Supplementary Table 2. Environmental sustainability model inputs. Supplementary Table 3. Documents excluded at full text review (n = 108). Supplementary Table 4. Studies included in the review. Supplementary Table 5. Summary of review included study characteristics. Supplementary Table 6. Summary of review included study population details. Supplementary Table 7. Risk of bias assessment of studies included in the review. Supplementary Table 8. Antibiotic use for SSI (presenting only those studies reporting eligible data by arm). Supplementary Table 9. Hospital stay (presenting only those studies reporting eligible data by arm). Supplementary Table 10. Severity of SSIs (presenting only those studies reporting eligible data by arm). Supplementary Figure 3. Baujat diagnostic plot. Supplementary Figure 4. Leave-One-Out analysis. Supplementary Figure 5. Publication bias: funnel plot. Supplementary Figure 6a. Meta- analysis results – Adult only SSI incidence studies (with Stratafix). Supplementary Figure 6b. Meta- analysis results – Children only SSI incidence studies (with Stratafix). Supplementary Figure 6c. Meta- analysis results – Clean wound only SSI incidence studies (with Stratafix). Supplementary Figure 6d. Meta- analysis results – Non-clean wound only SSI incidence studies (with Stratafix). Supplementary Figure 7. Meta- analysis results – Without Stratafix sensitivity analysis. Supplementary Figure 8. Meta- analysis results – including Falcon trial. Supplementary Figure 9. Labbe plot – including Falcon trial. Supplementary Figure 10. Baujat plot – including Falcon trial. Supplementary Figure 11. Left-one-out plot – including Falcon trial. Supplementary Figure 12. Funnel plot – including Falcon trial. Supplementary Table 11. Threshold/breakeven analyses results. Supplementary Table 12. Subgroup analyses fo [file 12893_2023_2187_MOESM1_ESM.docx]

**Plus Sutures for preventing surgical site infection: a systematic review to inform economic and environmental models**

Supplementary file

# Systematic review methods

The full search strategy for MEDLINE is presented in Supplementary Figure 1. This strategy was translated appropriately for the other databases.

### Supplementary_Figure_1: Search strategies

Source: MEDLINE ALL

Interface / URL: OvidSP

Database coverage dates: 1946 to January 29, 2021

Search date: 01/02/21

Retrieved records: 422

Search strategy:

1 Sutures/ (17365)

2 Suture Techniques/ (43238)

3 sutur$.ti,ab,kf. (81242)

4 stitch$.ti,ab,kf. (5666)

5 ((surg$ or dissect$ or excis$ or fascia$ or incis$ or intraoperat$ or operat$ or postdissect$ or postexcis$ or postincis$ or postoperat$ or postsurg$ or perioperat$ or skin or skins or tissue$ or wound$) and (ligat$ or loop$ or thread$)).ti,ab,kf. (81457)

6 or/1-5 (185804)

7 Surgical Fixation Devices/ (189)

8 Wound Closure Techniques/ (1628)

9 ((surg$ or dissect$ or excis$ or fascia$ or incis$ or intraoperat$ or operat$ or postdissect$ or postexcis$ or postincis$ or postoperat$ or postsurg$ or perioperat$ or skin or skins or tissue$ or wound$) adj6 (approximat$ or clos$ or fasten$ or fixat$ or secur$)).ti,ab,kf. (103269)

10 (device$ adj6 (approximat$ or clos$ or fasten$ or fixat$ or secur$)).ti,ab,kf. (14057)

11 ((fascia$ or skin or skins or tissue$ or wound$) adj6 device$).ti,ab,kf. (7848)

12 or/7-11 (122588)

13 6 or 12 (293804)

14 Triclosan/ (2951)

15 triclosan$.ti,ab,kf,rn,nm. (4315)

16 (cgp433$ or cgp-433$ or ch3565$ or ch-3565$ or cloxifenol$ or dndi1246774$ or dndi-1246774$ or dp300$ or dp-300$ or fat-80$ or fat80$ or gp41-353$ or gp41353$ or irgacare$ or irgacide$ or irgagard$ or irgasan$ or lexol-300$ or lexol300$ or ster-zac$ or sterzac$ or tcs or tricosan$).ti,ab,kf,rn,nm. (6302)

17 (222-182-2 or 3380-34-5 or 4640-01-1 or 4nm5039y5x or 5174ur1dp5).ti,ab,kf,rn,nm. (2951)

18 or/14-17 (9767)

19 ((antibacterial$ or anti-bacterial$ or antibiotic$ or anti-biotic$ or antiinfective$ or anti-infective$ or antimicrobial$ or anti-microbial$ or antimicrobical$ or anti-microbical$ or antiseptic$ or anti-septic$ or biocid$) adj20 (coat$ or impregnat$)).ti,ab,kf. (6564)

20 13 and (18 or 19) (456)

21 plus$ suture$.ti,ab,kf. (38)

22 ((antibacterial$ or anti-bacterial$ or antibiotic$ or anti-biotic$ or antiinfective$ or anti-infective$ or antimicrobial$ or anti-microbial$ or antimicrobical$ or anti-microbical$ or antiseptic$ or anti-septic$ or biocid$) adj sutur$).ti,ab,kf. (102)

23 ((pds$ or pds-ii) adj plus$).ti,ab,kf. (19)

24 ((pds$ adj4 plus$) and sutur$).ti,ab,kf. (27)

25 (monocryl$ adj4 plus$).ti,ab,kf. (9)

26 (vicryl$ adj4 plus$).ti,ab,kf. (60)

27 (pds$ or monocryl$ or vicryl$).ti,ab,kf. and (18 or 19) (70)

28 stratafix$.ti,ab,kf. (39)

29 tissue control device$.ti,ab,kf. (8)

30 ((polydioxanon$ or poliglecapron$ or polyglactin$) adj3 plus$).ti,ab,kf. (28)

31 (polydioxanon$ or poliglecapron$ or polyglactin$).ti,ab,kf. and (18 or 19) (63)

32 or/21-31 (251)

33 20 or 32 (589)

34 exp animals/ not humans/ (4782208)

35 (news or editorial).pt. (761558)

36 33 not (34 or 35) (489)

37 limit 36 to english language (449)

38 limit 37 to yr="2000 -Current" (422)

## Additional outcomes added to NICE scope

Following consultation with clinicians and patient groups, three further outcomes were added to the NICE scope, by which time data extraction for the clinical review had concluded. Data for these outcomes were extracted separately and were summarised with a narrative synthesis. These were:

- Type of SSI (deep/superficial).
- Incidence of wound dehiscence (wound opening).
- Patient reported pain.

# Meta-analysis methods

In order to include a study in the analyses, a mean or median and suitable variance data for the outcome in question were required for both the intervention and comparator arms of the study. The total number of patients analysed for that outcome per arm was also required.

The Mantel-Haenszel method was used to pool effect sizes ^[1, 2]^ and the Sidik-Jonkman estimator was used to calculate τ2 in the random effects models ^[3]^. The Hartung-Knapp adjustment ^[4]^ was used in the random effects models. Finally, a continuity correction of 0.5 was used in studies with zero event counts.

Three heterogeneity measures were used to assess the degree of heterogeneity within the pooled studies; Cochrane’s Q, Higgins and Thompson’s I^2^ and τ2. Furthermore, prediction intervals are displayed for all meta-analyses to provide a range of expected effects for future studies to fall within based on current evidence ^[5]^.

Studies were defined as an outlier if the study’s confidence interval did not overlap the confidence interval of the pooled effect (i.e. there is high certainty that the study cannot be part of the “population” of effect sizes used within the meta-analysis). Funnel plot analysis and Egger’s test of the intercept were used to assess publication bias ^[6]^.

In addition to the similarity assessment, influence analysis was also conducted to detect and remove any extreme influence on the overall effect size, with a Baujat diagnostic plot ^[7]^ and a Leave-One-Out analysis conducted.

## Selection of data for the meta-analyses: Thimour-Bergström 2013

Thimour-Bergström 2013 contributed two datasets to the meta-analysis. Patients in this study were undergoing coronary artery bypass or coronary artery bypass plus valve surgery, using a saphenous vein graft and sternotomy. The primary paper ^[8]^ reported details of leg wounds, and a secondary paper ^[9]^ reported details of sternum wounds. These data are indicated in the analysis plots by the tags “LEG” and “STERNUM”. Clinical input deemed both wound sites as independent of each other. Therefore, both datasets were included in the analysis.

# Modelling methods

### Supplementary_Table_1: Model inputs

| **Parameter/outcomes** | **Input and source** | **Range or distribution** | **Explanatory notes** |
| --- | --- | --- | --- |
| **Baseline risk of infection with comparator sutures** | | | |
| Base case (all surgeries) | 1.04% ^[10]^ | Lower and upper bound 0.5% to 9.1% (based on hip/knee replacement at the lower end to bile duct, liver or pancreatic surgery at the upper end)  Distribution Beta (Alpha: 7040, Beta 670303) | This was calculated by taking the total number of SSI reported in all surgical categories for inpatient and readmission and dividing by the total number of operations.  This value is specific to adults. Based on clinical advice this value is expected to be conservative when applied to children. That is, the true baseline risk of infection in children is expected to be higher than that used in the model. |
| Adults (subgroup) |  |  |  |
| Children (subgroup) |  |  |  |
| Clean (subgroup) | 0.8% ^[10, 11]^ | Lower and upper bound 0.5% to 3.0% (based on hip/knee replacement at the lower end to coronary artery bypass graft at the upper end)  Distribution Beta (Alpha: 5186, Beta 645042) | SSI incidence for inpatient and readmission displayed in table 2 (of the publication) for clean surgeries as assessed by Troughton et al. – weighted average calculated based on the number of SSIs for each surgical category resulting in incidence of 0.8% for surgeries likely to result in clean wounds. |
| Non-clean (subgroup) | 6.8% ^[10, 11]^ | Lower and upper bound 1.8% to 9.1% (based on abdominal hysterectomy at the lower end to bile duct, liver or pancreatic surgery at the upper end)  Distribution Beta (Alpha: 1854, Beta 25261) | SSI incidence for inpatient and readmission displayed in table 2 (of the publication) for non-clean surgeries as assessed by Troughton et al. – weighted average calculated based on the number of SSIs for each surgical category resulting in incidence of 6.8% for surgeries likely to result in non-clean wounds. |
| **Relative risk of infection with Plus Sutures** | | | |
| Base case (all surgeries and all Plus Suture types including Stratafix) | 0.71  Meta-analysis as reported in the main manuscript | Lower and upper confidence interval 0.64 to 0.79  Distribution Lognormal (ln mean: -0.342, ln SE: 0.0537) | Fixed effects model. We used the meta-analysis result with stratafix to ensure that all Plus Sutures were captured. |
| Adults (subgroup) | 0.73  Meta-analysis as reported in Section 6 of this Supplementary Material | Lower and upper confidence interval 0.65 to 0.82  Distribution Lognormal (ln mean: -0.315, SE: 0.0893) | As above, but meta-analysis results based on adult subgroup analyses. |
| Children (subgroup) | 0.52  Meta-analysis as reported in Section 6 of this Supplementary Material | Lower and upper confidence interval 0.32 to 0.87  Distribution Lognormal (ln mean: -0.654, SE: 0.2551) | As above, but meta-analysis results based on children subgroup analyses. |
| Clean (subgroup) | 0.75  Meta-analysis as reported in Section 6 of this Supplementary Material | Lower and upper confidence interval 0.62 to 0.9  Distribution Lognormal (ln mean: -0.288, SE: 0.0951) | As above, but meta-analysis results based on clean subgroup analyses. |
| Non-clean (subgroup) | 0.66  Meta-analysis as reported in Section 6 of this Supplementary Material | Lower and upper confidence interval 0.54 to 0.8  Distribution Lognormal (ln mean: -0.416, SE: 0.1003) | As above, but meta-analysis results based on non-clean subgroup analyses. |
| **Mortality associated with SSI** | | | |
| Base case (all surgeries) | 1.87% ^[12, 13]^ | Lower and upper confidence interval 1.6% to 2.2%  Distribution Beta (Alpha: 157, Beta 8225) | Mortality with SSI for ‘All surgery’ presented in table HE08 in the NICE economic report: 1.87%. This was also used for adult and children subgroups. |
| Adults (subgroup) |  |  |  |
| Children (subgroup) |  |  |  |
| Clean (subgroup) | 2.55 % ^[11-13]^ | Lower and upper bound 2.1% to 3.0%  Distribution Beta (Alpha: 125, Beta 4781) | Mortality with SSI for each surgical category from the NICE economic report was weighted by the number of SSI by surgery from the Public Health England 2017 data. The 2017 PHE report was used because the mortality reported in the NICE SSI guideline is based on this dataset. This approach was taken for clean and non-clean surgical groups respectively as per Troughton et al. ^[11]^. This resulted in values of 2.55% and 2.54% for clean and non-clean subgroups. It is noted that the value for all surgeries reported in the NICE economic report could not be replicated so values for clean and non-clean are both higher than that used for the all surgery, adult and children subgroups. |
| Non-clean (subgroup) | 2.54% ^[11-13]^ | Lower and upper bound 2.0% to 3.1%  Distribution Beta (Alpha: 88, Beta 3388) |  |
| **Mortality for those without an SSI** | | | |
| Base case (all surgeries) | 1.30%^[12, 13]^ | Lower and upper confidence interval 1.27% to 1.33%  Distribution Beta (Alpha: 8507, Beta 645854) | Mortality without SSI for ‘All surgery’ presented in table HE08 in the NICE economic report: 1.30%. This was also used for adult and children subgroups |
| Adults (subgroup) | As above | As above | As above |
| Children (subgroup) | As above | As above | As above |
| Clean (subgroup) | 1.30% ^[12, 13]^ | Lower and upper confidence interval 1.27% to 1.33%  Distribution Beta (Alpha: 7758, Beta 586101) | Mortality without an SSI for each surgical category was weighted by the number of SSI by surgery from the Public Health England 2017 data. The 2017 report was used because the mortality reported in the NICE report is based on this dataset. This approach was taken for clean and non-clean surgical groups respectively as per Troughton et al. ^[11]^. This resulted in values of 1.30% and 2.45% for clean and non-clean subgroups. It is noted that the value for all surgeries reported in the NICE economic report could not be replicated so values for clean and non-clean are both higher than that used for the all surgery, adult and children subgroups. |
| Non-clean (subgroup) | 2.45% ^[12, 13]^ | Lower and upper confidence interval 2.33% to 2.58%  Distribution Beta (Alpha: 1414, Beta 58300) |  |
| **Cost of resource use associated with SSI** | | | |
| Base case (all surgeries) | £6,016 ^[14]^ | Lower and upper confidence interval £5,307 to £7715  Jenks 2014 ^[14]^  Wider variation explored in two-way SA based on NICE health economic report (with cost inflated to current price year*) £3,374 used for lower value  Distribution Gamma  Standard error £614, calculated from confidence intervals in Jenks 2014 ^[14]^ | Inflated from 2011/12 to the current price year (2019/20) using Personal Social Services and Research Unit Healthcare inflation indices. |
| Adults (subgroup) | £6,016 ^[14]^ |  |  |
| Children (subgroup) | £6,016 ^[11, 14]^ |  |  |
| Clean (subgroup) | £7,543 ^[11, 14]^ |  | For clean and non-clean subgroups cost of an SSI from Jenks for each surgical category was weighted by the number of SSI by surgery from the Public Health England 2020 data. This approach was taken for clean and non-clean surgical groups respectively as per Troughton et al. ^[11]^.  Values inflated as above. |
| Non-clean (subgroup) | £6,227 ^[11, 14]^ |  |  |
| **Cost of sutures per patient** | | | |
| Plus sutures | 5 x £4.25 = £21.25 ^[15]^ | Lower and upper bound £3.40 to £5.10  Assumption based on 20% variation from the mean  Distribution Gamma  Standard error 0.85 Assumption based on 20% variation from the mean | 5 sutures used per procedure |
| Comparator sutures | 5 x £3.35 = £16.75 ^[15]^ | Lower and upper bound £2.68 to £4.02  Assumption based on 20% variation from the mean  Distribution Gamma  Standard error 0.67 Assumption based on 20% variation from the mean | 5 sutures used per procedure |

# Environmental sustainability model methods

The sustainability model structure is shown in Supplementary Figure 2. The potential environmental benefits of reductions in SSI with the use of Plus Sutures is calculated by applyingthe RR of infection derived from the meta-analysis to the base case SSI risk from PHE.

### Supplementary_Figure_2: Structure of the environmental model


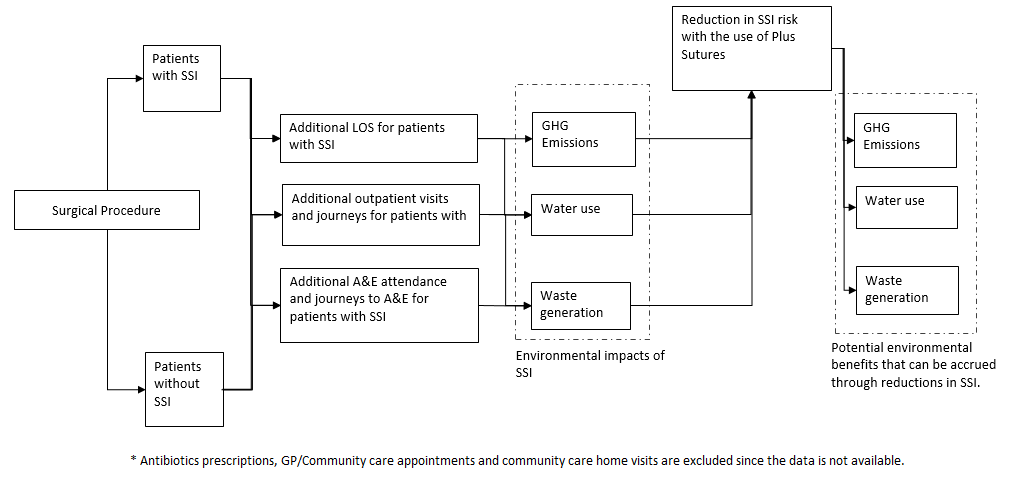


### Supplementary_Table_2: Environmental sustainability model inputs

| **Inputs per activity** | | | | |
| --- | --- | --- | --- | --- |
| ***Activity*** | ***Unit*** | ***GHG Emissions (kg CO₂e)*** | ***Fresh water use (m³)*** | ***Waste generation (kg)*** |
| Physician consultation ^[17]^ | per visit | 1.14 | 2.28 | 0.19 |
| Patient travel to elective care (self) ^[17]^ | per single trip | 2.9 | 0.53 | 0 |
| Emergency department visit ^[17]^ | per visit | 13.8 | 20.9 | 0.29 |
| Inpatient bed day (Low-intensity) ^[17]^ | per day | 37.9 | 60.8 | 3.3 |
| Inpatient bed day (High-intensity) ^[17]^ | per day | 89.5 | 137.5 | 13 |
| **Other inputs** | | | | |
| Additional LOS due to SSI (days) ^[14]^ | | | | 10 |
| The proportion of ICU LOS among total additional LOS due to SSI ^[18]^ | | | | 32% |
| Number of post-operative outpatient visits per patient (Patients with SSI) ^[19]^ | | | | 7.5 |
| Number of post-operative outpatient visits per patient (Patients without SSI) ^[19]^ | | | | 3.4 |
| Patient visits to the emergency department (%)(Patients with SSI) ^[19]^ | | | | 31% |
| Patient visits to the emergency department (%)(Patients without SSI) ^[19]^ | | | | 9% |

# Systematic review results

### Supplementary_Table_3: Documents excluded at full text review (n = 108)

| **Reference** | **Exclusion reason** |
| --- | --- |
| Ahmed I, Boulton AJ, Rizvi S, Carlos W, Dickenson E, Smith NA, et al. The use of triclosan-coated sutures to prevent surgical site infections: a systematic review and meta-analysis of the literature. BMJ Open. 2019;9(9):e029727. | SR or MA for reference checking |
| Allen G. Evidence appraisal of de Jonge SW, Atema JJ, Solomkin JS, Boermeester MA. Meta-analysis and trial sequential analysis of triclosan-coated sutures for the prevention of surgical-site infection.: Br J Surg. 2017;104(2):e118-e133. Aorn J. 2017;106(1):77-82. | Ineligible document type |
| Allen G. Evidence appraisal of Sandini M, Mattavelli I, Nespoli L, Uggeri F, Gianotti L. Systematic review and meta-analysis of sutures coated with triclosan for the prevention of surgical site infection after elective colorectal surgery according to the PRISMA statement.: Medicine. 2016;95(35):e4057. doi:10.1097/MD.0000000000004057. Aorn J. 2017;105(5):518-22. | Ineligible document type |
| Allen G. Evidence for practice. Antimicrobial suture wound closure. Aorn J. 2008;88(6):1014-15. | Ineligible document type |
| Arslan N, Terzi C, Atasoy G, Altintas T, Sirin A, Haciyanli M, et al. Effect of triclosan coated sutures on surgical site infection rate in pilonidal sinus disease: single-blinded randomized trial. Dis Colon Rectum. 2014; (5): e255. Available from: https://www.cochranelibrary.com/central/doi/10.1002/central/CN-01060926/full | Conference abstract |
| Assadian O, Below H, Kramer A. The effect of triclosan-coated sutures in wound healing and triclosan degradation in the environment. Journal of Plastic, Reconstructive & Aesthetic Surgery: JPRAS. 2009;62(2):264-5; author reply 64-5. | Ineligible study design |
| Australian College of Operating Room Nurses. Triclosan-coated sutures and abdominal surgical site infection rate. J Perioper Nurs Aust. 2014;27(3):33. | Ineligible document type |
| AZ St.-Dimpna Geel. Comparison of Laparoscopic Traditional and Knotless Sutures. In: ClinicalTrials.gov [internet]. Bethesda. US National Library of Medicine. 2016. Available from https://clinicaltrials.gov/show/NCT02720718. Identifier: NCT02720718 | Ineligible intervention |
| Barzilai Medical Center. Closing Uterine Incision During C-section Using Barbed Suture (Stratafix) or Vicryl Suture. In: ClinicalTrials.gov [internet]. Bethesda. US National Library of Medicine. 2017. Available from https://clinicaltrials.gov/show/NCT03159871. Identifier: NCT03159871 | Ineligible intervention |
| Brett K, Argaez C. Triclosan in Single Use Medical Devices for Preventing Infections: A Review of Clinical Effectiveness, Safety and Guidelines. Ottawa: CADTH; 2019. Available from: https://cadth.ca/triclosan-single-use-medical-devices-preventing-infections-review-clinical-effectiveness-safety-and. | SR or MA for reference checking |
| Cairo University. Barbed Versus Conventional Sutures for Vaginal Cuff Closure During Total Laparoscopic Hysterectomy. In: ClinicalTrials.gov [internet]. Bethesda. US National Library of Medicine. 2017. Available from https://ClinicalTrials.gov/show/NCT02998658. Identifier: NCT02998658 | Ineligible intervention |
| Cairo University. Comparison of Barbed and Conventional Sutures in Adhesion Formation Following Cesarean Section. In: ClinicalTrials.gov [internet]. Bethesda. US National Library of Medicine. 2017. Available from https://ClinicalTrials.gov/show/NCT03183362. Identifier: NCT03183362 | Ineligible intervention |
| Cairo University. Ultrasound Evaluation of Cesarean Scar After Uterotomy Closure With Barbed and Conventional Sutures. In: ClinicalTrials.gov [internet]. Bethesda. US National Library of Medicine. 2017. Available from https://ClinicalTrials.gov/show/NCT03182010. Identifier: NCT03182010 | Ineligible intervention |
| Chan VWK, Chan P-K, Chiu K-Y, Yan C-H, Ng F-Y. Does Barbed Suture Lower Cost and Improve Outcome in Total Knee Arthroplasty? A Randomized Controlled Trial. J Arthroplasty. 2017;32(5):1474-77. | Ineligible intervention |
| Chosun University Hospital. Prospective Randomized Control Study of Stratafix Vs. Standard-of-care for Deep Tissue Closure in Orthopaedic Surgery. In: Clinical Research Information Service (CRIS) [internet]. Cheongju. Korea Centers for Disease Control and Prevention (KCDC). 2019. Available from https://cris.nih.go.kr/cris/mobile/mobile_view_en.jsp?btype=2&seq=14253. Identifier: KCT0004190 | Ineligible intervention |
| Cozar Lozano C, Garcia-Botello S, Marti-Arevalo J, Bauza Collado M, Pla Marti V, Moro Valdezate D, et al. Use of triclosan-coated barbed monofilament suture (TCBMS) to reduce surgical site infection (SSI) in elective colorectal surgery. Dis Colon Rectum. 2020;63(6):e441. | Conference abstract |
| De Jonge SW, Atema JJ, Solomkin JS, Boermeester MA. Meta-analysis and trial sequential analysis of triclosan-coated sutures for the prevention of surgical-site infection. Br J Surg. 2017;104(2):e118-e33. | SR or MA for reference checking |
| De Jonge SW, Atema JJ, Solomkin JS, Boermeester MA. A meta-analysis using grade and trial sequential analysis of triclosan-coated sutures for the prevention of surgical site infection: Is the evidence final? J Am Coll Surg. 2016;223(4 suppl 1):e103. | Conference abstract |
| Defazio A, Datta M, Nezhat C. Does the use of Vicryl Plus antibacterial suture decrease the incidence of umbilical infection when compared to Vicryl suture? Fertil Steril. 2005; (suppl 1): S161. Available from: https://www.sciencedirect.com/science/article/abs/pii/S0015028205018546?via%3Dihub | Conference abstract |
| Deliaert AE, Van den Kerckhove E, Tuinder S, Fieuws S, Sawor JH, Meesters-Caberg MA, et al. The effect of triclosan-coated sutures in wound healing. A double blind randomised prospective pilot study. J Plast Reconstr Aesthet Surg. 2009;62(6):771-3. | Ineligible study design |
| Dinis P, Nunes P, Mota A. Comparison between the use of barbed and polyglactin sutures in urologic laparoscopic surgery - a systematic review. Acta Urologica Portuguesa. 2016;33(2):51-56. | Ineligible study design |
| Dr Prerna Karde. Comparative evaluation antimicrobial sutures versus plain sutures in periodontal flap surgery. In: Clinical Trials Registry - India (CTRI) [internet]. New Delhi. National Institute of Medical Statistics. 2017. Available from http://ctri.nic.in/Clinicaltrials/pmaindet2.php?trialid=20418&EncHid=&userName=2017/09/009940. Identifier: CTRI/2017/09/009940 | Reports no eligible outcomes |
| Elsolh B, Zhang L, Patel SV. The Effect of Antibiotic-Coated Sutures on the Incidence of Surgical Site Infections in Abdominal Closures: a Meta-Analysis. J Gastrointest Surg. 2017;21(5):896-903. | SR or MA for reference checking |
| Ethicon Inc. A Study of Two Types of Absorbable Surgical Sutures in the Suturing of Thyroid Surgery Incision. In: ClinicalTrials.gov [internet]. Bethesda. US National Library of Medicine. 2019. Available from https://clinicaltrials.gov/show/NCT03792737. Identifier: NCT03792737 | Ineligible comparator |
| Ethicon Inc. A Study of Two Types of Absorbable Surgical Sutures in the Suturing of Thyroid Surgery Incision. In: ClinicalTrials.gov [internet]. Bethesda. US National Library of Medicine. Available from https://ClinicalTrials.gov/show/NCT03792737. Identifier: NCT03792737 | Ineligible comparator |
| Evangelical Community Hospital Lewisburg. Study to Compare Suture Material in Closure of Uterine Incision in Cesarian Section. In: ClinicalTrials.gov [internet]. Bethesda. US National Library of Medicine. 2014. Available from https://clinicaltrials.gov/show/NCT02517710. Identifier: NCT02517710 | Reports no eligible outcomes |
| Giampaolino P, De Rosa N, Tommaselli GA, Santangelo F, Nappi C, Sansone A, et al. Comparison of bidirectional barbed suture Stratafix and conventional suture with intracorporeal knots in laparoscopic myomectomy by office transvaginal hydrolaparoscopic follow-up: a preliminary report. Eur J Obstet Gynecol Reprod Biol. 2015;195:146-50. | Ineligible intervention |
| Giampaolino P, Santangelo F, De Rosa N, Pellicano M, Nappi C. Comparison of bidirectional barbed suture stratafix and conventional suture with intracorporeal knots in laparoscopic myomectomy. Gynecol Surg. 2015; 12(suppl 1): S318. Available from: https://link.springer.com/article/10.1007%2Fs10397-015-0918-0 | Ineligible intervention |
| Grin L, Ivshin A, Rabinovich M, Namazov A, Shochat V, Shperberg A, et al. Barbed suture versus vicryl suture for uterine incision repair during a C-section: a randomised, controlled, assessor-blind trial. BJOG. 2018; 125(suppl 1): 70‐71. Available from: https://obgyn.onlinelibrary.wiley.com/doi/10.1111/1471-0528.7_15132 | Ineligible intervention |
| Guo J, Pan L-H, Li Y-X, Yang X-D, Li L-Q, Zhang C-Y, et al. Efficacy of triclosan-coated sutures for reducing risk of surgical site infection in adults: a meta-analysis of randomized clinical trials. J Surg Res. 2016;201(1):105-17. | SR or MA for reference checking |
| Gupta M. Antimicrobial coated sutures in Indian Market: A literature review of efficacy and safety in patients to prevent surgical site infections. J Indian Med Assoc. 2019;117(6):19-23. | Ineligible study design |
| Gys B, Gys T, Lafullarde T. The use of knotless barbed versus traditional suture for anastomosis closure in RYGB: preliminary results of an RCT. Obes Surg. 2015; 25(suppl 1): S45‐s46. Available from: https://link.springer.com/content/pdf/10.1007/s11695-015-1750-3.pdf | Ineligible intervention |
| Gys B, Gys T, Lafullarde T. The Use of Unidirectional Knotless Barbed Suture for Enterotomy Closure in Roux-en-Y Gastric Bypass: a Randomized Comparative Study. Obes Surg. 2017;27(8):2159-63. | Ineligible intervention |
| Gys B, Gys T, Lafullarde T. The use of unidirectional knotless barbed suture for enterotomy closure in Roux-en-y gastric bypass: a randomized comparative studyudy new (non standard) surgical techniques. Obes Surg. 2017; 27(1): 692. Available from: https://link.springer.com/content/pdf/10.1007/s11695-017-2774-7.pdf | Ineligible intervention |
| Han Y, Yang W, Pan J, Zeng L, Liang G, Lin J, et al. The efficacy and safety of knotless barbed sutures in total joint arthroplasty: a meta-analysis of randomized-controlled trials. Arch Orthop Trauma Surg. 2018;138(10):1335-45. | Ineligible intervention |
| Hayes Inc. Antibacterial suture for prevention of infection. Lansdale PA: Hayes Inc; 2011. Available from: http://www.hayesinc.com/hayes/crd/?crd=12022. | Document unobtainable |
| Hayes Inc. Antibiotic-coated sutures. Lansdale PA: Hayes Inc; 2012. Available from: http://www.hayesinc.com/hayes/crd/?crd=13609. | Document unobtainable |
| Hayes Inc. Comparative effectiveness review of antimicrobial versus conventional sutures. Lansdale PA: Hayes Inc; 2017. Available from: The report may be purchased from:http://www.hayesinc.com/hayes/crd/?crd=13609. | Document unobtainable |
| Heger P, Pianka F, Diener MK, Mihaljevic AL. [Current standards of abdominal wall closure techniques : Conventional suture techniques]. Chirurg. 2016;87(9):737-43. | Non-English publication |
| Henriksen NA, Deerenberg EB, Venclauskas L, Fortelny RH, Garcia-Alamino JM, Miserez M, et al. Triclosan-coated sutures and surgical site infection in abdominal surgery: the TRISTAN review, meta-analysis and trial sequential analysis. Hernia. 2017;21(6):833-41. | SR or MA for reference checking |
| Henriksen N, Deerenberg E, Venclauskas L, Fortelny R, Miserez M, Muysoms F. Triclosan-coated sutures and surgical site infection in abdominal surgery. A meta-analysis. Hernia. 2017;21(2 suppl 1):S166. | Conference abstract |
| Hughes J, Ballard DH, Macieski F, Ho MTT, Caldito G, Valiulis J. Wound Breakdown with Stratafix versus Monocryl Suture in Aesthetic and Reconstructive Plastic Surgery: Data from a Single Surgeon. Am Surg. 2017;83(1):e4-5. | Ineligible study design |
| Hunger R, Mantke A, Herrmann C, Mantke R. [Triclosan-coated sutures in colorectal surgery : Assessment and meta-analysis of the recommendations of the WHO guideline]. Chirurg. 2019;90(1):37-46. | Non-English publication |
| Huszár O, Baracs J, Tóth M, Damjanovich L, Kotán R, Lázár G, et al. Comparison of wound infection rates after colon and rectal surgeries using triclosan-coated or bare sutures -- a multi-center, randomized clinical study. Magyar sebeszet. 2012; 65(3): 83‐91. Available from: https://akjournals.com/view/journals/1046/65/3/article-p83.xml | Non-English publication |
| Icahn School of Medicine at Mount Sinai. Barbed Suture for Hysterotomy Closure During Cesarean Section. In: ClinicalTrials.gov [internet]. Bethesda. US National Library of Medicine. 2020. Available from https://clinicaltrials.gov/show/NCT04622267. Identifier: NCT04622267 | Ineligible comparator |
| Islamic Azad University. Accumulation of oral microorganisms around the suture materials in implant surgery. In: Iranian Registry of Clinical Trials [internet]. Tehran. Ministry of Health and Medical Education (MOHME), Iran University of Medical Sciences (IUMS). 2019. Available from http://en.irct.ir/trial/36296. Identifier: IRCT20180617040117N | Ineligible study design |
| Islamic Azad University. Accumulation of oral microorganisms around the suture materials in implant surgery. In: Iranian Registry of Clinical Trials [internet]. Tehran. Ministry of Health and Medical Education (MOHME), Iran University of Medical Sciences (IUMS). 2019. Available from http://en.irct.ir/trial/35214. Identifier: IRCT20180714040460N | Ineligible study design |
| Islamic Azad University. To investigate the effect of vicryl and vicryl plus sutures on wound situation after lower jaw impacted third molars surgery. In: Iranian Registry of Clinical Trials [internet]. Tehran. Ministry of Health and Medical Education (MOHME), Iran University of Medical Sciences (IUMS). 2017. Available from https://en.irct.ir/trial/20475. Identifier: IRCT2015092424167N | Ineligible study design |
| Jiang C, Huang D-G, Yan L, Hao D-J. The efficacy of triclosan coated sutures for preventing surgical site infections in orthopedic surgery: A systematic review and meta-analysis. Asian J Surg. 2020; 44(2): 506-07. Available from: https://www.sciencedirect.com/science/article/pii/S101595842030378X?via%3Dihub | SR or MA for reference checking |
| Johnson & Johnson Medical China. Symmetric on Total Knee Arthoplasty (TKA). In: ClinicalTrials.gov [internet]. Bethesda. US National Library of Medicine. 2017. Available from https://clinicaltrials.gov/show/NCT03305887. Identifier: NCT03305887 | Ineligible intervention |
| Jeppsson A, Thimour-Bergstrom L, Gudbjartsson T, Aneman C, Friberg O. Triclosan-coated sutures reduce surgical site infections after open vein harvesting in coronary artery bypass graft patients: A prospective randomized controlled trial. Interact Cardiovasc Thorac Surg. 2012;15(suppl 2):S134. | Conference abstract |
| Karde PA, Sethi KS, Mahale SA, Mamajiwala AS, Kale AM, Joshi CP. Comparative evaluation of two antibacterial-coated resorbable sutures versus noncoated resorbable sutures in periodontal flap surgery: A clinico-microbiological study. J Indian Soc Periodontol. 2019;23(3):220-25. | Reports no eligible outcomes |
| Khachatryan N, Dibirov M, Omelyanovsky V, Chupalov M, Gasanova G. Prevention of postoperative infections in abdominal surgery using reabsorbable suture with antibacterial activity (Vicryl Plus) versus reabsorbable standard sutures. Surg Infect (Larchmt). 2011; 12(2): A13‐4. Available from: https://www.liebertpub.com/doi/pdfplus/10.1089/sur.2011.9918 | Conference abstract |
| Knaebel HP, Kirschner MH, Reidel MA, Büchler MW, Seiler CM. Operative standardization in randomized controlled surgical trials. Meeting of the INSECT trial. Chirurg. 2006; 77(3): 267‐72. Available from: https://link.springer.com/content/pdf/10.1007/s00104-005-1149-0.pdf | Non-English publication |
| Konstantelias AA, Andriakopoulou CSI, Mourgela S. Triclosan-coated sutures for the prevention of surgical-site infections: a meta-analysis. Acta Chir Belg. 2017;117(3):137-48. | SR or MA for reference checking |
| Krishnamoorthy B, Shepherd N, Critchley WR, Nair J, Devan N, Nasir A, et al. A randomized study comparing traditional monofilament knotted sutures with barbed knotless sutures for donor leg wound closure in coronary artery bypass surgery. Interact Cardiovasc Thorac Surg. 2016;22(2):161-67. | Ineligible intervention |
| Leaper DJ, Edmiston CE, Jr., Holy CE. Meta-analysis of the potential economic impact following introduction of absorbable antimicrobial sutures. Br J Surg. 2017;104(2):e134-e44. | SR or MA for reference checking |
| Leonardo J, Rozzelle CJ. Antimicrobial suture use associated with a decreased incidence of cerebrospinal fluid shunt infections. Neurosurgery. 2006;59(2):478-78. | Conference abstract |
| Li D, Zhuang J, Liu YG, Zhou H, Chen KX, Cheng K, et al. Full fascia closure with interrupted absorbable suture and layered closure with interrupted silk suture in abdominal incision: comparison of curative effects and biocompatibility. CJTER. 2014; 18(43): 6996‐7000. Available from: https://www.cochranelibrary.com/central/doi/10.1002/central/CN-01096367/full | Non-English publication |
| Mahajan N, Pillai R, Chopra H, Grover A, Kohli A. An economic model to assess the value of triclosan-coated sutures in reducing the risk of surgical-site infection in coronary artery bypass graft in India. J Indian Coll Cardiol. 2020;10(2):79-84. | Ineligible study design |
| Mattavelli I, Nespoli L, Alfieri S, Cantore F, Sebastian-Douglas S, Cobianchi L. Triclosan-coated suture to reduce surgical site infection after colorectal surgery. Surg Infect (Larchmt). 2011; 12(2): A14‐A15. | Conference abstract |
| Meyer R, Sivan E, Sharon N, Fishel-Bartal M, Kalter A, Derazne E, et al. Infectious morbidity following cesarean deliveries: A comparison of barbed to standard suture for myometrial closure. Am J Obstet Gynecol. 2018;218(1 suppl 1):S335-S36. | Ineligible intervention |
| Mitchell MD, Betesh J, Umscheid C. Antimicrobial sutures for prevention of surgical infections. Pennsylvania: Penn Medicine Center for Evidence-based Practice (CEP); 2010. | Ineligible document type |
| Mohamed Zayed. Uterine Closure at C-section by Stratafix Tissue Control Device: randomized Case-Control Study. In: ClinicalTrials.gov [internet]. Bethesda. US National Library of Medicine. 2014. Available from https://clinicaltrials.gov/show/NCT02288013. Identifier: NCT02288013 | Ineligible intervention |
| Morioka Municipal Hospital. Does antimicrobial triclosan-coated PDS PLUS for skin closure reduce surgical site infections? A controlled clinical trial of class II abdominal surgeries. In: UMIN Clinical Trials Registry [internet]. Tokyo. University of Tokyo Hospital. 2016. Available from https://upload.umin.ac.jp/cgi-open-bin/ctr_e/ctr_view.cgi?recptno=R000025218. Identifier: JPRN-UMIN000021892 | Non-English publication |
| Mulder T, Abbas M, Harbarth S, Kluytmans J. Triclosan-coated sutures reduce the risk of surgical site infections: A systematic review and meta-analysis. Antimicrob Resist Infect Control. 2019;8(suppl 1):P21. | Conference abstract |
| NYU Langone Health. Knotless Suture in Revision Total Joint Arthroplasty. In: ClinicalTrials.gov [internet]. Bethesda. US National Library of Medicine. 2020. Available from https://ClinicalTrials.gov/show/NCT04403919. Identifier: NCT04403919 | Ineligible comparator |
| Olmez T, Colak T. The effect of triclosan coated suture material on surgical site infection of abdominal facial closure. Eur Surg Res. 2015;55(suppl 1):66-67. | Conference abstract |
| O'Neal PB, Itani KMF. Antimicrobial Formulation and Delivery in the Prevention of Surgical Site Infection. Surg Infect (Larchmt). 2016;17(3):275-85. | Ineligible study design |
| Onesti MG, Carella S, Scuderi N. Effectiveness of antimicrobial-coated sutures for the prevention of surgical site infection: a review of the literature. Eur Rev Med Pharmacol Sci. 2018;22(17):5729-39. | Ineligible study design |
| Osaka Prefectural Adult Disease Center. A randomized controlled trial of preventative effect on wound complication after gastrointestinal surgery by coated antibacterial suture. In: UMIN Clinical Trials Registry [internet]. Tokyo. University of Tokyo Hospital. 2009. Available from https://upload.umin.ac.jp/cgi-open-bin/ctr/ctr_view.cgi?recptno=R000003117. Identifier: JPRN-UMIN000002550 | Ineligible intervention |
| Otani N, Tomita K, Taminato M, Yano K, Hosokawa K. Efficacy of STRATAFIX in Inframammary Fold Recreation in Autologous Breast Reconstruction. Plast. 2018;6(4):e1702. | Ineligible study design |
| Peleg D, Ahmad RS, Warsof SL, Marcus-Braun N, Sciaky-Tamir Y, Ben Shachar I. A randomized clinical trial of knotless barbed suture vs conventional suture for closure of the uterine incision at cesarean delivery. Am J Obstet Gynecol. 2018;218(3):343. | Ineligible intervention |
| Pelz K, Todtmann N, Otten J-E. Comparison of antibacterial-coated and non-coated suture material in intraoral surgery by isolation of adherent bacteria. Ann Agric Environ Med. 2015;22(3):551-5. | Ineligible study design |
| Region Skane. Comparison of VicrylPlus® Versus Vicryl® for Repair of Perineal Tears. In: ClinicalTrials.gov [internet]. Bethesda. US National Library of Medicine. 2016. Available from https://clinicaltrials.gov/show/NCT02863874. Identifier: NCT02863874 | Ineligible population |
| Room H, Roberts G, Parwaiz H, Gergely S. Antibacterial coated sutures reduce laparoscopic post-operative surgical site infections. Br J Surg. 2013;100(suppl 7):212-13. | Ineligible study design |
| Roy PK, Kalita P, Lalhlenmawia H, Dutta RS, Thanzami K, Zothanmawia C, et al. Comparison of surgical site infection rate between antibacterial coated surgical suture and conventional suture: A randomized controlled single centre study for preventive measure of postoperative infection. IJPSR. 2019;10(5):2385-91. | Ineligible intervention |
| Sakaguchi H, Singh H, Klima U, Lee CN, Kofidis T. Antibacterial suture reduces surgical site infections in coronary artery bypass grafting. In: 17th Annual Meeting of the Asian society for Cardiovascular and Thoracic Surgery (ASCVTS); March 5-8 2009: Taipei: Asian Society for Cardiovascular & Thoracic Surgery; 111‐14. | Conference abstract |
| Sakdinakiattikoon M, Tanavalee A. Continuous barbed suture versus knotted interrupted suture for wound closure in total knee arthroplasty: A prospective randomized study. J Med Assoc Thai. 2019;102(3):361-67. | Ineligible intervention |
| Sala-Perez S, Lopez-Ramirez M, Quinteros-Borgarello M, Valmaseda-Castellon E, Gay-Escoda C. Antibacterial suture vs silk for the surgical removal of impacted lower third molars. A randomized clinical study. Med Oral Patol Oral Cir Bucal. 2016;21(1):e95-102. | Ineligible study design |
| Samsung Medical Center. Effects of Triclosan coated suture on the surgical site infection in gastric cancer surgery patients. In: Clinical Research Information Service (CRIS). 2011. Available from http://cris.nih.go.kr/cris/en/search/search_result_st01.jsp?seq=1421. Identifier: KCT0000209 | Ineligible study design |
| Sandini M, Mattavelli I, Nespoli L, Uggeri F, Gianotti L. Systematic review and meta-analysis of sutures coated with triclosan for the prevention of surgical site infection after elective colorectal surgery according to the PRISMA statement. Medicine. 2016;95(35):e4057. | SR or MA for reference checking |
| Sawada K, Nakayama K, Ishibashi T, Nakamura A, Yoshimura Y, Ono R, et al. A comparison of bidirectional stratafix bardcd suture with conventional suture for laparoscopic myomectomy. J Obstet Gynaecol Res. 2019;45(8):1744. | Ineligible intervention |
| Sedrakyan A. Precarious innovation of anti-infective coated devices. Lancet. 2014;384(9938):111-3. | Ineligible document type |
| Seoul National University Hospital. Effect of Barbed Suture Fascia Closure on Incisional Hernia in Midline Laparotomy for Gynecological Diseases (BARBHER). In: ClinicalTrials.gov [internet]. Bethesda. US National Library of Medicine. 2020. Available from https://ClinicalTrials.gov/show/NCT04643197. Identifier: NCT04643197 | Ineligible comparator |
| Serlo W, Renko M, Paalanne N, Tapaiainen T, Hinkanen M, Pokka T, et al. Triclosan-coated sutures in preventing surgical site infection in children: a randomized controlled series. Child's nervous system : ChNS : official journal of the International Society for Pediatric Neurosurgery. 2016; 32(suppl): 1983. Available from: https://link.springer.com/content/pdf/10.1007/s00381-016-3209-9.pdf | Conference abstract |
| Singh H, Emmert MY, Sakaguchi H, Neng Lee C, Kofidis T. Antibacterial suture reduces surgical site infections in coronary artery bypass grafting. Heart surgery forum. 2010; 13(suppl 2): S85. Available from: https://journal.hsforum.com/index.php/HSF/article/view/508 | Conference abstract |
| Spital Limmattal Schlieren. Vaginal Stump Infection After Laparoscopic Hysterectomy. In: ClinicalTrials.gov [internet]. Bethesda. US National Library of Medicine. 2018. Available from https://ClinicalTrials.gov/show/NCT04725981. Identifier: NCT04725981 | Ineligible intervention |
| Sprowson AP, Jensen C, Ahmed I, Parsons N, Partington P, Emmerson K, et al. Infographic: Triclosan-coated sutures and surgical site infections after hip and knee arthroplasty. Bone Joint J. 2018;100-B(3):294-95. | Conference abstract |
| St. Franziskus Hospital. Stratafix vs. Vicryl OAGB / MGB Suture Study. In: ClinicalTrials.gov [internet]. Bethesda. US National Library of Medicine. 2020. Available from https://clinicaltrials.gov/show/NCT04613635. Identifier: NCT04613635 | Ineligible intervention |
| Surgical Infection Society Europe. 26th European Congress on Surgical Infection. In: 26th European Congress on Surgical Infection 2013: Prague: Mary Ann Liebert Inc.; Surg Infect (Larchmt). 2013;14(2):A1-A17. Available from: https://www.liebertpub.com/doi/full/10.1089/sur.2013.9994 | Conference abstract |
| Tseng CH. Evidence-based effects of triclosan-coated sutures for the prevention of surgical-site infection. Int J Antimicrob Agents. 2017;50(suppl 2):S237. | Ineligible study design |
| Uchino M, Mizuguchi T, Ohge H, Haji S, Shimizu J, Mohri Y, et al. The Efficacy of Antimicrobial-Coated Sutures for Preventing Incisional Surgical Site Infections in Digestive Surgery: a Systematic Review and Meta-analysis. J Gastrointest Surg. 2018;22(10):1832-41. | SR or MA for reference checking |
| University Hospital Basel. Clinical Outcome in View of Surgical Site Infection (SSI) With Antibacterial Skin Sutures. In: ClinicalTrials.gov [internet]. Bethesda. US National Library of Medicine. 2012. Available from https://clinicaltrials.gov/show/NCT01540279. Identifier: NCT01540279 | Ineligible study design |
| University Hospital Freiburg. Oral Bacteria on Suture Materials - Clinical Comparison of an Antibacterial-coated and a Non-coated Suture Material. In: ClinicalTrials.gov [internet]. Bethesda. US National Library of Medicine. 2009. Available from https://clinicaltrials.gov/show/NCT00946049. Identifier: NCT00946049 | Ineligible study design |
| University Hospital Maastricht Department of Plastic Surgery. The effect of triclosan coated sutures in wound healing. A double blind randomized prospective pilot study. In: Nederlands Trial Register [internet]. Amsterdam. The Dutch Cochrane Centre. 2007. Available from https://www.trialregister.nl/trial/957. Identifier: NTR983 | Ineligible study design |
| University of Pecs. Abdominal Wall Closure With Triclosan-coated Suture. In: ClinicalTrials.gov [internet]. Bethesda. US National Library of Medicine. 2010. Available from https://clinicaltrials.gov/show/NCT01620294. Identifier: NCT01620294 | Reports no eligible outcomes |
| Won HS, Lee SW, Kim YM, Kim A. Clinical usefulness and safety of the anti-bacterial coated multifilament suture (Vicryl Plus) and monofilament suture (Monosyn) in hysterectomy. BJOG. 2012;119(suppl 1):44. | Reports no eligible outcomes |
| Wu X, Kubilay NZ, Ren J, Allegranzi B, Bischoff P, Zayed B, et al. Antimicrobial-coated sutures to decrease surgical site infections: a systematic review and meta-analysis. Eur J Clin Microbiol Infect Dis. 2017;36(1):19-32. | SR or MA for reference checking |
| Wu X, Kubilay NZ, Ren J, Allegranzi B, Bischoff P, Zayed B, et al. Correction to: Antimicrobial-coated sutures to decrease surgical site infections: a systematic review and meta-analysis. Eur J Clin Microbiol Infect Dis. 2018;37(10):2031-34. | SR or MA for reference checking |
| Yasuda S, Tomita K, Kiya K, Hosokawa K. STRATAFIX for Abdominal Wall Repair following Abdominal Flap Harvest. Plast. 2017;5(11):e1572. | Ineligible study design |
| Yam JM, Orlina EA. Effectiveness of antimicrobial sutures in preventing surgical site infection in clean-contaminated wounds-a preliminary study. Surgical infections. 2013; 14(suppl 1): S29. Available from: https://www.liebertpub.com/doi/pdfplus/10.1089/sur.2013.9996 | Conference abstract |
| Yanazume S, Togami S, Fukuda M, Kawamura T, Kamio M, Ota S, et al. New Continuous Barbed Suture Device with Stratafix for the Vaginal Stump in Laparoscopic Hysterectomy. Gynecol Minim Invasive Ther. 2018;7(4):167-71. | Ineligible study design |
| Yanazume S, Togami S, Fukuda M, Kamio M, Karakida N, Ota S. Utility of continuous sutures by STRATAFIX for closing vaginal stump in total laparoscopic hysterectomy. J Obstet Gynaecol Res. 2018;44(8):1590. | Conference abstract |
| Ye Z, Zhu W, Xi X, Wu Q. The efficacy of bidirectional barbed sutures for incision closure in total knee replacement: A protocol of randomized controlled trial. Medicine. 2020;99(34):e21867. | Ineligible intervention |
| Zayed MA, Fouda UM, Elsetohy KA, Zayed SM, Hashem AT, Youssef MA. Barbed sutures versus conventional sutures for uterine closure at cesarean section; a randomized controlled trial. J Matern Fetal Neonatal Med. 2019;32(5):710-17. | Ineligible intervention |
| Zhuang CP, Cai GY, Wang YQ. Comparison of two absorbable sutures in abdominal wall incision. CRTER. 2009; 13(21): 4045‐48. | Non-English publication |
| Ziv Hospital. Trial Comparing Barbed and Non-barbed Suture for Uterine Incision Closure at Cesarean Section. In: ClinicalTrials.gov [internet]. Bethesda. US National Library of Medicine. 2016. Available from https://clinicaltrials.gov/show/NCT02962011. Identifier: NCT02962011 | Ineligible intervention |

### Supplementary_Table_4: Studies included in the review

| **Study**  *Author, year, location* | **Study design**  *Include details of single / double blind if reported* | **Inclusion criteria** | **Patient population, setting, and withdrawals/lost to follow up** | **Intervention and comparator** | **Main outcomes**  *Note primary and secondary outcomes* |
| --- | --- | --- | --- | --- | --- |
| Arslan 2018, Turkey  Source: ^[20]^ | Randomised trial  Partially-blinded: the operating surgeon was not blinded as they recognised the sutures, whereas postoperative care and assessment of the surgical site were conducted by another surgeon, and was thus presumably blinded. Blinding of the patients was not reported | Patients ≥18 years old who underwent wide excision and primary closure for pilonidal disease | Adult patients undergoing wide excision and primary closure for pilonidal disease  Unspecified number of hospital surgical departments in Turkey  **PDS Plus + Vicryl Plus**: analysed (treated patients) n=86  92 randomized; 6 protocol violations  **Prolene + Vicryl**: analysed (treated patients) n=91  95 randomized; 4 protocol violations | Intervention: Triclosan-coated sutures (PDS Plus + Vicryl Plus)  Comparators(s): Uncoated sutures (Prolene + Vicryl) | Primary end-point: rate of SSI as defined by CDC guidelines (2017)  Secondary end-points: wound dehiscence without infection and rate of seroma. |
| Baracs 2011, Hungary  Other identifiers: NCT01123616  Source:  Primary: ^[21]^  Secondary: ^[22]^ | Multicentre, randomised study  NCT record states that masking was “double (Care Provider, Outcomes Assessor)” | Age between 18 and 80 years with benign or malignant colon or rectal disease undergoing an elective open surgical procedure involving an enterotomy | Adult patients up to 80 years of age undergoing an elective open surgical procedure involving an enterotomy  Patients attending seven Hungarian surgical institutions (3 university clinics and 4 high-volume hospitals)  **Total**: randomised 385  **PDS Plus:** randomised n = 188  **PDS II:** randomised n = 197  Patient withdrawals by arm NR  468 patients were suitable for randomisation, but 83 (18.1%) were excluded later. *(Inoperable tumor (45 cases; 54.2%), sepsis in the postoperative period (19 cases; 22.9%), breach of protocol (eight cases; 9.6%), patient request (two cases; 2.4%), and unsuccessful bowel preparation (nine cases; 10.8%))* | Intervention: Triclosan-coated sutures (PDS Plus)  Comparator(s): Uncoated sutures (PDS II) | Primary goals were to determine whether triclosan-coated polydiaxanone is able to reduce the number of SSIs after colorectal surgery  Secondary goals were to determine whether an SSI increases the length of the hospital stay, whether there are any additional costs, and the chances of late SSI after the patient has been discharged from the hospital |
| Diener 2014, Germany  Other identifiers: PROUD, DRKS00000390  Source:  Primary: ^[23]^  Secondary: ^[23-27]^ | Multicentre, randomised controlled group-sequential superiority trial  Patients, surgeons, and the outcome assessors were masked to the suture material used | Adult patients (aged ≥18 years) who underwent elective midline abdominal laparotomy for any reason | Adult patients undergoing elective midline abdominal laparotomy  24 secondary and tertiary care centres in Germany  **PDS Plus:** mITT = 587, PP = 451  607 allocated. 3 excluded, 108 terminated prematurely, 136 excluded from PP population  **PDS II:** mITT = 598, PP = 462  617 allocated. 2 excluded, 118 terminated prematurely, 136 excluded from PP population | Intervention: Triclosan-coated sutures (PDS Plus)  Comparator(s): Uncoated sutures (PDS II) | Primary endpoint: the occurrence of superficial or deep surgical site infection (according to the CDC Control and Prevention criteria) within 30 days of the operation  Secondary endpoints: frequency of wound dehiscence (cutaneous and subcutaneous layer), frequency of burst abdomen (fascial dehiscence), postoperative length of stay in intensive care unit, postoperative length of stay in hospital, 30-day mortality, and quality of life (collected using the EQ-5D questionnaire) |
| Ford 2005, USA  Source: ^[28]^ | Single-centre, open-label, RCT  Reported to be open-label, but no specific details provided except for the blinded assessment of the primary endpoint (overall intraoperative handling characteristics) | Children aged 1 to 18 years who were scheduled for clean or clean-contaminated surgical procedures | Paediatric patients scheduled for any general, clean or clean-contaminated surgical procedure  NR explicitly but author affiliations suggest one hospital in the USA  Total: 151 enrolled and randomised  **Vicryl Plus**: Observed cases: n=98 (baseline), n=76 (study end)  100* randomised; 2 withdrew prior to treatment; 22* withdrawals/lost to follow-up  **Vicryl**: Observed cases: n=49 (baseline), n=38 (study end)  51* randomised; 2 withdrew prior to treatment; 11* withdrawals/lost to follow-up | Intervention: Triclosan-coated sutures (Vicryl Plus)  Comparator(s): Uncoated sutures (Vicryl) | Primary outcome: the surgeon’s assessment of the overall intraoperative handling of the triclosan-coated suture and traditional uncoated suture  Secondary outcomes:   - Specific intraoperative suture handling measures (ease of passage through tissue, first-throw knot holding, knot tie-down smoothness, knot security, surgical “hand,” memory, and degree of fraying) - Wound healing assessments (healing progress, infection, edema, erythema, skin temperature, seroma, suture sinus, pain) |
| Galal 2011, Egypt  Source: ^[29]^ | Multcentre, double-blind RCT  Double-blind, with none of the research team (surgeon, nurse, microbiologist) or the patients being aware of the allocated treatment | All patients of any age, sex, and risk factors who were candidates for surgical intervention during the study period | Candidates for any surgical procedure during the study period  Unspecified number of centres in Egypt  This article only reported the results from one site, a university hospital  **Vicryl Plus**: ITT n=230  230 enrolled; no withdrawals or loss to follow-up  **Vicryl**: ITT n=220  220 enrolled; no withdrawals or loss to follow-up | Intervention: Triclosan-coated sutures (Vicryl Plus)  Comparator(s): Uncoated sutures (Vicryl) | Primary outcomes: Not explicitly reported but focus was on SSI according to modified CDC criteria (1992) at 30 days (or 1 year in case of prosthetic surgery)  Secondary outcomes: NR but study assessed postoperative stay, costs and health resources |
| Ichida 2018, Japan  Other identifiers:  UMIN000013054  Source:  Primary: ^[30]^  Secondary: ^[31]^ | Single-centre, double-blind, randomised controlled group-sequential superiority trial  Patients, surgeons, nurses in the surgical wards, and outcome assessors were all blinded to treatment allocation. The sutures were identical in physical properties and were indistinguishable once removed from their packaging and any identification marks. | Patients of any age undergoing gastroenterologic surgery | Patients undergoing gastroenterologic surgery  One medical university in Japan  **Vicryl Plus**: mITT n=508  512 randomised; 4 did not receive intervention (2 operation cancelled, 2 administrative error); no loss to follow-up or withdrawals  **Vicryl**: mITT n=505  511 randomised; 6 did not receive intervention (4 operation cancelled, 2 administrative error); no loss to follow-up or withdrawals | Intervention: Triclosan-coated sutures (Vicryl Plus)  Comparator(s): Uncoated sutures (Vicryl) | Primary end point: incidence of superficial or deep SSIs according to the CDC criteria  Secondary end points: NR |
| Isik 2012, Turkey  Source: ^[32]^ | Single-centre, double-blind RCT  Reported to be double-blind. Patients were assigned the treatment during the operation, when the nurse delivered the suture materials to the operating room | Patients undergoing cardiac surgery at a private hospital | Patients undergoing cardiac surgery  One private hospital in Turkey  **Vicryl Plus**:ITT n=170; evaluable patients n=170 (sternal site) and n=142 (leg site)  270 randomised; withdrawals/lost to follow-up NR  **Vicryl:** ITT n=340; evaluable patients n=340 (sternal site) and n=260 (leg site)  340 patients randomised; withdrawals/lost to follow-up NR | Intervention: Triclosan-coated sutures (Vicryl Plus)  Comparator(s): Uncoated sutures (Vicryl) | Primary outcome: incidence of sternal and leg wound infections, according to CDC criteria  No secondary outcomes reported |
| Justinger 2013, Germany  Other identifiers:  NCT00998907  Source:  Primary: ^[33]^  Secondary: ^[34]^ | Single-centre, double-blind, randomised clinical pathway controlled trial  Surgeons, patients, and wound monitors were all blinded to treatment allocation. The sutures were indistinguishable in terms of their physical properties | Patients scheduled to undergo a laparotomy  From NCT record:   - Age ≥18 years - Surgical pathologies accessed via midline or transverse abdominal incision   Primary fascial closure | Adult patients undergoing elective laparotomy  One hospital in Germany  Overall: 1042 patients consented and included, of which 967 operated on per protocol, 111* patients excluded from analysis (12 patients with abdomen not closed, 18 early burst abdomen, 71 revisions, 10 deaths); 856 analysed  **PDS Plus**: analysed (treatment completers) n=485  559 operated on per protocol; 485 of the randomised patients were evaluated  **PDS II**: analysed (treatment completers) n=371  408 operated on per protocol; 371 of the randomised patients were evaluated | Intervention: Triclosan-coated sutures (PDS Plus)  Comparator(s): Uncoated sutures (PDS II) | Primary end point: the number of infections at the laparotomy incision during the hospital stay and 2-week follow-up post-discharge, with SSI defined according to CDC criteria  Secondary end points: NR  From NCT record:  The number of incisional hernias at 6 months and after long-term follow-up (12 and 24 months) |
| Karip 2016, Turkey  Source: ^[35]^ | Single-centre, double-blind RCT  Reported to be double-blind.  Patients were unaware of the treatments assigned and were not given any inforrmation about the nature of them. Blinding of the operating surgeon was not specified, but another surgeon conducted post-operative examinations unaware of treatment allocation | Patients with pilonidal sinus disease who were scheduled to undergo sinus excision followed by Karydakis flap repair | Adults aged 18 to 55 years who were scheduled for sinus excision followed by Karydakis flap repair for pilonidal sinus disease  One training and research hospital in Turkey  **Monocryl Plus:** ITT n=54  54 randomised and analysed; no apparent withdrawals/loss to follow up  **Monocryl:** ITT n=52  52 randomised; and analysed; no apparent withdrawals/loss to follow up | Intervention: Triclosan-coated sutures (Monocryl Plus)  Comparator(s): Uncoated sutures (Monocryl) | In the revised and approved trial, the primary outcome was infection rates at 1 and 2 weeks after surgery  Secondary outcomes:   - Incision dehiscence 1 and 2 weeks after surgery   Recurrence rates 1, 3 and 6 months after surgery |
| Lin 2018, Taiwan  Other identifiers:  NCT02533492  Source:  Primary: ^[36]^  Secondary: ^[37]^ | Double-blind RCT  Patients, clinical staff, operating surgeons, and the independent study nurse who collected perioperative and outcome data, were all blinded to the suture material allocated | - Men and women aged 55 to 85 years - Diagnosis of degenerative osteoarthritis of the knee - No prior surgery to the index knee   From NCT record  Varus/valgus deformity knee | Patients aged 55 to 85 years diagnosed with degenerative osteoarthritis of the knee who were scheduled for unilateral total knee arthroplasty  One hospital in Taiwan  **Vicryl Plus**: ITT n=51  No withdrawals or losses to follow-up; 51 randomised patients completed study  **Vicryl**: ITT n=51  No withdrawals or losses to follow-up; 51 randomised patients completed study | Intervention: Triclosan-coated sutures (Vicryl Plus)  Comparator(s): Uncoated sutures (Vicryl) | Primary outcome: incidence of SSI within 3 months of surgery.  Secondary outcomes included:   - Length of hospital stay - Pain level - Functional scores - Wound condition (wound drainage, extent of erythema, local heat, and skin surface temperature) - Inflammatory markers during hospitalisation and within 3 months postoperatively   From NCT record:  Duration of antibiotic use |
| Mattavelli 2015, Italy  Other identifiers:  NCT01869257  Source:  Primary: ^[38]^  Secondary: ^[39]^ | Multicentre, single-blind RCT  Patients and outcome assessors were blinded to treatment allocation. Operating surgeons could identifiy the sutures from their packaging | Candidates for elective colorectal resection with a clean-contaminated field  From NCT record:  Age 18 to 85 years | Adults aged 18 to 85 years who were candidates for elective colorectal resection  Four university referral hospitals in Italy  **Vicryl Plus + PDS Plus:** analysed (treatment completers) n=140  150 randomised and received intervention;10 discontinued due to need for re-operation; 0 lost to follow-up  **Vicry + PDS II**: analysed (treatment completers) n=141  150 randomised and received intervention; 9 discontinued due to need for re-operation; 0 lost to follow-up | Intervention: Triclosan-coated sutures (Vicryl Plus + PDS Plus)  Comparator(s): Uncoated sutures (Vicryl + PDS II) | Primary outcome: the overall rate of incisional SSI (superficial and deep), defined according to CDC criteria (1999) within 30 days after hospital discharge  Secondary outcomes:   - Length of hospital stay   Overall rate of incisional complications, including skin swelling and redness, hematomas, and seromas |
| Mingmalairak 2009, Thailand  Source: ^[40]^ | Single-centre double-blind RCT  The surgeons and attending doctor were blind to the type of suture | Patients aged 15-60 years-old, both sexes, with appendicitis diagnosed by intra-operative who operated with right lower quadrant incision. The study included both acute and ruptured appendix. | Patients aged 15-60 years undergoing surgery for appendicitis (including emergency surgery)  One university hospital in Thailand  Study is a report of the first 100 patients recruited and treated  **Vicryl Plus**: ITT n = 50  **Vicryl:ITT n = 50**  No patients in either arm were excluded following randomisation or lost to follow up after surgery | Intervention: Triclosan-coated sutures (Vicryl Plus)  Comparator(s): Uncoated sutures (Vicryl) | Primary outcome: To assess reduction of surgical site infection following appendectomy operations.  Secondary outcome: To analyse the safety and physical properties of Vicryl plus |
| Nakamura 2013, Japan  Other identifiers: UMIN00003322  Source:  Primary: ^[41]^  Secondary: ^[42]^ | Single-centre single-blind RCT  Patients and the physicians who assessed the wound infections were blinded to the treatment assignment None of the surgeons were blinded to the suture used. | Patients of any age who were undergoing elective colorectal operations  From UMIN record:  Patients presenting with indication for operation | Patients who were undergoing elective colorectal surgery  One hospital in Japan  **Vicryl Plus**: ITT n=206  206 randomised and received allocated intervention; 0 lost to follow-up, discontinued intervention, or excluded from analysis  **Vicryl:** ITT n=204  204 randomised and received allocated intervention; 0 lost to follow-up, discontinued intervention, or excluded from analysis | Intervention: Triclosan-coated sutures (Vicryl Plus)  Comparator(s): Uncoated sutures (Vicryl) | Primary outcome: number of wound infections, according to CDC guidelines (1999)  Secondary outcome: additional cost of care for infected wound management  From UMIN record: postoperative length of stay and their cost |
| Olmez 2019, Turkey  Source: ^[43]^ | RCT; unclear whether double or single blind  Patient follow-up and control tests were done by a blinded researcher | Patients who were 18 years old or older and underwent elective or urgent GI surgery for any reason | Patients 18 years + undergoing any GI surgery  Unclear whether single or multiple site, in Turkey  Total: 890 enrolled  **PDS Plus:** ITT n = 445  **PDS II:** ITT n = 445  All patients were analysed | Intervention: Triclosan-coated sutures (PDS Plus)  Comparator(s): Uncoated sutures (PDS II) | Primary and secondary outcomes not explicitly specified  Study aimed to compare PDS and PDS Plus for incidence of SSI following GI surgery |
| Rasic 2011, Croatia  Source: ^[44]^ | Single-centre RCT  Unclear whether patients and personnel were blinded to suture assignment  Sealed and numbered opaque envelopes containing suture packets were prepared | Patients scheduled for elective surgery for colorectal cancer during a 12-month period | Patients undergoing elective surgery for colorectal cancer between September 2008 and September 2009  One university hospital in Croatia  **Vicryl Plus**: analysed NR  91 randomised;  study discontinuations NR  **Vicryl**: analysed NR  93 randomised;  study discontinuations NR | Intervention: Triclosan-coated sutures (Vicryl Plus)  Comparator(s): Uncoated sutures (Vicryl) | Primary and secondary outcomes not explicitly specified.  Parameters recorded were:   - Duration of operation - Duration of hospitalisation - Biochemical inflammatory markers - Wound complications: wound infection, dehiscence, haematoma or inflammatory reactions to the skin sutures (skin inflammation around the suture) - Postoperative hernias - Readmissions - Reoperations |
| Renko 2017, Finland  Other identifiers:  NCT01220700  Source:  Primary: ^[45]^  Secondary: ^[46]^ | Single-centre, double-blind RCT  With the exception of the two nurses who masked the suture packages, the patients, their parents, and all study personnel were unaware of the treatment assignments | Children aged <18 years in the paediatric surgery and orthopaedics ward awaiting any elective or emergency surgery scheduled for a daytime paediatric operation room and with anticipated use of absorbing sutures  Written informed consent from parent or caregiver, or child (if aged 7-17 years and could read, write, and understand the trial protocol) | Children in the paediatric surgery and orthopaedics ward awaiting daytime elective or emergency surgery for any reason  One university hospital in Finland  **Triclosan-coated (Plus) sutures:** modified ITT n=778, PP n=636  Of 814 randomized, 802 had an operation; 166* excluded (1 death, 19 inclusion error, 4 lost to follow-up, 124 did not receive study suture material, 15 follow-up only up to 10 days, 3 other protocol violation)  **Control (non-coated) sutures**: modified ITT n=779, PP n=651  Of 819 randomized, 813 had an operation; 162* excluded (27 inclusion error, 7 lost to follow-up, 107 did not receive study suture material; 18 follow-up only up to 10 days, 3 other protocol violation) | Intervention: Triclosan-coated sutures (Vicryl Plus, Monocryl Plus, or PDS Plus)  Comparator(s): Uncoated sutures (Vicryl, Monocryl, or PDS) | Primary outcome: the occurrence of a superficial or deep SSI, according to CDC criteria, within 30 days after the operation  Secondary outcomes: NR |
| Rozzelle 2008, USA  Source: ^[47]^ | Single-centre double-blind RCT | Patients of all ages requiring CSF shunt implantation or revision surgery | Patients of all ages requiring CSF shunt implantation or revision surgery  One hospital in New York state, USA  84 shunt procedures were performed in 61 patients. Procedure types consisted of 40 implants and 44 revisions.  Patients receiving new shunts following successful treatment of a shunt infection and patients undergoing revision more than 6 months after randomization were rerandomized  N procedures analysed:  **Vicryl Plus:** 46  **Vicryl:** 38 | Intervention: Vicryl Plus  Comparator(s): Vicryl | Primary outcome: incidence of shunt infection within 6 months of CSF shunt placement surgery  Secondary outcomes: Additional data were recorded prospectively pertaining to demographics, procedure type/time, and patient factors believed to influence infection risk |
| Ruiz-Tovar 2020, Spain  Other identifiers: NCT03763279  Source:  Primary: ^[48]^  Secondary:  ^[49]^ | Multicentre, randomised clinical trial  Double-blind trial in terms of patients and outcome assessors (nurses and non-operating surgeon) masked to treatment assignment  The surgeon knew the treatment assignment before initiating the surgery but was masked to treatment prior to that point  All wounds were checked daily during hospital stay by an epidemiology nurse, blinded to group allocation and 30 days after operation  Presence of evisceration was determined by a surgeon on the team, blinded to group allocation | Patients undergoing emergency surgery by laparotomy and midline approach, for community-acquired infection, peritoneal contamination secondary to perforation of the digestive tract, and ischemia of a segment of digestive tract requiring resection  From NCT record:   - Adults aged ≥18 years - Contaminated and dirty surgery - Included the following diagnosis: anastomotic leak of previous digestive surgery, colonic or bowel perforations, appendicitis, perforation of gastric or duodenal ulcer, intestinal ischemia | Adult patients undergoing emergency surgery by laparotomy and midline approach  Spanish hospitals  **Stratafix Symmetric**: PP =47  50 randomised; 0 lost to follow-up and study discontinuation; 3 excluded from analysis (2 re-operation, 1 mortality)  **PDS Plus Loop:** PP = 45  50 randomized; 0 lost to follow-up and study discontinuation; 5 excluded from analysis (3 re-operation, 2 mortality)  **PDS Loop**: PP = 47  50 randomised; 0 lost to follow-up and study discontinuation; 3 excluded from analysis (2 re-operation, 1 mortality)  Patients with post-enrolment events, such as reoperation, deceased, or lost to follow-up during the first 30 days postoperatively, and patients planned for a second-look surgery were excluded from the final analysis | Intervention:  (1) Triclosan-coated barbed suture (Stratafix Symmetric PDS Plus)  (2) Triclosan-coated non-barbed suture (PDS Plus Loop)  Comparator(s): Uncoated sutures (PDS Loop) | Primary endpoints: rates of incisional SSI and evisceration during follow up period of 30 days (evaluated according to the CDC definitions of SSI)  Secondary endpoints: postoperative pain and analytical acute phase reactants (48 hours after operation), and identification of micro-organisms present any incisional SSIs when present |
| Ruiz-Tovar, 2015, Spain  Source: ^[50]^ | Multicentre, randomised clinical trial  Those who made the diagnosis were not blinded to the treatment, but were blinded to the selection of the patient from the sequentially numbered container. Epidemiology nurse who evaluated the outcome of the surgical incision was the only person blinded to the allocated treatment | Inclusion criteria were intra-operative diagnosis of fecal peritonitis secondary to acute diverticulitis perforation, neoplastic tumor perforation, or colorectal anastomotic leak of previous elective colorectal resection. | Patients undergoing abdominal wall closure after presenting with fecal peritonitis  Two hospitals in Spain  Total randomised: 110  9 patients died before an assessment of SSI could be made  **Triclosan-coated sutures:** n analysed = 50  **Uncoated sutures:** n analysed = 51 | Intervention: Triclosan-coated sutures (brand NR)  Comparator(s): Uncoated sutures (brand NR) | Primary and secondary endpoints not explicitly reported but the aim of the study was to assess the effect of triclosan-coated sutures on the incidence of SSI in dirty surgery |
| Santos 2019, Brazil  Source: ^[51]^ | Single-centre double-blind RCT  Randomisation remained blinded to all participants in the surgical procedure, as well as to all those who were involved in its follow-up, except for the professionals responsible for randomisation and masking | Patients who underwent consecutively, prospectively, and exclusively on-pump and off-pump CABG, of both genders, and aged >30 years met the inclusion criteria for the study | Patients aged over 30 years undergoing saphenectomy during coronary artery bypass graft (CABG), with and without cardiopulmonary bypass (CPB)  One teaching hospital in Brazil  **Vicryl Plus**: Analysed (completers) n=251  289 allocated. 26 did not show up to at least two follow up appointments, and 12 died  **Vicryl:** Analysed (completers) n=257  294 allocated. 26 did not show up to at least two follow up appointments, and 11 died | Intervention: Triclosan-coated sutures (Vicryl plus)  Comparator(s): Uncoated sutures (Vicryl) | Primary and secondary outcomes not explicitly specified  The study measured the SSI rate (definition NR) wound pain, and wound hyperthermia |
| Seim 2012, Norway  Source: ^[52]^ | Single-centre randomised trial  All surgeons were aware of the suture material used. Blinding of the patients and outcomes assessors was not reported | Patients undergoing elective coronary artery bypass grafting | Patients undergoing elective coronary artery bypass grafting  One hospital in Norway  **Vicryl Plus**: analysed (treatment completers) n=160  164 randomised; 4 lost to follow-up  **Vicryl**: analysed (treatment completers) n=163  164 randomised; 1 lost to follow-up | Intervention: Triclosan-coated sutures (Vicryl Plus)  Comparator(s): Uncoated sutures (Vicryl) | Primary and secondary outcomes not explicitly specified.  The study examined the incidence of leg wound infections, and predictors of infection related to patient- and operative characteristics |
| Soomro 2017, Pakistan  Source: ^[53]^ | Single-centre randomised controlled trial  The principal investigator was blinded to suture allocation Surgery “was performed by 3^rd^ and 4^th^ year residents to avoid surgeon bias” | Patients undergoing minor clean surgery for benign breast pathologies (e.g., fibroadenoma), aged between 20 to 35 years | Patients undergoing minor clean surgery for benign breast pathologies, aged 20-35 years  One hospital in Karachi (Liaquat National Hospital)  **Triclosan-coated sutures:** ITT 189  **Plain sutures:** ITT 189  Loss to follow up: NR | Intervention: Triclosan-coated sutures (brand NR)  Comparator(s): Uncoated sutures (brand NR) | Primary and secondary outcomes not explicitly specified  The purpose of the study was to compare the frequency of infection in simple polyglactin versus triclosan-coated suture material in benign breast surgeries |
| Sprowson 2018, UK  Other identifiers:  ISRCTN17807356  Source:  Primary: ^[54]^  Secondary: ^[55]^ | Multi-centre, double-blind quasi-RCT  The patients, research team, statistician, clinical staff and outcome assessors were all blinded to the treatment allocated. The participating surgeons were aware of the treatment allocation. “Associates” were also blinded, although it is unclear what their role was. | - Age >18 years, of either gender - Medically fit for an operation - Suitable for total hip arthroplasty or total knee arthroplasty, to be conducted by an orthopaedic consultant working at the Trust - Willing to give informed consent   Negative MRSA swab prior to surgery | Adults over 18 years undergoing elective, primary total hip arthroplasty or total knee arthroplasty  Three hospitals in the UK  **Vicryl Plus:** mITT n=1164  1223 randomised and received allocated intervention; 63 lost to follow-up, 2 deaths within 6 weeks  **Vicryl:** mITT n=1273  1323 randomised and received allocated intervention; 58 lost to follow-up, 1 death within 6 weeks  Paper states that ITT analysis was conducted but patients who died or were lost to follow-up do not appear to have been included in the analyses. | Intervention: Triclosan-coated sutures (Vicryl Plus)  Comparator(s): Uncoated sutures (Vicryl) | Primary outcome: superficial SSI based on Health Protection Agency definitions (which originated from CDC 1992 criteria) at 30 days’ post-operative follow-up  Secondary outcomes:   - Deep incisional infection at 30 days (no implant) or 12 months (implant in place) postoperatively - 30- and 90-day mortality - Length of hospital stay - Clostridium difficile infections - Complications recorded during the course of the trial - Critical care admission - Specific postoperative complications (deep vein thrombosis and pulmonary embolism at 60 days; stroke, transient ischaemic attack, gastrointestinal bleed, urinary retention, urinary tract infection, myocardial infarction, and pneumonia, all at 30 days) - Readmission   From ISRCTN record:   - Surgeon grade- consultant orthopaedic surgeon, Specialist trainee or core training doctor - Cost analysis |
| Sukeik 2019, UK  Other identifiers:  ISRCTN 21430045  Source:  Primary: ^[56]^  Secondary: ^[57]^ | Single-centre. double-blind RCT  Double-blind study where patients, surgeons and outcome assessors all blinded to treatment allocation. The sutures were indistinguishable after removal of the package labelling. Use of sealed envelopes for cases and controls with assignment of letters and codes. | Adult patients (≥ 18 years old) who were undergoing primary total hip or knee arthroplasty under the care of one surgical team at the institute (Department of Trauma and Orthopaedics, University College London Hospital) | Adult patients undergoing primary total hip or knee arthroplasties  One university hospital in the UK  **Vicryl Plus**: ITT n=81  81randomized; 6 did not attend 6-week follow-up  **Vicryl:** ITT n=69  69 randomized; 5 did not attend 6-week follow-up  Trial terminated early due to end of contract with Ethicon and hence the sutures were no longer available (planned inclusion of 420 participants; inclusion of 150) | Intervention: Triclosan-coated sutures (Vicryl Plus)  Comparator(s): Uncoated sutures (Vicryl) | Primary outcome: ASEPSIS wound scoring system to evaluate wound healing for the first 6 weeks post-operatively.  Secondary outcomes:   - Time for wound closure (minutes) - Length of operation (minutes) - Length of hospital stay in days - Pain assessment (VAS scores) at 1, 3 and 5 days post-operatively - Post-operative complications |
| Sundaram 2020a, USA  Other identifiers: NCT03285529  Source:  Primary: ^[58]^  Secondary: ^[59]^ | Single-centre, single-blind RCT  Single-blind.  A random envelope, which dictated the suture to be used, was drawn at the start of each arthroplasty Research personnel revealed the treatment assigned to the participating surgeon, but patients remained unaware. Independent research personnel conducted a blinded outcome assessment | All patients undergoing a primary total knee arthroplasty  From NCT record:   - Males and female aged 18 to 80 years at time of providing informed consent - Able to understand and voluntarily sign an informed consent form prior to any study-related assessments or procedures - Able to adhere to the study visit schedule and other protocol requirements - Fluent in local language (can speak and understand) - If female, is non-pregnant (negative pregnancy test results at baseline and randomisation visit) and non-lactating - End-stage osteoarthritis patients planning to undergo primary total knee arthroplasty   BMI <40 kg/m2 | Adult patients aged 18 to 18 years who were undergoing a primary total knee arthroplasty for end-stage osteoarthritis  One hospital in the USA  **Stratafix Symmetric PDS Plus**: ITT n=30  30 randomised and received allocated intervention; no withdrawals or loss to follow-up  **Vicryl**: ITT n=30  30 randomised and received allocated intervention; no withdrawals or loss to follow-up | Intervention: Triclosan-coated barbed sutures (Stratafix Symmetric PDS Plus)  Comparator(s): Uncoated sutures (Vicryl) | Primary and secondary outcomes were not explicitly reported. Study focused on duration of arthrotomy closure, the rate of suture utilisation, wound complications, readmission and reoperation  From NCT record:  Primary outcome was time to complete skin closure per protocol and operative time   - Secondary outcome was the number of participants with wound complications (superficial wound infection, deep wound infection, periprosthetic joint infection, wound hematoma, and wound dehiscence); costs |
| Sundaram 2020b, USA  Other identifiers: NCT03285555  Source:  Primary: ^[60]^  Secondary: ^[61]^ | Single-centre, single-blind RCT  Single-blind. Patients and outcome assessors were blinded to the treatment allocated.  A random envelope, which dictated the suture to be used, was drawn at the start of each operation thus blinding the patients to the suture type used | - Patients undergoing primary total hip arthroplasty for osteoarthritis   From NCT record:   - Males and female aged between 18 to 80 years at time of providing informed consent - Able to understand and voluntarily sign an informed consent form prior to any study-related assessments or procedures - Able to adhere to the study visit schedule and other protocol requirements - Fluent in local language (can speak and understand) - If female, is non-pregnant (negative pregnancy test results at baseline and randomisation visit) and non-lactating - End-stage osteoarthritis patients planning to undergo primary total hip arthroplasty - BMI 40 kg/m2 | Adult patients aged 18 to 18 years who were undergoing primary total hip arthroplasty for end-stage osteoarthritis  One hospital in the USA  **Stratafix Symmetric PDS Plus**: ITT n=30  30 randomised and received allocated intervention; no withdrawals or loss to follow-up  **Vicryl**: ITT n=30  30 randomised and received allocated intervention; no withdrawals or loss to follow-up | Intervention: Triclosan-coated barbed sutures (Stratafix Symmetric PDS Plus)  Comparator(s): Uncoated sutures (Vicryl) | Primary and secondary outcomes were not explicitly reported. Study focused on arthrotomy closure duration, wound complications, readmission and reoperation  From NCT record:  Primary outcome was time to complete skin closure per protocol and operative time  Secondary outcome was the number of participants with wound complications (superficial wound infection, deep wound infection, periprosthetic joint infection, wound hematoma, and wound dehiscence); costs |
| Tabrizi 2019, Iran  Other identifiers: NCT03659344  Source:  Primary: ^[62]^  Secondary: ^[63]^ | Single-blind, randomised clinical trial conducted across two sites in Iran  Patients were blinded to the type of suture used | Patients undergoing dental surgery who received three implants in the posterior mandible | Patients undergoing dental surgery who received three implants in the posterior mandible  One university hospital in Tehran and one private medical clinic in Isfahan  **Vicryl Plus**: 160  **Vicryl:** 160  No loss to follow-up or withdrawals are reported | Intervention: Triclosan-coated sutures (Vicryl Plus)  Comparator(s): Uncoated sutures (Vicryl) | Primary and secondary outcomes not explicitly specified.  The aim of this study was to compare the incidence of surgical site infection following the use of Vicryl and Vicryl Plus Sutures in dental implant surgeries. |
| Thimour-Bergström 2013, Sweden  Other identifiers:  NCT01212315  Source:  Primary: ^[8, 9]^  Secondary: ^[64-66]^ | Single-centre, double-blind RCT  Surgeon, patients and outcome assessors were unaware of treatment assignment.  Research nurses  who were not involved in the patients’ follow-up revealed the assigned treatment, and delivered the assigned package to the operation room, where sutures were removed from their packages, and placed in the operating room without any identification marks prior to the surgeon’ arrival. Both the coated and non-coated sutures looked  identical  NCT record also states masking of care provider and investigator | Patients with scheduled coronary artery bypass graft (CABG), CABG + aortic valve replacement (AVR)  or CABG + mitral valve repair or replacement at Sahlgrenska  University Hospital with intended use of a saphenous vein graft  From NCT record: age 18 to 85 years eligible for study | Adult patients undergoing elective saphenous vein harvesting and sternotomy during cardiac surgery  One university hospital in Sweden  **Open vein harvesting:**  **Vicryl Plus + Monocryl Plus:** analysed (‘as-treated’) n=184  Randomised 193; received allocated treatment 187; loss to follow-up 3 (1 death, 2 declined follow-up)  **Vicryl + Monocryl**: analysed (‘as-treated’) n=190  Randomised 199; received allocated treatment 192; loss to follow-up 2 (1 death, 1 declined follow-up)  **Sternotomy:**  **Vicryl Plus + Monocryl Plus:** analysed (‘as-treated’) n=179  Randomised 193; received allocated treatment 191; loss to follow-up 12 (9 re-operations, 1 death, 2 not possible to reach)  **Vicryl + Monocryl**: analysed (‘as-treated’) n=178  Randomised 200; received allocated treatment 195; loss to follow-up 17 (13 re-operations, deaths, 2 not possible to reach) | Intervention: Triclosan-coated sutures (Vicryl Plus and Monocryl Plus)  Comparator(s): Uncoated sutures (Vicryl and Monocryl) | **Open vein harvesting:**  Primary endpoint: SSI in the vein-harvesting leg, according to CDC definition (1992), within 60 days after surgery  Secondary endpoints:   - Culture-proven SSI according to CDC definition, within 60 days after surgery - Antibiotic-treated SSI according to CDC definition within 60 days after surgery - ASEPSIS score at Days 30 and 60 postoperatively - Non-infectious leg-wound dehiscence within 60 days after surgery   **Secondary analysis of sternotomy outcomes:**  Primary endpoint: any sternal wound infection (either superficial or deep) as defined by the CDC within 60 days after the primary operation.  Other outcomes measured:   - Deep and superficial sternal wound infection according to the CDC’s definition within 60 days after surgery - Antibiotic-treated sternal SSI - Culture-proven sternal SSI - ASEPSIS score at days 4, 30 and 60 postoperatively |
| Turtiainen 2012, Finland  Source: ^[67]^ | Prospective, randomised, multicentre, double-blinded trial in five hospitals in Finland  Only the nurses in the operating theatre knew to which group each patient had been randomised. Neither the vascular surgeons, the nurses in the surgical ward, nor the patients knew to which group a patient had been randomised. | The study group comprised adult patients who underwent non-emergency lower-limb arterial surgery. | Three tertiary referral hospitals and two secondary referral hospitals in Finland  **Vicryl Plus and Monocryl Plus:** 139  6 patients died but all were included in the final analysis. 0 lost to follow up.  **Vicryl and Monocryl:** 137  4 patients died but all were included in the final analysis. 0 lost to follow up. | Intervention: Triclosan-coated sutures (Vicryl Plus and Monocryl Plus)  Comparator(s): Uncoated sutures (Vicryl and Monocryl) | Primary outcome: Occurrence of surgical wound infection  No secondary outcomes reported |
| Williams 2011, UK  Source: ^[68]^ | Single-centre double-blind RCT  The surgeon, patient, and the assessor at follow-up were blinded to which type of suture had been used | Female patients older than 18 years undergoing skin closure after breast cancer surgery | Adult women undergoing skin closure after breast cancer surgery  One hospital in UK  **Vicryl Plus or Monocryl Plus:** n = 75; analysed n = 66 at 6 weeks’ follow up  75 randomised; 9 withdrawn from study by 6 weeks. Patient request = 2; lost to follow up = 1; need for further surgery = 6  **Vicryl or Monocryl: ITT** n = 75; analysed n = 61 at 6 weeks’ follow up  75 randomised; 14 withdrawn from study by 6 weeks. Patient request = 1; lost to follow up = 3; need for further surgery = 10 | Intervention: Triclosan-coated sutures (Vicyl Plus or Monocryl Plus)  Comparator(s): Uncoated sutures (Vicryl or Monocryl) | Primary and secondary outcomes not explicitly specified  The study measured the SSI rate, based on CDC definitions, as well as ASEPSIS and Southampton wound scores |
| Zhang 2011, China  Other identifiers:  NCT00768222  Source:  Primary: ^[69]^  Secondary: ^[70]^ | Post-market, multi-centre, randomised, open-label pilot study  Open-label.  Treatment assignment was revealed to the patients and surgeon at the time of wound closure. Blinded assessment of primary outcome by a central assessor, and non-blinded assessment of secondary outcomes | - Women aged ≥18 years - Scheduled for clean modified radical mastectomy - Signed hospital approved informed consent - Class I (Clean) surgical wound (CDC SSI Surgical Wound Classification) | Women aged ≥18 years undergoing modified radical mastectomy for breast cancer  6 hospitals in China  **Vicryl Plus**: ITT n=51, PP n=46  51 randomised and received allocated intervention; 5 excluded from analysis (1 lost to follow-up, 1 discontinued intervention, 1 consent withdrawal, 2 protocol violations)  **Chinese silk**: ITT n=50, PP n=43  51 randomised and received allocated intervention; 7 excluded from analysis (1 lost to follow-up, 0 discontinued intervention, 3consent withdrawals, 3 protocol violations) | Intervention: Triclosan-coated sutures (Vicryl Plus)  Comparator(s): Uncoated sutures (Chinese silk) | Primary outcome: Cosmetic outcome, by VAS scoring of blinded surgical site wound photographs at 30 days  Secondary outcomes:   - Photograph score of cosmetic outcome at day 12 - Modified Hollander Cosmetic Scale score at days 12 and 30, as assessed by non-blinded nvestigator - Incidence of SSIs, based on ASEPSIS wound scores and CDC criteria   From NCT record:   - Mean SSI score on modified ASEPSIS scale at days 3, 5, 7, 12, 30, 90 |

### Supplementary_Table_5: Summary of review included study characteristics

| **Study**  *Author, year, location* | **Trial setting**  *Setting of surgery and number of sites* | **Details of intervention** | **Details of control** | **No. of participants randomised** | **Surgery type** | **Wound class**  *Clean / clean-contaminated / contaminated / dirty* | **Definition of SSI**  *CDC / other (if other, define)* | **Maximum duration of trial follow-up** |
| --- | --- | --- | --- | --- | --- | --- | --- | --- |
| Arslan 2018, Turkey  Source: ^[20]^ | Unspecified number of hospital surgical department | Triclosan-coated sutures (PDS Plus + Vicryl Plus)  (Wound closure following cyst excision:  1/0 PDS Plus for retention, 3/0 Vicryl Plus for subcutaneous tissue, and 3/0 PDS Plus for skin closure) | Uncoated sutures (Prolene + Vicryl)  (Wound closure following cyst excision:  1/0 Prolene for retention, 3/0 Vicryl for subcutaneous tissue, and 3/0 Prolene for skin closure) | Total: 177  **PDS Plus + Vicryl Plus**: 92 (86 analysed)  **Prolene + Vicryl**: 95 (91 analysed) | Wide excision and primary closure for pilonidal disease | NR | CDC guidelines (2017) | 30 days post-surgery |
| Baracs 2011, Hungary  Other identifiers: NCT01123616  Source:  Primary: ^[21]^  Secondary: ^[22]^ | 7 Hungarian surgical centres (3 university clinics and 4 high-volume hospitals) | Triclosan-coated sutures (PDS Plus)  (Abdominal fascia closure using triclosan-coated PDS Plus Sutures  Optional separate peritoneal closure and subcutaneous 2-0 sutures, depending on surgeon preference) | Uncoated sutures (PDS II)  (Abdominal fascia closure using uncoated PDS II sutures  Optional separate peritoneal closure and subcutaneous 2-0 sutures, depending on surgeon preference) | Total: 385  **PDS Plus:** 188  **PDS II:** 197 | Open colorectal surgery involving an enterotomy | NR but authors considered open and rectal procedures to be classified as clean-contaminated. | NR | One year |
| Diener 2014, Germany  Other identifiers: PROUD, DRKS00000390  Source:  Primary: ^[23]^  Secondary: ^[23-27]^ | Surgical departments of 24 secondary and tertiary care centres | Triclosan-coated sutures (PDS Plus)  (Abdominal fascia closure after midline laparotomy using triclosan-coated PDS Plus PDP9262T sutures (needle: CTX 48 mm 1/2 circle)) | Uncoated sutures (PDS II)  (Abdominal fascia closure using non-coated PDS II Z1950G sutures (needle: CTX 48 mm 1/2 circle)) | Total: 1224  **PDS Plus:** 607  **PDS II:** 617 | Abdominal laparotomy | **PDS Plus:** clean 144 (24.5%); clean-contaminated 430 (73.3%); contaminated 11 (1.9%); dirty 2 (0.3%)  **PDS II:** clean 138 (23.1%); clean-contaminated 450 (75.3%); contaminated 9 (1.5%); dirty 1 (0.2%) | Modified version of CDC 1992 criteria | 30 days post-surgery |
| Ford 2005, USA  Source: ^[28]^ | NR explicitly but authors’ affiliations suggest one hospital paediatric surgical department | Triclosan-coated sutures (Vicryl Plus)  (No surgical details relating to skin/tissue closure) | Uncoated  sutures (Vicryl)  (No surgical details relating to skin/tissue closure) | Total: 151  **Vicryl Plus**: 100*  **Vicryl**: 51* | General surgical procedures (no further details) | NR but study inclusion criteria stipulated clean or clean-contaminated surgical procedures | Other: observed redness >3–5mm from the wound margins, edema, purulent discharge, pain, and increased skin temperature were considered evidence of an infection; a confirmatory culture was not required | 80 (±5) days post-surgery |
| Galal 2011, Egypt  Source: ^[29]^ | Unspecified number of centres  This article reported the results from one university hospital surgical department | Triclosan-coated sutures (Vicryl Plus)  (Vicryl Plus sutures used in all steps, except for laparotomy closure and vascular structure) | Uncoated  sutures (Vicryl)  (Vicryl Plus sutures used in all steps, except for laparotomy closure and vascular structure) | Total: 450  **Vicryl Plus**: 230  **Vicryl**: 220 | Any type of surgery | Traditional wound classification  **Vicryl Plus**:  Clean: 117 (50.9%*)  Clean-contaminated: 71 (30.9%*)  Contaminated: 35 (15.2%*)  Infected/dirty: 0 (0)  **Vicryl**:  Clean: 119  (54.1%*)  Clean-contaminated: 72 (32.7%*)  Contaminated: 36 (16.4%*)  Infected/dirty: 0 (0) | Modified CDC (1992) criteria | 30 days post-discharge  (1 year for prosthetic surgery) |
| Ichida 2018, Japan  Other identifiers:  UMIN000013054  Source:  Primary: ^[30]^  Secondary: ^[31]^ | One surgical department in a medical university | Triclosan-coated sutures (Vicryl Plus)  (Closure of abdominal fascia and peritoneum using Vicryl Plus sutures) | Uncoated sutures (Vicryl)  (Closure of abdominal fascia and peritoneum using Vicryl sutures) | Total: 1023  **Vicryl Plus**: 512 (analysed: 508)  **Vicryl**: 511 (analysed: 505) | Gastroenterologic surgery | **Vicryl Plus**:  Clean: 6 (1.2%)  Clean-contaminated: 495 (97.4%)  Contaminated/Dirty: 7 (1.4%)  **Vicryl**:  Clean: 3 (0.6%) Clean-contaminated: 495 (98.0%)  Contaminated/Dirty: 7 (1.4%) | CDC criteria | Up to 30 days post-discharge |
| Isik 2012, Turkey  Source: ^[32]^ | One cardiovascular surgical department in a private hospital | Triclosan-coated sutures (Vicryl Plus)  (Closure of leg and sternal wound sites using Vicryl Plus sutures) | Uncoated sutures (Vicryl)  (Closure of leg and sternal wound sites using Vicryl sutures) | Total: 510  **Vicryl Plus:** 170  **Vicryl**: 340 | Various cardiac surgical procedures | NR | CDC criteria | 1 month post-surgery |
| Justinger 2013, Germany  Other identifiers:  NCT00998907  Source:  Primary: ^[33]^  Secondary: ^[34]^ | One surgical department in a university hospital | Triclosan-coated sutures (PDS Plus)  (Closure of abdominal fascia using 2-0 PDS Plus loop sutures) | Uncoated sutures (PDS II)  Closure of abdominal fascia 2-0 PDS II loop sutures) | Total: Randomised NR (967 operated on per protocol)  **PDS Plus**: NR (559 operated on per protocol) (analysed: 485)  **PDS II**: NR (408 operated on per protocol) (analysed: 371) | Scheduled laparotomy with abdominal wound closure following a standard clinical pathway | **PDS Plus**:  Clean: 286 (59%)  Clean-contaminated: 162 (33.4%)  Contaminated: 37 (7.6%)  Septic: 0 (0)  **PDS II**:  Clean:245 (66%)  Clean-contaminated: 97 (26.1%)  Contaminated: 25 (6.7%)  Septic: 4 (1.1) | CDC criteria | 2 weeks post-discharge |
| Karip 2016, Turkey  Source: ^[35]^ | General surgery clinics at one training and research hospital | Triclosan-coated sutures (Monocryl Plus)  (Incision closure using Monocryl Plus suture, ensuring that the suture line was not on the midline) | Uncoated sutures (Monocryl)  (Incision closure using Monocryl suture, ensuring that the suture line was not on the midline) | Revised trial, total: 106  **Monocryl Plus:** 54  **Monocryl**: 52 | Sinus excision followed by Karydakis flap repair | NR | Other: surgeon-assessed signs of infection (rash, fever, or purulent discharge) on physical examination | 6 months |
| Lin 2018, Taiwan  Other identifiers:  NCT02533492  Source:  Primary: ^[36]^  Secondary: ^[37]^ | One hospital | Triclosan-coated sutures (Vicryl Plus)  (3-layer closure using Vicryl Plus: arthrotomy, fascial layer, and subcutaneous wound) | Uncoated sutures (Vicryl)  (3-layer closure using Vicryl: arthrotomy, fascial layer, and subcutaneous wound) | Total: 102  **Vicryl Plus**: 51  **Vicryl**: 51 | Unilateral total knee arthroplasty using standard medial parapatellar technique | Clean: 102 (100%) | Other: Presence of SSI based on wound condition (surface temperature, digital photo, image analysis) | Within 3 months post-surgery |
| Mattavelli 2015, Italy  Other identifiers:  NCT01869257  Source:  Primary: ^[38]^  Secondary: ^[39]^ | Four university referral hospitals | Triclosan-coated sutures (Vicryl Plus + PDS Plus)  (Separate layer technique: closure of peritoneum with Vicryl Plus suture, then closure of abdominal fascia with PDS Plus suture; optional subcutaneous closure of subcutaneous tissue layer using 3/0 Vicryl Plus suture) | Uncoated sutures (Vicryl + PDS II)  (Separate layer technique: closure of peritoneum with Vicryl suture, then closure of abdominal fascia with PDS suture; optional subcutaneous closure of subcutaneous tissue layer using 3/0 Vicryl suture) | Total: 300  **Vicryl Plus + PDS Plus:** 150  **Vicryl + PDS II:** 150 | Elective colorectal resection | NR but study inclusion criteria stipulated colorectal resection with a clean-contaminated field | CDC 1999 criteria | 30 days post-discharge |
| Mingmalairak 2009, Thailand  Source: ^[40]^ | One univeraity hospital in Thailand | Vicryl Plus | Vicryl | 100 (this paper is a report of the first 100 patients randomised and treated) | Appendicitis surgery: types of appendicitis are were follows: | Wound class NR but study reports “degree of contamination”  **Vicryl Plus; n(%)**:  Mild 43 (86)  Moderate 4 (8)  Severe 3 (6)  **Vicryl; n(%):**  Mild 40 (80)  Moderate 6 (12)  Severe 4 (8) | “As defined by a surgeon”; further details NR | Paper states 1 year post-surgery, but also states that the patients were studied between August 2006 and March 2007, which is 9 months |
| Nakamura 2013, Japan  Other identifiers: UMIN00003322  Source:  Primary: ^[41]^  Secondary: ^[42]^ | One surgical department in a hospital | Triclosan-coated sutures (Vicryl Plus)  (Abdominal closure after laparotomy using Vicryl Plus suture) | Uncoated sutures (Vicryl)  (Abdominal closure after laparotomy using Vicryl suture) | Overall: 410  **Vicryl Plus**: 206  **Vicryl:** 204 | Elective colorectal surgery | **Vicryl Plus**:  Clean: 0 (0)  Clean-contaminated: 205 (99.5%*)  Contaminated: 1 (0.5%*)  Dirty: 0 (0)  **Vicryl:**  Clean: 0 (0)  Clean-contaminated: 203 (99.5%*)  Contaminated: 1 (0.5%*)  Dirty: 0 (0) | CDC 1999 guidelines | 30 days post-discharge |
| Olmez 2019, Turkey  Source: ^[43]^ | Sites NR; Turkey | PDS Plus | PDS II | Total: 900  **PDS Plus**: Enrolled n = 450. Analysed n = 445 (2 dropped from follow up, reason NR, 3 deaths)  **PDS II**: Enrolled n = 450. Analysed n = 445 (4 dropped from follow up, reason NR, 1 death) | Elective or urgent gastrointestinal surgery | Calculated from Table 5 of publication  **PDS Plus; n (%)**:  Clean 18 (4.0)  Clean-contaminated 396 (89.0)  Contaminated 30 (6.7)  Dirty 1 (0.2)  **PDS II; n(%):**  Clean 66 (14.8)  Clean-contaminated 255 (57.3)  Contaminated 122 (27.4)  Dirty 2 (0.4) | Unclear, although the authors reference NICE Guidance CG74 (2014) | 30 days post-surgery |
| Rasic 2011, Croatia  Source: ^[44]^ | One surgical department in a university hospital | Triclosan-coated sutures (Vicryl Plus  (Wound closure with 0 Vicryl Plus sutures using a continuous single-layer mass technique (peritoneum, muscle and fascia)  Skin was closed with polyamide: Ethicon 2-0) | Non Triclosan-coated sutures (Vicryl)  (Wound closure with 0 Vicryl sutures using a continuous single-layer mass technique (peritoneum, muscle and fascia)  Skin was closed with polyamide 2-0) | Total: 184  **Vicryl Plus:** 91  **Vicryl:** 93 | Elective colorectal carcinoma surgery through a midline incision | NR | NR | NR  “Hospitalisation period” (p 440 of paper) |
| Renko 2017, Finland  Other identifiers:  NCT01220700  Source:  Primary: ^[45]^  Secondary: ^[46]^ | Paediatric surgery and orthopaedics ward in a university hospital (serving as tertiary paediatric hospital)  Optional further follow up carried out at local health centre or private practice | Triclosan-coated sutures (Vicryl Plus, Monocryl Plus, or PDS Plus)  Surgeons could use other suture materials in addition to the study sutures during surgery if the study sutures were unsuitable for the procedure | Non-coated sutures (Vicryl, Monocryl, or PDS)  Surgeons could use other suture materials in addition to the study sutures during surgery if the study sutures were unsuitable for the procedure | Total: 1633  **Triclosan-coated sutures:** 814  (778 included in the mITT analysis)  **Non-coated sutures**: 819  (779 included in the mITT analysis) | NR  Target organs for surgery were: nervous system, chest wall and lungs, abdominal wall (including hernias), intra-abdominal (including gallbladder, intestines, and spleen) urinary system and genitals, musculoskeletal system, skin or other subcutaneous tissue, other | **Triclosan-coated sutures** (n=778):  Clean: 699 (99%);  Clean-contaminated: 26 (3%);  Contaminated: 0 (0);  Dirty or infected: 0 (0);  Missing data: 53 (7%)  **Non-coated sutures** (n=779):  Clean: 695 (89%);  Clean-contaminated: 27 (3%);  Contaminated: 1 (<1%);  Dirty or infected: 0 (0);  Missing data: 56 (7%) | CDC 1992 criteria | 30 days post- surgery |
| Rozzelle 2008, USA  Source: ^[47]^ | One hospital in New York state, USA | Vicryl Plus | Vicryl | Patients receiving new shunts following successful treatment of a shunt infection and patients undergoing revision 6 months after randomisation were re-randomised.  Total N operations: 84  No patients were lost to follow-up during the study period.  **Vicryl plus:** Randomised operations: n = 46  **Vicryl:** Randomised operations: n =38 | Implantation of cerebrospinal fluid (CSF) shunting device | NR | NR | This was intended to be 6 months post-surgery, but only results up to the second interim analysis (14 weeks) were presented |
| Ruiz-Tovar 2020, Spain  Other identifiers: NCT03763279  Source:  Primary: ^[48]^  Secondary:  ^[49]^ | Surgical departments of hospitals in Spain. Number NR but authors’ affiliations suggest up to 4. | Two intervention arms  Triclosan-coated barbed suture calibre 1, 48-mm sutures with cylindric needle (Stratafix Symmetric PDS Plus)  Triclosan-coated non-barbed suture calibre 1, 48-mm sutures with cylindric needle (PDS Plus Loop) | Uncoated sutures (PDS Loop)  (Abdominal fascia closure using uncoated PDS Loop sutures (standard calibre 1, 48-mm cylindric needle)) | Total: 150  **Stratafix Symmetric Plus**: 50  **PDS Plus Loop**: 50  **PDS Loop:** 50 | Emergency surgery by laparotomy and midline approach | NR but inclusion criteria specified contaminated and dirty surgery | CDC 1992 definition | 30 days post-surgery |
| Ruiz-Tovar, 2015, Spain  Source: ^[50]^ | Two university hospitals in Spain | Triclosan-coated polyglactin 910 antimicrobial loop suture size number 2 (brand NR) | Uncoated polyglactin 910 antimicrobial loop suture size number 2 (brand NR) | Total: 110  **Triclosan-coated sutures:** 55  **Uncoated sutures:** 55 | Abdominal closure following intraoperative diagnosis of faecal peritonitis secondary to acute diverticulitis perforation, neoplastic tumor perforation, or colorectal anastomotic leak of previous elective colorectal resection.; all patients underwent a Hartmann procedure | Dirty | CDC 1992 definition | 60 days post-surgery |
| Santos 2019, Brazil  Source: ^[51]^ | One teaching hospital in Brazil | Vicryl Plus | Vicryl | Total: 583  **Vicryl Plus**: 289  **Vicryl:** 257 | Saphenectomy during coronary artery bypass graft (CABG), with and without cardiopulmonary bypass: (CPB) | NR | NR | 30 days post-surgery |
| Seim 2012, Norway  Source: ^[52]^ | One cardiothoracic surgery department in a university hospital | Triclosan-coated sutures (Vicryl Plus)  (Leg wound closed using Vicryl Plus) | Uncoated sutures (Vicryl)  (Leg wound closed using Vicryl) | Total: 328  **Vicryl Plus**: 164 (160 analysed)  **Vicryl**: 164 (163 analysed) | Coronary artery bypass graft surgery with saphenous vein harvesting | NR | Other: SSI diagnosis based on positive bacterial culture and clinical judgement | 4 weeks post-surgery |
| Soomro 2017, Pakistan  Source: ^[53]^ | One breast unit at a national hospital | Triclosan-coated sutures (brand NR) | Uncoated sutures (brand NR) | **Total:** 378  **Triclosan-coated sutures:**189  **Plain sutures:** 189 | Minor clean breast surgeries in benign breast diseases | Clean | CDC guidelines (version NR) | 30 days post-surgery |
| Sprowson 2018, UK  Other identifiers:  ISRCTN17807356  Source:  Primary: ^[54]^  Secondary: ^[55]^ | Three acute teaching hospitals that were elective centres | Triclosan-coated sutures (Vicryl Plus)  (Closure of deep fascia to subcutaneous layer, dependent on surgeon preference, using Vicryl Plus suture) | Uncoated sutures (Vicryl)  (Closure of deep fascia to subcutaneous layer, dependent on surgeon preference, using Vicryl suture) | Total: 2546  **Vicryl Plus:** 1223  **Vicryl:** 1323 | Primary total hip or knee arthroplasty | NR | Health Protection Agency defiinitions | 12 months |
| Sukeik 2019, UK  Other identifiers:  ISRCTN 21430045  Source:  Primary: ^[56]^  Secondary: ^[57]^ | One Trauma and orthopaedic department in a university hospital | Triclosan-coated sutures (Vicryl Plus) to close the deep layers of the wound  (1 interrupted Vicryl Plus for closure of medial parapatellar incisions (knee) and fascia lata (hip); and  2-0 Vicryl Plus for closure of subcutaneous tissues (hip and knee))  Skin clips used for the outside skin closure | Non-coated sutures (Vicryl) to close the deep layers of the wound  (1 interrupted Vicryl for closure of medial parapatellar incisions (knee) and fascia lata (hip); and  2-0 Vicryl Plus for closure of subcutaneous tissues (hip and knee))  Skin clips used for the outside skin closure | Total: 150  **Vicryl Plus:** 81  **Vicryl:** 69 | Unilateral knee arthroplasty: medial parapatellar approach (+ cement)  Unilateral hip arthroplasty: posterior approach (uncemented prostheses) | NR | NR  Superficial SSIs defined as those resolved with oral antibiotics only  Deep SSIs defined as those not controlled with oral antibiotics or required washout/debridement or revision surgery. | 6-weeks post-surgery |
| Sundaram 2020a, USA  Other identifiers: NCT03285529  Source:  Primary: ^[58]^  Secondary: ^[59]^ | One hospital | Triclosan-coated barbed sutures (Stratafix Symmetric PDS Plus)  (3-layer closure: #1 Stratafix PDS Plus suture with symmetric barbs for closure of the capsule; then 2-0 Vicryl suture for closure of subcuticularous layer and finally 3-0 Monocryl suture for subcutaneous layer, followed by adhesive strips) | Uncoated sutures (Vicryl)  (3-layer closure: #1 Vicryl suture for closure of arthrototomy (deep layer) ; then 2-0 Vicryl suture for closure of intermediate layer and finally 3-0 Monocryl suture for subcutaneous layer, followed by adhesive strips) | Total**:** 60  **Stratafix Symmetric PDS Plus:** 30  **Vicryl:** 30 | Total knee arthroplasty using medial para-patella approach | NR | Other: definitions were adapted from consensus criteria from the Knee Society (2013) | 90 days post-surgery |
| Sundaram 2020b, USA  Other identifiers: NCT03285555  Source:  Primary: ^[60]^  Secondary: ^[61]^ | One orthopaedic surgery department in a hospital | Triclosan-coated barbed sutures (Stratafix Symmetric PDS Plus)  (4-layer closure: unidirectional #1 Stratafix PDS Plus suture with symmetric barbs for closure of the arthrotomy; then 2-0 Vicryl suture for closure of subucatenous layer and 3-0 Monocryl suture for subcuticular layer, followed by adhesive strips) | Uncoated sutures (Vicryl)  (4-layer closure: #1 Vicryl suture for closure of the arthrotomy (deep layer); then 2-0 Vicryl suture for closure of subucatenous layer and 3-0 Monocryl suture for subcuticular layer, followed by adhesive strips) | Total**:** 60  **Stratafix Symmetric PDS Plus:** 30  **Vicryl:** 30 | Posterior approach total hip arthroplasty with repair of posterior capsule and short external rotator | NR | Other: definitions developed by the Hip Society (2016) | 90 days post-surgery |
| Tabrizi 2019, Iran  Other identifiers: NCT03659344  Source:  Primary: ^[62]^  Secondary: ^[63]^ | Two sites; one university hospital and one private medical clinic | Triclosan-coated sutures (Vicryl Plus) | Uncoated sutures (Vicryl) | **Total:** 320  **Vicryl Plus:** 160  **Vicryl:** 160 | Dental implant surgery to place three dental implants in the posterior mandible | NR | Authors’ definition: “local erythematous changes in the mucosa around the dental implant with a purulent discharge, or localized abscess formation at the surgical site, and/or increasing pain and swelling in the operated area” | 28 days post-surgery |
| Thimour-Bergström 2013, Sweden  Other identifiers:  NCT01212315  Source:  Primary: ^[8, 9]^  Secondary: ^[64-66]^ | One surgical department in a university hospital | Triclosan-coated sutures (Vicryl Plus, Monocryl Plus)  (Saphenous vein skin closure: subcutaneously with 3.0 Vicryl Plus suture and intracutaneously with 4.0 Monocryl Plus suture)  (Fascia and subcutaneous tissue closed using 2.0 Vicryl Plus suture and intracutaneously using 4.0 Monocryl Plus suture)  The same kind of sutures was used to close the wound on both  the sternum and the leg | Non-coated sutures (Vicryl, Monocryl)  (Saphenous vein skin closure: subcutaneously with 3.0 Vicryl suture and intracutaneously with 4.0 Monocryl suture)  (Fascia and subcutaneous tissue closed using 2.0 Vicryl suture and intracutaneously using 4.0 Monocryl suture)  The same kind of sutures was used to close the wound on both  the sternum and the leg | Total: 392  ***Open vein harvesting:***  **Vicryl Plus + Monocryl Plus:** 193 (184 analysed)  **Vicryl + Monocryl**: 199 (190 analysed)  ***Sternotomy:***  **Vicryl Plus + Monocryl Plus:** 193 (179 analysed)  **Vicryl + Monocryl**: 200 (178 analysed) | CABG or CABG plus valve surgery using a saphenous vein graft and sternotomy | NR | CDC 1992 definition | 60 days post-surgery |
| Turtiainen 2012, Finland  Source: ^[67]^ | Three tertiary referral hospitals and two secondary referral hospitals in Finland | Triclosan-coated sutures (Vicryl Plus and Monocryl Plus) | Uncoated sutures (Vicryl and Monocryl) | **Total:** 276  **Vicryl Plus and Monocryl Plus**: 139  **Vicryl and Monocryl:** 137 | Non-emergency lower-limb arterial surgery | NR | CDC guidelines (1992) | Unclear; All patients were followed up for at least one month post-surgery, but some patients were followed up for at least 125 days post-surgery for safety outcomes. Definition of SSI is “within 30 days post-surgery”. |
| Williams 2011, UK  Source: ^[68]^ | Two breast surgeons at the Cardiff and Vale NHS Trust (single centre) | Vicryl Plus and Monocryl Plus | Vicryl and Monocryl | Total: 150  **Vicryl Plus and Monocryl Plus**: 75  **Vicryl and Monocryl:** 75 | Breast surgery | **Vicryl Plus and Monocryl Plus**: Clean 75 (100%)  **Vicryl and Monocryl:** Clean 75 (100%) | CDC 1999 guidelines | 6 weeks post surgery |
| Zhang 2011, China  Other identifiers:  NCT00768222  Source:  Primary: ^[69]^  Secondary: ^[70]^ | 6 Chinese first tier hospitals | Triclosan-coated sutures (Vicryl Plus)  (Intradermal, subcuticular skin closure using Vicryl Plus sutures in accordance with unified standard of care) | Uncoated sutures (Chinese silk)  (Simple interrupted transdermal skin closure using Chinese silk sutures in accordance with unified standard of care) | Total: 101  **Vicryl Plus:** 51  **Chinese Silk**: 50 | Clean (Class I) modified radical mastectomy | Clean: All patients | CDC criteria and ASEPSIS wound scores | 90 days |

### Supplementary_Table_6: Summary of review included study population details

| **Study**  *Author, year, location* | **Intervention or control, with name**  *One row per arm* | **Age**  *Mean (SD) years* | **Gender**  *N (%) male* | **Pre-operative preparation to facilitate wound healing**  *Bathing with soap: Yes / No / NR*  *Nasal decolonisation: Yes / No / NR*  *Other: Give details* | **Preoperative antibiotics**  *Yes / No; if yes, give n (%)* | **Other post-operative care to facilitate wound healing**  *Wound dressing: Yes / No / NR*  *Sterile saline wash: Yes / No / NR*  *Method of skin closure: Give details*  *Other: Give details* | **N (%) emergency or elective surgery** |
| --- | --- | --- | --- | --- | --- | --- | --- |
| Arslan 2018, Turkey  Source: {Arslan, 2018 #141} | PDS Plus + Vicryl Plus | 25.8 (6.5) | 79 (91.9*) | **Bathing with soap:** NR  **Nasal decolonisation:** NR  **Other:** Hair removal | Yes  Antibiotic prophylaxis: All patients | **Wound dressing:** NR  **Sterile saline wash:** NR  **Method of skin closure:** PSD Plus suture  **Other:** NR | Elective: 86 (100) |
|  | Prolene + Vicryl | 25.5 (5.5) | 76 (83.5*) |  | Yes  Antibiotic prophylaxis: All patients | **Wound dressing:** NR  **Sterile saline wash:** NR  **Method of skin closure:** Prolene suture  **Other:** NR | Elective: 91 (100) |
| Baracs 2011, Hungary  Source:  Primary: {Baracs, 2011 #327}  Secondary: {University of Pecs, 2010 #727} | PDS Plus | 62.6 (SD NR) | 110 (58.5*) | **Bathing with soap:** NR  **Nasal decolonisation:** NR  **Other:** NR | Prophylactic antibiotic (a second-generation cephalosporin and metronidazole 30 minutes before incision) were used in every case | **Wound dressing:** Yes  **Sterile saline wash:** NR  **Method of skin closure:** Suture (Monocryl Plus)  **Other:** Disposable drapes were used | All procedures were elective |
|  | PDS II | 63.5 (SD NR) | 111 (56.3*) |  |  |  |  |
| Diener 2014, Germany  Other identifiers: PROUD, DRKS00000390  Source:  Primary: {Diener, 2014 #273}  Secondary: {Diener, 2014 #273;Heger, 2011 #328;Universitätsklinik Heidelberg, 2010 #1411;Diener, 2014 #577;Fujita, 2014 #628} | PDS Plus | 64.7 (11.8) | 361 (61.5) | **Bathing with soap:** NR  **Nasal decolonisation:** NR  **Other:** Routine scrub and site preparation according to site centres | Yes (according to German national guidelines)  Antibiotic prophylaxis: 578 (98.5) | **Wound dressing:** NR  **Sterile saline wash:** NR  **Method of skin closure:** Staples  **Other:** Postoperative care was provided according to the principles and standards of the participating departments | All procedures were elective |
|  | PDS II | 65.0 (12.1) | 368 (61.5) |  | Yes (according to German national guidelines)  Antibiotic prophylaxis:586 (98.0) |  |  |
| Ford 2005, USA  Source: ^[28]^ | Vicryl Plus | NR (only overall across treatments: mean 9.8, range: 1-18 years) | NR (only overall across treatments: 52% male) | **Bathing with soap:** NR  **Nasal decolonisation:** NR  **Other:** Local protocol for infection control | Yes  IV antibiotics: 65* (65) | **Wound dressing:**  NR  **Sterile saline wash:** NR  **Method of skin closure:** NR  **Other:** Local protocol for infection control | Elective: 98 (100) |
|  | Vicryl |  |  |  | Yes  IV antibiotics: 40* (82) |  | Elective: 49 (100) |
| Galal 2011, Egypt  Source: {Galal, 2011 #337} | Vicryl Plus | Mean NR  Median NR  Age groups covered the range 21-60 years | 148 (64.3%*) | **Bathing with soap:** NR  **Nasal decolonisation:** NR  **Other:** Local protocol for infection control | No | **Wound dressing:**  NR  **Sterile saline wash:** NR  **Method of skin closure:** Monocryl suture  **Other:** Local protocol for infection control | Elective: 230 (100) |
|  | Vicryl |  | 127 (57.7%*) |  | No |  | Elective: 220 (100 |
| Ichida 2018, Japan  Other identifiers:  UMIN000013054  Source:  Primary: {Ichida, 2018 #7}  Secondary: {Department of Surgery Saitama Medical Center Jichi Medical University, 2014 #798} | Vicryl Plus | 67.0 (11.5) | 304 (59.8) | **Bathing with soap:** NR  **Nasal decolonisation:** NR  **Other:** Perioperative care protocols as recommended in CDC guidelines | Yes  Antibacterial prophylaxis: All patients | **Wound dressing:** sterile dressing for ≥48 hours  **Sterile saline wash:** Wound irrigation with normal saline  **Method of skin closure:** PDS Plus sutures  **Other:** Wound management according to CDC guideline recommendations | Emergency: 4 (0.8) |
|  | Vicryl | 67.5 (11.6) | 322 (63.8) |  | Yes  Antibacterial prophylaxis: All patients | **Wound dressing:** sterile dressing for ≥48 hours  **Sterile saline wash:** Wound irrigation with normal saline  **Method of skin closure:** PDS II sutures  **Other:** Wound management according to CDC guideline recommendations | Emergency: 7 (1.4) |
| Isik 2012, Turkey  Source: {Isik, 2012 #320} | Vicryl Plus | 60.15 (10.77) | 110 (64.7) | **Bathing with soap:** NR  **Nasal decolonisation:** NR  **Other:** NR | No | **Wound dressing:**  NR  **Sterile saline wash:** NR  **Method of skin closure:** NR  **Other:** Discharge training on wound care, arranged and provided by an experienced nurse specialised in cardiac rehabilitation | Elective: 168 (98.8)  Emergency: 2 (1.2) |
|  | Vicryl | 61.21 (10.25) | 228 (67.1) |  | No |  | Elective: 326 (95.9)  Emergency:14 (4.1) |
| Justinger 2013, Germany  Other identifiers:  NCT00998907  Source:  Primary: {Justinger, 2013 #291}  Secondary: {University Hospital, 2009 #698} | PDS Plus | 63 (SED 13) | 301 (62.1) | **Bathing with soap:** Regular shower without iodine within 24 hours before surgery  **Nasal decolonisation:** NR  **Other:** Abdominal hair removal following the preoperative shower | Yes  Antibacterial prophylaxis: All patients | **Wound dressing:** NR  **Sterile saline wash:** NRo  **Method of skin closure:** Staples  **Other:** Skin disinfected with polyvidone iodine in alcohol following skin closure; sterile drape for ≥24 hours | Elective: 485 (100) |
|  | PDS II | 63 (SED 13 | 224 (60.4) |  | Yes  Antibacterial prophylaxis: All patients |  | Elective: 371 (100) |
| Karip 2016, Turkey  Source: {Karip, 2016 #203} | Monocryl Plus | 25.89 (6.07) | NR (only overall across treatments: 83 (78.3%) male) | **Bathing with soap:** NR  **Nasal decolonisation:** NR  **Other:** NR | Yes in revised trial  IV antibacterial prophylaxis: All patients | **Wound dressing:** NR  **Sterile saline wash:** NR  **Method of skin closure:** NR  **Other:** Analgesics prescribed, but no antimicrobial therapy | Elective: 54 (100) |
|  | Monocryl | 25.73 (6.64) | NR (only overall across treatments: 83 (78.3%) male) |  | Yes in revised trial  IV antibacterial prophylaxis: All patients |  | Elective: 52 (100) |
| Lin 2018, Taiwan  Other identifiers:  NCT02533492  Source:  Primary: {Lin, 2018 #154}  Secondary: {Mel Shiuann-Sheng Lee, 2015 #796} | Vicryl Plus | 71.3 (7.7) | 15* (29.4*) | **Bathing with soap:** NR  **Nasal decolonisation:** NR  **Other:** Standard clinical pathway | Yes  Systemic antibacterial prophylaxis: All patients | **Wound dressing:** NR  **Sterile saline wash:** NR  **Method of skin closure:** Staples  **Other:** Standard clinical pathway | Elective 51 (100) |
|  | Vicryl | 70.0 (7.1) | 11* (21.6*) |  | Yes  Systemic antibacterial prophylaxis: All patients |  | Elective 51 (100) |
| Mattavelli 2015, Italy  Other identifiers:  NCT01869257  Source:  Primary: {Mattavelli, 2015 #245}  Secondary: {University of Milano Bicocca, 2013 #718} | Vicryl Plus + PDS Plus | Median 69 (IQR: 60-75) | 81 (57.8) | **Bathing with soap:** NR  **Nasal decolonisation:** NR  **Other:** Hair removal either the evening before the operation or the morning of the operation | Yes  Antibacterial prophylaxis: All patients | **Wound dressing:** NR  **Sterile saline wash:** NR  **Method of skin closure:** 3/0 Vicryl Plus suture  **Other:** NR | Elective: 140 (100) |
|  | Vicryl + PDS II | Median 69 (IQR: 60-76) | 74 (52.4) |  | Yes  Antibacterial prophylaxis: All patients |  | Elective: 141 (100) |
| Mingmalairak 2009, Thailand  Source: {Mingmalairak, 2009 #359} | Vicryl Plus | 29.1 (SD NR) | 26 (52) | **Bathing with soap:** NR  **Nasal decolonisation:** NR  **Other:** NR | Yes  Antibiotic prophylaxis: All patients. Gentamicin 240 mg and metronidazole 500 mg, were given intravenously 30-60 minutes before operation | **Wound dressing:** NR  **Sterile saline wash:** NR  **Method of skin closure:** Sutures as assessed  **Other:** “The appendectomy was done with standard technique.” | NR explicitly or by arm. Study does state that “Cases of appendicitis were divided into uncomplicated (76%), which were acute (24%) and suppurative (52%) and complicated appendicitis (24%), which were gangrene (8%) and ruptured (16%).” |
|  | Vicryl | 29.8 (SD NR) | 35 (70) |  | Yes  Antibiotic prophylaxis: All patients. Gentamicin 240 mg and metronidazole 500 mg, were given intravenously 30-60 minutes before operation |  |  |
| Nakamura 2013, Japan  Other identifiers: UMIN00003322  Source:  Primary: {Nakamura, 2013 #303}  Secondary: {Teine Keijinkai Hospital, 2010 #707} | Vicryl Plus | 69.4 (11.3) | 130 (63.1%*) | **Bathing with soap:** NR  **Nasal decolonisation:** NR  **Other:** NR | Yes  Antibacterial prophylaxis: All patients | **Wound dressing:** NR  **Sterile saline wash:** NR  **Method of skin closure:** Staples  **Other:** NR | Elective: 206 (100) |
|  | Vicryl | 70.2 (11.1) | 112 (54.9%*) |  | Yes  Antibacterial prophylaxis: All patients |  | Elective: 204 (100) |
| Olmez 2019, Turkey  Source: {Olmez, 2019 #106} | PDS Plus | 55.1 (16.3) | 192 (43.1) | **Bathing with soap:** Site cleaned with polyvidone-iodine  **Nasal decolonisation:** NR  **Other:** Site shaved prior to surgery (day before) | Yes  Antibiotic prophylaxis: All patients. 1000mg cefazolin, 1000mg ceftriaxone, 500mg metronidazole prior to / at start of surgery | **Wound dressing:** NR  **Sterile saline wash:** NR  **Method of skin closure:** 3/0 polypropylene suture, which was removed on post-operative day 14 is complications had not occurred in the incision  **Other:** NR | Emergency: 31 (6.9) |
|  | PDS II | 54.6 (16.9) | 223 (50.1) |  | Yes  Antibiotic prophylaxis: All patients. 1000mg cefazolin, 1000mg ceftriaxone, 500mg metronidazole prior to / at start of surgery |  | Emergency: 74 (16.6) |
| Rasic 2011, Croatia  Source: {Rasic, 2011 #334} | Vicryl Plus | 58 (14.5) | 49 (54) | **Bathing with soap:** NR  **Nasal decolonisation:** NR  **Other:** NR | Yes  Antibiotic prophylaxis (given intravenously during induction of anaesthesia): all patients | **Wound dressing:** NR  **Sterile saline wash:** NR  **Method of skin closure:** Polyamide Ethicon 2-0  **Other:** NR | Elective: 91 (100) |
|  | Vicryl | 57 (14.7) | 50 (54) |  |  |  | Elective: 93 (100) |
| Renko 2017, Finland  Other identifiers:  NCT01220700  Source:  Primary: {Renko, 2017 #187}  Secondary: {University of Oulu, 2010 #762} | Triclosan-coated sutures (Vicryl Plus, Monocryl Plus, or PDS Plus) | 7.2 (5.4) | 483 (62) | **Bathing with soap:** NR  **Nasal decolonisation:** NR  **Other:** NR | Not reported whether antibiotic use was pre- or post-operative  Prophylactic antibiotics: 236 (30%) | **Wound dressing:** NR  **Sterile saline wash:** NR  **Method of skin closure:** study suture (intracutaneous), non-absorbing skin sutures, staples, other sutures, undefined  **Other:** Operating room used standard hygienic procedures to prevent SSIs in accordance with CDC recommendations (1999) | Emergency surgery: 95/728 (13%) |
|  | Non-coated sutures (Vicryl, Monocryl, or PDS) | 7.1 (5.5) | 502 (64) |  | Not reported whether antibiotic use was pre- or post-operative  Prophylactic antibiotics: 245 (31%) |  | Emergency surgery: 92/725 (13%) |
| Rozzelle 2008, USA  Source: {Rozzelle, 2008 #366} | Vicryl Plus | 9.7 (11.4)  *The youngest patient in the study was 1 day old* | 30 (65) | **Bathing with soap:** Yes  All participants received preoperative chlorhexidine skin cleansing and, betadine skin preparation  **Nasal decolonisation:** NR  **Other:** Authors report use of iodine-impregnated adhesive drapes, and silicone shunt components were soaked in bacitracin solution before implantation | Yes  All participants received preoperative intravenous antibiotics (cefazolin, or vancomycin if allergic to cephalosporins) and antibiotic wound irrigation prior to closure | **Sterile saline wash:** NR  **Wound dressing:** NR  **Method of skin closure:** Skin closures for all procedures were performed with poliglecaprone 25 sutures (Monocryl; Ethicon, Inc.).  **Other:** NR | NR |
|  | Vicryl | 9.9 (9.8)  *The youngest patient in the study was 1 day old* | 18 (47) |  | Yes  All participants received preoperative intravenous antibiotics (cefazolin, or vancomycin if allergic to cephalosporins) and antibiotic wound irrigation prior to closure |  | NR |
| Ruiz-Tovar 2020, Spain  Other identifiers: NCT03763279  Source:  Primary: {Ruiz-Tovar, 2020 #12}  Secondary:  {Hospital General Universitario Elche, 2018 #702} | Stratafix Symmetric Plus | 65.8 (16.9) | 28 (56.0*) | **Bathing with soap:** NR  **Nasal decolonisation:** NR  **Other:** Clorhexidine-alcohol solution | Perioperative systemic antibiotics given and maintained for ≥5 days because all cases were considered severe intra-abdominal infection Prolongation decided on clinical evolution | **Wound dressing:** NR  **Sterile saline wash:** Yes  **Method of skin closure:** Staples  **Other:** NR | Emergency 50 (100) |
|  | PDS Plus Loop | 64.7 (15.9) | 26 (52.0*) |  |  |  | Emergency 50 (100) |
|  | PDS Loop | 63.2 (17.8) | 25 (50.0*) |  |  |  | Emergency: 50 (100) |
| Ruiz-Tovar, 2015, Spain  Source: {Ruiz-Tovar, 2015 #242} | Triclosan-coated sutures (brand NR) | 63.8 (15.5) | 31 (62*) | **Bathing with soap:** NR  **Nasal decolonisation:** NR  **Other:** NR | Yes  Peri-operative systemic antibiotics (imipenem 1 g/8 h intravenous [IV]) were used in both groups. In case of allergies to b-lactams, tigecycline (100 mg IV as starting dose, followed by 50 mg/12 h IV) was used. Both antibiotics were maintained for a minimum of 7 days | **Wound dressing:** NR  **Sterile saline wash:**Yes; After fascial closure, subcutaneous tissue was irrigated with 500 mL of normal saline  **Method of skin closure:** Staples  **Other:** During the operation, the skin was prepared with chlorhexidine-alcohol solution, the incision was protected with adhesive plastic devices, body temperature was maintained with thermal blankets, and intravenous fluid infusion was optimized with a FloTrac sensor (Edwards, Irvine, CA). | NR |
|  | Uncoated sutures (brand NR) | 65.6 (14.9) | 31 (60.8*) |  | Yes  Peri-operative systemic antibiotics (imipenem 1 g/8 h intravenous [IV]) were used in both groups. In case of allergies to b-lactams, tigecycline (100 mg IV as starting dose, followed by 50 mg/12 h IV) was used. Both antibiotics were maintained for a minimum of 7 days |  | NR |
| Santos 2019, Brazil  Source: {Santos, 2019 #125} | Vicryl Plus | 62.01 (8.62) | 175 (69.7) | **Bathing with soap:** Yes  Preoperative decolonisation with a chlorhexidine bath one hour before going to the surgical centre. Asepsis was done on the operating room with soap chlorhexidine followed by alcoholic chlorhexidine  **Nasal decolonisation: Yes**  Nasal Mupirocine twice a day during the five days before surgery  **Other:** NR | Yes  Antibiotic prophylaxis: All patients | **Wound dressing:** NR  **Sterile saline wash:** NR  **Method of skin closure:** Sutures as assessed  **Other:** NR | NR |
|  | Vicryl | 60.39 (9.03) | 180 (70.0) |  | Yes  Antibiotic prophylaxis: All patients |  | NR |
| Seim 2012, Norway  Source: {Seim, 2012 #316} | Vicryl Plus | 63.5 (0.7) | 143* (89.4*) | **Bathing with soap:** Yes (shower with soap and Hibiscrub (chlorhexidinegluconate) the evening before and day of surgery)  **Nasal decolonisation:** NR  **Other:** Hair removal on afternoon of the day before surgery. Skin disinfected with chlorohexidine solution (5 mg/ml in 70% ethanol) | Yes  Antibacterial prophylaxis: All patients | **Wound dressing:** NR  **Sterile saline wash:** NR  **Method of skin closure:** NR  **Other:** Drape, compresses, and elastic bandages initially; customized stockings for approximately 3 weeks | Elective 164 (100) |
|  | Vicryl | 63.1 (0.8) | 144* (88.3*) |  | Yes  Antibacterial prophylaxis: All patients |  | Elective 164 (100) |
| Soomro 2017, Pakistan  Source: {Soomro, 2017 #528} | Triclosan-coated sutures (brand NR) | Unclear: text states 25.70 (3.10) while table states 25.86 (3.51) | 0 | **Bathing with soap:** All wounds were prepped using povidone iodine scrub and solution  **Nasal decolonisation:** NR  **Other:** NR | Yes  Antibiotic prophylaxis: All patients | **Wound dressing:** Yes; “standard dressings”  **Sterile saline wash:** NR  **Method of skin closure:** NR  **Other:** Standard post-operative instructions were given to all patients for wound care | NR |
|  | Non-coated sutures (brand NR) | Unclear: text states 25.86 (3.51) while table states 25.70 (3.10) | 0 |  | Yes  Antibiotic prophylaxis: All patients |  | NR |
| Sprowson 2018, UK  Other identifiers:  ISRCTN17807356  Source:  Primary: {Sprowson, 2018 #152}  Secondary: {Sprowson, 2014 #263} | Vicryl Plus | 67.5 (10) | 563 (46.0%*) | **Bathing with soap:** NR  **Nasal decolonisation:** NR  **Other:** (Patients followed the standardised pathway from outpatient appointment to operation date) | Yes  Antibacterial prophylaxis: All patients | **Wound dressing:** From October 2009, Aquacel Surgical dressings; prior to this, dressing choice at preference of surgeon  **Sterile saline wash:** NR  **Method of skin closure:** Subcuticular  skin closure technique or metal clips  **Other:** Standardised enhanced recovery pathway | Elective: 1223 (100) |
|  | Vicryl | 67.2 (9.7) | 604 (45.6%*) |  | Yes  Antibacterial prophylaxis: All patients |  | Elective: 1323 (100) |
| Sukeik 2019, UK  Other identifiers:  ISRCTN 21430045  Source:  Primary: {Sukeik, 2019 #33}  Secondary: {University College London, 2013 #785} | Vicryl Plus | 68.65 (10.90) | 25 (30.9*) | **Bathing with soap:** NR  **Nasal decolonisation:** NR  **Other:** Perioperative care plans (unspecified) similar for hip and knee procedures  Patients have undergone pre-operative optimisation prior to surgery | Yes  Antibiotic prophylaxis (first dose at induction of anaesthesia). | **Wound dressing:** Yes (for knee arthroplasty)  **Sterile saline wash:** NR  **Method of skin closure:** Skin clips  **Other:** Anti-embolism stockings and low molecular weight heparin (thromboprophylaxis)  From ISRCTN: standard postoperative treatment | Elective: 81 (100) |
|  | Vicryl | 67.85 (9.85) | 24 (34.8*) |  |  |  | Elective: 69 (100) |
| Sundaram 2020a, USA  Other identifiers: NCT03285529  Source:  Primary: {Sundaram K, 2020a #1451}  Secondary: {The Cleveland Clinic, 2017 #778} | Stratafix Symmetric PDS Plus | 68 (7) | 14 (47) | **Bathing with soap:** NR  **Nasal decolonisation:** NR  **Other:** NR | No | **Wound dressing:** Surgical dressing  **Sterile saline wash:** NR  **Method of skin closure:** adhesive strips (Steristrips)  **Other:** NR | Elective: 30 (100) |
|  | Vicryl | 66 (7) | 13 (43) |  | No |  | Elective: 30 (100) |
| Sundaram 2020b, USA  Other identifiers: NCT03285555  Source:  Primary: {Sundaram, 2020b #1452}  Secondary: {The Cleveland Clinic, 2017 #779} | Stratafix Symmetric PDS Plus | 61 (13) | 17 (57) | **Bathing with soap:** NR  **Nasal decolonisation:** NR  **Other:** NR | No | **Wound dressing:** Surgical dressing  **Sterile saline wash:** NR  **Method of skin closure:** adhesive strips (Steri-strips)  **Other:** NR | NR but appears to be Elective: 30 (100) |
|  | Vicryl | 66 (10) | 11 (37) |  | No |  | NR but appears to be Elective: 30 (100) |
| Tabrizi 2019, Iran  Other identifiers: NCT03659344  Source:  Primary: {Tabrizi, 2019 #129}  Secondary: {Shiraz University of Medical Sciences, 2018 #722} | Vicryl Plus | 44.73 (12.82) | 83 (51.9) | **Bathing with soap:** NR  **Nasal decolonisation:** NR  **Other:** Patients rinsed with 0.2% Chlorhexidine mouthwash before dental implant surgery and were instructed to continue using it for 7 days postoperatively | Yes  Antibiotic prophylaxis: All patients | **Wound dressing:** NR  **Sterile saline wash:** Yes in patients with a peri-implant infection: surgical sites were irrigated locally with normal saline and chlorhexidine 0.2%  **Method of skin closure:** NR  **Other:** | NR |
|  | Vicryl | 44.64 (12.24) | 88 (55) |  | Yes  Antibiotic prophylaxis: All patients |  | NR |
| Thimour-Bergström 2013, Sweden  Other identifiers:  NCT01212315  Source:  Primary: {Thimour-Bergstrom, 2013 #300;Steingrimsson, 2015 #231}  Secondary: {Turtiainen, 2014 #287;Jeppsson, 2014 #288;Sahlgrenska University Hospital, 2010 #815} | Vicryl Plus + Monocryl Plus | **Open vein harvesting:** 67.6 (8.3)  **Sternotomy**: 67.6 (8.1) | **Open vein harvesting**: 145* (78.8*)  **Sternotomy:** 138* (77.1%*) | **Bathing with soap:** NR  **Nasal decolonisation:** NR  **Other:** NR but operations conducted using standard techniques | Yes  Antibacterial prophylaxis: All patients | **Wound dressing:** NR  **Sterile saline wash:** NR  **Method of skin closure:** one continuous subcutaneous suture and one continuous intracutaneous suture  **Other:** Wound covered with drape, compresses, and elastic bandage | Elective: 184 (100) |
|  | Vicryl + Monocryl | **Open vein harvesting:** 66.9 (8.1)  **Sternotomy:** 66.7 (8.2) | **Open vein harvesting:** 159* (83.7*)  **Sternotomy:** 150* (84.3%*) |  | Yes  Antibacterial prophylaxis: All patients |  | Elective: 190 (100) |
| Turtiainen 2012, Finland  Source: {Turtiainen, 2012 #317} | Vicryl Plus and Monocryl Plus | 72 (11) | 87 (63) | **Bathing with soap:** NR  **Nasal decolonisation:** NR  **Other:** NR | Yes  All but one of the included patients across both arms received antibiotic prophylaxis | **Wound dressing:** NR  **Sterile saline wash:** NR  **Method of skin closure:** NR  **Other:** NR | Non-emergency surgery: 139 (100) |
|  | Vicryl and Monocryl | 72 (11) | 86 (63) |  | Yes  All but one of the included patients across both arms received antibiotic prophylaxis |  | Non-emergency surgery: 137 (100) |
| Williams 2011, UK  Source: {Williams, 2011 #326} | Vicryl Plus or Monocryl Plus | Median: 61 (32–87) | 0 | **Bathing with soap:** NR  **Nasal decolonisation:** NR  **Other:** NR | Antibiotic prophylaxis for surgery considered at high risk (high BMI, mastectomy, or axillary clearance): n=5 | **Wound dressing:** Yes  Wounds were dressed with Steri-Strips (3M, St. Paul, MN) and Tegaderm (3M) or Cosmopore (Hartmann USA, Rock Hill, SC) or Primapore (Smith & Nephew, Hull, UK), or Cosmopore alone, again at the discretion of the surgeon**.**  **Sterile saline wash:** NR  **Method of skin closure:** Sutures as assessed  **Other:** NR | Elective: 175 (100) |
|  | Vicryl or Monocryl | Median: 59 (30– 80) | 0 |  | Antibiotic prophylaxis for surgery considered at high risk (high BMI, mastectomy, or axillary clearance): n=3 |  | Elective: 175 (100) |
| Zhang 2011, China  Other identifiers:  NCT00768222  Source:  Primary: {Zhang, 2011 #340}  Secondary: {Ethicon Inc., 2008 #731} | Vicryl Plus | Median 51.0 (range, min-max: 32.0-82.0) | 0 (0) | **Bathing with soap:** NR  **Nasal decolonisation:** NR  **Other:** NR | No | **Wound dressing:**  NR  **Sterile saline wash:** NR  **Method of skin closure:** NR  **Other:** NR | Elective: 51 (100) |
|  | Chinese silk | Median 52.0 (range, min-max: 34.0-75.0) | 0 (0) |  | No |  | Elective: 50 (100) |

### Supplementary_Table_7: Risk of bias assessment of studies included in the review

| **Study**  *Author, year, location* | **Was randomisation carried out appropriately?** | **Was the concealment of treatment allocation adequate?** | **Were the groups similar at the outset of the study in terms of prognostic factors, for example, severity of disease?** | **Were the care providers and participants blind to treatment allocation? If any of these people were not blinded, what might be the likely impact on the risk of bias (for each outcome)** | **Were the outcome assessors blind to treatment allocation? If any of these people were not blinded, what might be the likely impact on the risk of bias (for each outcome)** | **Were there any unexpected imbalances in dropouts between groups? If so, were they explained or  adjusted for?** | **Is there any evidence to suggest that the authors measured more outcomes than they reported?** | **Did the analysis include an intention to-treat analysis? If so, was this appropriate and were appropriate methods used to account for missing data?** | **Additional info** |
| --- | --- | --- | --- | --- | --- | --- | --- | --- | --- |
| Arslan 2018, Turkey ^[20]^ | Unclear  Randomised in blocks at a ratio of 1:1, but method of sequence generation (e.g., by computer) not reported | Unclear  No details of allocation concealment reported | Yes  Baseline and clinical characteristics for the groups appear similar, with non-significant p values between groups | No  Partially-blinded: the operating surgeon was not blinded as they recognised the sutures. However, since postoperative care was conducted by another surgeon they were presumably unaware of treatment assignment, although not explicitly stated. Blinding of the patients was not reported | Unclear  A surgeon other than the operating surgeon (who was not blinded) assessed the surgical site. He/she was presumably unaware of treatment assignment, although not explicitly stated | No  No unexpected imbalance in study discontinuations, which were few and all due to protocol violations, between groups | No  All pre-specified primary and secondary outcomes were reported | No  Analysis population comprised all treated patients | NA |
| Baracs 2011, Hungary ^[21]^ | Yes  Randomisation carried out by computer software (stored in a password protected website) and could not be influenced manually | No  No details of allocation concealment reported | Yes  Baseline characteristics for the groups appear similar, with non-significant p values between groups for all recorded data points | Unclear  TRR record states masking was “Double (Care Provider, Outcomes Assessor)” but no details in paper of how this was achieved | Unclear  TRR record states masking was “Double (Care Provider, Outcomes Assessor)” but no details in paper of how this was achieved | Unclear  Withdrawals were not reported by arm | Yes  Not all stated secondary outcomes were reported and outcomes in publication not stated in TRR | No  Per protocol population appears to have been used; no details of how this was adjusted for | NA |
| Diener 2014, Germany ^[23]^ | Yes  The authors used a centralised web-based device (Randomizer Software) for randomisation, with a specific code for each participating centre, to achieve equivalent groups. Permuted-block randomisation with an allocation ratio of 1:1 and a block size of 4 was used. | Yes  Use of randomisation software ensured “the randomisation  sequence was concealed” | Yes  The study groups were well balanced in terms of  patient and procedure characteristics | Yes  Patients, surgeons, and the outcome assessors were masked to the suture material used | Yes  Outcome assessment was masked and monitored | No imbalances  3.3% and 3.1% of patients in the intervention and control arm were excluded or dropped out | No  All outcomes reported | Yes  Analysis conducted using modified ITT to represent clinical practice  Missing values for primary outcome were replaced by random imputation with probability equal to the SSI rate recorded for the complete cases in the respective treatment group | NA |
| Ford 2005, USA ^[28]^ | Unclear  Patients were randomised to treatment at a ratio of 2:1, but method of sequence generation (e.g., by computer) not reported | Unclear  No details of allocation concealment reported | Unclear  Authors stated that there were no differences in baseline demographic variables between the treatment  groups. However, demographic details, where reported, were very limited and not reported separately according to suture group | No  Study reported to be open-label | Unclear  The primary endpoint was the surgeon’s blinded assessment of the overall intraoperative handling characteristics of each suture. However, the study was reported to be open-label | No  A similar proportion of patients withdrew or were lost to follow-up in each group | No  All pre-specified primary and secondary outcomes were reported | No  Analysis based on observed cases, i.e., patients at each assessment point | Small sample size with only 151 patients randomised to the two treatments; the 2:1 ratio meant group sizes of 100 and 51 patients |
| Galal 2011, Egypt ^[29]^ | Yes  A computer-generated list was used to randomise patients to treatment | Unclear  Treatment allocation was by random dispensing, one at a time, a sealed pack containing the suture; unclear whether the packs were numbered, opaque, and free of any identifying marks | Yes  No significant differences between the two groups in demographics and risk factors for SSI | Yes  Double-blind trial. The research team (surgeon, nurse, microbiologist) and patients were unaware of the treatment assigned | Yes  Double-blind trial. The research team (surgeon, nurse, microbiologist) and patients were unaware of the treatment assigned | No  No withdrawals or loss to follow-up. All enrolled patients were included in the analyses | No  Primary outcome reported. Secondary outcomes were not pre-specified but other outcomes evaluated were reported | Unclear  ITT not explicitly reported but all enrolled patients appear to have been analysed according to the treatment allocated. No methods to account for missing data were described | Yes  The number of patients in each group according to wound classification needs clarification as there is a potential error in the reporting in Table 2. However, it is unclear whether all patients had their wound classified and whether any patients had >1 wound site (e.g., CABG).  The authors acknowledged that the local protocol for infection control they followed may deviate from current modern practices |
| Ichida 2018, Japan ^[30]^ | Unclear  Patients randomised to treatments using permuted blocks with block size of 2, but method of sequence generation (e.g., by computer) not reported | Yes  Treatment allocation was conducted using sealed envelopes according to the randomisation list. A research nurse opened the sealed envelope and delivered the allocated sutures to the operating theatre. Neither the reseach nurse nor the doctor who prepared the envelopes were involved in the operation or follow-up | Yes  The treatment groups were well balanced in terms of preoperative demographic characteristics and there were no significant differences between them | Yes  Patients, surgeons, and nurses in the surgical wards, were all blinded to treatment allocation. Coated and uncoated sutures were removed from their packaging and placed in the operating theatre with any identifying marks. The sutures looked identical in physical appearance and were indistinguishable in terms of physical properties (e.g., texture, tying properties).  The randomisation code was kept separately from  the trial data until the end of the study | Yes  The surgeons who assessed the wound status were also  blinded, because the used suture material could not be identified  postoperatively. The randomisation code was kept separately from  the trial data until the end of the study | No  There were no losses to follow-up or study discontinuations in either group | No  The primary end point was reported. Secondary end points were not prespecified. However, the authors also reported the incidence of bacterial species found in infected wounds | No  The analysis was conducted using the modified ITT population (excluded patients who did not receive any of the allocated interventions) and methods used to account for missing data were not reported | Yes  Given a lack of published data, the authors performed the sample size calculation using data derived from a retrospective cohort of patients who underwent gastroenterologic surgery and had their abdominal wounds closed by the same procedure at their institution in 2012 |
| Isik 2012, Turkey ^[32]^ | No  Sequential randomisation of patients to treatment | Unclear  No details of allocation concealment reported | Yes  The two groups were similar with regard to demographics and clinical characteristics, with no significant differences between them | Unclear  Reported to be a double-blind trial, but no further details provided.Patients were allocated the treatment during the operation, when the nurse delivered the suture materials to the operating room | Unclear  Reported to be a double-blind trial; no other details relating to the outcome assessment were provided | No  Details of dropouts were not described. It appears that all included patients were analysed for sternal wound infections but not for leg wound infections. although this is likely to reflect the nature of the surgery undertaken | No  The main outcome was reported and there were no secondary outcomes | No  ITT analysis not explicitly conducted. The analysis appears to have been conducted on evaluable patients at follow-up at each of the two surgical sites (sternum and leg) | NA |
| Justinger 2013, Germany ^[33]^ | Unclear  Randomisation conducted in a group fashion, assigning groups of 50 to 100 consecutive patients to either of the two groups, rather than assigning treatments to individual patients. The method of sequence generation (e.g., by computer) not reported | Unclear  Details of group/treatment allocation were not reported | Yes  Baseline demographics of the two groups were generally comparable, with no significant differences between them | Yes  There is a discrepancy betweem the full text publication and the TRR in terms of masking. The published article describes this study as a double-blind trial. Surgeons and patients were all blinded to treatment allocation. The sutures were indistinguishable in terms of their physical properties.  The TRR describes the trial as open label (i.e. no masking) | Yes  Wound monitors were reported to blinded to treatment allocation | Unclear  Patient dropouts were reported overall but not by treatment group | Unclear  The primary outcome was reported. Although no secondary end points were pre-specified in the published article, there were some specified in the TRR which have not been reported. The authors did, however, report the proportion of bacterial species found in infected wounds | No  The analysis appears to have been conducted on randomised patients operated on who completed successful treatment. Methods to account for missing data were not reported | Yes  The study was a clinical pathway controlled trial, with randomisation conducted in a group fashion rather than individual patients. This was apparently used for logistic reasons and to facilitate a high patient recruitment rate. Details of patient flow through the study lacked clarity |
| Karip 2016, Turkey ^[35]^ | Yes  Patients randomised (1:1 ratio) to treatment using a randomisation program from the Internet | Unclear  No details of allocation concealment reported | Unclear  The two groups were of similar age and BMI, but no other baseline demographics or clinical characteristics were reported | Unclear  Reported to be double-blind, with patients unaware of and having no information on their treatment. However, there were no details of the operating surgeon being blinded to the suture material used | Yes  The surgeon who conducted post-operative assessments did not perform the surgery and was unaware of treatment allocation | No  Dropouts were not explicitly reported, but all randomised patients (in the revised trial) appear to have been included in the analysis | No  All pre-specified primary and secondary outcomes in the revised trial were reported | Unclear  ITT not explicitly reported but all randomised patients appear to have been included in the analysis. No methods to account for missing data were described | Yes  The original trial was designed primarily to investigate the effect of antibiotic prophylaxis, and secondarily of antibiotic-coated sutures. Following safety concerns, the ‘without antibiotic prophylaxis’ arm (n=21) was terminated early, and following protocol revision and approval, the trial continued with the 15 patients in the antibiotic prophylaxis arm and a further 91 patients recruited; the patients in the terminated arm were excluded from further analysis.  The overall sample size was small, with 106 patients randomised to the two suture materials |
| Lin 2018, Taiwan ^[36]^ | Unclear  Unspecified randomisation protocol was used to number sealed envelopes containing the suture materials, which were then randomly given to the patients | Yes  Treatments were allocated using consecutively numbered sealed envelopes containing the suture materials.  Only the circulating  nurse who opened the envelopes and the scrub nurse who  handled the suture materials were aware of the treatments allocated, but they were not involved in evaluating the study | Unclear  No significant differences between patients in the limited demographic characteristics reported (age, gender, height and weight) | Yes  Patients, clinical staff, operating surgeons, and the independent  study nurse who prospectively collected all perioperative information and outcome measures, were unaware of the assigned treatment | Yes  Radiographic and clinical assessments were conducted by an experienced clinician, blinded to group assignment and patients’ demographic data. Perioperative information and outcome measures were conducted by an independent study nurse who was also blinded to the treatment | No  No study withdrawals or loss to follow-up in either group | Yes  Not all secondary outcomes were reported, including length of hospital stay and some measures of skin condition  The TRR does not list all the outcomes specified in the publication, although it does refer to them in the trial rationale, with the addition of duration of antibiotic use | Yes  All patients completed the study and were included in the analysis. Methods to account for any missing data were not reported. | Yes  Specific age range of eligible patients. Small sample size, with approximately 50 patients randomised to each of the two groups. This was considered insufficient to demonstrate the superiority of triclosan-coated sutures in preventing SSIs in total knee arthroplasty. The authors also highlighted that the rigorous nature of the follow-up might have raised patient awareness of their wound conditions, and that the definition of SSI was limited to skin involvement only. |
| Mattavelli 2015, Italy ^[38]^ | Yes  Computerised randomisation list used to assign patients to treatment. Each study centre had an independent list | Yes  Treatment was allocated using  sealed, opaque, numbered envelopes that were opened sequentially by a registered nurse not involved in the trial | Yes  The two groups were well balanced in terms of demographic and baseline characteristics, although there was variation in some risk factors. The uncoated suture group contained a higher proportion of patients with a BMI <19 (7.1% vs 2.8% in the triclosan-coated group) and a lower proportion of patients with pre-operative radiochemotherapy (5.7% vs 12.1%). No statistical analysis was conducted | No  Patients were unaware of the treatment allocated for the full period of evaluation. Operating surgeons were aware of the suture material used as the trial organisers at each hospital were unable to obtain blind suture packages.  They were not  permitted to divulge the treatment allocation to patients or other staff during the study duration | Yes  Outcome assessors were unaware of the allocated treatment for the full  period of evaluation | No  Patient dropout due to a need for re-operation were similar in the two groups, and there were no losses to follow-up | No  All pre-specified primary and secondary outcomes were reported, as were the multivariate analyses of risk factors for SSI | No  The analysis appears to have been conducted on patients completing the study, and methods to account for missing data were not reported | Yes  The randomisation was not balanced for important and known patient and operative  risk factors for SSIs.  A second assessor confirmed all SSIs, but only 40% were confirmed by positive culture.  Organ/space  SSIs were not included in the primary outcome because suture coating was not expected to be involved in the occurrence of intra-peritoneal collection |
| Mingmalairak 2009, Thailand ^[40]^ | Yes  Sutures randomised by use of a random table (Fisher RA, Yate F. Statistic table for biological, agricultural and medical research. 6th ed. London: Longman Group; 1974: 134.) | Yes  Study was randomised and “The surgeon could not separate both types of sutures” | Yes  Groups were similar in age, weight and height; there were more men in the control arm but the difference did not reach statistical significance (p = 0.065) | Yes  Surgeons and surgical assistants were blinded | Unclear  Study claims to be double blind but no details are given beyond stating that the surgeons were blinded | No  All patients randomised were assessed and followed up | No  All stated outcomes are reported but reporting is incomplete in places (e.g., no SDs reported) | Yes  All patients completed the study and were included in the analysis. | No |
| Nakamura 2013, Japan ^[41]^ | Unclear  No details of random sequence generation or randomisation procedure | Unclear  Treatments were allocated using numbered envelopes, but appropriate safeguards (e.g., use of sealed or opaque envelopes, sequential numbers) were not described | Yes  Patients in both groups were similar in terms of demographics and risk factors for SSIs, with no significant differences between them | No  Patients, were blinded to the treatment assigned, whereas the surgeons were aware of the suture used | Yes  The physicians who assessed the wound infections were blinded to the treatment assignment | No  All randomised patients completed the study and were included in the analysis | No  All pre-specified primary and secondary outcomes were reported, including the secondary outcome (postoperative hospital stay) which was specified in the TRR but not the published article | Yes  ITT conducted since all randomised patients received the allocated intervention and were included in the analysis. Methods to account for any missing data were not reported | Yes  A high proportion (71%) of patients with wound infections were discharged after the same length of postoperative stay as non-infected patients, with infected wounds managed in the outpatient clinic. This was considered to be one of the factors why the reduction in hospital stay found with triclosan-coated sutures was less than that observed in other studies. |
| Olmez 2019, Turkey ^[43]^ | Yes  A computer generated list was used, created by an independent computer consultant | Unclear  Unclear how treatment concealment was carried out | No  More males in control group than intervention group (p = 0.037); higher BMI in control than intervention group (p = <0.0001); more smokers in control than intervention group (p = <0.0001) | Unclear  No details reported of blinding of surgeons, patients or care / nursing staff | Yes  Follow up and control tests were performed by a blinded researcher | No  Both groups enrolled 450 patients and analysed 445 | No  All stated outcomes were reported to some degree, although not all outcomes were clearly reported per arm | No  Study assessed completers only. No details given of methods for accounting for missing data | No |
| Rasic 2011, Croatia ^[44]^ | Yes  Computer-generated randomisation in blocks of 10 | Yes  Suture packets were prepared in sealed and numbered opaque envelopes, and assigned in order in the operating room | Yes  No statistically significant differences between groups in the limited baseline characteristics reported (age, gender, BMI) | Unclear  No details of blinding reported | Unclear  The patients were monitored by the same surgical team, but no details of blinding reported | Unclear  Study discontinuations were not reported, other than no deaths in either group | Yes  Primary and secondary outcomes were not explicit, but two of the parameters monitored appear not to have been reported (readmissions and haematomas)  No TRR available | Unclear  Analysis population not described. Table and figures did not report numbers of patients analysed. The percentage values reported in Table 2 appear to have been miscalculated using transposed numbers of patients randomised to the two groups | Yes  Outcome parameters were not assessed over the same time period for the entire study population, since they were only monitored during the hospitalisation period which would have varied on a patient basis |
| Renko 2017, Finland ^[45]^ | Yes  Computerised randomisation list in permuted blocks of four in a random order | Yes  Sealed, numbered opaque envelopes with the study group. The study nurse opened envelope and attached the study code and form to the child’s medical records, which accompanied child to the operating room | Yes  The groups were well balanced  in terms of baseline and perioperative characteristics | Yes  Two study nurses masked the suture packages, and all patients and their parents, and all other study personnel, were blinded to the study code. The packages containing the sutures were taped with opaque material so that only the  code was visible to the operating room staff. Suture materials were similar in colour, feel, and smell | Yes  Aside from the two study nurses who masked the suture packages and who did not participate in data collection or entry, all study personnel were blinded to the treatment code | No  Reasons for dropout were similar | No  The primary endpoint and all post hoc analyses were reported  Primary outcome reported in TRR, but no safety outcome reported or intention for post hoc analyses | Yes  The primary analysis was conducted using modified ITT, but methods to account for missing data were not described. If at least some amount of study  suture material was used during the operation according  to allocation, the patient was analysed in his or her allocation group. Per-protocol analyses were conducted for patients with no major protocol violations | The authors noted as a study limitation that not all suspected  SSIs were cultured or photographed because some patients  were treated at their own local health-care facilities instead of the study clinic |
| Rozzelle 2008, USA ^[47]^ | Yes  Randomisation was performed by the assignment of letter codes to study and placebo suture types | Yes  The suture type corresponding to a particular letter code was known only to operating room nurses and scrub technicians | Yes  Patient population characteristics did not differ significantly with regard to any factors known or suspected to influence shunt infection risk. Sex distribution between the groups was unequal, with a weak statistical trend toward more males in the Vicryl Plus group, but sex has never been identified as a risk factor for shunt infection | Yes  Participants and investigators were blinded to treatment assignment, because study and placebo sutures were indistinguishable after removal of the package labeling | Unclear  Unclear who performed outcome assessments | Unclear  NR by arm. Two patients with shunt infections subsequently died within the surveillance period. Both patients were infants with severe congenital anomalies whose parents ultimately decided to withdraw care | No  All specified outcomes are reported, although no correlation was found between patient baseline characteristics and shunt infection | Yes  All patients randomised were analysed | No |
| Ruiz-Tovar 2020, Spain ^[48]^ | Yes  Use of a random-number table | Unclear  Operating surgeon was unaware of treatment allocation before consenting, enrolling and initiating surgery. No other details of allocation concealment reported | Yes  No significant differences between groups in baseline characteristics or surgical procedure | No  Patients and epidemiology nurses were masked to the suture material used.  The operating surgeon knew the suture assignment before starting the abdominal wall closure | Yes  Outcomes were assessed by epidemiology nurses, and other surgeons in the team, who were masked to group assignment | No  No lost to follow-up or discontinuations in any group, and no significant difference between groups in patients excluded from the analysis | Yes  There is a discrepancy between the TRR and full text publication in how the primary and secondary outcomes are defined.  TRR reports one primary and one secondary outcome  Full text publication includes both TRR outcomes as primary and add others as secondary | No ITT  Per protocol analysis was used as authors considered that deceased patients or those undergoing reoperation might mask the results | Authors highlight that the study might be underpowered as they used a suboptimal estimation of the SSI rate in the control group for the power calculation.  The study was not powered for the development of the aggregation variables investigated in secondary analyses |
| Ruiz-Tovar 2015, Spain ^[50]^ | Yes  The patients were randomized by means of a sequentially numbered container method | Yes  Those who made the diagnosis…were blinded to the selection of the patient from the sequentially numbered container | Yes  Non-significant p values for all reported between group comparisons | No  Epidemiology nurse who evaluated the outcome of the surgical incision was the only person blinded to the allocated treatment | Yes  Epidemiology nurse who evaluated the outcome of the surgical incision was blinded to the allocated treatment | No  Death occurred in 9.1% and 7.3% of intervention and control groups respectively. No other dropouts were recorded | No  All stated outcomes were reported | No  Study assessed only those patients surviving to provide assessment data | NA |
| Santos 2019, Brazil ^[51]^ | Yes  A table was generated using a specific computational routine | Yes  The cardiovascular surgeon did not have prior access to the table (allocation was concealed) | Yes  P values comparing basline age, gender, BMI and diabetes status were all non-significant | Yes  Randomisation remained blinded to all participants in the surgical procedure, as well as to all those who were involved in its follow-up, except for the professionals responsible for randomisation and masking. In the masking process, counselors, the nurses responsible for the randomisation, the secretary, and surgical technologists learned about the drawn sutures/patients. Surgeons, the researchers and their assistants, and the patients were masked | Yes  The researchers and their assistants were masked | No  Drop outs were similar across arms (38 and 37 for intervention and control groups respectively) | No  All specified outcomes are reported | No  The study assessed outcomes using completers, with no description of any accounting for missing data | No |
| Seim 2012, Norway ^[52]^ | Unclear  No details of random sequence generation or randomisation procedure | Unclear  Treatment allocation was conducted using sealed envelopes which the surgeon opened on the day of surgery. It was not reported whether sequentially numbered, opaque envelopes were used. | Yes  Baseline demographic and clinical characteristics of the groups were comparable. With the exception of glucose levels, which were significantly higher in the Vicryl group (p=0.05), there were no significant differences between groups | No  All surgeons were aware of the suture material used. Blinding of the patients and other study personnel was not reported | Unclear  Blinding of the outcome assessors and patients was not reported. Following discharge, patients appear to have monitored their own wound healing | No  Drop-outs were few in both groups, and all were losses to follow-up | No  Primary and secondary outcomes were not explicitly specified. However, the study did report appropriate data in relation to the study aims | No  The analysis included all treatment completers | Yes  No scheduled follow-up visits.  Patients only appear to have been referred for GP examination post-discharge in the case of adverse healing or signs of infection |
| Soomro 2017, Pakistan ^[53]^ | No  No details of random sequence generation or randomiation procedure provided | No  No details of treatment concealment procedure provided | Unclear  The only baseline demographic reported is age, which was similar between arms | No  The principal investigator was blinded. Blinding of the patients and other study personnel such as surgeons was not reported | Yes  The principal investigator was blinded | No  All patients randomised are accounted for in reporting of the outcome | No  Primary and secondary outcomes were not explicitly specified. However, the study did report appropriate data in relation to the study aims | Yes  All patients completed the study and were included in the analysis. Methods to account for any missing data were not reported | Yes  Study included only clean wounds and the authors state that further studies with a larger sample size are needed |
| Sprowson 2018, UK ^[54]^ | Unclear  Quasi-randonised trial with treatments assigned according to date of surgery. Randomisation based on monthly assignment of the hospitals to one of the two interventions, with each centre providing one of the treatments for a calendar month | Yes  Treatments allocated using sealed, opaque envelopes randomised according to date of surgery. Envelopes were opened at the start of the month so allocation was unknown at the time when the patient was put on the waiting list (mean 3 months prior to surgery) | Yes  The two groups were well matched in demographics and comorbidities and were reported to be representative of patients undergoing total hip or knee arthroplasty in the UK. There were no statistically significant differences between the two groups | No  The patients, research team, statistician, and clinical staff were all blinded to the treatment assigned. The participating surgeon was aware of the suture material allocated | Yes  Outcome assessors were blinded to the treatment assigned. The statistician was also blinded | No  Losses to follow-up and deaths in the first 6 weeks were similar in the two groups, and no patients withdrew consent following randomisation | No  All primary and secondary endpoints specified in the published study were reported.  There were a few discrepancies between the secondary endpoints reported in the TRR, published protocol, and full text article | No  Rreported to be ITT but appears to be a modified ITT as patients who died or discontinued the study were not included in the analyses. Missing data was not expected to be a major concern, but was imputed if judged appropriate. Imputed datasets were analysed and reported, along with appropriate sensitivity analyses.  Table II reports demographics and cpmorbidities etc. for patients randomised to each group (n=1223 and n=1323) , but data appear to have been based on the mITT set (n=1164 and n=1273 | Yes  Authors stated that it was impossible to randomise individual patients to treatments for practical reasons (outlined in published protocol), and the approach taken was the best option.  There was a significant difference in the numbers of operations conducted at the three hospitals (p<0.001).  Neither the differences in surgical approach between surgeons, nor the grade of the surgeon were taken into consideration |
| Sukeik 2019, UK ^[56]^ | Yes  Randomisation was conducted by an external company. Block randomisation with unequal block size. Randomisation codes were only broken in the case of a  serious adverse event | Yes  Letter codes, corresponding to suture type, were assigned to the two groups and were known only to a team member who was not involved in the operation.  Consecutive allocation of treatments was conducted using sealed envelopes containing letter code cards | Yea  Patient demographics were comparable between groups, although there was a non-statistically significant difference in the proportion of patients with diabetes (12.3%* Vicryl Plus vs 5.8%* Vicryl) | Yes  Patients and surgeons were blinded to the assigned treatment. Both sets of sutures were indistinguishable once nurses had removed the package labelling | Yes  Personnel involved in assessing the wounds were blinded to treatment assignment | No  Similar numbers of patients in each group did not attend the 6-week follow-up | No  TRR retrospectively registered  Few discrepancies in primary and secondary endpoints reported in TRR and full text | Unclear  ITT analysis conducted but methods used to address missing data were not reported and 11 patients overall did not attend the 6-week follow-up | Yes  The trial was terminated prematurely due to the unavailability of the sutures after Dec 2014 (with 150 of 420 intended patients). The study was thus underpowered and the binary variable (ASEPSIS ≤10 vs >10) considered insignificant |
| Sundaram 2020a, USA ^[58]^ | Unclear  Computerised randomisation system used to randomise patients (1:1 ratio), but method of sequence generation (e.g. by computer) was not reported | Unclear  Sealed envelopes in a random order were used to allocate patient treatment. Not stated whether the envelopes were opaque and sequentially numbered | Yes  Table 1 shows that the two groups were well balanced in demographics and baseline characteristics, with no statistically significant differences between the two groups | No  Reported to be a single-blind trial.  A random envelope, which dictated the suture to be used, was drawn at the start of each arthroplasty.  Research personnel revealed the treatment assignment to the surgeon, but the patients remained unaware of the assigmment | Yes  Research personnel who conducted outcome assessments were blinded to the allocated treatment | No  All randomised patients completed the study; there were no losses to follow-up or study withdrawals | No  Primary and secondary outcomes were not explicitly specified, but the outcome measures defined appear to have been reported  There were a few discrepancies between the full publication and the TRR in the outcomes assessed | Yes  ITT analysis with all randomised patients included in the analysis. Methods to account for missing data were not described | Yes  Smalll sample size with only 60 patients overall.  There is a slight discrepancy between the full publication and the TRR in the eligibility criteria relating to BMI.  The study was considered adequately powered to detect differences in primary outcomes, but the sample size was limited for drawing conclusions  on secondary outcomes such as wound complications.  In addition, although the 90-day follow-up should capture most complications associated with this operation, it would miss those occurring outside of that time period |
| Sundaram 2020b, USA ^[60]^ | Unclear  Computerised randomisation system used to randomise patients (1:1 ratio) at time of consent, but method of sequence generation (e.g., by computer) was not reported | Unclear  Sealed envelopes in a random order were used to allocate patient treatment. Not stated whether the envelopes were opaque and sequentially numbered | No  Table 1 shows variations in the demographics and baseline characteristics between the two groups, although none were statistically significant. The most notable of these was the presence of more males in the Stratafix Symmetric PDS Plus group (57%) than in the Vicryl group (37%), (p=0.598) | No  Reported to be a single-blind trial. The patients were unaware of their assigned treatment as a random envelope, which dictated the suture to be used, was drawn at the start of each operation | Yes  Research personnel who conducted outcome assessments were blinded to the treatment allocation | No  All randomised patients completed the study; there were no losses to follow-up or study withdrawals | Yes  Primary and secondary outcomes were not explicitly specified, and not all outcome measures defined were reported (e.g., readmission and reoperation). Wound complications were reported overall and for two specific complications, not all those monitored.  There were few discrepancies between the full publication and the TRR in the outcomes assessed | Yes  ITT analysis with all randomised patients included in the analysis. Methods to account for missing data were not described | Yes  Smalll sample size with only 60 patients overall.  the power calculation was based on duration of arthrotomy closure, i.e. an operative measure, rather than one of patient efficacy.  There is a slight discrepancy between the full publication and the TRR in the eligibility criteria relating to BMI.  The authors highlighted that a formal cost analysis was outside the scope of the study. However, the TRR had pre-specified a cost comparison as a secondary endpoint. In addition, they had not used continuous locked suturing techniques in the comparator group as it was not the standard of care and there were safety concerns |
| Tabrizi, 2019, Iran ^[62]^ | Yes  Patients were randomly divided into two groups using a computer-generated randomisation list | Unclear  No details reported of methods of treatment allocation concealment | No  Difference between arms in patients receiving fresh socket implant (Vicryl Plus 21.2% vs Vicryl 15%) may have led to bias in results | No  Patients were blinded to the type of suture used. | No  Trial record states that the trial was single (participant) blinded only | No  All patients randomised are accounted for in reporting of the outcome | No  Primary and secondary outcomes were not explicitly specified. However, the study did report appropriate data in relation to the study aims | Yes  All patients completed the study and were included in the analysis. Methods to account for any missing data were not reported | Yes  Difference between arms in patients receiving fresh socket implant (Vicryl Plus 21.2% v. Vicryl 15%) may have led to bias in results |
| Thimour-Bergström 2013, Sweden ^[8]^ | Unclear  Randomised in blocks of 25, with stratification for diabetes, but method of sequence generation (e.g., by computer) not reported | Yes  Treatment allocated using sealed envelopes. A nurse not involved in patient follow-up opened the  randomisation envelope and delivered the sutures to the operating room | Yes  Table 1 shows the groups were similar in terms of patient characteristics, with no statistically significant differences between them | Yes  Surgeons and patients were unaware of the treatment assignment as a nurse not involved in the patients’ follow-up delivered and prepared the assigned treatment before the surgeon arrived at the operating room. Both the coated and non-coated sutures looked  identical outside of their packages, and were placed on the assist table without any identification marks | Yes  All the research nurses  involved in the follow-up of the patients were blinded to the treatment assignment.  All wound problems were classified by two independent observers, using the CDC definition, before the  randomisation code was broken | No  No unexpected imbalance in study discontinuations between groups, and reasons for dropout were similar | No  All pre-specified primary and secondary endpoints were reported.  A secondary analysis, based on the same patient cohort, aimed to investigate whether triclosan-coated sutures influenced the rate of sternal wound infections after CABG (Steingrimsson 2015). This also reported all pre-specified primary and secondary endpoints | No  Analysis conducted on the ‘as treated’ population | Yes  The secondary analysis of sternal wound infections was potentially underpowered: the power analysis was performed for leg wound infections that have a somewhat higher incidence than  in the sternotomy wound |
| Turtiainen 2012, Finland ^[67]^ | Yes  The coordinating centre performed block randomisation with a block size of four. The block randomisation was performed separately for each centre | Yes  A research secretary placed pieces of paper containing the randomisation allocations into sealed envelopes. A nurse opened each randomisation envelope in the operating theatre before the surgery. Only the nurses in the operating theatre knew to which group each patient had been randomised. | Yes  Baseline characteristics are tabulated and appear similar between arms | Yes  Neither the vascular surgeons, the nurses in the surgical ward, nor the patients knew to which group a patient had been randomised | Yes  Neither the vascular surgeons, the nurses in the surgical ward, nor the patients knew to which group a patient had been randomised. | No  All patients randomised are accounted for in reporting of the outcome | No  All stated outcomes are reported | Yes  All patients completed the study and were included in the analysis. Methods to account for any missing data were not reported | NA |
| Williams 2011, UK ^[68]^ | Yes  Randomisation was undertaken in blocks of 50 using random computer numbers | Yes  Randomisation was performed in the operating theatres using sequential sealed envelopes. Sutures used during the operations corresponded to the randomisation code | Yes  The authors report that “None of the [baseline] parameters were significantly different.” | Yes  Surgeon, patient, and the assessor at follow-up were blinded to which type of suture had been used. | Yes  Surgeon, patient, and the assessor at follow-up were blinded to which type of suture had been used. | No  Drop out rates were 14/75 (intervention) and 9/75 (comparator), so 19% and 12% per arm. Drop out rates due to a need for further surgery were also similar per arm (5/75 and 10/75) | No  All specified outcomes are reported | No  The study assessed outcomes using completers, with no description of any accounting for missing data | No |
| Zhang 2011, China ^[69]^ | Yes  Computer-generated randomisation schedule used. To ensure equal distribution of treatments in each centre, block randomisation (block size of 4) was conducted on a site basis | Yes  Patients allocated to treatment using sequentially numbered sealed envelopes, based on randomisation schedule | Yes  The two groups were comparable in baseline characteristics | No  Open label study in which the patients and surgeons were blinded up until the time of wound closure when the envelope was opened and the suture material revealed | No  Blinded assessment of the primary outcome was conducted by a central assessor. Assessment of the secondary outcomes does not appear to have been blinded | No  Droputs from the study were similar in both groups | Yes  Numerical data only reported for cosmetic outcomes and adverse effects.  Aside from a brief narrative description, the secondary outcomes of mean ASEPIS scores at various time points were not reported in the full publication despite numerical data being available from the TRR | Yes  ITT analysis conducted but missing data for the primary endpoint were not imputed. Per protocol analysis was also conducted on evaluable patients | Since this was a pilot study, a formal sample size calculation was not performed. Thus the study might be underpowered.  Small sample size (only 101 participants)  The authors highlighted that the study was not stratified to separate out the effect of the antibacterial properties of the active suture or the suturing technique; they considered this a limitation |

## 5.1. Results of the qualitative analyses

Length of hospital stay, hospital readmissions and severity of SSI were generally not well reported, with high heterogeneity between studies (e.g. different surgery types, different healthcare systems, and different reporting of outcomes).

### Post-operative use of antibiotics

Six studies ^[8, 20, 23, 28, 30, 36]^ reported information on antibiotic use for SSI (Supplementary Table 8). This outcome was generally poorly reported, and no differences in antibiotic use between the intervention and comparator arms were shown. Antibiotic use was not a primary outcome in any study, and only one ^[30]^ reported a significance value. Treatment decisions were not standardised, and in two studies ^[23, 28]^ it was unclear if the decision to use antibiotics was because of the SSI or due to other reasons (e.g. pre-existing infection).

### Supplementary_Table_8: Antibiotic use for SSI (presenting only those studies reporting eligible data by arm)

| **Study**  *Author, year, location* | **Timepoint of assessment** | **Subgroup: age**  *Adult / child / both / NR* | **Subgroup: wound class**  *Clean / clean-contaminated / contaminated / dirty / all / NR* | **Intervention or control, with name** | **Number of patients analysed**  *ITT or mITT unless specified* | **N (%) patients receiving post-operative antibiotics** | **Reason for antibiotics, if reported**  *SSI / NR* |
| --- | --- | --- | --- | --- | --- | --- | --- |
| Arslan 2018, Turkey ^[20]^ | Within 30 days post-surgery | Adult | All | PDS Plus + Vicryl Plus | 92 | 2 (2.2*) | SSI |
|  | Within 30 days post-surgery | Adult | All | Prolene + Vicryl | 95 | 2 (2.1*) | SSI |
| Diener 2014, Germany ^[23]^ | Within 30 days after index operation | Adults | All | PDS Plus | 587 | 126 (21.5) | NR |
|  | Within 30 days after index operation | Adults | All | PDS II | 598 | 112 (18.7) | NR |
| Ford 2005, USA ^[28]^ | 80 (± 5) days post-implantation | Child | Clean or clean-contaminated | Vicryl Plus | 76 (observed cases) | 17* (22) | NR |
|  | 80 (± 5) days post-implantation | Child | Clean or clean-contaminated | Vicryl | 38 (observed cases) | 11* (29) | NR |
| Ichida 2018, Japan ^[30]^ | Up to 30 days post-discharge | Both | All | Vicryl Plus | 508 | 88 (17.3) | SSI |
|  | Up to 30 days post-discharge | Both | All | Vicryl Plus | 505 | 85 (16.8) | SSI |
| Lin 2018, Taiwan ^[36]^ | Within 3 months post-surgery | Adult | Clean | Vicryl Plus | 51 | 0 | SSI |
|  | Within 3 months post-surgery | Adult | Clean | Vicryl | 51 | 2 (3.9)* | SSI |
| Thimour-Bergström 2013, Sweden ^[8]^ | 60 days post-surgery | Adults | NR | Vicryl Plus + Monocryl Plus | **Open vein harvesting:**184 (treated)  **Sternotomy**: 179 | **Open vein harvesting:** 20 (10.9)  **Sternotomy**: 24 (13.4) | SSI |
|  | 60 days post-surgery | Adults | NR | Vicryl + Monocryl | **Open vein harvesting:**190 (treated)  **Sternotomy**: 178 | **Open vein harvesting:** 35 (18.4)  **Sternotomy:** 24 (13.4) | SSI |

### Length of Hospital Stay and Incidence of Readmission

Twelve studies ^[23, 33, 38, 40, 41, 43, 44, 48, 50, 54, 56, 67]^ presented data on length of hospital stay (Supplementary Table 9). Nine of these found no statistical difference between the intervention and control arms ^[23, 33, 38, 40, 41, 50, 54, 56, 67]^. Ruiz-Tovar 2020 ^[48]^ reported a reduced length of stay for patients receiving Stratafix Plus and PDS Plus compared with non-coated PDS sutures (p = 0.006). Rasic 2011 ^[44]^ showed a shorter length of stay with Vicryl Plus (13.2 ± 1.3 days) compared with Vicryl sutures (21.4 ± 2.8 days; p < 0.05), and Olmez 2019 ^[43]^ reported a longer stay with PDS Plus (7.46 ± 1.7) compared with PDS sutures (6.70 ± 2.2; p < 0.0001).

Three studies reported rates of readmission (Supplementary Table 9); Sundaram 2020a ^[58]^ reported no readmissions in either arm, Sprowson 2018 ^[54]^ reported two (0.17%) readmissions in the intervention arm and none in the comparator arm, and Renko 2017 ^[45]^ reported five (1%) readmissions in the intervention arm and 17 (2%) in the comparator arm.

### Supplementary_Table_9: Hospital stay (presenting only those studies reporting eligible data by arm)

|  |  |  |  |  |  | **Initial stay** | | | **Readmission** | |
| --- | --- | --- | --- | --- | --- | --- | --- | --- | --- | --- |
| **Study**  *Author, year, location* | **Outcome definition and measure** | **Timepoint of assessment** | **Subgroup: age**  *Adult / child / both / NR* | **Subgroup: wound class**  *Clean / clean-contaminated / contaminated / dirty / all / NR* | **Intervention** | **Number of patients analyse**  *ITT or mITT unless specified* | **Length of initial post-operative hospital stay**  *Mean (SD)* | **Reason for stay**  *Due to SSI / overall / NR* | **Number of patients analysed**  *ITT or mITT unless specified* | **N (%) patients readmitted due to SSIs** |
| Diener 2014, Germany ^[23]^ | Overall postoperative hospital stay in days | Within 30 days after index operation | Adult | All | PDS Plus | 587 | 13.0 (7.4) | Overall | NR | NR |
|  |  |  | Adult | All | PDS II | 598 | 12.5 (6.3) | Overall | NR | NR |
|  |  | Within 30 days after index operation | Adult | All | PDS Plus | 587 | 2.3 (3.8) | NR | NR | NR |
|  |  |  | Adult | All | PDS II | 598 | 2.3 (3.6) | NR | NR | NR |
| Justinger 2013, Germany ^[33]^ | Duration of hospital stay, days | Up to 2 weeks post-discharge | Adult | All | PDS Plus | 485 (treatment completers) | Mean 11 (SEM 18) (median NR, range: 2 – 209) | NR | NR | NR |
|  |  | Up to 2 weeks post-discharge | Adult | All | PDS II | 371 (treatment completers) | Mean 15 (SEM 13) (median NR, range: 2 – 134) | NR | NR | NR |
| Mattavelli 2015, Italy ^[38]^ | Duration of hospital stay, days | Within 30 days post-discharge | Adult | Clean-contaminated | Vicryl Plus + PDS Plus | 140 (treatment completers) | Mean 12.3 (SD 6.5)  Median 11 (IQR: 9-15) | NR | NR | NR |
|  |  | Within 30 days post-discharge | Adult | Clean-contaminated | Vicryl + PDS II | 141 (treatment completers) | Mean 13.5 (SD 10.4)  Median 11 (IQR: 9-15 | NR | NR | NR |
| Mingmalairak 2009, Thailand ^[40]^ | Hospitalisation time in days | Unclear | Both | NR | Vicryl Plus | 50 | 3.7 (SD NR) | Overall | NR | NR |
|  |  | Unclear | Both | NR | Vicryl | 50 | 3.7 (SD NR) | Overall | NR | NR |
| Nakamura 2013, Japan ^[41]^ | Length of postoperative hospital stay, days | Within 30 days post-discharge | Both | Clean-contaminated | Vicryl Plus | 206 | Mean 15.2 (SD 11.6)  Median 11 (range: 6-79) | Overall | NR | NR |
|  |  | Within 30 days post-discharge | Both | Clean-contaminated | Vicryl | 204 | Mean 15.6 (SD 10.4)  Median 11.5 (range: 6-93) | Overall | NR | NR |
| Olmez 2019, Turkey ^[43]^ | Total hospital stay in days | Within 30 days post-surgery | Adult | All | PDS Plus | 445 (completers) | 7.46 (1.7) | Overall | NR | NR |
|  |  | Within 30 days post-surgery | Adult | All | PDS II | 445 (completers) | 6.70 (2.2) | Overall | NR | NR |
|  | Intensive care unit stay in days | Within 30 days post-surgery | Adult | All | PDS Plus | 445 (completers) | 2.98 (1.0) | Overall | NR | NR |
|  |  | Within 30 days post-surgery | Adult | All | PDS II | 445 (completers) | 2.69 (0.8) | Overall | NR | NR |
| Rasic 2011, Croatia ^[44]^ | Duration of hospital stay in days | Hospitalisation period | Adult | NR | Vicryl Plus | 91 | 13.2 (1.3) | NR | NR | NR |
|  |  | Hospitalisation period | Adult | NR | Vicryl | 93 | 21.4 (2.8) | NR | NR | NR |
| Renko 2017, Finland ^[45]^ | Readmission due to SSIs | Within 30 days post-surgery | Child | All | Triclosan-coated sutures (Vicryl Plus, Monocryl Plus, or PDS Plus | NR | NR | NR | 778 | 5 (1) |
|  |  | Within 30 days post-surgery | Child | All | Non-coated sutures (Vicryl, Monocryl, or PDS) | NR | NR | NR | 779 | 17 (2) |
| Ruiz-Tovar 2020, Spain ^[48]^ | Duration of hospital stay in days | 30 days postoperatively | Adult | Contaminated and dirty | Stratafix Symmetric Plus | 47 | Median: 4 (range: 2-14) | NR | NR | NR |
|  |  | 30 days postoperatively | Adult | Contaminated and dirty | PDS Plus Loop | 45 | Median: 5 (range: 2-21) | NR | NR | NR |
|  |  | 30 days postoperatively | Adult | Contaminated and dirty | PDS Loop | 47 | Median: 8 (range: 2-60) | NR | NR | NR |
| Ruiz-Tovar 2015, Spain ^[50]^ | Duration of hospital stay in days | 60 days post-surgery | NR | Dirty | Triclosan-coated suture | 50 | Median: 9 (range: 7-32) | NR | NR | NR |
|  |  | 60 days post-surgery | NR | Dirty | Uncoated suture | 51 | Median: 9.5 (range: 7-54) | NR | NR | NR |
| Sprowson 2018, UK ^[54]^ | Length of hospital stay, calculated as the number of nights in hospital from patient admission to discharge | 30 days post-surgery | Adult | NR | Vicryl Pus | 1164 | Median 3.9 | NR | 1164 | 2 (0.17) |
|  |  | 30 days post-surgery | Adult | NR | Vicryl | 1273 | Median 4.1 | NR | 1273 | 0 (0) |
| Sukeik 2019, UK ^[56]^ | Duration of hospital stay in days | Discharge from hospital | Adult | NR | Vicryl Plus | 81 | 6.23 (4.11) | NR | NR | NR |
|  |  | Discharge from hospital | Adult | NR | Vicryl | 69 | 6.13 (4.23) | NR | NR | NR |
| Sundaram 2020a, USA ^[58]^ | Wound-related readmission | 90 days post-surgery | Adult | NR | Stratafix Symmetric PDS Plus | NR | NR | NR | 30 | 0 (0) |
|  |  | 90 days post-surgery | Adult | NR | Vicryl | NR | NR | NR | 30 | 0 (0) |
| Turtiainen 2012, Finland ^[67]^ | Postoperative hospital stay in days | Unclear | Adult | NR | Vicryl Plus and Monocryl Plus | 139 | 5.5 (6.5) | NR | NR | NR |
|  |  | Unclear | Adult | NR | Vicryl and Monocryl | 137 | 5.2 (4.3) | NR | NR | NR |

### Severity of SSI and ASEPSIS score

Only three studies ^[8, 56, 69]^ reported severity using a mean or median ASEPSIS score by arm (Supplementary Table 10). Mean ASEPSIS score for wounds in the intervention arms varied from 2.54 (Sukeik 2019) to 3.7 (Thimour-Bergstrom 2013, leg wounds) and from 1.41 (Sukeik 2019) to 5.4 (Thimour-Bergstrom 2013, leg wounds) for wounds in the comparator arms.

Insufficient data were available for a meta-analysis of this outcome.

### Supplementary_Table_10: Severity of SSIs (presenting only those studies reporting eligible data by arm)

| **Study**  *Author, year, location* | **Outcome definition and measure** | **Timepoint of assessment** | **Subgroup**  *Overall / Adult / child / both / NR* | **Subgroup: wound class**  *Clean / clean-contaminated / contaminated / dirty / all / NR* | **Intervention** | **Number of patients analysed**  *ITT or mITT unless specified* | **N (%) of patients by score**  *As reported* | **ASEPSIS score by arm** |
| --- | --- | --- | --- | --- | --- | --- | --- | --- |
| Arslan 2018, Turkey ^[20]^ | Superficial or deep SSI, according to CDC (1992) guidelines | Within 30 days post-surgery | Adult | All | PDS Plus + Vicryl Plus | 86 (treated) | Superficial: 8 (9.3%*)  Deep: 1 (1.2%*) | NR |
|  | Superficial or deep SSI, according to CDC (1992) criteria | Within 30 days post-surgery | Adult | All | Prolene + Vicryl | 91 (treated) | Superficial: 18 (19.8%*)  Deep: 1 (1.1%*) | NR |
| Diener 2014, Germany ^[23]^ | Superficial or deep, according to modified CDC (1992) criteria | Within 30 days after index operation | Adult | All | PDS Plus | 587 | Superficial: 53  Deep: 22  Missing data: 12 | NR |
|  |  |  | Adult | All | PDS II | 598 | Superficial: 56  Deep: 25  Missing data: 15 | NR |
| Ichida 2018, Japan ^[30]^ | Incidence of superficial or deep SSI according to the CDC criteria | Within 30 days post-surgery | Both | All | Vicryl Plus | 508 | Superficial: 23 (4.5)  Deep: 12 (2.4) | NR |
|  |  | Within 30 days post-surgery | Both | All | Vicryl | 505 | Superficial:19 (3.7)  Deep: 11 (2.2) | NR |
| Lin 2018, Taiwan ^[36]^ | Incidence of SSI within based on wound condition | Within 3 months post-surgery | Adult | Clean | Vicryl Plus | 51 | Superficial: 0 (0)  Deep: 0 (0) | NR |
|  |  | Within 3 months post-surgery | Adult | Clean | Vicryl | 51 | Superficial: 2 (3.9%)  Deep: 0 (0) | NR |
| Mattavelli 2015, Italy ^[38]^ | Rate of superficial or deep incisional SSIs, according to CDC 1999 criteria | Within 30 days post-discharge | Adult | Clean-contaminated | Vicryl Plus + PDS PLus | 140 (treatment completers) | Deep: 4 (2.9)  Superficial: 14 (10.0) | NR |
|  |  | Within 30 days post-discharge | Adult | Clean-contaminated | Vicryl + PDS II | 141 (treatment completers) | Deep: 8 (5.7)  Superficial: 7 (4.7) | NR |
| Mingmalairak 2009, Thailand ^[40]^ | Incidence of superficial or deep incisional SSI; definition NR | Unclear | Both | NR | Vicryl Plus | 50 | Deep: 0  Superficial: 5 (10) | NR |
|  |  | Unclear | Both | NR | Vicryl | 50 | Deep: 1 (2)  Superficial: 3 (6) | NR |
| Renko 2017 Finland ^[45]^ | Superficial or deep SSI, according to CDC (1992) criteria | Within 30 days post-surgery | Child | All | Triclosan-coated sutures (Vicryl Plus, Monocryl Plus, or PDS Plus | 778 | Superficial: 17 (2)  Deep: 3 (<1) | NR |
|  |  | Within 30 days post-surgery | Child | All | Non-coated sutures (Vicryl, Monocryl, or PDS | 779 | Superficial: 28 (4)  Deep: 14 (2) | NR |
| Sprowson 2018, UK ^[54]^ | Rate of superficial or deep SSI according to Health Protection Agency definitions | 30 days post-surgery | Adult | NR | Vicryl Pus | 1164 | Superficial: 8 (0.7)  Deep: 13 (1.1) | NR |
|  |  | 30 days post-surgery | Adult | NR | Vicryl | 1273 | Superficial: 11 (0.8)  Deep: 21 (1.6) | NR |
| Sukeik 2019, UK ^[56]^ | ASEPSIS (1986) scoring system | Days 2 or 3 after the operation, and again on days 4 or 5 if the patient was still in hospital | Adult | NR | Vicryl Plus | 81 | Score 0-10: 75 (92.6*)  Score >10: 6 (7.4*) | Mean: 2.54 (SD range: 1.41-3.68) |
|  |  | Days 2 or 3 after the operation, and again on days 4 or 5 if the patient was still in hospital | Adult | NR | Vicryl | 69 | Score 0-10: 65 (94.2*)  Score >10: 4 (5.8*) | Mean: 1.41 (SD range: 0.38-2.43) |
| Sundaram 2020a, USA ^[58]^ | Occurrence of SSI (superficial or deep), using definitions adapted from Knee Society consensus (2013), was assessed as part of ‘overall wound complications’ | 90 days post-surgery | Adult | NR | Stratafix Symmetric PDS Plus | 30 | Superficial: 1 (3.33)  Deep: NR | NR |
|  |  | 90 days post-surgery | Adult | NR | Vicryl | 30 | Superficial: 0 (0)  Deep: NR | NR |
| Thimour-Bergström 2013, Sweden ^[8]^ | ASEPSIS score at day 60 postoperatively: leg wounds | 60 days post-surgery | Adult | NR | Vicryl Plus + Monocryl Plus | 184 (treated) | NR | Mean (SD): 3.7 (8.7)  Median (range): 0 (0-45) |
|  |  |  |  |  | Vicryl + Monocryl | 190 (treated) | NR | Mean (SD): 5.4 (10.0)  Median (range): 0 (0-43) |
|  | ASEPSIS score at day 60 postoperatively: sternum wounds | 60 days post-surgery | Adult | NR | Vicryl Plus + Monocryl Plus | 179 (treated) | NR | Mean (SD): 3.3 (8.9)  Median (range): 0 (0-42) |
|  |  |  |  |  | Vicryl + Monocryl | 178 (treated) | NR | Mean (SD): 3.3 (8.5)  Median (range): 0 (0-45) |
| Turtiainen 2012, Finland ^[67]^ | Deep or superficial infection according to CDC definition | 30 days post-surgery | Adult | NR | Vicryl Plus and Monocryl Plus | 139 | Superficial: 24 (77% of all SSIs)  Deep: 5 (16% of all SSIs) | NR |
|  |  | 30 days post-surgery | Adult | NR | Vicryl and Monocryl | 137 | Superficial: 22 (73% of all SSIs)  Deep: 5 (17% of all SSIs) | NR |
| Williams 2011, UK ^[68]^ | ASEPSIS score at 6 weeks post-surgery | 6 weeks post-surgery | Adult | Clean | Vicryl Plus or Monocryl Plus | 66 (completers) | 0: 59 (89.4)  1-9: 1 (1.5)  10+: 6 (9.1) | NR |
|  |  | 6 weeks post-surgery | Adult | Clean | Vicryl or Monocryl | 61 (completers) | 0: 53 (86.9)  1-9: 2 (3.3)  10+: 6 (9.8) | NR |
| Zhang 2011, China ^[69]^ | Mean SSI score on modified ASEPSIS scale | Day 90 | Adult | Clean | Vicryl Plus | 51 | NR | Mean 3.2 (SD 3.6) |
|  |  | Day 90 | Adult | Clean | Chinese silk | 50 | NR | Mean 4.3 (SD 3.3) |

### Additional outcomes added to NICE scope: results

Superficial and deep SSIs

Incidence of superficial and deep SSI were poorly reported with no consistent differences between arms. Three studies ^[20, 45, 48]^ reported a difference between arms for superficial and deep wounds, while the remaining ten studies reporting these data ^[8, 23, 30, 36, 38, 40, 54, 56, 58, 67]^ showed no differences for either wound type.

Wound dehiscence

Nine studies ^[8, 20, 23, 35, 44, 45, 56, 58, 62]^ reported data by arm on incidence of wound dehiscence. Five studies ^[8, 20, 35, 45, 58]^ showed no significant differences between triclosan-coated sutures and uncoated sutures, and another ^[44]^ found a significant reduction in dehiscence with triclosan. The remaining three studies ^[23, 56, 62]^ showed a slight reduction in wound dehiscence incidence with triclosan-coated sutures. However, no significance values were reported.

Pain

Seven studies reported pain by treatment arm ^[23, 28, 36, 45, 48, 51, 56]^. Three found no significant differences between arms ^[23, 45, 56]^, and three reported significant differences, albeit in different directions ^[28, 36, 48]^. The final study ^[51]^ indicated that fewer patients in the triclosan arm experienced pain (no p value presented).

# Additional results of the meta-analyses

## Assessment of heterogeneity

Similarity of the studies was confirmed by quantitative assessment. A Baujat diagnostic plot showed that no study highly influenced the pooled effect size while also highly contributing to the overall heterogeneity of the meta-analysis, and a Leave-One-Out analysis showed that no single study highly influenced heterogeneity or the pooled effect size with I^2^ ranging from 33% to 41% and the pooled effect size ranging from 0.67 to 0.70.

### Supplementary_Figure_3: Baujat diagnostic plot


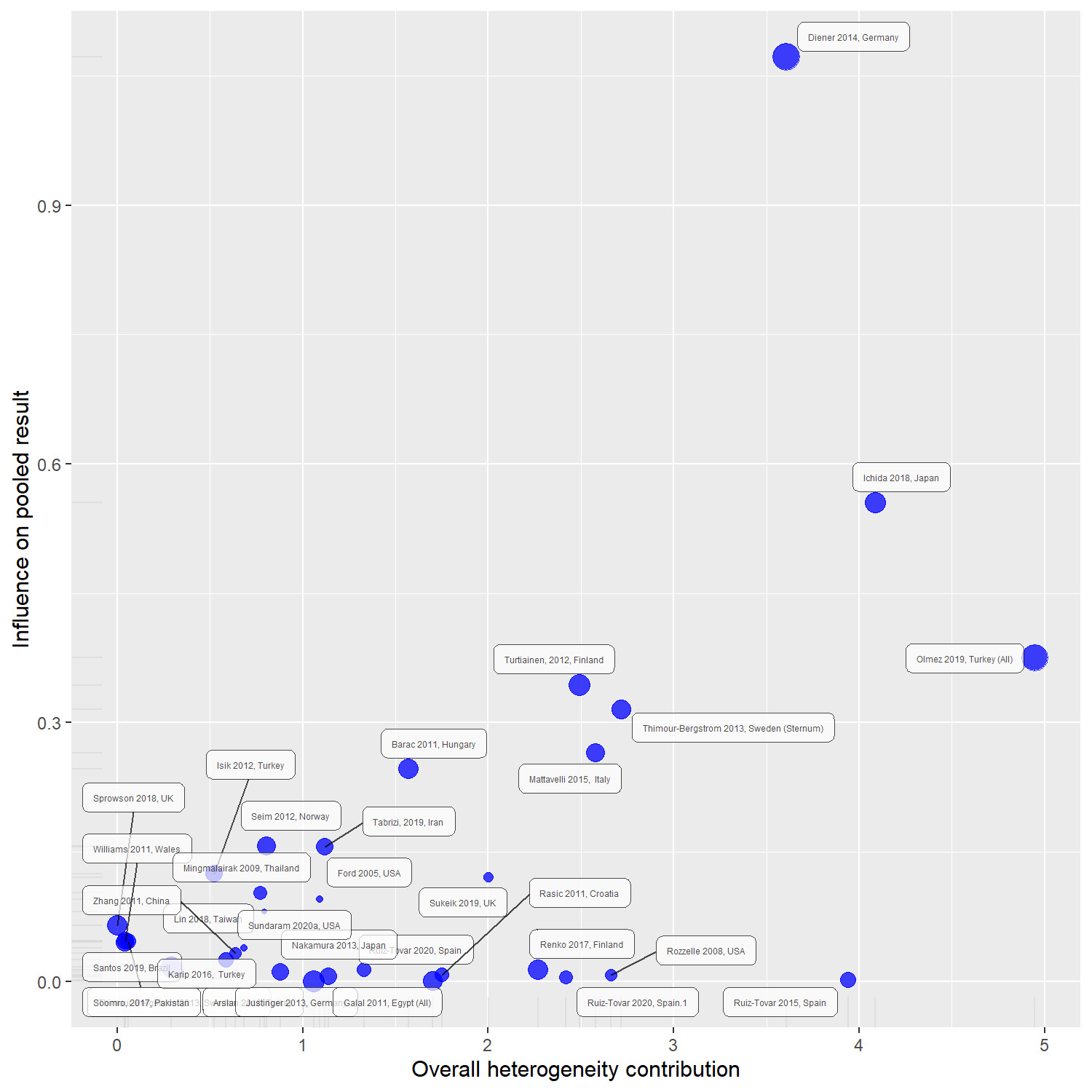


### Supplementary_Figure_4: Leave-One-Out analysis


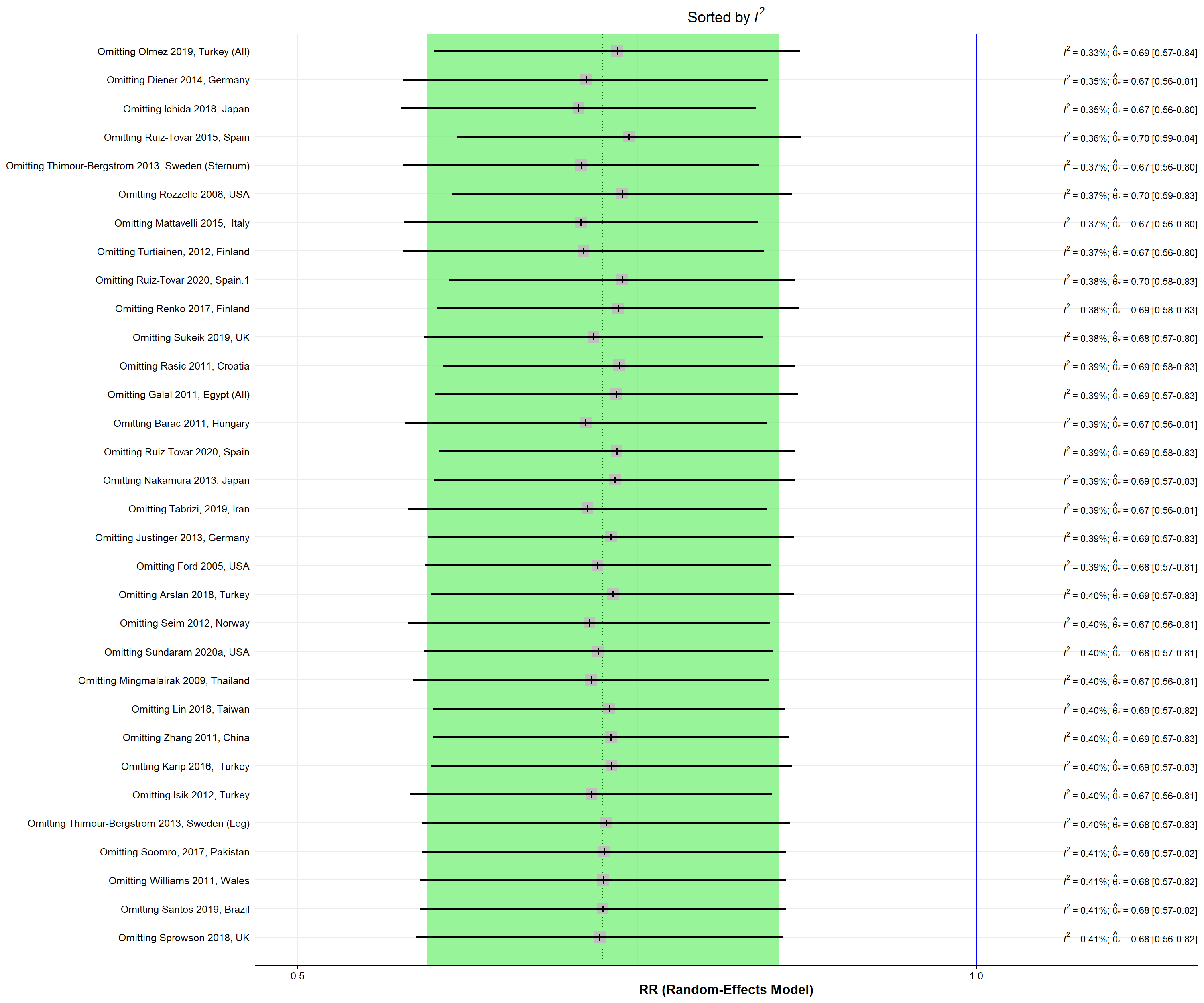


## Assessment of publication bias

No outliers or publication bias were noted during the analysis (see Supplementary Figure 5).

### Supplementary_Figure_5: Publication bias: funnel plot


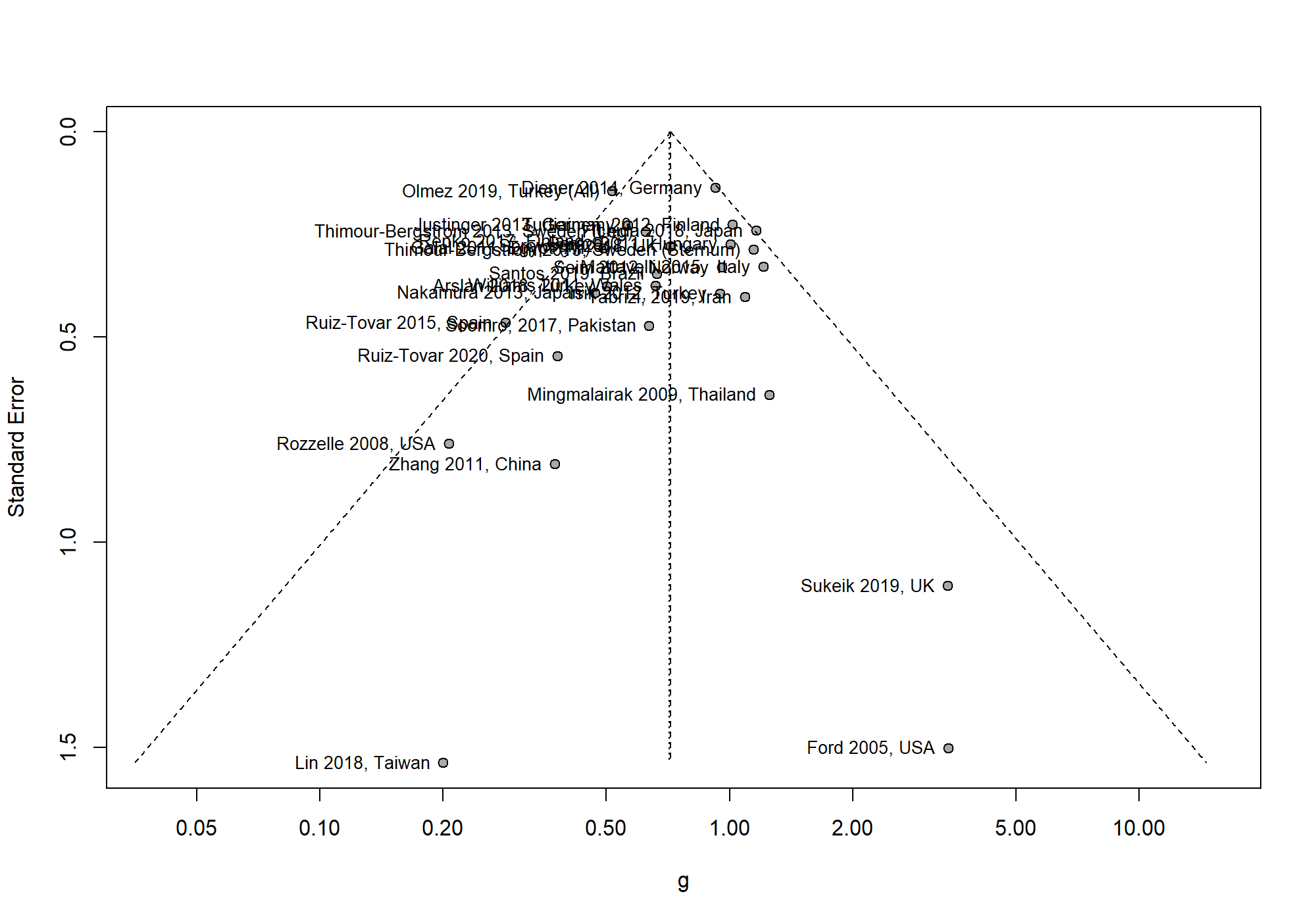


## Subgroups

Results of the meta-analysis of incidence of SSI across the whole population are reported in the main manuscript.

### Adults only

Both the fixed and random effects models produced an estimated risk ratio (RR) less than one (Supplementary Figure 2a). Moreover, in both models the 95% confidence interval does not include one, indicating a statistically significant reduction in the risk of SSI development (p < 0.001 and p = 0.002, respectively).

The fixed effects model estimated a RR of 0.72 (95% confidence interval (CI); 0.64 to 0.81). This indicates those in the Plus Sutures group had a 28% reduction in the risk of developing an SSI compared with those in the control group. The random effects model also estimated a RR of 0.72 (95% CI; 0.60 to 0.88). No outliers or publication bias were noted during the analysis of the available evidence. Results are based on 4,953 and 4,958 total patients and 415 and 568 events in the Plus Sutures and control arm respectively.

### Supplementary_Figure_6a: Meta- analysis results – Adult only SSI incidence studies (with Stratafix)


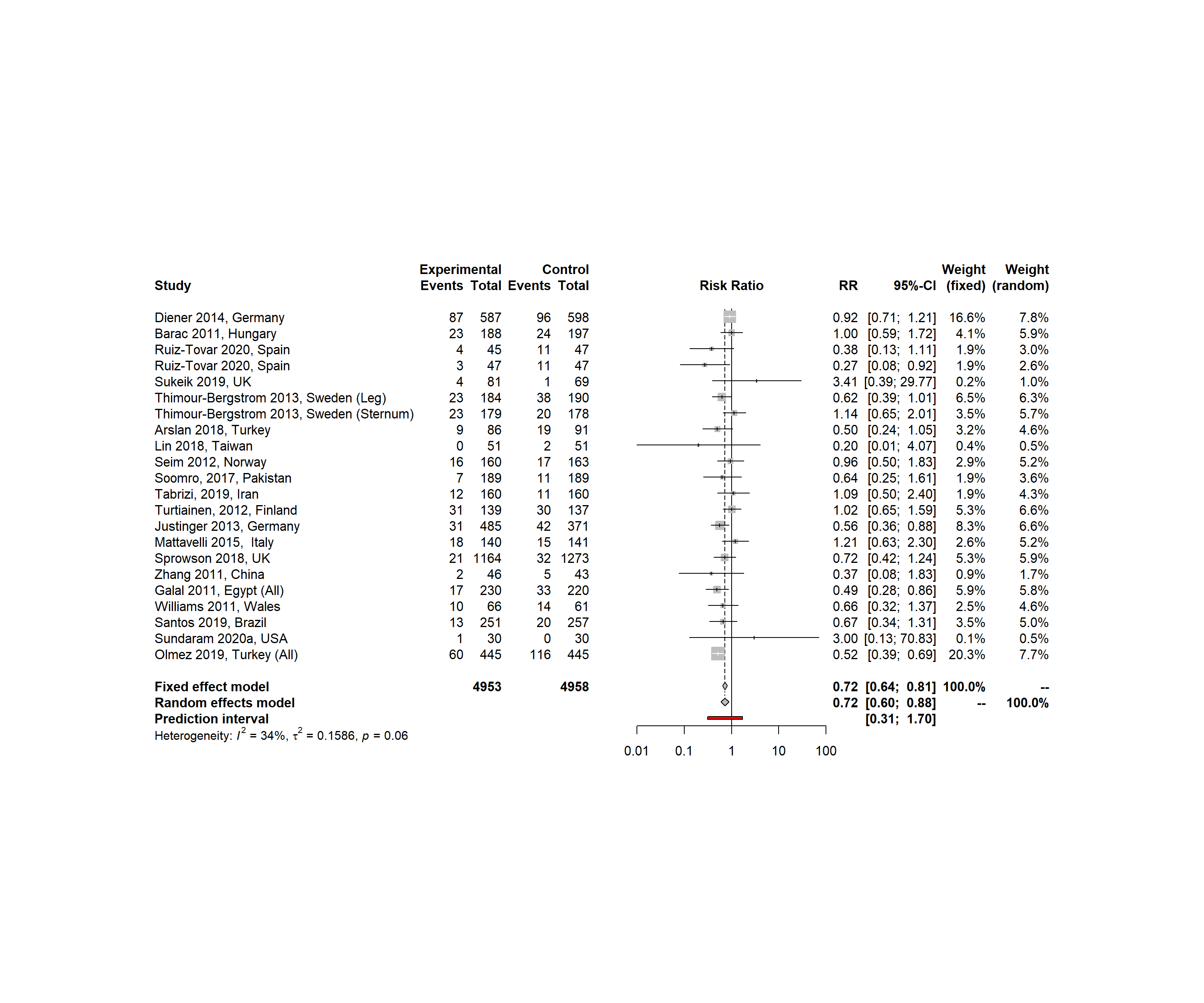


### Children only

Only two studies were conducted in children. Therefore, due to a lack of data, a robust random effects model could not be constructed (i.e. the model resulted in clinically implausible confidence intervals). As a result, only a fixed effects model was performed for the children only subgroup. The fixed effect model produced an estimated RR less than one (Supplementary Figure 2b). Moreover, the 95% confidence interval does not include one, indicating a statistically significant reduction in the risk of SSI development (p = 0.012).

The fixed effect model estimated a RR of 0.52 (95% confidence interval; 0.32 to 0.87). This indicates those in the Plus Sutures group had a 48% reduction in the risk of developing an SSI compared with those in the control group. No outliers or publication bias were noted during the analysis of the available evidence. Results are based on 869 and 823 total patients and 23 and 42 events in the Plus Sutures and control arm respectively.

### Supplementary_Figure_6b: Meta- analysis results – Children only SSI incidence studies (with Stratafix)


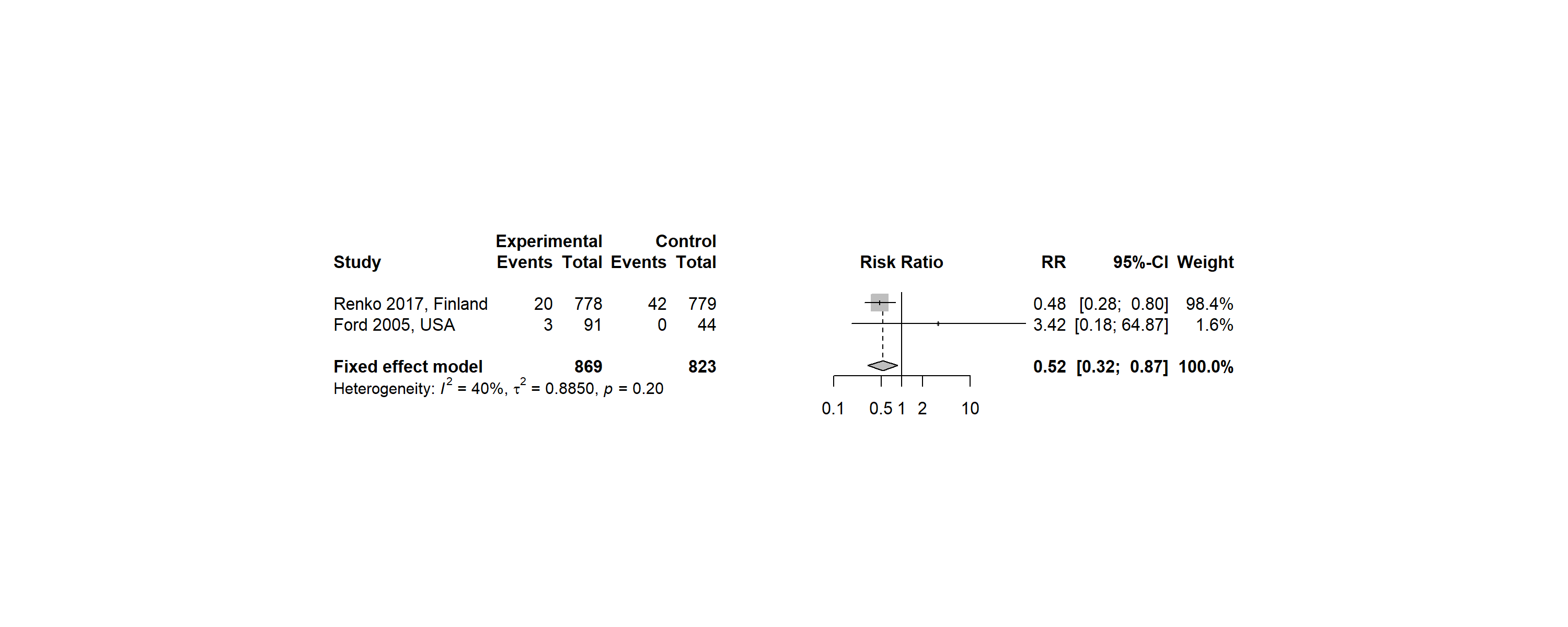


### Clean wounds only

Both the fixed and random effects models produced an estimated RR less than one (Supplementary Figure 2c). Moreover, in both models the 95% confidence interval does not include one, indicating a statistically significant reduction in the risk of SSI development (p = 0.003 and p = 0.037, respectively).

The fixed effects model estimated a RR of 0.75 (95% confidence interval; 0.62 to 0.91). This indicates that those in the Plus Sutures group had a 25% reduction in the risk of developing an SSI compared with those in the control group. The random effects model estimated a RR of 0.72 (95% CI; 0.53 to 0.98). No outliers or publication bias were noted during the analysis of the available evidence. Results are based on 2891 and 3204 total patients and 166 and 243 events in the Plus Sutures and control arm respectively.

### Supplementary_Figure_6c: Meta- analysis results – Clean wound only SSI incidence studies (with Stratafix)


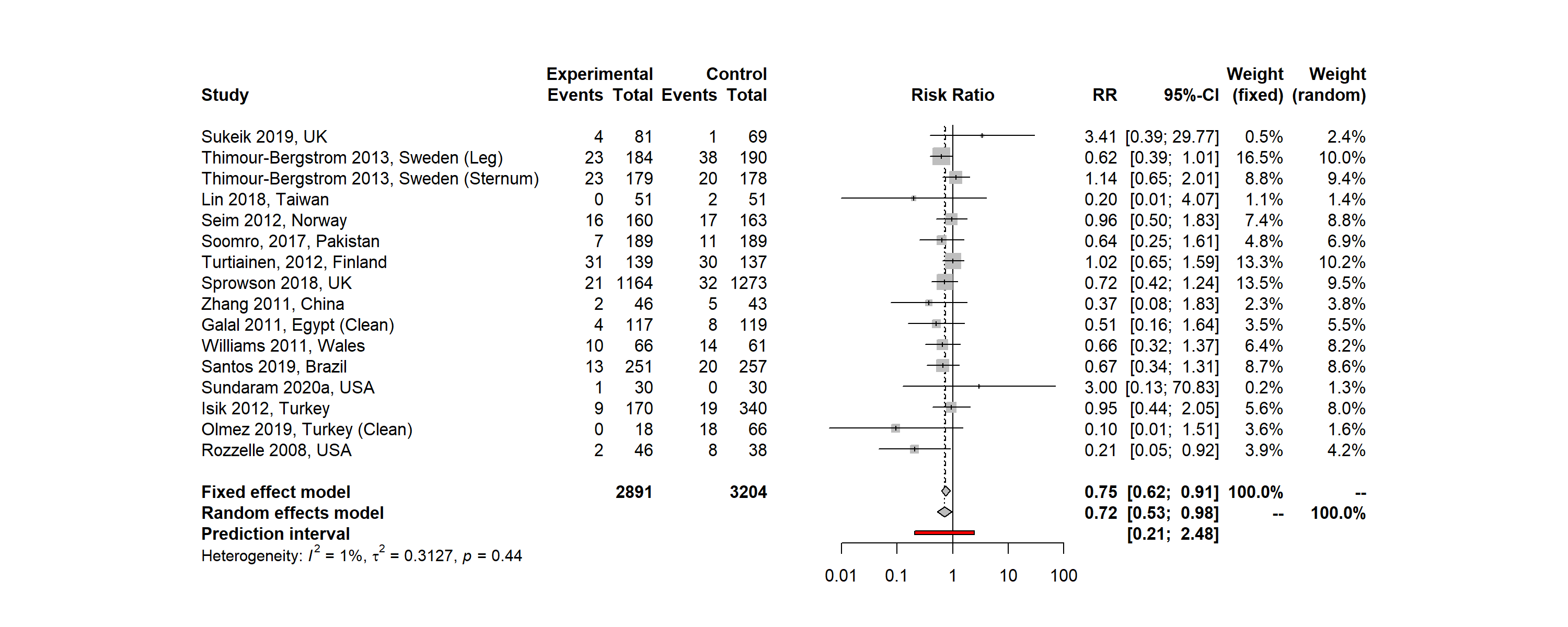


### Non-clean wounds only

Both the fixed and random effects models produced an estimated RR less than one (Supplementary Figure 6d). Moreover, in both models the 95% confidence interval does not include one, indicating a statistically significant reduction in the risk of SSI development (p < 0.001 and p = 0.011 respectively).

The fixed effects model estimated a RR of 0.64 (95% confidence interval; 0.53 to 0.78). This indicates those in the Plus Sutures group had a 36% reduction in the risk of developing an SSI compared with those in the control group. The random effects model also estimated a RR of 0.64 (95% CI; 0.46 to 0.88). No outliers or publication bias were noted during the analysis of the available evidence. Results are based on 1509 and 1426 total patients and 154 and 234 events in the Plus Sutures and control arm respectively.

### Supplementary_Figure_6d: Meta- analysis results – Non-clean wound only SSI incidence studies (with Stratafix)


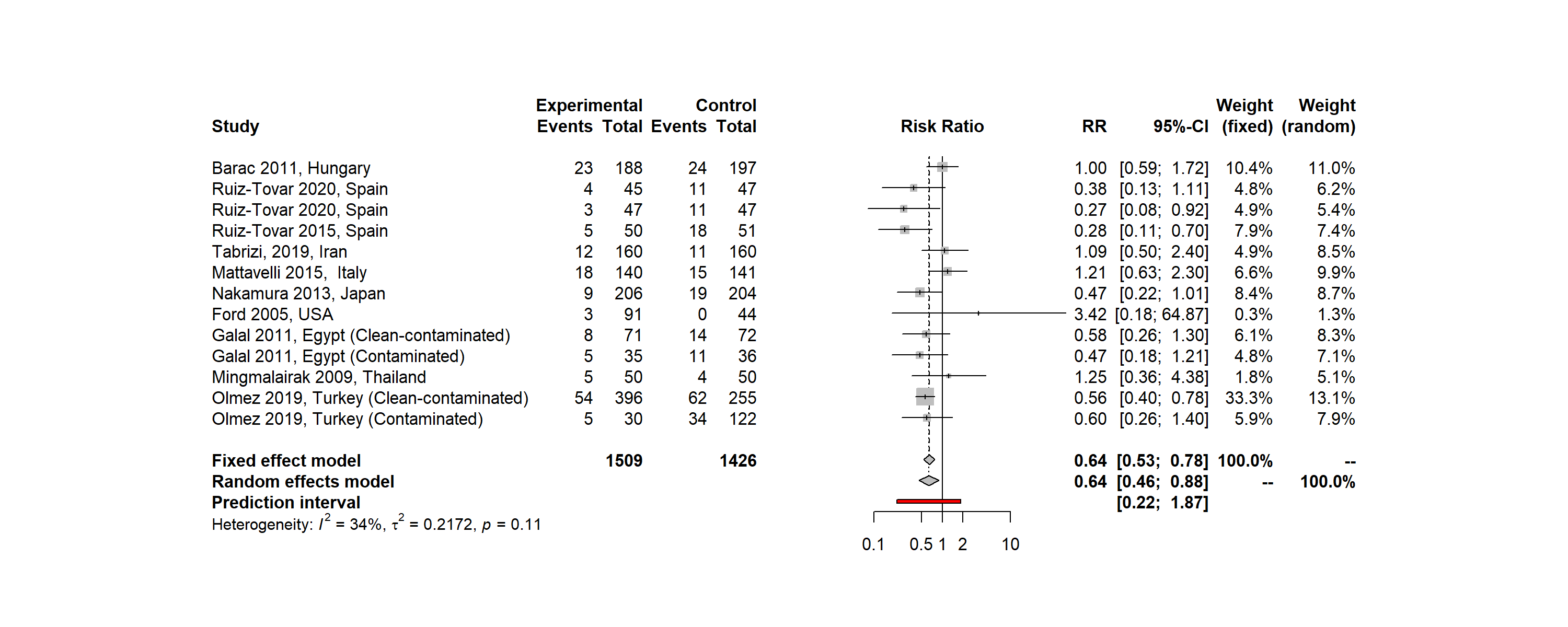


## Results of the sensitivity analysis without Stratafix

Both the fixed and random effect model produced an estimated RR less than one (Supplementary Figure 7). Moreover, in both models the 95% confidence interval does not include one, indicating a statistically significant reduction in the risk of SSI development (p <0.001 and p=0.001, respectively).

The fixed effects model estimated a RR of 0.72 (95% confidence interval; 0.64 to 0.80). This indicates those in the Plus Sutures (including Stratafix Plus) group had a 28% reduction in the risk of developing an SSI compared with those in the control group. The random effects model estimated a RR of 0.71 (95% CI; 0.59 to 0.85). No outliers or publication bias were noted during the analysis of the available evidence. Results are based on 6775 and 6892 total patients and 499 and 697 events in the Plus Sutures and control arm respectively.

### Supplementary_Figure_7: Meta- analysis results – Without Stratafix sensitivity analysis


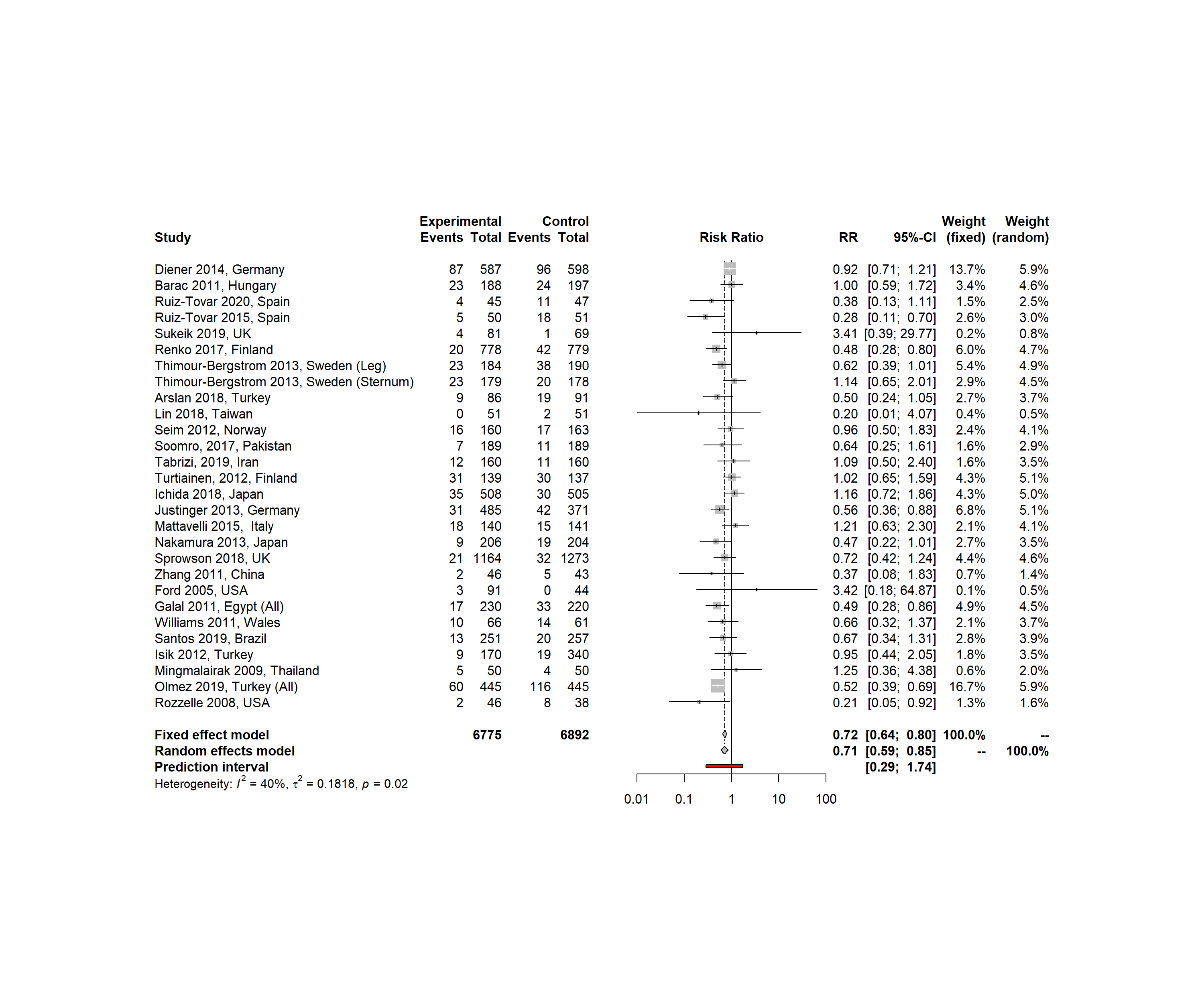


## Results of the meta-analysis including the Falcon trial

Both the fixed and random effect model produced an estimated RR less than one (Supplementary Figure 8). Moreover, in both models the 95% confidence interval does not include one, indicating a statistically significant reduction in the risk of SSI development.

The fixed effects model estimated a RR of 0.82 (95% confidence interval; 0.76 to 0.88). This indicates those in the Plus Sutures (including Stratafix Plus) group had a 18% reduction in the risk of developing an SSI compared with those in the control group. The random effects model estimated a RR of 0.71 (95% CI; 0.60 to 0.85). Results are based on 9492 and 9613 total patients and 1061 and 1298 events in the Plus Sutures and control arm respectively. There was important clinical and methodological heterogeneity, leading to a statistical heterogeneity I² of 50% (Supplementary Figures 9-11), supporting the use of the random effect model over the fixed effect model. No publication bias was identified (Supplementary Figure 12).

### Supplementary_Figure_8: Meta- analysis results – including Falcon trial


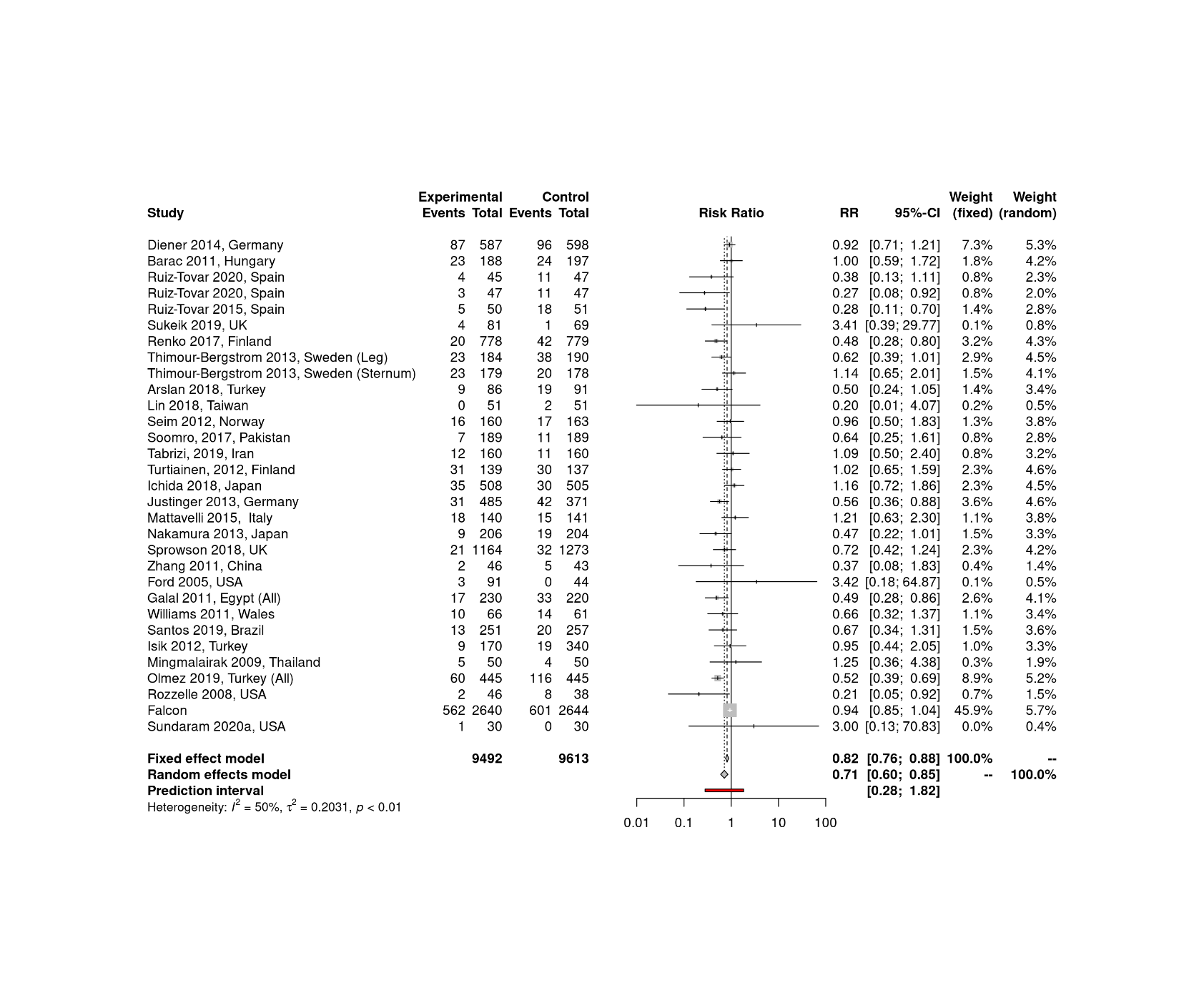


###
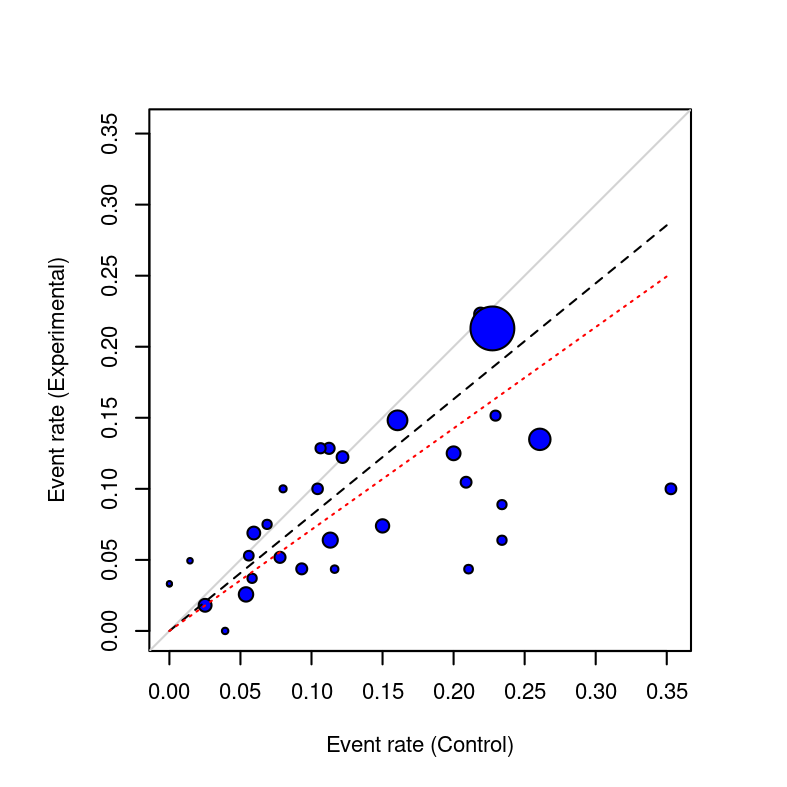
Supplementary_Figure_9: Labbe plot – including Falcon trial

### Supplementary_Figure_10: Baujat plot – including Falcon trial


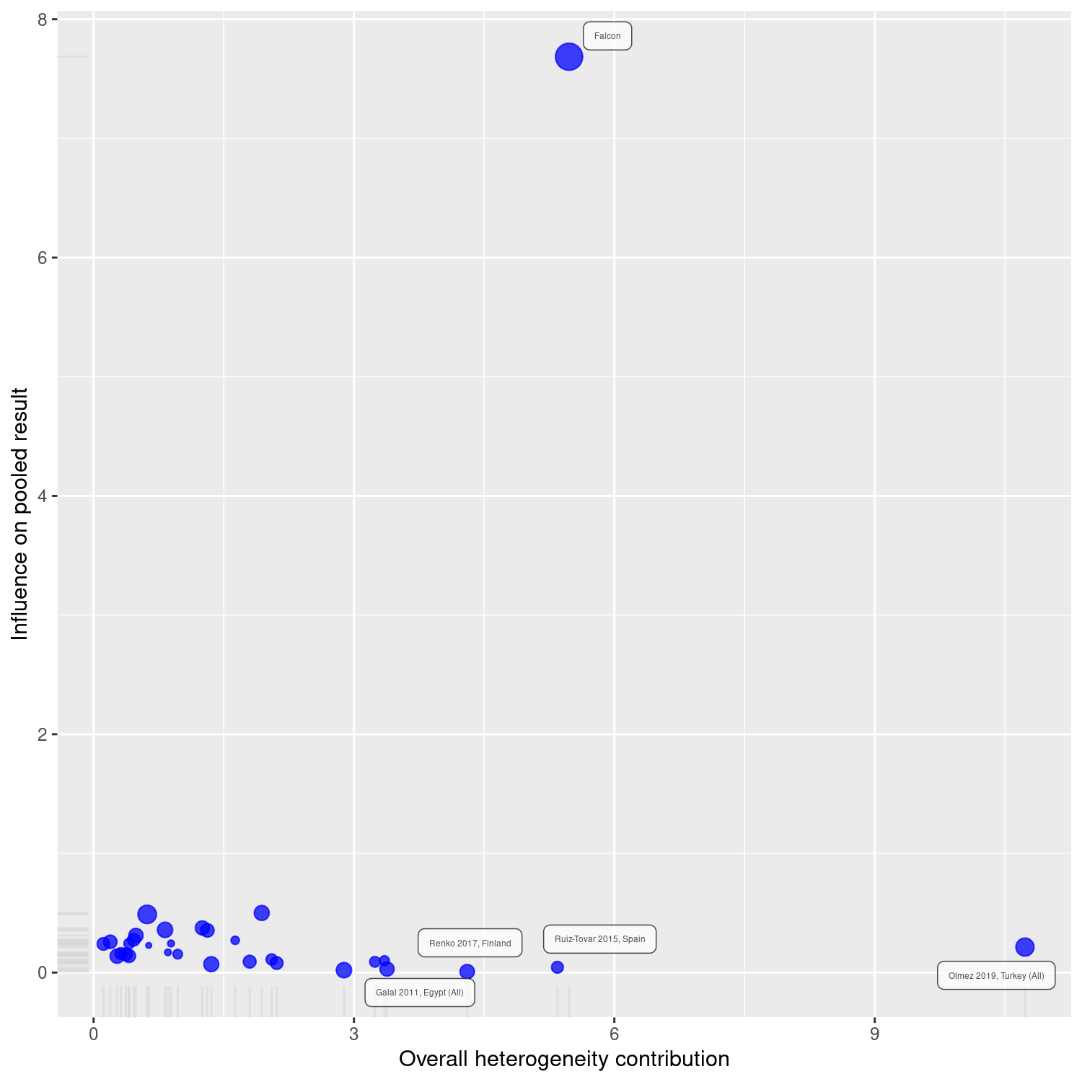


### Supplementary_Figure_11: Left-one-out plot – including Falcon trial


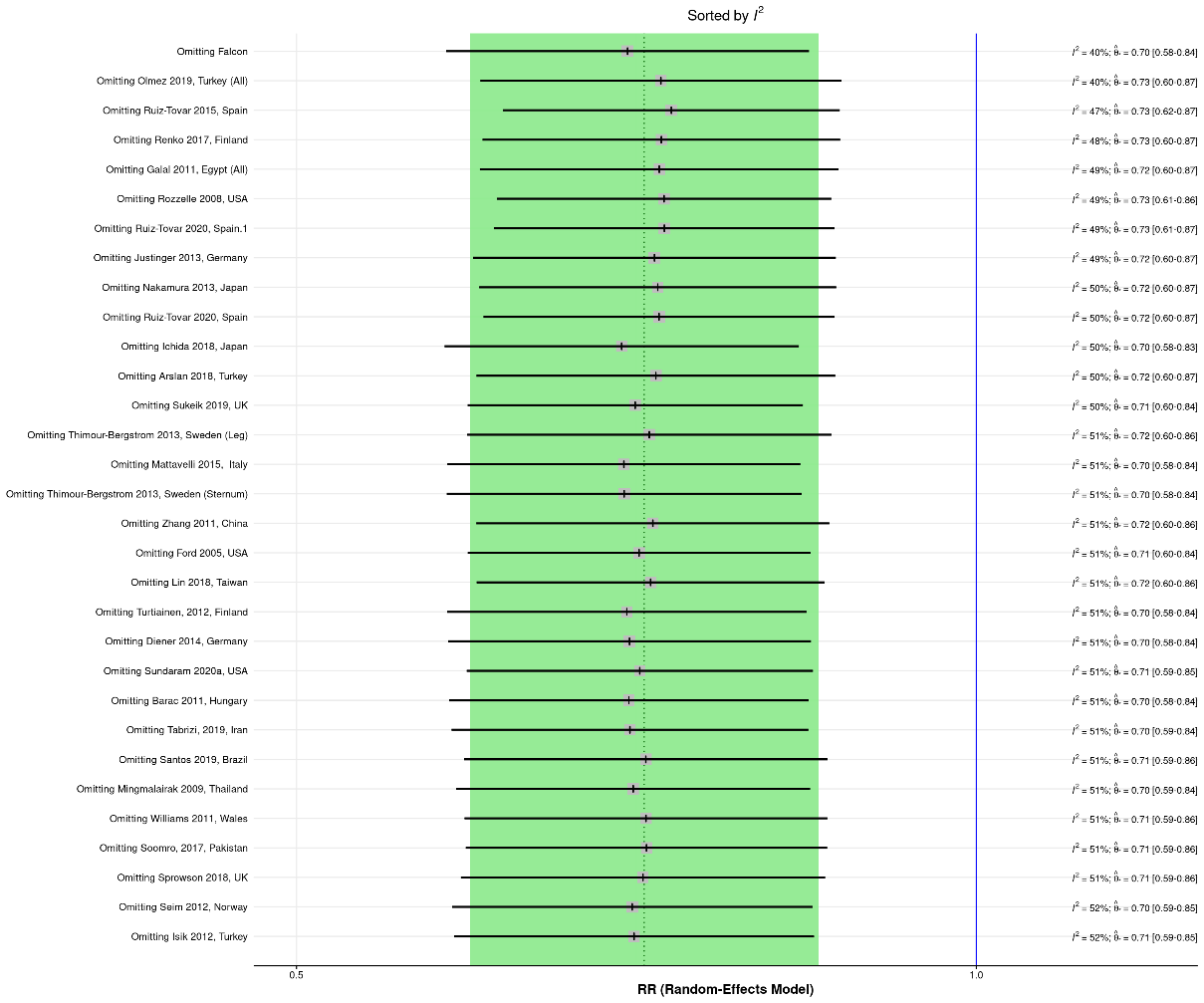


### Supplementary_Figure_12: Funnel plot – including Falcon trial


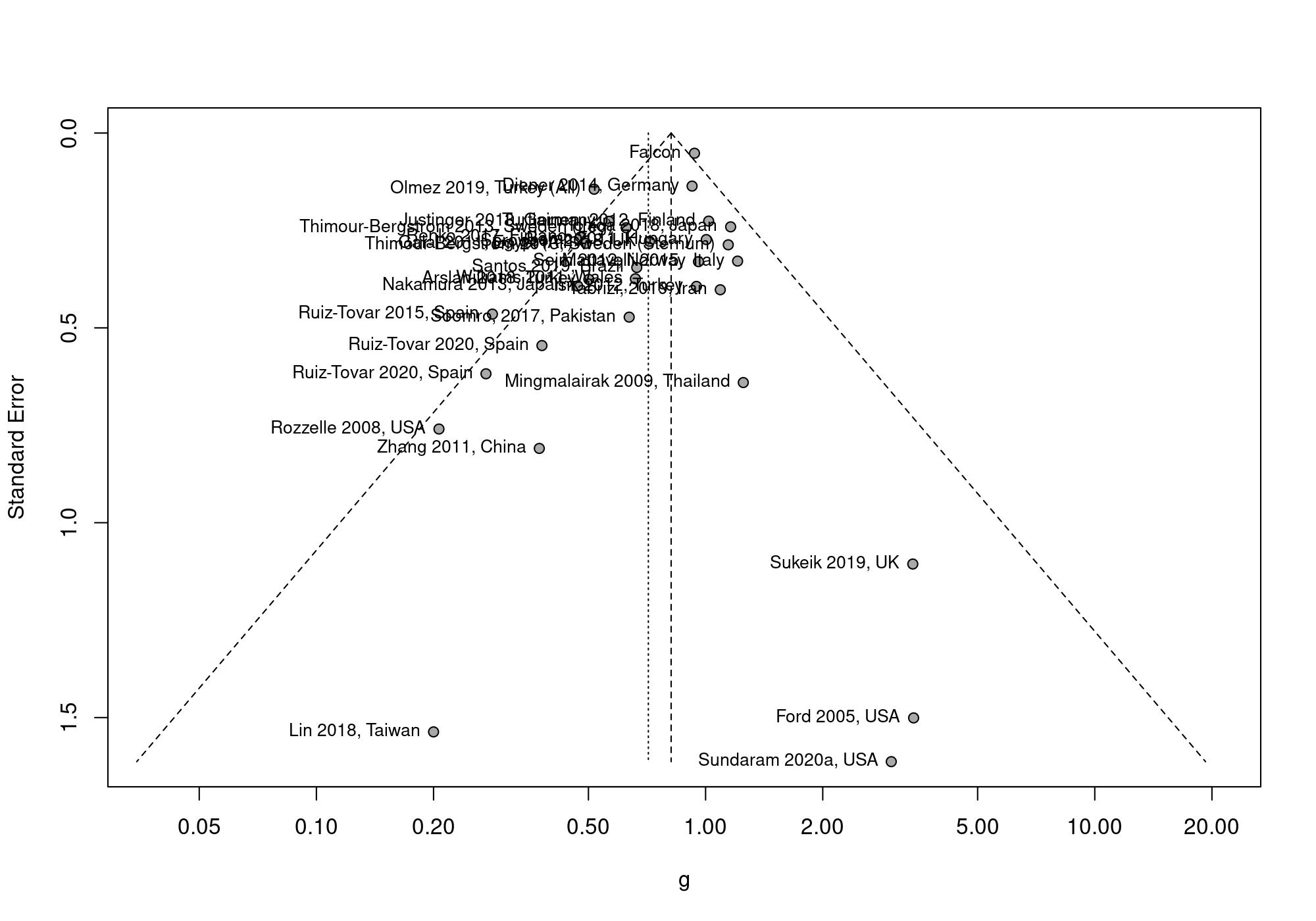


# Additional results of the economic model

### Supplementary_Table_11: Threshold/breakeven analyses results

| **Parameter** | **Base case value** | **Threshold/breakeven value** |
| --- | --- | --- |
| Cost of SSI | £6,016 | £1,493 |
| Baseline risk of SSI with comparator sutures | 1.04% | 0.26% |
| Relative risk reduction with Plus Sutures | 0.71 | 0.93 |
| Average number of sutures per procedure | 5 | 20 |

## Subgroup analyses results

Results for each of the subgroup analyses for adults, children, clean wounds, and non-clean wounds are presented below (Supplementary Tables 12 to 15). As shown, Plus Sutures is estimated to be cost saving in all subgroups.

### Supplementary_Table_12: Subgroup analyses for adults only

|  | **Plus Sutures** | **Comparator sutures*** | **Difference (Plus Sutures minus Comparator)**** |
| --- | --- | --- | --- |
| ***Key model outcomes*** | | | |
| Device cost (Mean cost per patient - £) | £21.25 | £16.75 | £4.50 |
| Cost of SSI treatment (Mean cost per patient - £) | £45.65 | £62.53 | -£16.88 |
| **Total cost per patient** | **£66.90** | **£79.28** | **-£12.38** |
| **Total cost (per 1,000 patients)** | **£66,895** | **£79,278** | **-£12,382** |
| ***Other model outcomes*** | | | |
| Number of SSIs per 1,000 patients | 7.6 | 10.4 | -2.8 |
| **Cost per SSI averted** | | | **Dominant** |
| Number of deaths per 1,000 patients | 13.04 | 13.06 | -0.02 |
| **Cost per death averted** | | | **Dominant** |

* Sutures that do not contain an antibacterial agent

** Negative values indicate a cost saving

*** Dominant = more effective and less costly than the comparator

### Supplementary_Table_13: Subgroup analyses for children only

|  | **Plus Sutures** | **Comparator sutures*** | **Difference (Plus Sutures minus Comparator)**** |
| --- | --- | --- | --- |
| ***Key model outcomes*** | | | |
| Device cost (Mean cost per patient - £) | £21.25 | £16.75 | £4.50 |
| Cost of SSI treatment (Mean cost per patient - £) | £32.51 | £62.53 | -£30.01 |
| **Total cost per patient** | **£53.76** | **£79.28** | **-£25.51** |
| **Total cost (per 1,000 patients)** | **£53,764** | **£79,278** | **-£25,513** |
| ***Other model outcomes*** | | | |
| Number of SSIs per 1,000 patients | 5.4 | 10.4 | -5.0 |
| **Cost per SSI averted** | | | **Dominant** |
| Number of deaths per 1,000 patients | 13.03 | 13.06 | -0.03 |
| **Cost per death averted** | | | **Dominant** |

* Sutures that do not contain an antibacterial agent

** Negative values indicate a cost saving

*** Dominant = more effective and less costly than the comparator

### Supplementary_Table_14: Subgroup analyses for clean wounds only

|  | **Plus Sutures** | **Comparator sutures*** | **Difference (Plus Sutures minus Comparator)**** |
| --- | --- | --- | --- |
| ***Key model outcomes*** | | | |
| Device cost (Mean cost per patient - £) | £21.25 | £16.75 | £4.50 |
| Cost of SSI treatment (Mean cost per patient - £) | £45.12 | £60.16 | -£15.04 |
| **Total cost per patient** | **£66.37** | **£76.91** | **-£10.54** |
| **Total cost (per 1,000 patients)** | **£66,371** | **£76,912** | **-£10,540** |
| ***Other model outcomes*** | | | |
| Number of SSIs per 1,000 patients | 6.0 | 8.0 | -2.0 |
| **Cost per SSI averted** | | | **Dominant** |
| Number of deaths per 1,000 patients | 13.08 | 13.10 | -0.02 |
| **Cost per death averted** | | | **Dominant** |

* Sutures that do not contain an antibacterial agent

** Negative values indicate a cost saving

*** Dominant = more effective and less costly than the comparator

### Supplementary_Table_15: Subgroup analyses for non-clean wounds only

|  | **Plus Sutures** | **Comparator sutures*** | **Difference (Plus Sutures minus Comparator)**** |
| --- | --- | --- | --- |
| ***Key model outcomes*** | | | |
| Device cost (Mean cost per patient - £) | £21.25 | £16.75 | £4.50 |
| Cost of SSI treatment (Mean cost per patient - £) | £281.00 | £425.76 | -£144.76 |
| **Total cost per patient** | **£302.25** | **£442.51** | **-£140.26** |
| **Total cost (per 1,000 patients)** | **£302,249** | **£442,506** | **-£140,257** |
| ***Other model outcomes*** | | | |
| Number of SSIs per 1,000 patients | 45.1 | 68.4 | -23.2 |
| **Cost per SSI averted** | | | **Dominant** |
| Number of deaths per 1,000 patients | 24.56 | 24.58 | -0.02 |
| **Cost per death averted** | | | **Dominant** |

* Sutures that do not contain an antibacterial agent

** Negative values indicate a cost saving

*** Dominant = more effective and less costly than the comparator

# PRISMA 2020 Checklist

| **Section and Topic** | **Item #** | **Checklist item** | **Location where item is reported** |
| --- | --- | --- | --- |
| **TITLE** | | |  |
| Title | 1 | Identify the report as a systematic review. | Title |
| **ABSTRACT** | | |  |
| Abstract | 2 | See the PRISMA 2020 for Abstracts checklist. | “Abstract” |
| **INTRODUCTION** | | |  |
| Rationale | 3 | Describe the rationale for the review in the context of existing knowledge. | “Introduction” |
| Objectives | 4 | Provide an explicit statement of the objective(s) or question(s) the review addresses. | “Methods” and Table 1 |
| **METHODS** | | |  |
| Eligibility criteria | 5 | Specify the inclusion and exclusion criteria for the review and how studies were grouped for the syntheses. | Table 1 and “Subgroup and sensitivity analyses” |
| Information sources | 6 | Specify all databases, registers, websites, organisations, reference lists and other sources searched or consulted to identify studies. Specify the date when each source was last searched or consulted. | Table 2 |
| Search strategy | 7 | Present the full search strategies for all databases, registers and websites, including any filters and limits used. | Supplementary Figure 1 |
| Selection process | 8 | Specify the methods used to decide whether a study met the inclusion criteria of the review, including how many reviewers screened each record and each report retrieved, whether they worked independently, and if applicable, details of automation tools used in the process. | “Systematic review methods” |
| Data collection process | 9 | Specify the methods used to collect data from reports, including how many reviewers collected data from each report, whether they worked independently, any processes for obtaining or confirming data from study investigators, and if applicable, details of automation tools used in the process. | “Systematic review methods” |
| Data items | 10a | List and define all outcomes for which data were sought. Specify whether all results that were compatible with each outcome domain in each study were sought (e.g. for all measures, time points, analyses), and if not, the methods used to decide which results to collect. | Review protocol; see Open Science Foundation database |
|  | 10b | List and define all other variables for which data were sought (e.g. participant and intervention characteristics, funding sources). Describe any assumptions made about any missing or unclear information. | Supplementary Tables 4 and 5 |
| Study risk of bias assessment | 11 | Specify the methods used to assess risk of bias in the included studies, including details of the tool(s) used, how many reviewers assessed each study and whether they worked independently, and if applicable, details of automation tools used in the process. | “Screening, selection and data extraction” |
| Effect measures | 12 | Specify for each outcome the effect measure(s) (e.g. risk ratio, mean difference) used in the synthesis or presentation of results. | “Meta-analysis methods” |
| Synthesis methods | 13a | Describe the processes used to decide which studies were eligible for each synthesis (e.g. tabulating the study intervention characteristics and comparing against the planned groups for each synthesis (item #5)). | “Systematic review methods: Synthesis” |
|  | 13b | Describe any methods required to prepare the data for presentation or synthesis, such as handling of missing summary statistics, or data conversions. | No missing data were imputed; no data conversions were performed |
|  | 13c | Describe any methods used to tabulate or visually display results of individual studies and syntheses. | “Meta-analysis methods” |
|  | 13d | Describe any methods used to synthesize results and provide a rationale for the choice(s). If meta-analysis was performed, describe the model(s), method(s) to identify the presence and extent of statistical heterogeneity, and software package(s) used. | “Meta-analysis methods” |
|  | 13e | Describe any methods used to explore possible causes of heterogeneity among study results (e.g. subgroup analysis, meta-regression). | “Meta-analysis methods” |
|  | 13f | Describe any sensitivity analyses conducted to assess robustness of the synthesized results. | “Subgroup and sensitivity analyses” |
| Reporting bias assessment | 14 | Describe any methods used to assess risk of bias due to missing results in a synthesis (arising from reporting biases). | Supplementary section “Meta-analysis methods” |
| Certainty assessment | 15 | Describe any methods used to assess certainty (or confidence) in the body of evidence for an outcome. | “Meta-analysis methods”, “Results of the meta-analysis” and Supplementary section “Meta-analysis methods” |
| **RESULTS** | | |  |
| Study selection | 16a | Describe the results of the search and selection process, from the number of records identified in the search to the number of studies included in the review, ideally using a flow diagram. | “Results of the systematic review” |
|  | 16b | Cite studies that might appear to meet the inclusion criteria, but which were excluded, and explain why they were excluded. | Supplementary Table 2 |
| Study characteristics | 17 | Cite each included study and present its characteristics. | Supplementary Tables 4 and 5 |
| Risk of bias in studies | 18 | Present assessments of risk of bias for each included study. | Supplementary Table 6 |
| Results of individual studies | 19 | For all outcomes, present, for each study: (a) summary statistics for each group (where appropriate) and (b) an effect estimate and its precision (e.g. confidence/credible interval), ideally using structured tables or plots. | Figure 3 and Supplementary Figures 3a-d and 4  See also “Results of the qualitative analyses” |
| Results of syntheses | 20a | For each synthesis, briefly summarise the characteristics and risk of bias among contributing studies. | Assessed quantitatively; see “Results of the meta-analyses” |
|  | 20b | Present results of all statistical syntheses conducted. If meta-analysis was done, present for each the summary estimate and its precision (e.g. confidence/credible interval) and measures of statistical heterogeneity. If comparing groups, describe the direction of the effect. | “Results of the meta-analyses” |
|  | 20c | Present results of all investigations of possible causes of heterogeneity among study results. | “Results of the meta-analyses” |
|  | 20d | Present results of all sensitivity analyses conducted to assess the robustness of the synthesized results. | Supplementary Section 6 |
| Reporting biases | 21 | Present assessments of risk of bias due to missing results (arising from reporting biases) for each synthesis assessed. | Supplementary Table 6 |
| Certainty of evidence | 22 | Present assessments of certainty (or confidence) in the body of evidence for each outcome assessed. | “Results of the meta-analysis”; see presented p values and confidence intervals |
| **DISCUSSION** | | |  |
| Discussion | 23a | Provide a general interpretation of the results in the context of other evidence. | “Clinical evidence” |
|  | 23b | Discuss any limitations of the evidence included in the review. | “Strengths and limitations of the clinical evidence” |
|  | 23c | Discuss any limitations of the review processes used. | “Strengths and limitations of the clinical evidence” |
|  | 23d | Discuss implications of the results for practice, policy, and future research. | “Conclusions” |
| **OTHER INFORMATION** | | |  |
| Registration and protocol | 24a | Provide registration information for the review, including register name and registration number, or state that the review was not registered. | “Systematic review methods” |
|  | 24b | Indicate where the review protocol can be accessed, or state that a protocol was not prepared. | “Systematic review methods” |
|  | 24c | Describe and explain any amendments to information provided at registration or in the protocol. | NA |
| Support | 25 | Describe sources of financial or non-financial support for the review, and the role of the funders or sponsors in the review. | “Funding and Other Information” |
| Competing interests | 26 | Declare any competing interests of review authors. | “Funding and Other Information” |
| Availability of data, code and other materials | 27 | Report which of the following are publicly available and where they can be found: template data collection forms; data extracted from included studies; data used for all analyses; analytic code; any other materials used in the review. | Not publicly available |

## References for Supplementary Material

*Please note that reference numbers for individual papers throughout this document differ from those used in the main manuscript*

1. Mantel N, Haenszel W. Statistical aspects of the analysis of data from retrospective studies of disease. J Natl Cancer Inst. 1959;22(4):719-48.

2. Robins J, Greenland S, Breslow NE. A general estimator for the variance of the Mantel-Haenszel odds ratio. Am J Epidemiol. 1986;124(5):719-23.

3. Sidik K, Jonkman JN. A comparison of heterogeneity variance estimators in combining results of studies. Stat Med. 2007;26(9):1964-81.

4. IntHout J, Ioannidis JP, Borm GF. The Hartung-Knapp-Sidik-Jonkman method for random effects meta-analysis is straightforward and considerably outperforms the standard DerSimonian-Laird method. BMC Med Res Methodol. 2014; 14: 25. Available from: <https://bmcmedresmethodol.biomedcentral.com/articles/10.1186/1471-2288-14-25>

5. IntHout J, Ioannidis JP, Rovers MM, Goeman JJ. Plea for routinely presenting prediction intervals in meta-analysis. BMJ Open. 2016;6(7):e010247.

6. Egger M, Davey Smith G, Schneider M, Minder C. Bias in meta-analysis detected by a simple, graphical test. BMJ. 1997;315(7109):629-34.

7. Baujat B, Mahe C, Pignon JP, Hill C. A graphical method for exploring heterogeneity in meta-analyses: application to a meta-analysis of 65 trials. Stat Med. 2002;21(18):2641-52.

8. Thimour-Bergstrom L, Roman-Emanuel C, Schersten H, Friberg O, Gudbjartsson T, Jeppsson A. Triclosan-coated sutures reduce surgical site infection after open vein harvesting in coronary artery bypass grafting patients: a randomized controlled trial. Eur J Cardiothorac Surg. 2013;44(5):931-8.

9. Steingrimsson S, Thimour-Bergstrom L, Roman-Emanuel C, Schersten H, Friberg O, Gudbjartsson T*, et al.* Triclosan-coated sutures and sternal wound infections: a prospective randomized clinical trial. Eur J Clin Microbiol Infect Dis. 2015;34(12):2331-8.

10. Public Health England. Surveillance of surgical site infections in NHS hospitals in England. England: 2020. Available from: <https://assets.publishing.service.gov.uk/government/uploads/system/uploads/attachment_data/file/945712/SSI_Annual_Report_2019_20.pdf>.

11. Troughton R, Birgand G, Johnson AP, Naylor N, Gharbi M, Aylin P*, et al.* Mapping national surveillance of surgical site infections in England: needs and priorities. J Hosp Infect. 2018;100(4):378-85.

12. National Institute for Health and Care Excellence. Surgical site infections: prevention and treatment. Health economic model report. England: 2019. Available from: <https://www.nice.org.uk/guidance/ng125/evidence/health-economic-model-report-pdf-6727106989>.

13. Public Health England. Surveillance of surgical site infections in NHS hospitals in England. England: 2017. Available from: <https://assets.publishing.service.gov.uk/government/uploads/system/uploads/attachment_data/file/666465/SSI_annual_report_NHS_hospitals_2016-17.pdf>.

14. Jenks PJ, Laurent M, McQuarry S, Watkins R. Clinical and economic burden of surgical site infection (SSI) and predicted financial consequences of elimination of SSI from an English hospital. J Hosp Infect. 2014;86(1):24-33.

15. National Institute for Health and Care Excellence (NICE). Plus Sutures for preventing surgical site infection. Medical technologies guidance [MTG59] [Webpage]. London & Manchester: NICE; 2021. [cited August 2021]. Available from: <https://www.nice.org.uk/guidance/mtg59>.

16. De Jonge SW, Atema JJ, Solomkin JS, Boermeester MA. Meta-analysis and trial sequential analysis of triclosan-coated sutures for the prevention of surgical-site infection. Br J Surg. 2017;104(2):e118-e33.

17. Coalition for Sustainable Pharmaceuticals and Medical Devices. Care pathways: Guidance on Appraising Sustainability. Cambridge: NHS Sustainable Development Unit; 2015. [cited February 2021]. Available from: <https://www.sduhealth.org.uk/areas-of-focus/carbon-hotspots/pharmaceuticals/cspm/sustainable-care-pathways-guidance.aspx>.

18. Shepard J, Ward W, Milstone A, Carlson T, Frederick J, Hadhazy E*, et al.* Financial impact of surgical site infections on hospitals: the hospital management perspective. JAMA Surg. 2013;148(10):907-14.

19. Perencevich EN, Sands KE, Cosgrove SE, Guadagnoli E, Meara E, Platt R. Health and economic impact of surgical site infections diagnosed after hospital discharge. Emerg Infect Dis. 2003;9(2):196-203.

20. Arslan NC, Atasoy G, Altintas T, Terzi C. Effect of triclosan-coated sutures on surgical site infections in pilonidal disease: prospective randomized study. Int J Colorectal Dis. 2018;33(10):1445-52.

21. Baracs J, Huszar O, Sajjadi SG, Horvath OP. Surgical site infections after abdominal closure in colorectal surgery using triclosan-coated absorbable suture (PDS Plus) vs. uncoated sutures (PDS II): a randomized multicenter study. Surg Infect (Larchmt). 2011;12(6):483-9.

22. University of Pecs. Abdominal Wall Closure With Triclosan-coated Suture (TCS09-10). Identifier: NCT01123616. In: ClinicalTrials.gov [internet]. Bethesda: US National Library of Medicine: 2010. Available from <https://clinicaltrials.gov/show/NCT01123616>.

23. Diener MK, Knebel P, Kieser M, Schuler P, Schiergens TS, Atanassov V*, et al.* Effectiveness of triclosan-coated PDS Plus versus uncoated PDS II sutures for prevention of surgical site infection after abdominal wall closure: the randomised controlled PROUD trial. Lancet. 2014;384(9938):142-52.

24. Heger U, Voss S, Knebel P, Doerr-Harim C, Neudecker J, Schuhmacher C*, et al.* Prevention of abdominal wound infection (PROUD trial, DRKS00000390): study protocol for a randomized controlled trial. Trials. 2011; 12: 245. Available from: <https://trialsjournal.biomedcentral.com/articles/10.1186/1745-6215-12-245>

25. Universitätsklinik Heidelberg. Prevention of abdominal wound infection. Identifier: DRKS00000390. In: German Clinical Trials Register [internet]. Freiburg: Institute for Medical Biometry and Statistics - University of Freiburg: 2010. Available from <http://www.drks.de/DRKS00000390>.

26. Diener MK, Knebel P, Kieser M, Probst P, Buchler MW. Antibiotic sutures against surgical site infections - Authors' reply. The Lancet. 2014;384(9952):1425-26.

27. Fujita T. Correspondence: Antibiotic sutures against surgical site infections. Lancet. 2014;384(9952):1424-25.

28. Ford HR, Jones P, Gaines B, Reblock K, Simpkins DL. Intraoperative handling and wound healing: controlled clinical trial comparing coated VICRYL plus antibacterial suture (coated polyglactin 910 suture with triclosan) with coated VICRYL suture (coated polyglactin 910 suture). Surg Infect (Larchmt). 2005;6(3):313-21.

29. Galal I, El-Hindawy K. Impact of using triclosan-antibacterial sutures on incidence of surgical site infection. Am J Surg. 2011;202(2):133-8.

30. Ichida K, Noda H, Kikugawa R, Hasegawa F, Obitsu T, Ishioka D*, et al.* Effect of triclosan-coated sutures on the incidence of surgical site infection after abdominal wall closure in gastroenterological surgery: a double-blind, randomized controlled trial in a single center. Surgery. 2018;164(1):91-95.

31. Department of Surgery Saitama Medical Center Jichi Medical University. Study of the efficacy of antibacterial suture for reducing the surgical site infection. Identifier: JPRN-UMIN000013054. In: UMIN Clinical Trials Registry [internet]. Tokyo: University of Tokyo Hospital: 2014. Available from <https://upload.umin.ac.jp/cgi-open-bin/ctr_e/ctr_view.cgi?recptno=R000015230>.

32. Isik I, Selimen D, Senay S, Alhan C. Efficiency of antibacterial suture material in cardiac surgery: a double-blind randomized prospective study. Heart Surg Forum. 2012;15(1):E40-45.

33. Justinger C, Slotta JE, Ningel S, Graber S, Kollmar O, Schilling MK. Surgical-site infection after abdominal wall closure with triclosan-impregnated polydioxanone sutures: results of a randomized clinical pathway facilitated trial (NCT00998907). Surgery. 2013;154(3):589-95.

34. University Hospital S. PDS*Plus and Wound Infections After Laparotomy. Identifier: NCT00998907. In: ClinicalTrials.gov [internet]. Bethesda: US National Library of Medicine: 2009. Available from <https://clinicaltrials.gov/show/NCT00998907>.

35. Karip AB, Celik K, Aydin T, Yazicilar H, Iscan Y, Agalar C*, et al.* Effect of Triclosan-Coated Suture and Antibiotic Prophylaxis on Infection and Recurrence after Karydakis Flap Repair for Pilonidal Disease: A Randomized Parallel-Arm Double-Blinded Clinical Trial. Surg Infect (Larchmt). 2016;17(5):583-8.

36. Lin S-J, Chang F-C, Huang T-W, Peng K-T, Shih HN, Lee MS. Temporal Change of Interleukin-6, C-Reactive Protein, and Skin Temperature after Total Knee Arthroplasty Using Triclosan-Coated Sutures. Biomed Res Int. 2018: 9136208. Available from: <https://www.hindawi.com/journals/bmri/2018/9136208/>

37. Mel Shiuann-Sheng Lee. Compare Antimicrobial to Conventional Suture in Patients Receiving Primary Total Knee Replacement. Identifier: NCT02533492. In: ClinicalTrials.gov [internet]. Bethesda: US National Library of Medicine: 2015. Available from <https://clinicaltrials.gov/show/NCT02533492>.

38. Mattavelli I, Rebora P, Doglietto G, Dionigi P, Dominioni L, Luperto M*, et al.* Multi-Center Randomized Controlled Trial on the Effect of Triclosan-Coated Sutures on Surgical Site Infection after Colorectal Surgery. Surg Infect (Larchmt). 2015;16(3):226-35.

39. University of Milano Bicocca. Impact of Triclosan-coated Suture on Surgical Site Infection After Colorectal Surgery. Identifier: NCT01869257. In: ClinicalTrials.gov [internet]. Bethesda: US National Library of Medicine: 2013. Available from <https://clinicaltrials.gov/show/NCT01869257>.

40. Mingmalairak C, Ungbhakorn P, Paocharoen V. Efficacy of antimicrobial coating suture coated polyglactin 910 with tricosan (Vicryl plus) compared with polyglactin 910 (Vicryl) in reduced surgical site infection of appendicitis, double blind randomized control trial, preliminary safety report. J Med Assoc Thai. 2009;92(6):770-5.

41. Nakamura T, Kashimura N, Noji T, Suzuki O, Ambo Y, Nakamura F*, et al.* Triclosan-coated sutures reduce the incidence of wound infections and the costs after colorectal surgery: a randomized controlled trial. Surgery. 2013;153(4):576-83.

42. Teine Keijinkai Hospital. Triclosan Coated Sutures for the Reduction of Abdominal Wound Infections and Economic Considerations : single institutional prospective randomized control trial. Identifier: JPRN-UMIN000003322. In: UMIN Clinical Trials Registry [internet]. Tokyo: University of Tokyo Hospital: 2010. Available from <https://upload.umin.ac.jp/cgi-open-bin/ctr_e/ctr_view.cgi?recptno=R000004032>.

43. Olmez T, Berkesoglu M, Turkmenoglu O, Colak T. Effect of Triclosan-Coated Suture on Surgical Site Infection of Abdominal Fascial Closures. Surg Infect (Larchmt). 2019;20(8):658-64.

44. Rasic Z, Schwarz D, Adam VN, Sever M, Lojo N, Rasic D*, et al.* Efficacy of antimicrobial triclosan-coated polyglactin 910 (Vicryl* Plus) suture for closure of the abdominal wall after colorectal surgery. Coll Antropol. 2011;35(2):439-43.

45. Renko M, Paalanne N, Tapiainen T, Hinkkainen M, Pokka T, Kinnula S*, et al.* Triclosan-containing sutures versus ordinary sutures for reducing surgical site infections in children: a double-blind, randomised controlled trial. Lancet Infect Dis. 2017;17(1):50-57.

46. University of Oulu. Antimicrobial Coated Sutures in Paediatric Surgery. Identifier: NCT01220700. In: ClinicalTrials.gov [internet]. Bethesda: US National Library of Medicine: 2010. Available from <https://clinicaltrials.gov/show/NCT01220700>.

47. Rozzelle CJ, Leonardo J, Li V. Antimicrobial suture wound closure for cerebrospinal fluid shunt surgery: a prospective, double-blinded, randomized controlled trial. J Neurosurg Pediatrics. 2008;2(2):111-7.

48. Ruiz-Tovar J, Llavero C, Jimenez-Fuertes M, Duran M, Perez-Lopez M, Garcia-Marin A. Incisional Surgical Site Infection after Abdominal Fascial Closure with Triclosan-Coated Barbed Suture vs Triclosan-Coated Polydioxanone Loop Suture vs Polydioxanone Loop Suture in Emergent Abdominal Surgery: A Randomized Clinical Trial. J Am Coll Surg. 2020;230(5):766-74.

49. Hospital General Universitario Elche. Effect of Barbed Suture and Triclosan-coated Monofilament in Emergency Surgery. Identifier: NCT03763279. In: ClinicalTrials.gov [internet]. Bethesda: US National Library of Medicine: 2018. Available from <https://clinicaltrials.gov/show/NCT03763279>.

50. Ruiz-Tovar J, Alonso N, Morales V, Llavero C. Association between Triclosan-Coated Sutures for Abdominal Wall Closure and Incisional Surgical Site Infection after Open Surgery in Patients Presenting with Fecal Peritonitis: A Randomized Clinical Trial. Surg Infect (Larchmt). 2015;16(5):588-94.

51. Santos PSF, Santos M, Colafranceschi AS, Pragana ANdS, Correia MG, Simoes HH*, et al.* Effect of Using Triclosan-Impregnated Polyglactin Suture to Prevent Infection of Saphenectomy Wounds in CABG: A Prospective, Double-Blind, Randomized Clinical Trial. Braz. 2019;34(5):588-95.

52. Seim BE, Tonnessen T, Woldbaek PR. Triclosan-coated sutures do not reduce leg wound infections after coronary artery bypass grafting. Interactive Cardiovascular & Thoracic Surgery. 2012;15(3):411-5.

53. Soomro R, Khurshaidi N, Rahman SSU, Hassan R. Does antibiotic coated polyglactin helps in reducing surgical site infection in clean surgery? Medical Forum Monthly. 2017;28(2):23-26.

54. Sprowson AP, Jensen C, Parsons N, Partington P, Emmerson K, Carluke I*, et al.* The effect of triclosan-coated sutures on the rate of surgical site infection after hip and knee arthroplasty: a double-blind randomized controlled trial of 2546 patients. Bone Joint J. 2018;100-B(3):296-302.

55. Sprowson AP, Jensen CD, Parsons N, Partington P, Emmerson K, Carluke I*, et al.* The effect of triclosan coated sutures on rate of surgical site infection after hip and knee replacement: a protocol for a double-blind randomised controlled trial. BMC Musculoskelet Disord. 2014; 15: 237. Available from: <https://bmcmusculoskeletdisord.biomedcentral.com/articles/10.1186/1471-2474-15-237>

56. Sukeik M, George D, Gabr A, Kallala R, Wilson P, Haddad FS. Randomised controlled trial of triclosan coated vs uncoated sutures in primary hip and knee arthroplasty. World J Orthop. 2019;10(7):268-77.

57. University College London. A randomised controlled trial of triclosan coated sutures in primary total hip and total knee arthroplasty. Identifier: ISRCTN21430045. 2013. Available from <https://www.isrctn.com/ISRCTN21430045>.

58. Sundaram K, Warren J, Klika A, Piuzzi N, Mont M, Krebs V. Barbed sutures reduce arthrotomy closure duration compared to interrupted conventional sutures for total knee arthroplasty: a randomized controlled trial. Musculoskelet Surg. 2020a: Available from: <https://link.springer.com/article/10.1007/s12306-020-00654-y>

59. The Cleveland Clinic. The Use of STRATAFIX Suture Device Compared to Standard-of-care for Deep Tissue Closure in Total Knee Arthroplasty. Identifier: NCT03285529. In: ClinicalTrials.gov [internet]. Bethesda: US National Library of Medicine: 2017. Available from <https://clinicaltrials.gov/show/NCT03285529>.

60. Sundaram K, Piuzzi NS, Klika AK, Molloy RM, Higuera-Rueda CA, Krebs VE*, et al.* Barbed sutures reduce arthrotomy closure duration and suture utilisation compared to interrupted conventional sutures for primary total hip arthroplasty: a randomised controlled trial. Hip Int. 2020b: 1120700020911891. Available from: <https://journals.sagepub.com/doi/pdf/10.1177/1120700020911891>

61. The Cleveland Clinic. Prospective Randomized Trial of Stratafix vs. Vicryl in Total Hip Arthroplasty. Identifier: NCT03285555. In: ClinicalTrials.gov [internet]. Bethesda: US National Library of Medicine: 2017. Available from <https://clinicaltrials.gov/show/NCT03285555>.

62. Tabrizi R, Mohajerani H, Bozorgmehr F. Polyglactin 910 suture compared with polyglactin 910 coated with triclosan in dental implant surgery: randomized clinical trial. Int J Oral Maxillofac Surg. 2019;48(10):1367-71.

63. Shiraz University of Medical Sciences. Efficacy of Antimicrobial Coating Suture Coated Vicryl Plus Compared With Vicryl in Reduced Surgical Site Infection of Dental Implant Surgeries: a Uni-Blind Randomized Clinical Trial Study. Identifier: NCT03659344. In: ClinicalTrials.gov [internet]. Bethesda: US National Library of Medicine: 2018. Available from <https://clinicaltrials.gov/show/NCT03659344>.

64. Turtiainen J, Hakala T. Does the use of triclosan-coated sutures really reduce surgical site infection after open vein bypass grafting patients? Eur J Cardiothorac Surg. 2014;45(5):956.

65. Jeppsson A, Thimour-Bergstrom L, Friberg O, Gudbjartsson T. Reply to Turtiainen and Hakala. Eur J Cardiothorac Surg. 2014;45(5):957.

66. Sahlgrenska University Hospital. Effects of Triclosan-coated Sutures in Cardiac Surgery. Identifier: NCT01212315. In: ClinicalTrials.gov [internet]. Bethesda: US National Library of Medicine: 2010. Available from <https://clinicaltrials.gov/show/NCT01212315>.

67. Turtiainen J, Saimanen EIT, Makinen KT, Nykanen AI, Venermo MA, Uurto IT*, et al.* Effect of triclosan-coated sutures on the incidence of surgical wound infection after lower limb revascularization surgery: a randomized controlled trial. World J Surg. 2012;36(10):2528-34.

68. Williams N, Sweetland H, Goyal S, Ivins N, Leaper DJ. Randomized trial of antimicrobial-coated sutures to prevent surgical site infection after breast cancer surgery. Surg Infect (Larchmt). 2011;12(6):469-74.

69. Zhang Z-T, Zhang H-W, Fang X-D, Wang L-M, Li X-X, Li Y-F*, et al.* Cosmetic outcome and surgical site infection rates of antibacterial absorbable (Polyglactin 910) suture compared to Chinese silk suture in breast cancer surgery: a randomized pilot research. Chin Med J. 2011;124(5):719-24.

70. Ethicon Inc. Coated VICRYL* Plus Suture Compared to Chinese Silk in Scheduled Breast Cancer Surgery. Identifier: NCT00768222. In: ClinicalTrials.gov [internet]. Bethesda: US National Library of Medicine: 2008. Available from <https://clinicaltrials.gov/show/NCT00768222>.

71. National Institute for Health and Care Excellence. Plus Sutures for preventing surgical site infection: Medtech innovation briefing [MIB204]. London: NICE; 2020. Available from: <https://www.nice.org.uk/advice/mib204>.

72. Ahmed I, Boulton AJ, Rizvi S, Carlos W, Dickenson E, Smith NA*, et al.* The use of triclosan-coated sutures to prevent surgical site infections: a systematic review and meta-analysis of the literature. BMJ Open. 2019;9(9):e029727.

73. Onesti MG, Carella S, Scuderi N. Effectiveness of antimicrobial-coated sutures for the prevention of surgical site infection: a review of the literature. Eur Rev Med Pharmacol Sci. 2018;22(17):5729-39.

74. Leaper DJ, Edmiston CE, Jr., Holy CE. Meta-analysis of the potential economic impact following introduction of absorbable antimicrobial sutures. Br J Surg. 2017;104(2):e134-e44.

75. Wu X, Kubilay NZ, Ren J, Allegranzi B, Bischoff P, Zayed B*, et al.* Antimicrobial-coated sutures to decrease surgical site infections: a systematic review and meta-analysis. Eur J Clin Microbiol Infect Dis. 2017;36(1):19-32.

76. Clarivate Analytics. Endnote [X9 for Windows & Mac]. [program] Philadelphia: Clarivate Analytics; 2018. Available from: <http://endnote.com/>.

## Supplementary Appendix A: Full Search Strategies for the Systematic Review

The results from the searches detailed below informed the systematic literature review of clinical effects and safety evidence for Plus Sutures. Searches of economic databases were included because search results were also considered for use in the cost-effectiveness model of Plus Sutures. All records retrieved by searches of all databases were assessed, but only studies fitting the eligibility criteria described in Table 1 of the main manuscript were eligible for inclusion in the systematic literature review of clinical effects and safety evidence for Plus Sutures.

A MEDLINE (OvidSP) search strategy was designed to identify studies reporting clinical effects and adverse effects for Plus Sutures for prevention of SSIs. The final MEDLINE strategy is presented below (source A.1).

The main structure of the strategy comprised 2 concepts:

• Sutures (search lines 1 to 13)

• Triclosan (search lines 14 to 20)

The concepts were combined as follows: sutures AND triclosan.

In addition, the search included a set of search lines designed to retrieve records that explicitly referred to the device name (PDS Plus, MONOCRYL Plus, VICRYL Plus or STRATAFIX Plus) (search lines 21 to 32).

Search concepts were captured using subject headings and textword searches in Title, Abstract, Keyword Heading Word, Name of Substance Word, and CAS Registry/EC Number/Name of Substance fields. The search terms were identified through discussion within the research team, scanning background literature, browsing database thesauri and use of the PubMed PubReminer tool (http://hgserver2.amc.nl/cgi-bin/miner/miner2.cgi).

The strategy excluded animal studies from MEDLINE using a standard algorithm (search line 34). The strategy also excluded some publication types that were unlikely to yield relevant study reports (editorials and news items) (search line 35). Reflecting the eligibility criteria, the strategy was restricted to studies published in English from 2000 to date.

The performance of terms in the strategy was tested by checking retrieval of records for 46 known, potentially relevant studies. The references were sourced from the selected studies table (Table 1) in the NICE Medtech innovation briefing (MIB) on Plus Sutures for preventing surgical site infection ^[71]^ and the references included in 5 recent potentially relevant reviews ^[16, 72-75]^. Across the NICE MIB and the 5 reviews, 46 unique studies were identified for which records were available in MEDLINE. The suture-specific terms (search lines 1 to 6) successfully retrieved records for all 46 studies. The triclosan-specific terms (search lines 14 to 18) successfully retrieved records for all 46 studies. Of the 46 records, all those that included non-specific antimicrobial terms in any context (33 records) were successfully retrieved by search lines 19 or 22. Before language limits were applied, the strategy successfully retrieved records for all 46 studies.

Although the test suggested records for relevant studies would include suture-specific terms (search lines 1 to 6), the search terms for the sutures concept were enhanced by including terms to retrieve records which used variant descriptions in the context of wound closure (search lines 7 to 12). Although the test suggested records for relevant studies would include triclosan-specific terms (search lines 14 to 18), the terms for the triclosan concept were enhanced by including terms to retrieve records that only referred to non-specific antibacterial coatings in the database record (search line 19).

The search approach was discussed and agreed within the research team.

The final Ovid MEDLINE strategy was peer-reviewed by a second Information Specialist for errors in spelling, syntax and line combinations.

The searches were conducted using each database or resource listed below (sources A1 to A15). The resources included: databases covering biomedical healthcare and nursing journal literature; databases of controlled trials, systematic reviews and health technology assessments; databases containing conference abstracts; databases containing information on ongoing trials. The final agreed Ovid MEDLINE strategy was translated appropriately. Translation included consideration of differences in database interfaces and functionality, in addition to variation in indexing languages and thesauri.

The research team also asked the manufacturer to supply details of any eligible published, unpublished and ongoing studies that they were aware of. Three records were sourced in this way (see right hand box of PRISMA diagram; Figure 2 of main manuscript). The team also checked the reference lists of any relevant systematic reviews published in the last 5 years for any eligible studies that might have been missed by the database searches. No additional records were retrieved from checking of systematic reviews.

Where possible, the results of searches were downloaded in a tagged format and loaded into EndNote bibliographic software ^[76]^. The results were deduplicated using several algorithms and the duplicate references held in a separate EndNote database for checking if required. Results from resources that did not allow export in a format compatible with EndNote were saved in Word or Excel documents as appropriate and manually deduplicated.

1. **Source: MEDLINE ALL**

Interface / URL: OvidSP

Database coverage dates: 1946 to January 29, 2021

Search date: 01/02/21

Retrieved records: 422

Search strategy:

1 Sutures/ (17365)

2 Suture Techniques/ (43238)

3 sutur$.ti,ab,kf. (81242)

4 stitch$.ti,ab,kf. (5666)

5 ((surg$ or dissect$ or excis$ or fascia$ or incis$ or intraoperat$ or operat$ or postdissect$ or postexcis$ or postincis$ or postoperat$ or postsurg$ or perioperat$ or skin or skins or tissue$ or wound$) and (ligat$ or loop$ or thread$)).ti,ab,kf. (81457)

6 or/1-5 (185804)

7 Surgical Fixation Devices/ (189)

8 Wound Closure Techniques/ (1628)

9 ((surg$ or dissect$ or excis$ or fascia$ or incis$ or intraoperat$ or operat$ or postdissect$ or postexcis$ or postincis$ or postoperat$ or postsurg$ or perioperat$ or skin or skins or tissue$ or wound$) adj6 (approximat$ or clos$ or fasten$ or fixat$ or secur$)).ti,ab,kf. (103269)

10 (device$ adj6 (approximat$ or clos$ or fasten$ or fixat$ or secur$)).ti,ab,kf. (14057)

11 ((fascia$ or skin or skins or tissue$ or wound$) adj6 device$).ti,ab,kf. (7848)

12 or/7-11 (122588)

13 6 or 12 (293804)

14 Triclosan/ (2951)

15 triclosan$.ti,ab,kf,rn,nm. (4315)

16 (cgp433$ or cgp-433$ or ch3565$ or ch-3565$ or cloxifenol$ or dndi1246774$ or dndi-1246774$ or dp300$ or dp-300$ or fat-80$ or fat80$ or gp41-353$ or gp41353$ or irgacare$ or irgacide$ or irgagard$ or irgasan$ or lexol-300$ or lexol300$ or ster-zac$ or sterzac$ or tcs or tricosan$).ti,ab,kf,rn,nm. (6302)

17 (222-182-2 or 3380-34-5 or 4640-01-1 or 4nm5039y5x or 5174ur1dp5).ti,ab,kf,rn,nm. (2951)

18 or/14-17 (9767)

19 ((antibacterial$ or anti-bacterial$ or antibiotic$ or anti-biotic$ or antiinfective$ or anti-infective$ or antimicrobial$ or anti-microbial$ or antimicrobical$ or anti-microbical$ or antiseptic$ or anti-septic$ or biocid$) adj20 (coat$ or impregnat$)).ti,ab,kf. (6564)

20 13 and (18 or 19) (456)

21 plus$ suture$.ti,ab,kf. (38)

22 ((antibacterial$ or anti-bacterial$ or antibiotic$ or anti-biotic$ or antiinfective$ or anti-infective$ or antimicrobial$ or anti-microbial$ or antimicrobical$ or anti-microbical$ or antiseptic$ or anti-septic$ or biocid$) adj sutur$).ti,ab,kf. (102)

23 ((pds$ or pds-ii) adj plus$).ti,ab,kf. (19)

24 ((pds$ adj4 plus$) and sutur$).ti,ab,kf. (27)

25 (monocryl$ adj4 plus$).ti,ab,kf. (9)

26 (vicryl$ adj4 plus$).ti,ab,kf. (60)

27 (pds$ or monocryl$ or vicryl$).ti,ab,kf. and (18 or 19) (70)

28 stratafix$.ti,ab,kf. (39)

29 tissue control device$.ti,ab,kf. (8)

30 ((polydioxanon$ or poliglecapron$ or polyglactin$) adj3 plus$).ti,ab,kf. (28)

31 (polydioxanon$ or poliglecapron$ or polyglactin$).ti,ab,kf. and (18 or 19) (63)

32 or/21-31 (251)

33 20 or 32 (589)

34 exp animals/ not humans/ (4782208)

35 (news or editorial).pt. (761558)

36 33 not (34 or 35) (489)

37 limit 36 to english language (449)

38 limit 37 to yr="2000 -Current" (422)

1. **Source: Embase**

Interface / URL: OvidSP

Database coverage dates: 1974 to 2021 February 01

Search date: 02/02/21

Retrieved records: 671

Search strategy:

1 exp suture/ (64181)

2 suture technique/ or suturing method/ or suture material/ or absorbable suture material/ or nonabsorbable suture material/ (32258)

3 sutur$.ti,ab,kw,dq,dv,my. (114491)

4 stitch$.ti,ab,kw,dq,dv,my. (8765)

5 ((surg$ or dissect$ or excis$ or fascia$ or incis$ or intraoperat$ or operat$ or postdissect$ or postexcis$ or postincis$ or postoperat$ or postsurg$ or perioperat$ or skin or skins or tissue$ or wound$) and (ligat$ or loop$ or thread$)).ti,ab,kw,dq,dv,my. (114523)

6 or/1-5 (254311)

7 orthopedic fixation device/ (1772)

8 wound closure/ (18286)

9 ((surg$ or dissect$ or excis$ or fascia$ or incis$ or intraoperat$ or operat$ or postdissect$ or postexcis$ or postincis$ or postoperat$ or postsurg$ or perioperat$ or skin or skins or tissue$ or wound$) adj6 (approximat$ or clos$ or fasten$ or fixat$ or secur$)).ti,ab,kw,dq,dv,my. (135687)

10 (device$ adj6 (approximat$ or clos$ or fasten$ or fixat$ or secur$)).ti,ab,kw,dq,dv,my. (23491)

11 ((fascia$ or skin or skins or tissue$ or wound$) adj6 device$).ti,ab,kw,dq,dv,my. (11055)

12 or/7-11 (171475)

13 6 or 12 (402921)

14 triclosan/ (5498)

15 triclosan$.ti,ab,kw,rn,tn,dq,dy. (5944)

16 (cgp433$ or cgp-433$ or ch3565$ or ch-3565$ or cloxifenol$ or dndi1246774$ or dndi-1246774$ or dp300$ or dp-300$ or fat-80$ or fat80$ or gp41-353$ or gp41353$ or irgacare$ or irgacide$ or irgagard$ or irgasan$ or lexol-300$ or lexol300$ or ster-zac$ or sterzac$ or tcs or tricosan$).ti,ab,kw,rn,tn,dq,dy. (9065)

17 (222-182-2 or 3380-34-5 or 4640-01-1 or 4nm5039y5x or 5174ur1dp5).ti,ab,kw,rn,tn,dq,dy. (5213)

18 or/14-17 (13921)

19 ((antibacterial$ or anti-bacterial$ or antibiotic$ or anti-biotic$ or antiinfective$ or anti-infective$ or antimicrobial$ or anti-microbial$ or antimicrobical$ or anti-microbical$ or antiseptic$ or anti-septic$ or biocid$) adj20 (coat$ or impregnat$)).ti,ab,kw,dq,dv,my. (7725)

20 13 and (18 or 19) (674)

21 plus$ suture$.ti,ab,kw,dq,dv,my,dm. (43)

22 ((antibacterial$ or anti-bacterial$ or antibiotic$ or anti-biotic$ or antiinfective$ or anti-infective$ or antimicrobial$ or anti-microbial$ or antimicrobical$ or anti-microbical$ or antiseptic$ or anti-septic$ or biocid$) adj sutur$).ti,ab,kw,dq,dv,my,dm. (136)

23 ((pds$ or pds-ii) adj plus$).ti,ab,kw,dq,dv,my,dm. (50)

24 ((pds$ adj4 plus$) and sutur$).ti,ab,kw,dq,dv,my,dm. (52)

25 (monocryl$ adj4 plus$).ti,ab,kw,dq,dv,my,dm. (24)

26 (vicryl$ adj4 plus$).ti,ab,kw,dq,dv,my,dm. (113)

27 (pds$ or monocryl$ or vicryl$).ti,ab,kw,dq,dv,my,dm. and (18 or 19) (114)

28 stratafix$.ti,ab,kw,dq,dv,my,dm. (115)

29 tissue control device$.ti,ab,kw,dq,dv,my,dm. (17)

30 ((polydioxanon$ or poliglecapron$ or polyglactin$) adj3 plus$).ti,ab,kw,dq,dv,my,dm. (34)

31 (polydioxanon$ or poliglecapron$ or polyglactin$).ti,ab,kw,dq,dv,my,dm. and (18 or 19) (102)

32 or/21-31 (453)

33 20 or 32 (944)

34 (animal/ or animal experiment/ or animal model/ or animal tissue/ or nonhuman/) not exp human/ (6187800)

35 editorial.pt. (683611)

36 33 not (34 or 35) (757)

37 limit 36 to english language (702)

38 limit 37 to yr="2000 -Current" (671)

1. **Source: CINAHL Complete**

Interface / URL: EBSCOhost

Database coverage dates: 1937 to date

Search date: 04/02/21

Retrieved records: 162

Search strategy:

All search lines – Limiters/Expanders:

"Expanders - Apply equivalent subjects

Search modes - Boolean/Phrase"

S34 S19 OR S31 Limiters - Published Date: 20000101-20211231; English Language 162

S33 S19 OR S31 Limiters - English Language 163

S32 S19 OR S31 164

S31 S20 OR S21 OR S22 OR S23 OR S24 OR S25 OR S26 OR S27 OR S28 OR S29 OR S30 77

S30 (TI(polydioxanon* or poliglecapron* or polyglactin*) or AB(polydioxanon* or poliglecapron* or polyglactin*)) AND (S17 OR S18)

17

S29 TI((polydioxanon* or poliglecapron* or polyglactin*) N3 plus*) or AB((polydioxanon* or poliglecapron* or polyglactin*) N3 plus*)

8

S28 TI("tissue control device*") or AB("tissue control device*") 5

S27 TI stratafix* or AB stratafix* 20

S26 (TI(pds* or monocryl* or vicryl*) or AB(pds* or monocryl* or vicryl*)) AND (S17 OR S18) 11

S25 TI(vicryl* N4 plus*) or AB(vicryl* N4 plus*) 5

S24 TI(monocryl* N4 plus*) or AB(monocryl* N4 plus*) 0

S23 TI((pds* N4 plus*) and sutur*) or AB((pds* N4 plus*) and sutur*) 9

S22 TI((pds* or pds-ii) N0 plus*) or AB((pds* or pds-ii) N0 plus*) 11

S21 TI((antibacterial* or anti-bacterial* or antibiotic* or anti-biotic* or antiinfective* or anti-infective* or antimicrobial* or anti-microbial* or antimicrobical* or anti-microbical* or antiseptic* or anti-septic* or biocid*) N0 sutur*) or AB((antibacterial* or anti-bacterial* or antibiotic* or anti-biotic* or antiinfective* or anti-infective* or antimicrobial* or anti-microbial* or antimicrobical* or anti-microbical* or antiseptic* or anti-septic* or biocid*) N0 sutur*) 27

S20 TI("plus* suture*") OR AB("plus* suture*") 8

S19 S12 AND (S17 OR S18) 119

S18 TI((antibacterial* or anti-bacterial* or antibiotic* or anti-biotic* or antiinfective* or anti-infective* or antimicrobial* or anti-microbial* or antimicrobical* or anti-microbical* or antiseptic* or anti-septic* or biocid*) N20 (coat* or impregnat*)) or AB((antibacterial* or anti-bacterial* or antibiotic* or anti-biotic* or antiinfective* or anti-infective* or antimicrobial* or anti-microbial* or antimicrobical* or anti-microbical* or antiseptic* or anti-septic* or biocid*) N20 (coat* or impregnat*)) 929

S17 S13 OR S14 OR S15 OR S16 1,163

S16 TI(222-182-2 or 3380-34-5 or 4640-01-1 or 4nm5039y5x or 5174ur1dp5) or AB(222-182-2 or 3380-34-5 or 4640-01-1 or 4nm5039y5x or 5174ur1dp5) 0

S15 TI(cgp433* or cgp-433* or ch3565* or ch-3565* or cloxifenol* or dndi1246774* or dndi-1246774* or dp300* or dp-300* or fat-80* or fat80* or gp41-353* or gp41353* or irgacare* or irgacide* or irgagard* or irgasan* or lexol-300* or lexol300* or ster-zac* or sterzac* or tcs or tricosan*) or AB(cgp433* or cgp-433* or ch3565* or ch-3565* or cloxifenol* or dndi1246774* or dndi-1246774* or dp300* or dp-300* or fat-80* or fat80* or gp41-353* or gp41353* or irgacare* or irgacide* or irgagard* or irgasan* or lexol-300* or lexol300* or ster-zac* or sterzac* or tcs or tricosan*) 698

S14 TI triclosan* or AB triclosan* 396

S13 (MH "Triclosan") 271

S12 S6 OR S11 46,949

S11 S7 OR S8 OR S9 OR S10 26,296

S10 TI((fascia* or skin or skins or tissue* or wound*) N6 device*) or AB((fascia* or skin or skins or tissue* or wound*) N6 device*) 1,824

S9 TI(device* N6 (approximat* or clos* or fasten* or fixat* or secur*)) or AB(device* N6 (approximat* or clos* or fasten* or fixat* or secur*))

4,076

S8 TI((surg* or dissect* or excis* or fascia* or incis* or intraoperat* or operat* or postdissect* or postexcis* or postincis* or postoperat* or postsurg* or perioperat* or skin or skins or tissue* or wound*) N6 (approximat* or clos* or fasten* or fixat* or secur*)) or AB((surg* or dissect* or excis* or fascia* or incis* or intraoperat* or operat* or postdissect* or postexcis* or postincis* or postoperat* or postsurg* or perioperat* or skin or skins or tissue* or wound*) N6 (approximat* or clos* or fasten* or fixat* or secur*)) 21,392

S7 (MH "Surgical Fixation Devices") 156

S6 S1 OR S2 OR S3 OR S4 OR S5 23,613

S5 TI((surg* or dissect* or excis* or fascia* or incis* or intraoperat* or operat* or postdissect* or postexcis* or postincis* or postoperat* or postsurg* or perioperat* or skin or skins or tissue* or wound*) and (ligat* or loop* or thread*)) or AB((surg* or dissect* or excis* or fascia* or incis* or intraoperat* or operat* or postdissect* or postexcis* or postincis* or postoperat* or postsurg* or perioperat* or skin or skins or tissue* or wound*) and (ligat* or loop* or thread*)) 8,022

S4 TI stitch* or AB stitch* 1,028

S3 TI sutur* or AB sutur* 12,048

S2 (MH "Suture Techniques") 6,190

S1 (MH "Sutures") 3,697

1. **Source: Cochrane Central Register of Controlled Trials**

Interface / URL: Cochrane Library / Wiley

Database coverage dates: Information not found. Issue searched: Issue 2 of 12, February 2021

Search date: 03/02/21

Retrieved records: 203

Search strategy:

#1 [mh ^Sutures] 919

#2 [mh ^"Suture Techniques"] 1786

#3 sutur* 9351

#4 stitch* 812

#5 ((surg* or dissect* or excis* or fascia* or incis* or intraoperat* or operat* or postdissect* or postexcis* or postincis* or postoperat* or postsurg* or perioperat* or skin or skins or tissue* or wound*) and (ligat* or loop* or thread*)) 5758

#6 #1 or #2 or #3 or #4 or #5 14730

#7 [mh ^"Surgical Fixation Devices"] 11

#8 [mh ^"Wound Closure Techniques"] 155

#9 ((surg* or dissect* or excis* or fascia* or incis* or intraoperat* or operat* or postdissect* or postexcis* or postincis* or postoperat* or postsurg* or perioperat* or skin or skins or tissue* or wound*) near/6 (approximat* or clos* or fasten* or fixat* or secur*)) 13625

#10 (device* near/6 (approximat* or clos* or fasten* or fixat* or secur*)) 1940

#11 ((fascia* or skin or skins or tissue* or wound*) near/6 device*) 1447

#12 #7 or #8 or #9 or #10 or #11 15964

#13 #6 or #12 27511

#14 [mh ^Triclosan] 410

#15 triclosan* 715

#16 (cgp433* or cgp next 433* or ch3565* or ch next 3565* or cloxifenol* or dndi1246774* or dndi next 1246774* or dp300* or dp next 300* or "fat-80" or "fat-80r" or "fat-80tm" or fat80* or gp41 next 353* or gp41353* or irgacare* or irgacide* or irgagard* or irgasan* or lexol next 300* or lexol300* or ster next zac* or sterzac* or tcs or tricosan*) 485

#17 ("222-182-2" or "3380-34-5" or "4640-01-1" or 4nm5039y5x or 5174ur1dp5) 0

#18 #14 or #15 or #16 or #17 1170

#19 ((antibacterial* or anti next bacterial* or antibiotic* or anti next biotic* or antiinfective* or anti next infective* or antimicrobial* or anti next microbial* or antimicrobical* or anti next microbical* or antiseptic* or anti next septic* or biocid*) near/20 (coat* or impregnat*)) 593

#20 #13 and (#18 or #19) 198

#21 (plus* next suture*) 23

#22 ((antibacterial* or anti next bacterial* or antibiotic* or anti next biotic* or antiinfective* or anti next infective* or antimicrobial* or anti next microbial* or antimicrobical* or anti next microbical* or antiseptic* or anti next septic* or biocid*) next sutur*) 49

#23 ((pds* or "pds-ii") next plus*) 18

#24 ((pds* near/4 plus*) and sutur*) 20

#25 (monocryl* near/4 plus*) 9

#26 (vicryl* near/4 plus*) 41

#27 (pds* or monocryl* or vicryl*) and (#18 or #19) 50

#28 stratafix* 30

#29 (tissue next control next device*) 8

#30 ((polydioxanon* or poliglecapron* or polyglactin*) near/3 plus*) 13

#31 (polydioxanon* or poliglecapron* or polyglactin*) and (#18 or #19) 48

#32 #21 or #22 or #23 or #24 or #25 or #26 or #27 or #28 or #29 or #30 or #31 154

#33 #20 or #32 266

#34 #33 with Publication Year from 2000 to 2021, in Trials 203

1. **Source: Cochrane Database of Systematic Reviews**

Interface / URL: Cochrane Library / Wiley

Database coverage dates: Information not found. Issue searched: Issue 2 of 12, February 2021

Search date: 03/02/21

Retrieved records: 21

Search strategy:

#1 [mh ^Sutures] 919

#2 [mh ^"Suture Techniques"] 1786

#3 sutur*:ti,ab,kw 9004

#4 stitch*:ti,ab,kw 764

#5 ((surg* or dissect* or excis* or fascia* or incis* or intraoperat* or operat* or postdissect* or postexcis* or postincis* or postoperat* or postsurg* or perioperat* or skin or skins or tissue* or wound*) and (ligat* or loop* or thread*)):ti,ab,kw 5028

#6 #1 or #2 or #3 or #4 or #5 13720

#7 [mh ^"Surgical Fixation Devices"] 11

#8 [mh ^"Wound Closure Techniques"] 155

#9 ((surg* or dissect* or excis* or fascia* or incis* or intraoperat* or operat* or postdissect* or postexcis* or postincis* or postoperat* or postsurg* or perioperat* or skin or skins or tissue* or wound*) near/6 (approximat* or clos* or fasten* or fixat* or secur*)):ti,ab,kw 12286

#10 (device* near/6 (approximat* or clos* or fasten* or fixat* or secur*)):ti,ab,kw 1782

#11 ((fascia* or skin or skins or tissue* or wound*) near/6 device*):ti,ab,kw 1276

#12 #7 or #8 or #9 or #10 or #11 14512

#13 #6 or #12 25376

#14 [mh ^Triclosan] 410

#15 triclosan* 715

#16 (cgp433* or cgp next 433* or ch3565* or ch next 3565* or cloxifenol* or dndi1246774* or dndi next 1246774* or dp300* or dp next 300* or "fat-80" or "fat-80r" or "fat-80tm" or fat80* or gp41 next 353* or gp41353* or irgacare* or irgacide* or irgagard* or irgasan* or lexol next 300* or lexol300* or ster next zac* or sterzac* or tcs or tricosan*) 485

#17 ("222-182-2" or "3380-34-5" or "4640-01-1" or 4nm5039y5x or 5174ur1dp5) 0

#18 #14 or #15 or #16 or #17 1170

#19 ((antibacterial* or anti next bacterial* or antibiotic* or anti next biotic* or antiinfective* or anti next infective* or antimicrobial* or anti next microbial* or antimicrobical* or anti next microbical* or antiseptic* or anti next septic* or biocid*) near/20 (coat* or impregnat*)) 593

#20 #13 and (#18 or #19) 156

#21 (plus* next suture*) 23

#22 ((antibacterial* or anti next bacterial* or antibiotic* or anti next biotic* or antiinfective* or anti next infective* or antimicrobial* or anti next microbial* or antimicrobical* or anti next microbical* or antiseptic* or anti next septic* or biocid*) next sutur*) 49

#23 ((pds* or "pds-ii") next plus*) 18

#24 ((pds* near/4 plus*) and sutur*) 20

#25 (monocryl* near/4 plus*) 9

#26 (vicryl* near/4 plus*) 41

#27 (pds* or monocryl* or vicryl*) and (#18 or #19) 50

#28 stratafix* 30

#29 (tissue next control next device*) 8

#30 ((polydioxanon* or poliglecapron* or polyglactin*) near/3 plus*) 13

#31 (polydioxanon* or poliglecapron* or polyglactin*) and (#18 or #19) 48

#32 #21 or #22 or #23 or #24 or #25 or #26 or #27 or #28 or #29 or #30 or #31 154

#33 #20 or #32 225

#34 #33 with Cochrane Library publication date Between Jan 2000 and Feb 2021, in Cochrane Reviews, Cochrane Protocols 21

1. **Source: Database of Abstracts of Reviews of Effects (DARE)**

Interface / URL: https://www.crd.york.ac.uk/CRDWeb

Database coverage dates: Information not found. Bibliographic records were published on DARE until 31st March 2015. Searches of MEDLINE, Embase, CINAHL, PsycINFO and PubMed were continued until the end of the 2014.

Search date: 03/02/21

Retrieved records: 21

Search strategy:

1 MeSH DESCRIPTOR Sutures 86

2 MeSH DESCRIPTOR Suture Techniques 187

3 (sutur*) 442

4 (stitch*) 27

5 (((surg* or dissect* or excis* or fascia* or incis* or intraoperat* or operat* or postdissect* or postexcis* or postincis* or postoperat* or postsurg* or perioperat* or skin or skins or tissue* or wound*) and (ligat* or loop* or thread*))) 263

6 #1 OR #2 OR #3 OR #4 OR #5 687

7 MeSH DESCRIPTOR Surgical Fixation Devices 5

8 MeSH DESCRIPTOR Wound Closure Techniques 21

9 (((surg* or dissect* or excis* or fascia* or incis* or intraoperat* or operat* or postdissect* or postexcis* or postincis* or postoperat* or postsurg* or perioperat* or skin or skins or tissue* or wound*) and (approximat* or clos* or fasten* or fixat* or secur*))) 2836

10 ((device* and (approximat* or clos* or fasten* or fixat* or secur*))) 462

11 (((fascia* or skin or skins or tissue* or wound*) and device*)) 329

12 #7 OR #8 OR #9 OR #10 OR #11 3247

13 (#6 OR #12) 3697

14 MeSH DESCRIPTOR Triclosan 12

15 (triclosan*) 23

16 ((cgp433* or cgp-433* or ch3565* or ch-3565* or cloxifenol* or dndi1246774* or dndi-1246774* or dp300* or dp-300* or fat-80* or fat80* or gp41-353* or gp41353* or irgacare* or irgacide* or irgagard* or irgasan* or lexol-300* or lexol300* or ster-zac* or sterzac* or tcs or tricosan*) ) 7

17 ((222-182-2 or 3380-34-5 or 4640-01-1 or 4nm5039y5x or 5174ur1dp5)) 0

18 #14 OR #15 OR #16 OR #17 30

19 (((antibacterial* or anti-bacterial* or antibiotic* or anti-biotic* or antiinfective* or anti-infective* or antimicrobial* or anti-microbial* or antimicrobical* or anti-microbical* or antiseptic* or anti-septic* or biocid*) AND (coat* or impregnat*))) 138

20 (#13 and (#18 or #19)) 38

21 (plus* suture*) 1

22 (((antibacterial* or anti-bacterial* or antibiotic* or anti-biotic* or antiinfective* or anti-infective* or antimicrobial* or anti-microbial* or antimicrobical* or anti-microbical* or antiseptic* or anti-septic* or biocid*) adj0 sutur*)) 8

23 (((pds* or pds-ii) adj0 plus*)) 2

24 ((pds* and plus* and sutur*)) 2

25 ((monocryl* and plus*)) 1

26 ((vicryl* and plus*)) 1

27 ((pds* or monocryl* or vicryl*) and (#18 or #19) ) 2

28 (stratafix*) 0

29 (tissue control device*) 0

30 (((polydioxanon* or poliglecapron* or polyglactin*) and plus*)) 2

31 ((polydioxanon* or poliglecapron* or polyglactin*) and (#18 or #19)) 2

32 #21 OR #22 OR #23 OR #24 OR #25 OR #26 OR #27 OR #28 OR #29 OR #30 OR #31 13

33 #20 OR #32 47

34 (#33) FROM 2000 TO 2021 44

35 (#33) IN DARE FROM 2000 TO 2021 21

1. **Source: NHS Economic Evaluation Database (NHS EED)**

Interface / URL: https://www.crd.york.ac.uk/CRDWeb

Database coverage dates: Information not found. Bibliographic records were published on NHS EED until 31st March 2015. Searches of MEDLINE, Embase, CINAHL, PsycINFO and PubMed were continued until the end of the 2014.

Search date: 03/02/21

Retrieved records: 9

Search strategy:

1 MeSH DESCRIPTOR Sutures 86

2 MeSH DESCRIPTOR Suture Techniques 187

3 (sutur*) 442

4 (stitch*) 27

5 (((surg* or dissect* or excis* or fascia* or incis* or intraoperat* or operat* or postdissect* or postexcis* or postincis* or postoperat* or postsurg* or perioperat* or skin or skins or tissue* or wound*) and (ligat* or loop* or thread*))) 263

6 #1 OR #2 OR #3 OR #4 OR #5 687

7 MeSH DESCRIPTOR Surgical Fixation Devices 5

8 MeSH DESCRIPTOR Wound Closure Techniques 21

9 (((surg* or dissect* or excis* or fascia* or incis* or intraoperat* or operat* or postdissect* or postexcis* or postincis* or postoperat* or postsurg* or perioperat* or skin or skins or tissue* or wound*) and (approximat* or clos* or fasten* or fixat* or secur*))) 2836

10 ((device* and (approximat* or clos* or fasten* or fixat* or secur*))) 462

11 (((fascia* or skin or skins or tissue* or wound*) and device*)) 329

12 #7 OR #8 OR #9 OR #10 OR #11 3247

13 (#6 OR #12) 3697

14 MeSH DESCRIPTOR Triclosan 12

15 (triclosan*) 23

16 ((cgp433* or cgp-433* or ch3565* or ch-3565* or cloxifenol* or dndi1246774* or dndi-1246774* or dp300* or dp-300* or fat-80* or fat80* or gp41-353* or gp41353* or irgacare* or irgacide* or irgagard* or irgasan* or lexol-300* or lexol300* or ster-zac* or sterzac* or tcs or tricosan*) ) 7

17 ((222-182-2 or 3380-34-5 or 4640-01-1 or 4nm5039y5x or 5174ur1dp5)) 0

18 #14 OR #15 OR #16 OR #17 30

19 (((antibacterial* or anti-bacterial* or antibiotic* or anti-biotic* or antiinfective* or anti-infective* or antimicrobial* or anti-microbial* or antimicrobical* or anti-microbical* or antiseptic* or anti-septic* or biocid*) AND (coat* or impregnat*))) 138

20 (#13 and (#18 or #19)) 38

21 (plus* suture*) 1

22 (((antibacterial* or anti-bacterial* or antibiotic* or anti-biotic* or antiinfective* or anti-infective* or antimicrobial* or anti-microbial* or antimicrobical* or anti-microbical* or antiseptic* or anti-septic* or biocid*) adj0 sutur*)) 8

23 (((pds* or pds-ii) adj0 plus*)) 2

24 ((pds* and plus* and sutur*)) 2

25 ((monocryl* and plus*)) 1

26 ((vicryl* and plus*)) 1

27 ((pds* or monocryl* or vicryl*) and (#18 or #19) ) 2

28 (stratafix*) 0

29 (tissue control device*) 0

30 (((polydioxanon* or poliglecapron* or polyglactin*) and plus*)) 2

31 ((polydioxanon* or poliglecapron* or polyglactin*) and (#18 or #19)) 2

32 #21 OR #22 OR #23 OR #24 OR #25 OR #26 OR #27 OR #28 OR #29 OR #30 OR #31 13

33 #20 OR #32 47

34 (#33) FROM 2000 TO 2021 44

35 (#33) IN DARE FROM 2000 TO 2021 21

36 (#33) IN NHSEED FROM 2000 TO 2021 9

1. **Source: HTA Database**

Interface / URL: https://www.inahta.org/hta-database/

Database coverage dates: Information not found. The former database was produced by the CRD until March 2018, at which time the addition of records was stopped as INAHTA was in the process of rebuilding the new database platform. In July 2019, the database records were exported from the CRD platform and imported into the new platform that was developed by INAHTA. The rebuild of the new platform was launched in June 2020.

Search date: 03/02/21

Retrieved records: 14

Search strategy:

32 #31 AND #30 14

31 * FROM 2000 TO 2021 16140

30 #29 OR #22 15

29 #28 OR #27 OR #26 OR #25 OR #24 OR #23 11

28 (polydioxanon* OR poliglecapron* OR polyglactin*) 0

27 "tissue control device" OR "tissue control devices" 0

26 stratafix* 0

25 (pds* OR monocryl* OR vicryl*) 4

24 ((antibacterial* OR "anti-bacterial" OR "anti-bacterials" OR antibiotic* OR "anti-biotic" OR "anti-biotics" OR antiinfective* OR "anti-infective" OR "anti-infectives" OR antimicrobial* OR "anti-microbial" OR "anti-microbials" OR antimicrobical* OR "anti-microbical" OR "anti-microbicals" OR antiseptic* OR "anti-septic" OR "anti-septics" OR biocid*) AND sutur*) 8

23 plus* AND suture* 2

22 #21 OR #20 7

21 #19 AND #13 6

20 #18 AND #13 3

19 ((antibacterial* OR "anti-bacterial" OR "anti-bacterials" OR antibiotic* OR "anti-biotic" OR "anti-biotics" OR antiinfective* OR "anti-infective" OR "anti-infectives" OR antimicrobial* OR "anti-microbial" OR "anti-microbials" OR antimicrobical* OR "anti-microbical" OR "anti-microbicals" OR antiseptic* OR "anti-septic" OR "anti-septics" OR biocid*) AND (coat* OR impregnat*)) 21

18 #17 OR #16 OR #15 OR #14 6

17 (4nm5039y5x OR 5174ur1dp5) 0

16 (cgp433* OR "cgp-433" OR "cgp-433r" OR "cgp-433tm" OR ch3565* OR cloxifenol* OR dndi1246774* OR "dndi-1246774" OR "dndi-1246774r" OR "dndi-1246774tm" OR dp300* OR "fat-80r" OR "fat-80tm" OR fat80* OR "gp41-353" OR "gp41-353r" OR "gp41-353tm" OR gp41353* OR irgacare* OR irgacide* OR irgagard* OR irgasan* OR "lexol-300" OR "lexol-300r" OR "lexol-300tm" OR lexol300* OR "ster-zac" OR "ster-zacr" OR "ster-zactm" OR sterzac* OR tcs OR tricosan*) 2

15 triclosan* 4

14 "Triclosan"[mh] 1

13 #12 OR #6 703

12 #11 OR #10 OR #9 OR #8 OR #7 648

11 ((fascia* OR skin OR skins OR tissue* OR wound*) AND device*) 97

10 (device* AND (approximat* OR clos* OR fasten* OR fixat* OR secur*)) 129

9 ((surg* OR dissect* OR excis* OR fascia* OR incis* OR intraoperat* OR operat* OR postdissect* OR postexcis* OR postincis* OR postoperat* OR postsurg* OR perioperat* OR skin OR skins OR tissue* OR wound*) AND (approximat* OR clos* OR fasten* OR fixat* OR secur*)) 508

8 "Wound Closure Techniques"[mh] 0

7 "Surgical Fixation Devices"[mh] 0

6 #5 OR #4 OR #3 OR #2 OR #1 81

5 ((surg* OR dissect* OR excis* OR fascia* OR incis* OR intraoperat* OR operat* OR postdissect* OR postexcis* OR postincis* OR postoperat* OR postsurg* OR perioperat* OR skin OR skins OR tissue* OR wound*) AND (ligat* OR loop* OR thread*)) 22

4 stitch* 1

3 sutur* 55

2 "Suture Techniques"[mh] 10

1 "Sutures"[mh] 15

Search note: It is not possible to search on terms containing less than three characters in the HTA Database. The following terms were therefore not included in the search strategy:

- "ch-3565"
- "ch-3565r"
- "ch-3565tm"
- "dp-300"
- "dp-300r"
- "dp-300tm"
- "fat-80"
- "222-182-2"
- "3380-34-5"
- "4640-01-1"
- "pds-ii"

1. **Source: Econlit**

Interface / URL: OvidSP

Database coverage dates: 1886 to January 21,2021

Search date: 03/02/21

Retrieved records: 0

Search strategy:

1 sutur$.af. (7)

2 stitch$.af. (45)

3 ((surg$ or dissect$ or excis$ or fascia$ or incis$ or intraoperat$ or operat$ or postdissect$ or postexcis$ or postincis$ or postoperat$ or postsurg$ or perioperat$ or skin or skins or tissue$ or wound$) and (ligat$ or loop$ or thread$)).af. (303)

4 or/1-3 (354)

5 ((surg$ or dissect$ or excis$ or fascia$ or incis$ or intraoperat$ or operat$ or postdissect$ or postexcis$ or postincis$ or postoperat$ or postsurg$ or perioperat$ or skin or skins or tissue$ or wound$) adj6 (approximat$ or clos$ or fasten$ or fixat$ or secur$)).af. (955)

6 (device$ adj6 (approximat$ or clos$ or fasten$ or fixat$ or secur$)).af. (66)

7 ((fascia$ or skin or skins or tissue$ or wound$) adj6 device$).af. (3)

8 or/5-7 (1017)

9 4 or 8 (1357)

10 triclosan$.af. (0)

11 (cgp433$ or cgp-433$ or ch3565$ or ch-3565$ or cloxifenol$ or dndi1246774$ or dndi-1246774$ or dp300$ or dp-300$ or fat-80$ or fat80$ or gp41-353$ or gp41353$ or irgacare$ or irgacide$ or irgagard$ or irgasan$ or lexol-300$ or lexol300$ or ster-zac$ or sterzac$ or tcs or tricosan$).af. (86)

12 (222-182-2 or 3380-34-5 or 4640-01-1 or 4nm5039y5x or 5174ur1dp5).af. (0)

13 or/10-12 (86)

14 ((antibacterial$ or anti-bacterial$ or antibiotic$ or anti-biotic$ or antiinfective$ or anti-infective$ or antimicrobial$ or anti-microbial$ or antimicrobical$ or anti-microbical$ or antiseptic$ or anti-septic$ or biocid$) adj20 (coat$ or impregnat$)).af. (0)

15 9 and (13 or 14) (0)

16 plus$ suture$.af. (0)

17 ((antibacterial$ or anti-bacterial$ or antibiotic$ or anti-biotic$ or antiinfective$ or anti-infective$ or antimicrobial$ or anti-microbial$ or antimicrobical$ or anti-microbical$ or antiseptic$ or anti-septic$ or biocid$) adj sutur$).af. (0)

18 ((pds$ or pds-ii) adj plus$).af. (0)

19 ((pds$ adj4 plus$) and sutur$).af. (0)

20 (monocryl$ adj4 plus$).af. (0)

21 (vicryl$ adj4 plus$).af. (0)

22 (pds$ or monocryl$ or vicryl$).af. and (13 or 14) (0)

23 stratafix$.af. (0)

24 tissue control device$.af. (0)

25 ((polydioxanon$ or poliglecapron$ or polyglactin$) adj3 plus$).af. (0)

26 (polydioxanon$ or poliglecapron$ or polyglactin$).af. and (13 or 14) (0)

27 or/16-26 (0)

28 15 or 27 (0)

29 limit 28 to english (0)

30 limit 29 to yr="2000 -Current" (0)

1. **Source: Conference Proceedings Citation Index – Science (CPCI-S)**

Interface / URL: Web of Science

Database coverage dates: 1990 - present

Search date: 03/02/21

Retrieved records: 50

Search strategy:

All lines: Indexes=CPCI-S

# 29 50 (#28) AND LANGUAGE: (English) Timespan=2000-2021

# 28 60 #27 OR #15

# 27 16 #26 OR #25 OR #24 OR #23 OR #22 OR #21 OR #20 OR #19 OR #18 OR #17 OR #16

# 26 0 TS=(polydioxanon* or poliglecapron* or polyglactin*) and (#13 or #14)

# 25 2 TS=((polydioxanon* or poliglecapron* or polyglactin*) near/3 plus*)

# 24 0 TS="tissue control device*"

# 23 0 TS=stratafix*

# 22 4 TS=(pds* or monocryl* or vicryl*) and (#13 or #14)

# 21 6 TS=(vicryl* near/4 plus*)

# 20 1 TS=(monocryl* near/4 plus*)

# 19 0 TS=((pds* near/4 plus*) and sutur*)

# 18 0 TS=((pds* or "pds-ii") near/0 plus*)

# 17 10 TS=((antibacterial* or "anti-bacterial*" or antibiotic* or "anti-biotic*" or antiinfective* or "anti-infective*" or antimicrobial* or "anti-microbial*" or antimicrobical* or "anti-microbical*" or antiseptic* or "anti-septic*" or biocid*) near/0 sutur*)

# 16 1 TS="plus* suture*"

# 15 48 #9 and (#13 or #14)

# 14 956 TS=((antibacterial* or "anti-bacterial*" or antibiotic* or "anti-biotic*" or antiinfective* or "anti-infective*" or antimicrobial* or "anti-microbial*" or antimicrobical* or "anti-microbical*" or antiseptic* or "anti-septic*" or biocid*) near/20 (coat* or impregnat*) )

# 13 1,639 #12 OR #11 OR #10

# 12 0 TS=("222-182-2" or "3380-34-5" or "4640-01-1" or "4nm5039y5x" or "5174ur1dp5")

# 11 1,323 TS=(cgp433* or "cgp-433*" or ch3565* or "ch-3565*" or cloxifenol* or dndi1246774* or "dndi-1246774*" or dp300* or "dp-300*" or "fat-80" or "fat-80r" or "fat-80tm" or fat80* or "gp41-353*" or gp41353* or irgacare* or irgacide* or irgagard* or irgasan* or "lexol-300*" or lexol300* or "ster-zac*" or sterzac* or tcs or tricosan*)

# 10 350 TS=triclosan*

# 9 77,194 #8 OR #4

# 8 36,418 #7 OR #6 OR #5

# 7 1,907 TS=((fascia* or "skin" or "skins" or tissue* or wound*) near/6 device*)

# 6 9,777 TS=(device* near/6 (approximat* or clos* or fasten* or fixat* or secur*) )

# 5 25,592 TS=((surg* or dissect* or excis* or fascia* or incis* or intraoperat* or operat* or postdissect* or postexcis* or postincis* or postoperat* or postsurg* or perioperat* or "skin" or "skins" or tissue* or wound*) near/6 (approximat* or clos* or fasten* or fixat* or secur*) )

# 4 43,920 #3 OR #2 OR #1

# 3 34,066 TS=((surg* or dissect* or excis* or fascia* or incis* or intraoperat* or operat* or postdissect* or postexcis* or postincis* or postoperat* or postsurg* or perioperat* or "skin" or "skins" or tissue* or wound*) and (ligat* or loop* or thread*) )

# 2 3,982 TS=stitch*

# 1 6,380 TS=sutur*

1. **Source: Epistemonikos**

Interface / URL: https://www.epistemonikos.org/en/

Database coverage dates: Information not found

Search date: 03/02/21

Retrieved records: 193

Search strategy:

The following 10 searches were conducted separately. The searches were conducted using the Advanced search interface at <https://www.epistemonikos.org/en/advanced_search>.

Terms were entered into the main search box. No field tags were used. From the results screen the Filter options were used to limit the results. A custom year range of 2000-2021 was applied for "Publication Year". Results were limited by "Publication type" to systematic review.

The 10 sets of results (257 in total) were downloaded and imported into an empty ENL. Records were deduplicated using EndNote default settings. 64 records were removed as duplicates. The remaining 193 records were retrieved for assessment.

Search 1: ((sutur* OR stitch*) AND (triclosan* OR cgp433* OR "cgp-433" OR "cgp-433r" OR "cgp-433tm" OR ch3565* OR "ch-3565" OR "ch-3565r" OR "ch-3565tm" OR cloxifenol* OR dndi1246774* OR "dndi-1246774" OR "dndi-1246774r" OR "dndi-1246774tm" OR dp300* OR "dp-300" OR "dp-300r" OR "dp-300tm" OR "fat-80" OR "fat-80r" OR "fat-80tm" OR fat80* OR "gp41-353" OR "gp41-353r" OR "gp41-353tm" OR gp41353* OR irgacare* OR irgacide* OR irgagard* OR irgasan* OR "lexol-300" OR "lexol-300r" OR "lexol-300tm" OR lexol300* OR "ster-zac" OR "ster-zacr" OR "ster-zactm" OR sterzac* OR tcs OR tricosan* OR "222-182-2" OR "3380-34-5" OR "4640-01-1" OR 4nm5039y5x OR 5174ur1dp5)) = 27

Search 2: (stitch* AND (antibacterial* OR "anti-bacterial" OR "anti-bacterials" OR antibiotic* OR "anti-biotic" OR "anti-biotics" OR antiinfective* OR "anti-infective" OR "anti-infectives" OR antimicrobial* OR "anti-microbial" OR "anti-microbials" OR antimicrobical* OR "anti-microbical" OR "anti-microbicals" OR antiseptic* OR "anti-septic" OR "anti-septics" OR biocid*) AND (coat* OR impregnat*)) = 0

Search 3: ((surg* OR dissect* OR excis* OR fascia* OR incis* OR intraoperat* OR operat* OR postdissect* OR postexcis* OR postincis* OR postoperat* OR postsurg* OR perioperat* OR skin* OR tissue* OR wound*) AND (ligat* OR loop* OR thread* OR approximat* OR clos* OR fasten* OR fixat* OR secur*) AND (triclosan* OR cgp433* OR "cgp-433" OR "cgp-433r" OR "cgp-433tm" OR ch3565* OR "ch-3565" OR "ch-3565r" OR "ch-3565tm" OR cloxifenol* OR dndi1246774* OR "dndi-1246774" OR "dndi-1246774r" OR "dndi-1246774tm" OR dp300* OR "dp-300" OR "dp-300r" OR "dp-300tm" OR "fat-80" OR "fat-80r" OR "fat-80tm" OR fat80* OR "gp41-353" OR "gp41-353r" OR "gp41-353tm" OR gp41353* OR irgacare* OR irgacide* OR irgagard* OR irgasan* OR "lexol-300" OR "lexol-300r" OR "lexol-300tm" OR lexol300* OR "ster-zac" OR "ster-zacr" OR "ster-zactm" OR sterzac* OR tcs OR tricosan* OR "222-182-2" OR "3380-34-5" OR "4640-01-1" OR 4nm5039y5x OR 5174ur1dp5)) = 46

Search 4: ((surg* OR dissect* OR excis* OR fascia* OR incis* OR intraoperat* OR operat* OR postdissect* OR postexcis* OR postincis* OR postoperat* OR postsurg* OR perioperat* OR skin* OR tissue* OR wound*) AND (ligat* OR loop* OR thread* OR approximat* OR clos* OR fasten* OR fixat* OR secur*) AND (antibacterial* OR "anti-bacterial" OR "anti-bacterials" OR antibiotic* OR "anti-biotic" OR "anti-biotics" OR antiinfective* OR "anti-infective" OR "anti-infectives" OR antimicrobial* OR "anti-microbial" OR "anti-microbials" OR antimicrobical* OR "anti-microbical" OR "anti-microbicals" OR antiseptic* OR "anti-septic" OR "anti-septics" OR biocid*) AND (coat* OR impregnat*)) = 41

Search 5: (device* AND (approximat* OR clos* OR fasten* OR fixat* OR secur* OR fascia* OR skin* OR tissue* OR wound*) AND (triclosan* OR cgp433* OR "cgp-433" OR "cgp-433r" OR "cgp-433tm" OR ch3565* OR "ch-3565" OR "ch-3565r" OR "ch-3565tm" OR cloxifenol* OR dndi1246774* OR "dndi-1246774" OR "dndi-1246774r" OR "dndi-1246774tm" OR dp300* OR "dp-300" OR "dp-300r" OR "dp-300tm" OR "fat-80" OR "fat-80r" OR "fat-80tm" OR fat80* OR "gp41-353" OR "gp41-353r" OR "gp41-353tm" OR gp41353* OR irgacare* OR irgacide* OR irgagard* OR irgasan* OR "lexol-300" OR "lexol-300r" OR "lexol-300tm" OR lexol300* OR "ster-zac" OR "ster-zacr" OR "ster-zactm" OR sterzac* OR tcs OR tricosan* OR "222-182-2" OR "3380-34-5" OR "4640-01-1" OR 4nm5039y5x OR 5174ur1dp5)) = 5

Search 6: (device* AND (approximat* OR clos* OR fasten* OR fixat* OR secur* OR fascia* OR skin* OR tissue* OR wound*) AND (antibacterial* OR "anti-bacterial" OR "anti-bacterials" OR antibiotic* OR "anti-biotic" OR "anti-biotics" OR antiinfective* OR "anti-infective" OR "anti-infectives" OR antimicrobial* OR "anti-microbial" OR "anti-microbials" OR antimicrobical* OR "anti-microbical" OR "anti-microbicals" OR antiseptic* OR "anti-septic" OR "anti-septics" OR biocid*) AND (coat* OR impregnat*)) = 15

Search 7: ("plus suture" OR "plus sutures" OR "pds plus" OR "pdsii plus" OR "pds-ii plus" OR (pds* AND plus* AND suture*) OR monocryl* OR vicryl* OR stratafix* OR "tissue control device" OR "tissue control devices" OR polydioxanon* OR poliglecapron* OR polyglactin*) = 34

Search 8: (pds* AND (triclosan* OR cgp433* OR "cgp-433" OR "cgp-433r" OR "cgp-433tm" OR ch3565* OR "ch-3565" OR "ch-3565r" OR "ch-3565tm" OR cloxifenol* OR dndi1246774* OR "dndi-1246774" OR "dndi-1246774r" OR "dndi-1246774tm" OR dp300* OR "dp-300" OR "dp-300r" OR "dp-300tm" OR "fat-80" OR "fat-80r" OR "fat-80tm" OR fat80* OR "gp41-353" OR "gp41-353r" OR "gp41-353tm" OR gp41353* OR irgacare* OR irgacide* OR irgagard* OR irgasan* OR "lexol-300" OR "lexol-300r" OR "lexol-300tm" OR lexol300* OR "ster-zac" OR "ster-zacr" OR "ster-zactm" OR sterzac* OR tcs OR tricosan* OR "222-182-2" OR "3380-34-5" OR "4640-01-1" OR 4nm5039y5x OR 5174ur1dp5)) = 31

Search 9: (pds* AND (antibacterial* OR "anti-bacterial" OR "anti-bacterials" OR antibiotic* OR "anti-biotic" OR "anti-biotics" OR antiinfective* OR "anti-infective" OR "anti-infectives" OR antimicrobial* OR "anti-microbial" OR "anti-microbials" OR antimicrobical* OR "anti-microbical" OR "anti-microbicals" OR antiseptic* OR "anti-septic" OR "anti-septics" OR biocid*) AND (coat* OR impregnat*)) = 1

Search 10: ((antibacterial* OR "anti-bacterial" OR "anti-bacterials" OR antibiotic* OR "anti-biotic" OR "anti-biotics" OR antiinfective* OR "anti-infective" OR "anti-infectives" OR antimicrobial* OR "anti-microbial" OR "anti-microbials" OR antimicrobical* OR "anti-microbical" OR "anti-microbicals" OR antiseptic* OR "anti-septic" OR "anti-septics" OR biocid*) AND sutur*) = 57

1. **Source: ClinicalTrials.gov**

Interface / URL: https://clinicaltrials.gov/ct2/home

Database coverage dates: Information not found. ClinicalTrials.gov was created as a result of the Food and Drug Administration Modernization Act of 1997 (FDAMA). The site was made available to the public in February 2000.

Search date: 05/02/21 (all searches apart from 2 and 6); 08/02/21 (searches 2 and 6)

Retrieved records: 138

Search strategy:

The following 15 searches were conducted separately. All search terms were entered using the Expert search interface.

12 of the searches retrieved results. The 12 sets of results were imported into an empty EndNote library (302 records) and deduplicated using EndNote default de-duplication settings. 164 records were identified as duplicates and removed from the EndNote library. The remaining 138 records were retrieved for assessment.

Search 1. (suture OR sutures OR suturing OR sutured OR stitch OR stitches OR stitching OR stitched) AND (triclosan OR cgp433 OR cgp-433 OR ch3565 OR ch-3565 OR cloxifenol OR dndi1246774 OR dndi-1246774 OR dp300 OR dp-300 OR fat-80 OR fat80 OR gp41-353 OR gp41353 OR irgacare OR irgacide OR irgagard OR irgasan OR lexol-300 OR lexol300 OR ster-zac OR sterzac OR tcs OR tricosan OR cgp433R OR cgp-433R OR ch3565R OR ch-3565R OR cloxifenolR OR dndi1246774R OR dndi-1246774R OR dp300R OR dp-300R OR fat-80R OR fat80R OR gp41-353R OR gp41353R OR irgacareR OR irgacideR OR irgagardR OR irgasanR OR lexol-300R OR lexol300R OR ster-zacR OR sterzacR OR tricosanR OR cgp433TM OR cgp-433TM OR ch3565TM OR ch-3565TM OR cloxifenolTM OR dndi1246774TM OR dndi-1246774TM OR dp300TM OR dp-300TM OR fat-80TM OR fat80TM OR gp41-353TM OR gp41353TM OR irgacareTM OR irgacideTM OR irgagardTM OR irgasanTM OR lexol-300TM OR lexol300TM OR ster-zacTM OR sterzacTM OR tricosanTM OR 222-182-2 OR 3380-34-5 OR 4640-01-1 OR 4nm5039y5x OR 5174ur1dp5) = 28

Search 2. (ligate OR ligates OR ligating OR ligated OR ligature OR ligatures or loop OR loops OR looping OR looped OR thread OR threads OR threading OR threaded) AND (triclosan OR cgp433 OR cgp-433 OR ch3565 OR ch-3565 OR cloxifenol OR dndi1246774 OR dndi-1246774 OR dp300 OR dp-300 OR fat-80 OR fat80 OR gp41-353 OR gp41353 OR irgacare OR irgacide OR irgagard OR irgasan OR lexol-300 OR lexol300 OR ster-zac OR sterzac OR tcs OR tricosan OR cgp433R OR cgp-433R OR ch3565R OR ch-3565R OR cloxifenolR OR dndi1246774R OR dndi-1246774R OR dp300R OR dp-300R OR fat-80R OR fat80R OR gp41-353R OR gp41353R OR irgacareR OR irgacideR OR irgagardR OR irgasanR OR lexol-300R OR lexol300R OR ster-zacR OR sterzacR OR tricosanR OR cgp433TM OR cgp-433TM OR ch3565TM OR ch-3565TM OR cloxifenolTM OR dndi1246774TM OR dndi-1246774TM OR dp300TM OR dp-300TM OR fat-80TM OR fat80TM OR gp41-353TM OR gp41353TM OR irgacareTM OR irgacideTM OR irgagardTM OR irgasanTM OR lexol-300TM OR lexol300TM OR ster-zacTM OR sterzacTM OR tricosanTM OR 222-182-2 OR 3380-34-5 OR 4640-01-1 OR 4nm5039y5x OR 5174ur1dp5) = 7

Search 3. (approximate OR approximates OR approximating OR approximated or close OR closes OR closing OR closed OR closure OR closures OR fasten OR fastens OR fastening OR fastened or fixate OR fixates OR fixating OR fixated OR fixation OR fixations or secure OR secures OR securing OR secured) AND (triclosan OR cgp433 OR cgp-433 OR ch3565 OR ch-3565 OR cloxifenol OR dndi1246774 OR dndi-1246774 OR dp300 OR dp-300 OR fat-80 OR fat80 OR gp41-353 OR gp41353 OR irgacare OR irgacide OR irgagard OR irgasan OR lexol-300 OR lexol300 OR ster-zac OR sterzac OR tcs OR tricosan OR cgp433R OR cgp-433R OR ch3565R OR ch-3565R OR cloxifenolR OR dndi1246774R OR dndi-1246774R OR dp300R OR dp-300R OR fat-80R OR fat80R OR gp41-353R OR gp41353R OR irgacareR OR irgacideR OR irgagardR OR irgasanR OR lexol-300R OR lexol300R OR ster-zacR OR sterzacR OR tricosanR OR cgp433TM OR cgp-433TM OR ch3565TM OR ch-3565TM OR cloxifenolTM OR dndi1246774TM OR dndi-1246774TM OR dp300TM OR dp-300TM OR fat-80TM OR fat80TM OR gp41-353TM OR gp41353TM OR irgacareTM OR irgacideTM OR irgagardTM OR irgasanTM OR lexol-300TM OR lexol300TM OR ster-zacTM OR sterzacTM OR tricosanTM OR 222-182-2 OR 3380-34-5 OR 4640-01-1 OR 4nm5039y5x OR 5174ur1dp5) = 65

Search 4. (fascia OR fasciae OR fascial OR skin or skins or tissue OR tissues or wound OR wounds OR woundcare) AND (device OR devices) AND (triclosan OR cgp433 OR cgp-433 OR ch3565 OR ch-3565 OR cloxifenol OR dndi1246774 OR dndi-1246774 OR dp300 OR dp-300 OR fat-80 OR fat80 OR gp41-353 OR gp41353 OR irgacare OR irgacide OR irgagard OR irgasan OR lexol-300 OR lexol300 OR ster-zac OR sterzac OR tcs OR tricosan OR cgp433R OR cgp-433R OR ch3565R OR ch-3565R OR cloxifenolR OR dndi1246774R OR dndi-1246774R OR dp300R OR dp-300R OR fat-80R OR fat80R OR gp41-353R OR gp41353R OR irgacareR OR irgacideR OR irgagardR OR irgasanR OR lexol-300R OR lexol300R OR ster-zacR OR sterzacR OR tricosanR OR cgp433TM OR cgp-433TM OR ch3565TM OR ch-3565TM OR cloxifenolTM OR dndi1246774TM OR dndi-1246774TM OR dp300TM OR dp-300TM OR fat-80TM OR fat80TM OR gp41-353TM OR gp41353TM OR irgacareTM OR irgacideTM OR irgagardTM OR irgasanTM OR lexol-300TM OR lexol300TM OR ster-zacTM OR sterzacTM OR tricosanTM OR 222-182-2 OR 3380-34-5 OR 4640-01-1 OR 4nm5039y5x OR 5174ur1dp5) = 14

Search 5. (suture OR sutures OR suturing OR sutured OR stitch OR stitches OR stitching OR stitched) AND (antibacterial OR anti-bacterial OR antibiotic OR anti-biotic OR antiinfective OR anti-infective OR antimicrobial OR anti-microbial OR antimicrobical OR anti-microbical OR antiseptic OR anti-septic OR antibacterials OR anti-bacterials OR antibiotics OR anti-biotics OR antiinfectives OR anti-infectives OR antimicrobials OR anti-microbials OR antimicrobicals OR anti-microbicals OR antiseptics OR anti-septics OR biocide OR biocides OR biocidal) AND (coat OR coats OR coating OR coated OR impregnate OR impregnates OR impregnating OR impregnated) = 48

Search 6. (ligate OR ligates OR ligating OR ligated OR ligature OR ligatures or loop OR loops OR looping OR looped OR thread OR threads OR threading OR threaded) AND (antibacterial OR anti-bacterial OR antibiotic OR anti-biotic OR antiinfective OR anti-infective OR antimicrobial OR anti-microbial OR antimicrobical OR anti-microbical OR antiseptic OR anti-septic OR antibacterials OR anti-bacterials OR antibiotics OR anti-biotics OR antiinfectives OR anti-infectives OR antimicrobials OR anti-microbials OR antimicrobicals OR anti-microbicals OR antiseptics OR anti-septics OR biocide OR biocides OR biocidal) AND (coat OR coats OR coating OR coated OR impregnate OR impregnates OR impregnating OR impregnated) = 8

Search 7. "antibacterial suture" OR "anti-bacterial suture" OR "antibiotic suture" OR "anti-biotic suture" OR "antiinfective suture" OR "anti-infective suture" OR "antimicrobial suture" OR "anti-microbial suture" OR "antimicrobical suture" OR "anti-microbical suture" OR "antiseptic suture" OR "anti-septic suture" OR "antibacterial sutures" OR "anti-bacterial sutures" OR "antibiotic sutures" OR "anti-biotic sutures" OR "antiinfective sutures" OR "anti-infective sutures" OR "antimicrobial sutures" OR "anti-microbial sutures" OR "antimicrobical sutures" OR "anti-microbical sutures" OR "antiseptic sutures" OR "anti-septic sutures" OR "antibacterial suturing" OR "anti-bacterial suturing" OR "antibiotic suturing" OR "anti-biotic suturing" OR "antiinfective suturing" OR "anti-infective suturing" OR "antimicrobial suturing" OR "anti-microbial suturing" OR "antimicrobical suturing" OR "anti-microbical suturing" OR "antiseptic suturing" OR "anti-septic suturing" OR "antibacterial sutured" OR "anti-bacterial sutured" OR "antibiotic sutured" OR "anti-biotic sutured" OR "antiinfective sutured" OR "anti-infective sutured" OR "antimicrobial sutured" OR "anti-microbial sutured" OR "antimicrobical sutured" OR "anti-microbical sutured" OR "antiseptic sutured" OR "anti-septic sutured" = 10

Search 8. ("biocide suture" OR "biocide sutures" OR "biocide suturing" OR "biocide sutured" OR "biocidal suture" OR "biocidal sutures" OR "biocidal suturing" OR "biocidal sutured") = 0

Search 9. "plus suture" OR plus sutures" OR "plusTM suture" OR plusTM sutures" OR "plusR suture" OR plusR sutures" OR "plus sutureTM" OR plus suturesTM" OR "plus sutureR" OR "plus suturesR" OR "pds plus" OR "pds plusTM" OR "pds plusR" OR "pdsii plus" OR "pdsii plusTM" OR "pdsii plusR" OR "pds-ii plus" OR "pds-ii plusTM" OR "pds-ii plusR" OR "monocryl plus" OR "monocryl plusTM" OR "monocryl plusR" OR "vicryl plus" OR "vicryl plusTM" OR "vicryl plusR" OR stratafix OR stratafixTM OR stratafixR OR "tissue control device" OR "tissue control devices" = 52

Search 10. (pds OR pdsii OR pds-ii OR pdsTM OR pdsiiTM OR pds-iiTM OR pdsR OR pdsiiR OR pds-iiR OR monocryl OR monocrylTM OR monocrylR OR vicryl OR vicrylTM OR vicrylR) AND (triclosan OR cgp433 OR cgp-433 OR ch3565 OR ch-3565 OR cloxifenol OR dndi1246774 OR dndi-1246774 OR dp300 OR dp-300 OR fat-80 OR fat80 OR gp41-353 OR gp41353 OR irgacare OR irgacide OR irgagard OR irgasan OR lexol-300 OR lexol300 OR ster-zac OR sterzac OR tcs OR tricosan OR cgp433R OR cgp-433R OR ch3565R OR ch-3565R OR cloxifenolR OR dndi1246774R OR dndi-1246774R OR dp300R OR dp-300R OR fat-80R OR fat80R OR gp41-353R OR gp41353R OR irgacareR OR irgacideR OR irgagardR OR irgasanR OR lexol-300R OR lexol300R OR ster-zacR OR sterzacR OR tricosanR OR cgp433TM OR cgp-433TM OR ch3565TM OR ch-3565TM OR cloxifenolTM OR dndi1246774TM OR dndi-1246774TM OR dp300TM OR dp-300TM OR fat-80TM OR fat80TM OR gp41-353TM OR gp41353TM OR irgacareTM OR irgacideTM OR irgagardTM OR irgasanTM OR lexol-300TM OR lexol300TM OR ster-zacTM OR sterzacTM OR tricosanTM OR 222-182-2 OR 3380-34-5 OR 4640-01-1 OR 4nm5039y5x OR 5174ur1dp5) = 21

Search 11. (pds OR pdsii OR pds-ii OR pdsTM OR pdsiiTM OR pds-iiTM OR pdsR OR pdsiiR OR pds-iiR OR monocryl OR monocrylTM OR monocrylR OR vicryl OR vicrylTM OR vicrylR) AND (antibacterial OR anti-bacterial OR antibiotic OR anti-biotic OR antiinfective OR anti-infective OR antimicrobial OR anti-microbial OR antimicrobical OR anti-microbical OR antiseptic OR anti-septic OR antibacterials OR anti-bacterials OR antibiotics OR anti-biotics OR antiinfectives OR anti-infectives OR antimicrobials OR anti-microbials OR antimicrobicals OR anti-microbicals OR antiseptics OR anti-septics OR biocide OR biocides OR biocidal) AND (coat OR coats OR coating OR coated OR impregnate OR impregnates OR impregnating OR impregnated) = 25

Search 12. ("polydioxanon plus" OR "polydioxanone plus" OR "poliglecapron plus" OR "poliglecaprone plus" OR "polyglactin plus" OR "polyglactine plus" OR "polydioxanon plusTM" OR "polydioxanone plusTM" OR "poliglecapron plusTM" OR "poliglecaprone plusTM" OR "polyglactin plusTM" OR "polyglactine plusTM" OR "polydioxanon plusR" OR "polydioxanone plusR" OR "poliglecapron plusR" OR "poliglecaprone plusR" OR "polyglactin plusR" OR "polyglactine plusR") = 0

Search 13. ("poliglecapron 25 plus" OR "poliglecaprone 25 plus" OR "polyglactin 910 plus" OR "polyglactine 910 plus" OR "poliglecapron 25 plusTM" OR "poliglecaprone 25 plusTM" OR "polyglactin 910 plusTM" OR "polyglactine 910 plusTM" OR "poliglecapron 25 plusR" OR "poliglecaprone 25 plusR" OR "polyglactin 910 plusR" OR "polyglactine 910 plusR") = 0

Search 14. (polydioxanon OR polydioxanone OR poliglecapron OR poliglecaprone OR polyglactin OR polyglactine) AND (triclosan OR cgp433 OR cgp-433 OR ch3565 OR ch-3565 OR cloxifenol OR dndi1246774 OR dndi-1246774 OR dp300 OR dp-300 OR fat-80 OR fat80 OR gp41-353 OR gp41353 OR irgacare OR irgacide OR irgagard OR irgasan OR lexol-300 OR lexol300 OR ster-zac OR sterzac OR tcs OR tricosan OR cgp433R OR cgp-433R OR ch3565R OR ch-3565R OR cloxifenolR OR dndi1246774R OR dndi-1246774R OR dp300R OR dp-300R OR fat-80R OR fat80R OR gp41-353R OR gp41353R OR irgacareR OR irgacideR OR irgagardR OR irgasanR OR lexol-300R OR lexol300R OR ster-zacR OR sterzacR OR tricosanR OR cgp433TM OR cgp-433TM OR ch3565TM OR ch-3565TM OR cloxifenolTM OR dndi1246774TM OR dndi-1246774TM OR dp300TM OR dp-300TM OR fat-80TM OR fat80TM OR gp41-353TM OR gp41353TM OR irgacareTM OR irgacideTM OR irgagardTM OR irgasanTM OR lexol-300TM OR lexol300TM OR ster-zacTM OR sterzacTM OR tricosanTM OR 222-182-2 OR 3380-34-5 OR 4640-01-1 OR 4nm5039y5x OR 5174ur1dp5) = 12

Search 15. (polydioxanon OR polydioxanone OR poliglecapron OR poliglecaprone OR polyglactin OR polyglactine) AND (antibacterial OR anti-bacterial OR antibiotic OR anti-biotic OR antiinfective OR anti-infective OR antimicrobial OR anti-microbial OR antimicrobical OR anti-microbical OR antiseptic OR anti-septic OR antibacterials OR anti-bacterials OR antibiotics OR anti-biotics OR antiinfectives OR anti-infectives OR antimicrobials OR anti-microbials OR antimicrobicals OR anti-microbicals OR antiseptics OR anti-septics OR biocide OR biocides OR biocidal) AND (coat OR coats OR coating OR coated OR impregnate OR impregnates OR impregnating OR impregnated) = 12

Search note: ClinicalTrials.gov has relatively limited search functionality compared to Ovid MEDLINE. Basic and more advanced functionality such as truncation or proximity operators is not available. In the context of this functionality, attempting to translate the element of the MEDLINE strategy that combined non-specific wound closure terms with non-specific antibacterial coating terms for ClinicalTrials.gov was judged to be an inefficient search approach. In this context it was felt appropriate to focus the ClinicalTrials.gov search on retrieval of records that included terms known to be found in database records for relevant studies.

1. **Source: WHO International Clinical Trials Registry Portal (ICTRP)**

Interface / URL: http://apps.who.int/trialsearch/Default.aspx

Database coverage dates: Information not found. Data sets from data providers are updated every Friday evening according to a schedule. On the date of search, files had been imported from data providers between January 2021 and February 2021.

Search date: 05/02/21

Retrieved records: 84

Search strategy:

The following 31 searches were conducted separately using the search interface at: <https://apps.who.int/trialsearch/>

For all searches 'Without synonyms' was selected.

The search help page ('Search Tips') was not accessible on the day of search.

16 of the searches retrieved results. The 16 sets of results were imported into an empty EndNote Library (175 records) and deduplicated using Endnote default settings. 91 results were identified as duplicates and removed from the Endnote library. The remaining 84 results were retrieved for assessment.

Search 1. sutur* AND triclosan* OR stitch* AND triclosan* OR ligat* AND triclosan* OR loop* AND triclosan* OR thread* AND triclosan* OR sutur* AND tcs OR stitch* AND tcs OR ligat* AND tcs OR loop* AND tcs OR thread* AND tcs = 32 (33 records for 32 trials found)

Search 2. approximat* AND triclosan* OR clos* AND triclosan* OR fasten* AND triclosan* OR fixat* AND triclosan* OR secur* AND triclosan* OR approximat* AND tcs OR clos* AND tcs OR fasten* AND tcs OR fixat* AND tcs OR secur* AND tcs = 14 records for 14 trials found

Search 3. device* AND triclosan* OR device* AND tcs = 3 records for 3 trials found

Search 4. cgp433* OR cgp-433* OR ch3565* OR ch-3565* OR cloxifenol* OR dndi1246774* OR dndi-1246774* OR dp300* OR dp-300* OR fat-80* OR fat80* OR gp41-353* OR gp41353* OR irgacare* OR irgacide* OR irgagard* OR irgasan* OR lexol-300* OR lexol300* OR ster-zac* OR sterzac* OR tricosan* OR 222-182-2 OR 3380-34-5 OR 4640-01-1 OR 4nm5039y5x OR 5174ur1dp5 = 8 records for 8 trials found

Search 5. sutur* AND antibacterial* AND coat* OR sutur* AND anti-bacterial* AND coat* OR sutur* AND antibiotic* AND coat* OR sutur* AND anti-biotic* AND coat* OR sutur* AND antiinfective* AND coat* OR sutur* AND anti-infective* AND coat* OR sutur* AND antimicrobial* AND coat* OR sutur* AND anti-microbial* AND coat* OR sutur* AND antimicrobical* AND coat* OR sutur* AND anti-microbical* AND coat* OR sutur* AND antiseptic* AND coat* OR sutur* AND anti-septic* AND coat* OR sutur* AND biocid* AND coat* = 16 records for 16 trials found

Search 6. sutur* AND antibacterial* AND impregnat* OR sutur* AND anti-bacterial* AND impregnat* OR sutur* AND antibiotic* AND impregnat* OR sutur* AND anti-biotic* AND impregnat* OR sutur* AND antiinfective* AND impregnat* OR sutur* AND anti-infective* AND impregnat* OR sutur* AND antimicrobial* AND impregnat* OR sutur* AND anti-microbial* AND impregnat* OR sutur* AND antimicrobical* AND impregnat* OR sutur* AND anti-microbical* AND impregnat* OR sutur* AND antiseptic* AND impregnat* OR sutur* AND anti-septic* AND impregnat* OR sutur* AND biocid* AND impregnat* = 4 records for 4 trials found

Search 7. stitch* AND antibacterial* AND coat* OR stitch* AND anti-bacterial* AND coat* OR stitch* AND antibiotic* AND coat* OR stitch* AND anti-biotic* AND coat* OR stitch* AND antiinfective* AND coat* OR stitch* AND anti-infective* AND coat* OR stitch* AND antimicrobial* AND coat* OR stitch* AND anti-microbial* AND coat* OR stitch* AND antimicrobical* AND coat* OR stitch* AND anti-microbical* AND coat* OR stitch* AND antiseptic* AND coat* OR stitch* AND anti-septic* AND coat* OR stitch* AND biocid* AND coat* = 1 trial found

Search 8. stitch* AND antibacterial* AND impregnat* OR stitch* AND anti-bacterial* AND impregnat* OR stitch* AND antibiotic* AND impregnat* OR stitch* AND anti-biotic* AND impregnat* OR stitch* AND antiinfective* AND impregnat* OR stitch* AND anti-infective* AND impregnat* OR stitch* AND antimicrobial* AND impregnat* OR stitch* AND anti-microbial* AND impregnat* OR stitch* AND antimicrobical* AND impregnat* OR stitch* AND anti-microbical* AND impregnat* OR stitch* AND antiseptic* AND impregnat* OR stitch* AND anti-septic* AND impregnat* OR stitch* AND biocid* AND impregnat* = 0

Search 9. ligat* AND antibacterial* AND coat* OR ligat* AND anti-bacterial* AND coat* OR ligat* AND antibiotic* AND coat* OR ligat* AND anti-biotic* AND coat* OR ligat* AND antiinfective* AND coat* OR ligat* AND anti-infective* AND coat* OR ligat* AND antimicrobial* AND coat* OR ligat* AND anti-microbial* AND coat* OR ligat* AND antimicrobical* AND coat* OR ligat* AND anti-microbical* AND coat* OR ligat* AND antiseptic* AND coat* OR ligat* AND anti-septic* AND coat* OR ligat* AND biocid* AND coat* = 0

Search 10. ligat* AND antibacterial* AND impregnat* OR ligat* AND anti-bacterial* AND impregnat* OR ligat* AND antibiotic* AND impregnat* OR ligat* AND anti-biotic* AND impregnat* OR ligat* AND antiinfective* AND impregnat* OR ligat* AND anti-infective* AND impregnat* OR ligat* AND antimicrobial* AND impregnat* OR ligat* AND anti-microbial* AND impregnat* OR ligat* AND antimicrobical* AND impregnat* OR ligat* AND anti-microbical* AND impregnat* OR ligat* AND antiseptic* AND impregnat* OR ligat* AND anti-septic* AND impregnat* OR ligat* AND biocid* AND impregnat* = 0

Search 11. loop* AND antibacterial* AND coat* OR loop* AND anti-bacterial* AND coat* OR loop* AND antibiotic* AND coat* OR loop* AND anti-biotic* AND coat* OR loop* AND antiinfective* AND coat* OR loop* AND anti-infective* AND coat* OR loop* AND antimicrobial* AND coat* OR loop* AND anti-microbial* AND coat* OR loop* AND antimicrobical* AND coat* OR loop* AND anti-microbical* AND coat* OR loop* AND antiseptic* AND coat* OR loop* AND anti-septic* AND coat* OR loop* AND biocid* AND coat* = 0

Search 12. loop* AND antibacterial* AND impregnat* OR loop* AND anti-bacterial* AND impregnat* OR loop* AND antibiotic* AND impregnat* OR loop* AND anti-biotic* AND impregnat* OR loop* AND antiinfective* AND impregnat* OR loop* AND anti-infective* AND impregnat* OR loop* AND antimicrobial* AND impregnat* OR loop* AND anti-microbial* AND impregnat* OR loop* AND antimicrobical* AND impregnat* OR loop* AND anti-microbical* AND impregnat* OR loop* AND antiseptic* AND impregnat* OR loop* AND anti-septic* AND impregnat* OR loop* AND biocid* AND impregnat* = 0

Search 13. thread* AND antibacterial* AND coat* OR thread* AND anti-bacterial* AND coat* OR thread* AND antibiotic* AND coat* OR thread* AND anti-biotic* AND coat* OR thread* AND antiinfective* AND coat* OR thread* AND anti-infective* AND coat* OR thread* AND antimicrobial* AND coat* OR thread* AND anti-microbial* AND coat* OR thread* AND antimicrobical* AND coat* OR thread* AND anti-microbical* AND coat* OR thread* AND antiseptic* AND coat* OR thread* AND anti-septic* AND coat* OR thread* AND biocid* AND coat* = 0

Search 14. thread* AND antibacterial* AND impregnat* OR thread* AND anti-bacterial* AND impregnat* OR thread* AND antibiotic* AND impregnat* OR thread* AND anti-biotic* AND impregnat* OR thread* AND antiinfective* AND impregnat* OR thread* AND anti-infective* AND impregnat* OR thread* AND antimicrobial* AND impregnat* OR thread* AND anti-microbial* AND impregnat* OR thread* AND antimicrobical* AND impregnat* OR thread* AND anti-microbical* AND impregnat* OR thread* AND antiseptic* AND impregnat* OR thread* AND anti-septic* AND impregnat* OR thread* AND biocid* AND impregnat* = 1 trial found

Search 15. antibacterial sutur* OR anti-bacterial sutur* OR antibiotic sutur* OR anti-biotic sutur* OR antiinfective sutur* OR anti-infective sutur* OR antimicrobial sutur* OR anti-microbial sutur* OR antimicrobical sutur* OR anti-microbical sutur* OR antiseptic sutur* OR anti-septic sutur* OR biocide sutur* OR biocidal sutur* = 14 records for 14 trials found

Search 16. plus suture* OR plusTM suture* OR plusR suture* OR pds plus* OR pdsii plus* OR pds-ii plus* OR monocryl plus* OR vicryl plus* OR stratafix* OR tissue control device* = 47 records for 46 trials found

Search 17. pds* AND triclosan* OR pds* AND tcs OR monocryl* AND triclosan* OR monocryl* AND tcs OR vicryl* AND triclosan* OR vicryl* AND tcs = 19 records for 18 trials found

Search 18. pds* AND antibacterial* AND coat* OR pds* AND anti-bacterial* AND coat* OR pds* AND antibiotic* AND coat* OR pds* AND anti-biotic* AND coat* OR pds* AND antiinfective* AND coat* OR pds* AND anti-infective* AND coat* OR pds* AND antimicrobial* AND coat* OR pds* AND anti-microbial* AND coat* OR pds* AND antimicrobical* AND coat* OR pds* AND anti-microbical* AND coat* OR pds* AND antiseptic* AND coat* OR pds* AND anti-septic* AND coat* OR pds* AND biocid* AND coat* = 2 records for 2 trials found

Search 19. monocryl* AND antibacterial* AND coat* OR monocryl* AND anti-bacterial* AND coat* OR monocryl* AND antibiotic* AND coat* OR monocryl* AND anti-biotic* AND coat* OR monocryl* AND antiinfective* AND coat* OR monocryl* AND anti-infective* AND coat* OR monocryl* AND antimicrobial* AND coat* OR monocryl* AND anti-microbial* AND coat* OR monocryl* AND antimicrobical* AND coat* OR monocryl* AND anti-microbical* AND coat* OR monocryl* AND antiseptic* AND coat* OR monocryl* AND anti-septic* AND coat* OR monocryl* AND biocid* AND coat* = 2 records for 2 trials found

Search 20. vicryl* AND antibacterial* AND coat* OR vicryl* AND anti-bacterial* AND coat* OR vicryl* AND antibiotic* AND coat* OR vicryl* AND anti-biotic* AND coat* OR vicryl* AND antiinfective* AND coat* OR vicryl* AND anti-infective* AND coat* OR vicryl* AND antimicrobial* AND coat* OR vicryl* AND anti-microbial* AND coat* OR vicryl* AND antimicrobical* AND coat* OR vicryl* AND anti-microbical* AND coat* OR vicryl* AND antiseptic* AND coat* OR vicryl* AND anti-septic* AND coat* OR vicryl* AND biocid* AND coat* = 9 records for 9 trials found

Search 21. pds* AND antibacterial* AND impregnat* OR pds* AND anti-bacterial* AND impregnat* OR pds* AND antibiotic* AND impregnat* OR pds* AND anti-biotic* AND impregnat* OR pds* AND antiinfective* AND impregnat* OR pds* AND anti-infective* AND impregnat* OR pds* AND antimicrobial* AND impregnat* OR pds* AND anti-microbial* AND impregnat* OR pds* AND antimicrobical* AND impregnat* OR pds* AND anti-microbical* AND impregnat* OR pds* AND antiseptic* AND impregnat* OR pds* AND anti-septic* AND impregnat* OR pds* AND biocid* AND impregnat* = 0

Search 22. monocryl* AND antibacterial* AND impregnat* OR monocryl* AND anti-bacterial* AND impregnat* OR monocryl* AND antibiotic* AND impregnat* OR monocryl* AND anti-biotic* AND impregnat* OR monocryl* AND antiinfective* AND impregnat* OR monocryl* AND anti-infective* AND impregnat* OR monocryl* AND antimicrobial* AND impregnat* OR monocryl* AND anti-microbial* AND impregnat* OR monocryl* AND antimicrobical* AND impregnat* OR monocryl* AND anti-microbical* AND impregnat* OR monocryl* AND antiseptic* AND impregnat* OR monocryl* AND anti-septic* AND impregnat* OR monocryl* AND biocid* AND impregnat* = 0

Search 23. vicryl* AND antibacterial* AND impregnat* OR vicryl* AND anti-bacterial* AND impregnat* OR vicryl* AND antibiotic* AND impregnat* OR vicryl* AND anti-biotic* AND impregnat* OR vicryl* AND antiinfective* AND impregnat* OR vicryl* AND anti-infective* AND impregnat* OR vicryl* AND antimicrobial* AND impregnat* OR vicryl* AND anti-microbial* AND impregnat* OR vicryl* AND antimicrobical* AND impregnat* OR vicryl* AND anti-microbical* AND impregnat* OR vicryl* AND antiseptic* AND impregnat* OR vicryl* AND anti-septic* AND impregnat* OR vicryl* AND biocid* AND impregnat* = 0

Search 24. polydioxanon plus* OR polydioxanone plus* OR poliglecapron plus* OR poliglecaprone plus* OR polyglactin plus* OR polyglactine plus* OR poliglecapron 25 plus* OR poliglecaprone 25 plus* OR polyglactin 910 plus* OR polyglactine 910 plus* = 0

Search 25. polydioxanon* AND triclosan* OR polydioxanon* AND tcs OR poliglecapron* AND triclosan* OR poliglecapron* AND tcs OR poliglecapron* AND triclosan* OR poliglecapron* AND tcs = 1 trial found

Search 26. polydioxanon* AND antibacterial* AND coat* OR polydioxanon* AND anti-bacterial* AND coat* OR polydioxanon* AND antibiotic* AND coat* OR polydioxanon* AND anti-biotic* AND coat* OR polydioxanon* AND antiinfective* AND coat* OR polydioxanon* AND anti-infective* AND coat* OR polydioxanon* AND antimicrobial* AND coat* OR polydioxanon* AND anti-microbial* AND coat* OR polydioxanon* AND antimicrobical* AND coat* OR polydioxanon* AND anti-microbical* AND coat* OR polydioxanon* AND antiseptic* AND coat* OR polydioxanon* AND anti-septic* AND coat* OR polydioxanon* AND biocid* AND coat* = 0

Search 27. poliglecapron* AND antibacterial* AND coat* OR poliglecapron* AND anti-bacterial* AND coat* OR poliglecapron* AND antibiotic* AND coat* OR poliglecapron* AND anti-biotic* AND coat* OR poliglecapron* AND antiinfective* AND coat* OR poliglecapron* AND anti-infective* AND coat* OR poliglecapron* AND antimicrobial* AND coat* OR poliglecapron* AND anti-microbial* AND coat* OR poliglecapron* AND antimicrobical* AND coat* OR poliglecapron* AND anti-microbical* AND coat* OR poliglecapron* AND antiseptic* AND coat* OR poliglecapron* AND anti-septic* AND coat* OR poliglecapron* AND biocid* AND coat* = 0

Search 28. polyglactin* AND antibacterial* AND coat* OR polyglactin* AND anti-bacterial* AND coat* OR polyglactin* AND antibiotic* AND coat* OR polyglactin* AND anti-biotic* AND coat* OR polyglactin* AND antiinfective* AND coat* OR polyglactin* AND anti-infective* AND coat* OR polyglactin* AND antimicrobial* AND coat* OR polyglactin* AND anti-microbial* AND coat* OR polyglactin* AND antimicrobical* AND coat* OR polyglactin* AND anti-microbical* AND coat* OR polyglactin* AND antiseptic* AND coat* OR polyglactin* AND anti-septic* AND coat* OR polyglactin* AND biocid* AND coat* = 4 records for 4 trials found

Search 29. polydioxanon* AND antibacterial* AND impregnat* OR polydioxanon* AND anti-bacterial* AND impregnat* OR polydioxanon* AND antibiotic* AND impregnat* OR polydioxanon* AND anti-biotic* AND impregnat* OR polydioxanon* AND antiinfective* AND impregnat* OR polydioxanon* AND anti-infective* AND impregnat* OR polydioxanon* AND antimicrobial* AND impregnat* OR polydioxanon* AND anti-microbial* AND impregnat* OR polydioxanon* AND antimicrobical* AND impregnat* OR polydioxanon* AND anti-microbical* AND impregnat* OR polydioxanon* AND antiseptic* AND impregnat* OR polydioxanon* AND anti-septic* AND impregnat* OR polydioxanon* AND biocid* AND impregnat* = 0

Search 30. poliglecapron* AND antibacterial* AND impregnat* OR poliglecapron* AND anti-bacterial* AND impregnat* OR poliglecapron* AND antibiotic* AND impregnat* OR poliglecapron* AND anti-biotic* AND impregnat* OR poliglecapron* AND antiinfective* AND impregnat* OR poliglecapron* AND anti-infective* AND impregnat* OR poliglecapron* AND antimicrobial* AND impregnat* OR poliglecapron* AND anti-microbial* AND impregnat* OR poliglecapron* AND antimicrobical* AND impregnat* OR poliglecapron* AND anti-microbical* AND impregnat* OR poliglecapron* AND antiseptic* AND impregnat* OR poliglecapron* AND anti-septic* AND impregnat* OR poliglecapron* AND biocid* AND impregnat* = 0

Search 31. polyglactin* AND antibacterial* AND impregnat* OR polyglactin* AND anti-bacterial* AND impregnat* OR polyglactin* AND antibiotic* AND impregnat* OR polyglactin* AND anti-biotic* AND impregnat* OR polyglactin* AND antiinfective* AND impregnat* OR polyglactin* AND anti-infective* AND impregnat* OR polyglactin* AND antimicrobial* AND impregnat* OR polyglactin* AND anti-microbial* AND impregnat* OR polyglactin* AND antimicrobical* AND impregnat* OR polyglactin* AND anti-microbical* AND impregnat* OR polyglactin* AND antiseptic* AND impregnat* OR polyglactin* AND anti-septic* AND impregnat* OR polyglactin* AND biocid* AND impregnat* = 0

Search note: ICTRP has relatively limited search functionality compared to Ovid MEDLINE. Basic and more advanced functionality such as proximity operators or grouping sets of terms using parentheses is not available. In the context of this functionality, attempting to translate the element of the MEDLINE strategy that combined non-specific wound closure terms with non-specific antibacterial coating terms for ICTRP was judged to be an inefficient search approach. In this context it was felt appropriate to focus the ICTRP search on retrieval of records that included terms known to be found in database records for relevant studies.

1. **Source: National Institute for Health Research (NIHR) Be Part of Research**

Interface / URL: https://bepartofresearch.nihr.ac.uk/

Database coverage dates: Information not found

Search date: 05/02/21

Retrieved records: 0

Search strategy:

No search help pages were identified. Test searches indicated that:

- Boolean OR is supported
- Boolean AND is supported
- Truncation using * is supported

The following 16 searches were conducted separately. Returned results were screening by the Information Specialist for relevance to the eligible interventions. Potentially relevant studies were retrieved for further consideration.

Search 1. triclosan* = 0 returned

Search 2. cgp433* OR cgp-433* OR ch3565* OR ch-3565* = 0 returned

Search 3. cloxifenol* OR dndi1246774* OR dndi-1246774* = 0 returned

Search 4. dp300* OR dp-300* OR fat-80* OR fat80* OR gp41-353* OR gp41353* = 0 returned

Search 5. irgacare* OR irgacide* OR irgagard* OR irgasan* = 0 returned

Search 6. lexol-300* OR lexol300* OR ster-zac* OR sterzac* = 0 returned

Search 7. tcs OR tricosan* = 0 returned

Search 8. 222-182-2 OR 3380-34-5 OR 4640-01-1 = 0 returned

Search 9. 4nm5039y5x OR 5174ur1dp5 = 0 returned

Search 10. coat* = 22 returned, 0 retrieved

Search 11. impregnat* = 1 returned, 0 retrieved

Search 12. sutur* = 11 returned, 0 retrieved

Search 13. pds* = 2 returned, 0 retrieved

Search 14. monocryl* OR vicryl* OR stratafix* = 0 returned

Search 15. tissue control device* = 2 returned, 0 retrieved

Search 16. polydioxanon* OR poliglecapron* OR polyglactin* = 0 returned

0 records were retrieved for further consideration

1. **Source: IDEAS**

Interface / URL: https://ideas.repec.org/

Database coverage dates: Information not found

Search date: 08/02/21

Retrieved records: 0

Search strategy:

No help pages were found with detailed information on search functionality. Test searches indicated that:

- truncation and Boolean OR are not supported
- Boolean AND is automatically inserted between search terms
- phrase searches using "" are supported

The following searches were conducted separately. Returned results were screening by the Information Specialist for relevance to the eligible interventions. Potentially relevant studies were checked against results retrieved already via other search sources – duplicates were excluded. Remaining relevant results were retrieved for further consideration.

triclosan = 0 retrieved (12 returned)

cgp433 = 0 returned

"cgp-433" = 0 returned

ch3565 = 0 returned

"ch-3565" = 0 returned

cloxifenol = 0 returned

dndi1246774 = 0 returned

"dndi-1246774" = 0 returned

dp300 = 0 returned

"dp-300" = 0 returned

"fat-80" = 0 returned

fat80 = 0 returned

"gp41-353" = 0 returned

gp41353 = 0 returned

irgacare = 0 returned

irgacide = 0 returned

irgagard = 0 returned

irgasan = 0 returned

"lexol-300" = 0 returned

lexol300 = 0 returned

"ster-zac" = 0 returned

sterzac = 0 returned

tricosan = 0 returned

cgp433r = 0 returned

"cgp-433r" = 0 returned

ch3565r = 0 returned

"ch-3565r" = 0 returned

cloxifenolr = 0 returned

dndi1246774r = 0 returned

"dndi-1246774r" = 0 returned

dp300r = 0 returned

"dp-300r" = 0 returned

"fat-80r" = 0 returned

fat80r = 0 returned

"gp41-353r" = 0 returned

gp41353r = 0 returned

irgacarer = 0 returned

irgacider = 0 returned

irgagardr = 0 returned

irgasanr = 0 returned

"lexol-300r" = 0 returned

lexol300r = 0 returned

"ster-zacr" = 0 returned

sterzacr = 0 returned

tricosanr = 0 returned

cgp433tm = 0 returned

"cgp-433tm" = 0 returned

ch3565tm = 0 returned

"ch-3565tm" = 0 returned

cloxifenoltm = 0 returned

dndi1246774tm = 0 returned

"dndi-1246774tm" = 0 returned

dp300tm = 0 returned

"dp-300tm" = 0 returned

"fat-80tm" = 0 returned

fat80tm = 0 returned

"gp41-353tm" = 0 returned

gp41353tm = 0 returned

irgacaretm = 0 returned

irgacidetm = 0 returned

irgagardtm = 0 returned

irgasantm = 0 returned

"lexol-300tm" = 0 returned

lexol300tm = 0 returned

"ster-zactm" = 0 returned

sterzactm = 0 returned

tricosantm = 0 returned

"222-182-2" = 0 returned

"3380-34-5" = 0 returned

"4640-01-1" = 0 returned

4nm5039y5x = 0 returned

5174ur1dp5 = 0 returned

tcs suture = 0 returned

tcs sutures = 0 returned

tcs suturing = 0 returned

tcs stitch = 0 returned

tcs stitches = 0 returned

tcs stitching = 0 returned

tcs loop = 0 retrieved (3 returned)

tcs loops = 0 retrieved (1 returned)

tcs looping = 0 retrieved (2 returned)

tcs looped = 0 returned

tcs thread = 0 returned

tcs threads = 0 returned

tcs threading = 0 returned

tcs threaded = 0 returned

suture coat = 0 returned

suture coats = 0 returned

suture coating = 0 retrieved (1 returned)

suture coated = 0 retrieved (1 returned)

suture impregnate = 0 returned

suture impregnates = 0 returned

suture impregnating = 0 returned

suture impregnated = 0 returned

sutures coat = 0 returned

sutures coats = 0 returned

sutures coating = 0 returned

sutures coated = 0 returned

sutures impregnate = 0 returned

sutures impregnates = 0 returned

sutures impregnating = 0 returned

sutures impregnated = 0 returned

suturing coat = 0 returned

suturing coats = 0 returned

suturing coating = 0 returned

suturing coated = 0 returned

suturing impregnate = 0 returned

suturing impregnates = 0 returned

suturing impregnating = 0 returned

suturing impregnated = 0 returned

sutured = 0 retrieved (7 returned)

stitch coat = 0 returned

stitch coats = 0 returned

stitch coating = 0 retrieved (2 returned)

stitch coated = 0 returned

stitch impregnate = 0 returned

stitch impregnates = 0 returned

stitch impregnating = 0 returned

stitch impregnated = 0 returned

stitches coat = 0 returned

stitches coats = 0 returned

stitches coating = 0 returned

stitches coated = 0 returned

stitches impregnate = 0 returned

stitches impregnates = 0 returned

stitches impregnating = 0 returned

stitches impregnated = 0 returned

stitching coat = 0 returned

stitching coats = 0 returned

stitching coating = 0 returned

stitching coated = 0 returned

stitching impregnate = 0 returned

stitching impregnates = 0 returned

stitching impregnating = 0 returned

stitching impregnated = 0 returned

stitched = 0 retrieved (36 returned)

ligate = 0 returned

ligates = 0 returned

ligating = 0 retrieved (3 returned)

ligated = 0 retrieved (5 returned)

ligature = 0 retrieved (4 returned)

ligatures = 0 retrieved (5 returned)

loop coat = 0 retrieved (1 returned)

loop coats = 0 returned

loop coating = 0 retrieved (4 returned)

loop coated = 0 retrieved (2 returned)

loop impregnate = 0 returned

loop impregnates = 0 returned

loop impregnating = 0 returned

loop impregnated = 0 returned

loops coat = 0 returned

loops coats = 0 returned

loops coating = 0 retrieved (2 returned)

loops coated = 0 retrieved (1 returned)

loops impregnate = 0 returned

loops impregnates = 0 returned

loops impregnating = 0 returned

loops impregnated = 0 returned

looping coat = 0 returned

looping coats = 0 returned

looping coating = 0 retrieved (3 returned)

looping coated = 0 retrieved (2 returned)

looping impregnate = 0 returned

looping impregnates = 0 returned

looping impregnating = 0 returned

looping impregnated = 0 retrieved (6 returned)

looped coat = 0 returned

looped coats = 0 returned

looped coating = 0 returned

looped coated = 0 returned

looped impregnate = 0 returned

looped impregnates = 0 returned

looped impregnating = 0 returned

looped impregnated = = 0 returned

thread coat = 0 returned

thread coats = 0 retrieved (5 returned)

thread coating = 0 retrieved (2 returned)

thread coated = 0 returned

thread impregnate = 0 returned

thread impregnates = 0 returned

thread impregnating = 0 returned

thread impregnated = 0 retrieved (1 returned)

threads coat = 0 returned

threads coats = 0 returned

threads coating = 0 returned

threads coated = 0 returned

threads impregnate = 0 returned

threads impregnates = 0 returned

threads impregnating = 0 returned

threads impregnated = 0 returned

threading coat = 0 returned

threading coats = 0 returned

threading coating = 0 returned

threading coated = 0 returned

threading impregnate = 0 returned

threading impregnates = 0 returned

threading impregnating = 0 returned

threading impregnated = 0 returned

threaded coat = 0 returned

threaded coats = 0 returned

threaded coating = 0 retrieved (1 returned)

threaded coated = 0 returned

threaded impregnate = 0 returned

threaded impregnates = 0 returned

threaded impregnating = 0 returned

threaded impregnated = 0 returned

plus suture = 0 returned

plus sutures = 0 returned

plusR = 0 returned

plusTM = 0 returned

sutureR = 0 returned

sutureTM = 0 returned

"antibacterial suture"= 0 returned

"anti-bacterial suture" = 0 returned

"antibiotic suture" = 0 returned

"anti-biotic suture" = 0 returned

"antiinfective suture" = 0 returned

"anti-infective suture" = 0 returned

"antimicrobial suture" = 0 returned

"anti-microbial suture" = 0 returned

"antimicrobical suture" = 0 returned

"anti-microbical suture" = 0 returned

"antiseptic suture" = 0 returned

"anti-septic suture" = 0 returned

"antibacterial sutures"= 0 returned

"anti-bacterial sutures" = 0 returned

"antibiotic sutures" = 0 returned

"anti-biotic sutures" = 0 returned

"antiinfective sutures" = 0 returned

"anti-infective sutures" = 0 returned

"antimicrobial sutures" = 0 returned

"anti-microbial sutures" = 0 returned

"antimicrobical sutures" = 0 returned

"anti-microbical sutures" = 0 returned

"antiseptic sutures" = 0 returned

"anti-septic sutures"= 0 returned

"antibacterial suturing"= 0 returned

"anti-bacterial suturing" = 0 returned

"antibiotic suturing" = 0 returned

"anti-biotic suturing" = 0 returned

"antiinfective suturing" = 0 returned

"anti-infective suturing" = 0 returned

"antimicrobial suturing" = 0 returned

"anti-microbial suturing" = 0 returned

"antimicrobical suturing" = 0 returned

"anti-microbical suturing" = 0 returned

"antiseptic suturing" = 0 returned

"anti-septic suturing" = 0 retrieved

"biocide suture"= 0 returned

"biocide sutures"= 0 returned

"biocide suturing"= 0 returned

"biocidal suture"= 0 returned

"biocidal sutures"= 0 returned

"biocidal suturing" = 0 returned

pds plus = 0 retrieved (1 returned)

pdsR = 0 retrieved (1 returned)

pdsTM = 0 returned

"pds-ii" = 0 returned

"pds-iiR" = 0 returned

"pds-iiTM" = 0 returned

"pdsii" = 0 returned

"pdsiiR" = 0 returned

"pdsiiTM" = 0 returned

monocryl = 0 returned

monocrylR = 0 returned

monocrylTM = 0 returned

vicryl = 0 retrieved (2 returned)

vicrylR = 0 returned

vicrylTM = 0 returned

stratafix = 0 returned

stratafixR = 0 returned

stratafixTM = 0 returned

"tissue control device" = 0 returned

"tissue control devices" = 0 returned

polydioxanon = 0 returned

polydioxanone = 0 returned

poliglecapron = 0 returned

poliglecaprone = 0 returned

polyglactin = 0 returned

polyglactine = 0 returned

0 results were retrieved

Search note: IDEAS has relatively limited search functionality compared to Ovid MEDLINE. Basic and more advanced functionality such as Boolean OR, proximity operators or grouping sets of terms using parentheses is not available. In the context of this functionality, attempting to translate the element of the MEDLINE strategy that combined non-specific wound closure terms with non-specific antibacterial coating terms for IDEAS was judged to be an inefficient search approach. In this context it was felt appropriate to focus the IDEAS search on retrieval of records that included terms known to be found in database records for relevant studies.
